# Family genetic designs in MoBa provide insights into health and functioning

**DOI:** 10.1038/s41586-026-10926-5

**Published:** 2026-08-19

**Authors:** Elizabeth C. Corfield, Alexey A. Shadrin, Oleksandr Frei, Zillur Rahman, Bayram Cevdet Akdeniz, Tahir Tekin Filiz, Aihua Lin, Isabella Badini, Laura Hegemann, Lavinia Athanasiu, Robyn E. Wootton, Chloe Austerberry, Amanda M. Hughes, Martin Tesli, Espen Hagen, Ragnhild E. Brandlistuen, Espen Moen Eilertsen, Lars T. Westlye, Pål R. Njølstad, Per Magnus, Eivind Hovig, Tetyana Zayats, Helga Ask, Ted Reichborn-Kjennerud, Gibran Hemani, Neil M. Davies, Laurie J. Hannigan, Ole A. Andreassen, Alexandra Karoline Havdahl

**Affiliations:** 1 PsychGen Centre for Genetic Epidemiology and Mental Health, Norwegian Institute of Public Health, Oslo, Norway; 2 Psychiatric Genetic Epidemiology Group, Research Department & Nic Waals Institute, Lovisenberg Diaconal Hospital, Oslo, Norway; 3 MRC Integrative Epidemiology Unit, Bristol Medical School, University of Bristol, Bristol, UK; 4 Population Health Sciences, Bristol Medical School, University of Bristol, Bristol, UK; 5 Centre for Precision Psychiatry, Division of Mental Health and Addiction, Oslo University Hospital and University of Oslo, Oslo, Norway; 6 KG Jebsen Centre for Neurodevelopmental disorders, University of Oslo, Oslo, Norway; 7 Department of Pharmacy, University of Oslo, Oslo, Norway; 8 Department of Tumor biology, Institute for Cancer Research, Oslo University Hospital, Oslo, Norway; 9 Division of Psychiatry, University College London, London, UK; 10 School of Psychological Science, University of Bristol, Bristol, UK; 11 Research Department of Clinical, Educational and Health Psychology, University College London, London, UK; 12 Centre for Child, Adolescent and Family Research, University of Cambridge, Cambridge, UK; 13 Department of Mental Health and Suicide, Norwegian Institute of Public Health, Oslo, Norway; 14 Division of Mental Health and Addiction, Oslo University Hospital, Oslo, Norway; 15 Promenta Research Center, Department of Psychology, University of Oslo, Oslo, Norway; 16 Department of Psychology, University of Oslo, Oslo, Norway; 17 Department of Clinical Science, University of Bergen, Bergen, Norway; 18 Department of Children and Adolescent Medicine, Haukeland University Hospital, Bergen, Norway; 19 Centre for Fertility and Health, Norwegian Institute of Public Health, Oslo, Norway; 20 Department of Informatics University of Oslo, Oslo, Norway; 21 Analytic and Translational Unit, Massachusetts General Hospital, Boston, MA USA; 22 Stanley Center for Psychiatric Disorders, Broad Institute of MIT and Harvard, Boston, MA USA; 23 Department of Child Health and Development, Norwegian Institute of Public Health, Oslo, Norway; 24 Institute of Clinical Medicine, University of Oslo, Oslo, Norway; 25 Department of Statistical Science, University College London, London, UK; 26 Department of Public Health and Nursing, Norwegian University of Science and Technology, Trondheim, Norway

**Keywords:** Population genetics, Behavioural genetics, Predictive markers, Risk factors, Signs and symptoms

## Abstract

Genome-wide association studies using large, population-based samples of unrelated individuals have discovered thousands of genetic associations with health and disease^1^. These studies can help explain genetic and environmental risks. However, increasing evidence suggests that population-based estimates, while precise, can also reflect confounding that affects their use and interpretation. This confounding can be overcome using data from genotyped family members, such as nuclear mother–father–child trios^2,3^. However, samples of genotyped families are rare^4–11^. Here we illustrate some of the advantages of familial data using the Norwegian Mother, Father and Child Cohort Study (MoBa), a population-based cohort of parents and offspring with extensive genotype data (*n* **≈** 230,000) (ref. ^3^), along with broad and longitudinal phenotyping of health and functioning. We provide an overview of MoBa and describe the quality control of genotype data tailored to this extensively related sample. We then use trio data to illustrate how family-based genomic designs can identify distinct direct and indirect sources of genetic influence and structural confounding. As examples, we analyse children’s height, educational achievement, depressive symptoms and sleep duration. These demonstrations highlight MoBa as a broadly valuable resource for advancing understanding of health and functioning across the lifecourse and generations.

## Abstract (web summary)

The Norwegian Mother, Father and Child Cohort Study demonstrates how family-based genomic data can disentangle direct genetic effects from confounding, improving studies of health and human development.

## Main

The field of genome-wide association studies (GWAS) has continued to advance through ever-increasing sample sizes and imputation with improved reference panels^12–23^. This has allowed the identification of thousands of single-nucleotide polymorphisms (SNPs) associated with a multitude of human traits^1^. Advancements in statistical methodologies and modelling software have allowed increasingly complex research questions to be addressed, for example, using multivariate GWAS^24^, polygenic score analysis^25–27^ and Mendelian randomization^28,29^.

Most of the GWAS so far have used clinical or population-based samples of unrelated individuals^3,30^. However, increasing evidence suggests that genetic associations estimated from unrelated individuals reflect not only direct genetic effects—in which an individual’s genetic variants causally influence their own phenotype—but also indirect effects of other individuals’ genotypes, such as parental genotypes shaping the rearing environment and thereby influencing offspring outcomes^31,32^. Estimates of indirect genetic effects in parent–offspring models reflect the association between parental genotypes and offspring outcomes after conditioning on the offspring’s genotype. These estimates capture ‘genetic nurture’ effects, as well as components arising from broader population structure, such as assortative mating and population stratification. Disentangling direct from indirect genetic effect estimates is very challenging in samples of unrelated individuals^3,33^. By contrast, family-based analyses can directly control for parental genotypes and exploit the random inheritance of genetic variation from parents to offspring to preclude many forms of environmental confounding. Therefore, using large samples of genotyped, related individuals^4–11^ is one of the most compelling ways to estimate the contributions of direct and indirect effects of both genotypes and phenotypes.

Genome-wide significant associations between SNPs and educational attainment in population-based GWAS of unrelated individuals are particularly attenuated in family-based studies^34^. A within-family GWAS of 179,086 siblings also suggested substantial attenuation of population-based estimates within families for height, age at first birth, number of children, cognitive ability, depressive symptoms and smoking^2^. Other studies of parent–offspring duos and trios have explicitly demonstrated that the parents’ non-transmitted genetic variants related to educational attainment associated with their offspring’s outcomes^31,35^. These findings provide strong evidence that within-family analyses can meaningfully disentangle direct and indirect genetic effects; a task that is beyond the reach of standard population-based approaches. Methods using familial relatedness in genotyped samples to estimate population parameters hold marked potential to advance our understanding of familial aggregation of health conditions and disabilities, co-occurrence between different health conditions and identification of causal risk and protective factors^36–42^.

Despite the valuable insights possible from studying genotyped families, few cohorts or biobanks have implemented large-scale family-based sampling^4–11^. Here, we emphasize the important role of genotyped family cohorts in advancing genomic research on health and functioning in the years ahead, highlighting the Norwegian Mother, Father and Child Cohort Study (MoBa) as a prime resource. MoBa combines extensive family-based genetic data with rich prospective and longitudinal follow-up of more than 100,000 children from prenatal life into adulthood^43^.

## MoBa, a family-based genetic resource

**Fig. 1:**
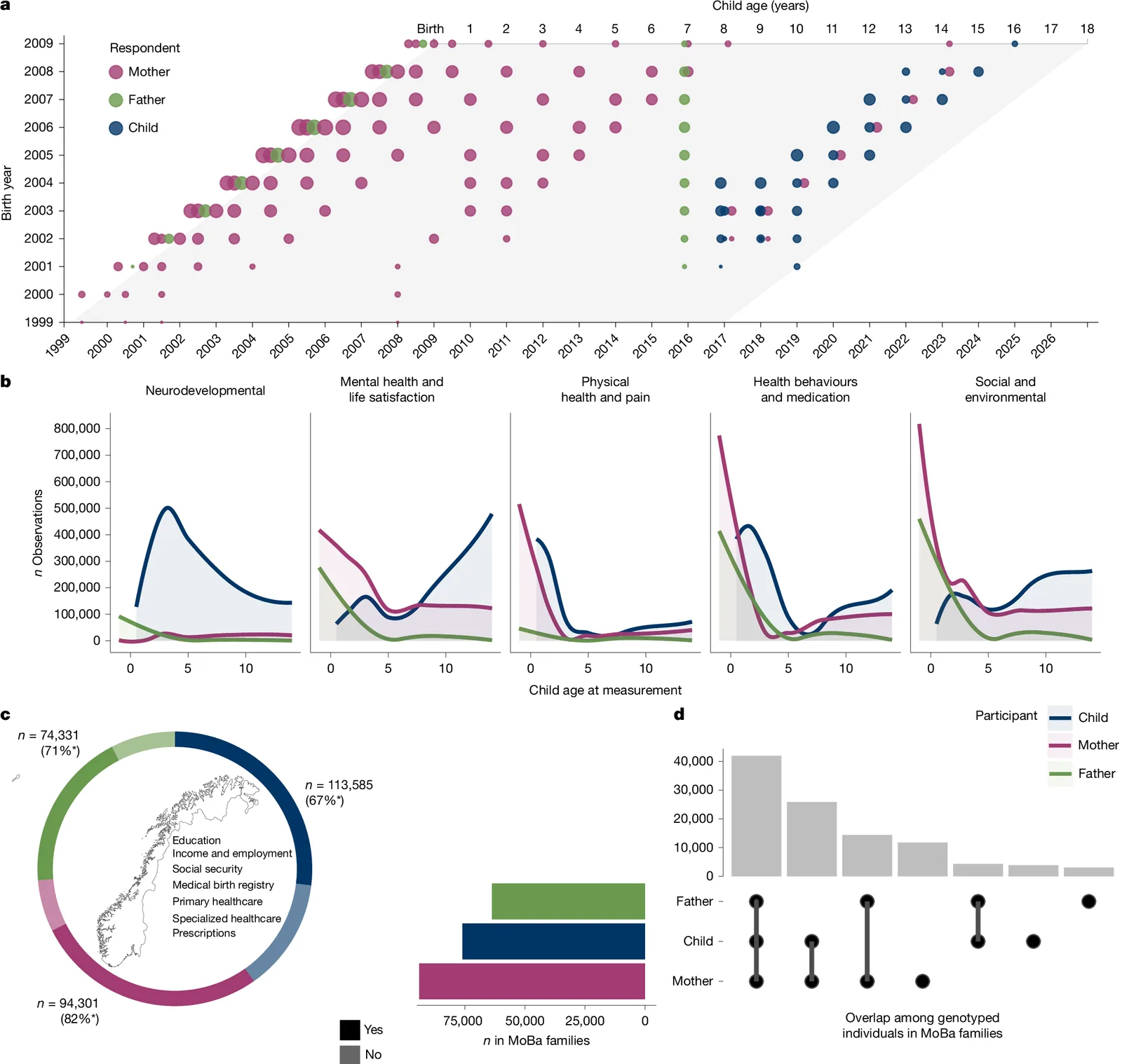
Overview of phenotypic and genetic data availability among MoBa participants across the first 16 years of data collection. **a**, Core MoBa questionnaires by respondent and child year during the first 16 years, points scaled in size relative to proportion of the full baseline sample of participating MoBa children with data available at each data collection wave. **b**, Summary of information obtained in different domains across core questionnaires administered in the first 14 years of core data collections; ‘*n* observations’ is calculated as the product of the number of responses by each participant type at each wave and the number of distinct measurement items in each domain at the same wave. **c**, Total *n* of mothers, fathers and children available in MoBa. Asterisk indicates the percentage of individuals with available genotype data passing quality control and with genetic similarity to the 1000 Genomes Project European superpopulation. The list is indicative of key linkable nationwide registries. **d**, Overlap among genotyped individuals within families in **c**—this is calculated at the level of individual MoBa children, meaning that genotyped mothers and fathers participating with more than one child are included more than once.

MoBa represents a uniquely powerful resource for family-based genetic analyses, offering a wide range of phenotypic measures on mothers, fathers and offspring, collected from early pregnancy and spanning more than 25 years. Within the main data collections covering the first 16 years of life alone (Fig. 1a), MoBa has amassed observations on neurodevelopment, mental health, physical health, health behaviours and medication use, social and environmental exposures (Fig. 1b), and broader aspects of functioning such as sleep, school achievement and social participation.

Ongoing waves of data collection follow the children through adolescence and into early adulthood. The rich prospective and longitudinal observations are complemented with linkage to Norwegian registry data (Fig. 1c). Norway has an extensive, high-quality health data infrastructure, including national health and administrative registries with continuous information on contacts, diagnoses, functioning assessments, treatments, care and support from the primary health and care services, the specialized health care services, and educational and social services. MoBa also includes an extensive range of environmental data collected through repeated questionnaires and registry linkages, as well as diverse biological samples (including plasma, urine and primary teeth), enabling integrated studies of genetic, environmental and biological mechanisms across the lifecourse. Furthermore, numerous nested clinic studies in MoBa have collected additional diverse biological samples and deep phenotype data, further expanding the potential to derive insights into human health and functioning using family-based genetic analyses^43^.

This potential^3^ is underpinned by the widespread availability of genotype data among MoBa parents and children. A considerable amount of relatedness between participating families in the cohort, combined with complexities in genotyping, necessitated the implementation of a quality control pipeline tailored to MoBa. Therefore, we developed and applied a family-based quality control and imputation pipeline that verifies and accounts for diverse types of relatedness in large population-based samples of individuals while appropriately handling differences arising from a varied genotyping strategy (Supplementary Note 1). The dataset passing post-imputation quality control included 77,634 mothers, 53,358 fathers and 76,577 children with the following relationships identified (Supplementary Note 2): 287 monozygotic twin pairs, 22,884 full-siblings, 117,006 parent–offspring pairs, 23,299 second-degree relative pairs, and 10,828 third-degree relative pairs. This included 44,017 full father–mother–child trios (Fig. 1d), which form a basis for refined genomic discovery and enhanced downstream genetic analyses.

Here, we demonstrate the valuable insights that can be gained by incorporating maternal and paternal genotypes in the investigation of genetic effects on offspring health and functioning. We apply a range of trio-based methods to four example offspring phenotypes spanning different domains of health and functioning, all measured in middle childhood. Children’s height (age 7 years), sleep duration (age 7 years) and depressive symptoms (age 8 years) were reported by mothers in MoBa questionnaires, whereas educational achievement was assessed using a composite score of numeracy, reading and English skills derived from the mandatory national tests of the educational registry in grade 5 (age 10 years). Descriptive information for these measures is presented in Table 1. To provide additional context for interpreting the offspring genetic and phenotypic results, descriptive statistics and Pearson correlations for similar outcomes to the four offspring phenotypes are presented for mothers and fathers in Extended Data Table 1. Spousal Pearson correlations are presented in Extended Data Table 2.

**Table 1:** Descriptive statistics and Pearson correlation matrix (with 95% CI) for the four middle childhood phenotypes

Phenotype	*n*^e^	Mean (s.d.^f^)	Range	Height	Educational achievement	Sleep duration	Depression symptoms
Height^a^	39,594	126.74 (5.77)	106–149	1.00			
Educational achievement^b^	72,774	59.44 (17.99)	0–100	0.039 (0.029, 0.049)	1.00		
Sleep duration^c^	40,612	10.12 (0.72)	8–12	−0.019 (−0.029, −0.010)	−0.008 (−0.017, 0.002)	1.00	
Depression symptoms^d^	31,359	1.83 (2.42)	0–25^g^	0.014 (0.003, 0.025)	−0.090 (−0.101, −0.078)	−0.066 (−0.077, −0.055)	1.00

Note: All four phenotype scores were standardized for use in the trio analyses to place them on a common scale and to facilitate interpretation and comparison of effect sizes. ^a^Height, in centimetres, was maternally reported at age 7 years. ^b^Educational achievement was assessed as a composite of national test scores in numeracy, reading and English at age 10 years. Scaled scores (0–100) are presented because the raw score ranges differed across test years; rescaling provides a consistent, comparable metric across test years.^c^Sleep duration, in hours, was maternally reported at age 7 years. ^d^Depression symptoms were maternally reported at age 8 years using the Short Mood and Feelings Questionnaire (SMFQ). ^e^*n* genotyped children. ^f^Standard deviation. ^g^The full possible range of the SMFQ is 0–26; however, the range in the genotyped children was 0–25.

## Refined genomic discovery with trio-GWAS

The availability of rich phenotypic and genotypic data from family cohorts such as MoBa opens new opportunities to identify specific common genetic variants causing variability in health and functioning. GWAS using parent–offspring data are particularly useful for disentangling the different sources of genetic association at the parent and offspring levels^2^. We performed population-based and trio-GWAS on the four offspring traits and compared SNP–phenotype associations between population-based and family-based analyses.

**Fig. 2:**
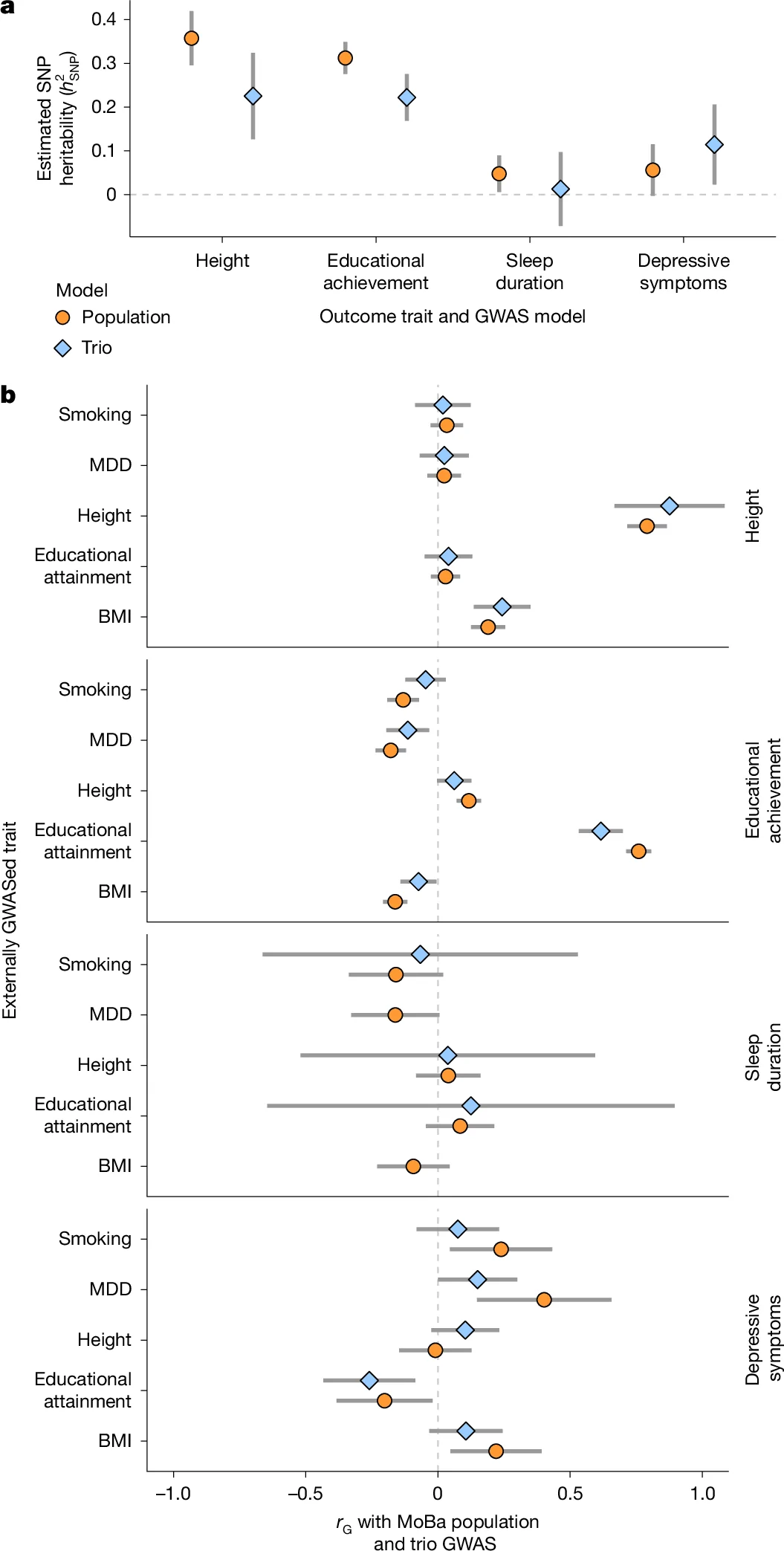
GWAS conducted within-family trios refine genomic discovery and reduce bias. **a**, SNP heritability estimates calculated using LDSC based on summary statistics from population- and trio-based GWAS models of children’s height, educational achievement, sleep duration and depressive symptoms. Data are presented as LDSC model estimates with 95% CIs. **b**, Estimates of genetic correlations (*r*_G_s) between children’s height, educational achievement, sleep duration and depressive symptoms and five externally GWASed traits were calculated using LDSC based on either population- or trio-based GWAS models conducted in MoBa (note that sleep duration (trio model) – major depressive disorder (MDD)/BMI *r*_G_s could not be stably estimated). Data are presented as LDSC model estimates with 95% CIs. *n* trios by outcome: educational achievement, 41,761; height, 23,786; sleep duration, 24,376; and depressive symptoms, 18,701.

In the population models, SNP-based heritability (${h}_{\mathrm{SNP}}^{2}$) for height and educational achievement was ${h}_{\mathrm{SNP}}^{2}$ = 35% (standard error (s.e.) = 3%) and ${h}_{\mathrm{SNP}}^{2}$ = 31% (s.e. = 2%), respectively. Estimates were lower for sleep duration (${h}_{\mathrm{SNP}}^{2}$ = 5%, s.e. = 2%) and depressive symptoms (${h}_{\mathrm{SNP}}^{2}$ = 6%, s.e. = 3%). In the trio models, which conditioned on parental genotypes, we saw the heritability estimates for height and education attenuate to ${h}_{\mathrm{SNP}}^{2}$ = 22% (5%) and ${h}_{\mathrm{SNP}}^{2}$ = 22% (3%), respectively (Fig. 2a). Heritability for sleep duration was reduced to near zero within-families (${h}_{\mathrm{SNP}}^{2}$ = 1%, s.e. = 4%), whereas the within-family estimate for depressive symptoms was higher (${h}_{\mathrm{SNP}}^{2}$ = 11%, s.e. = 5%).

Figure 2b presents the linkage disequilibrium score regression (LDSC) estimates of genetic correlations (*r*_G_s) between each MoBa GWAS (height, educational achievement, sleep duration and depressive symptoms) and large external population-based GWAS of height^44^, body mass index (BMI)^45^, educational attainment^46^, smoking initiation^47^ and depression^48^. For MoBa children’s educational achievement, the within-family estimated genetic correlations were attenuated for all phenotypes. The genetic correlation with educational attainment was lower in the family-based (*r*_G_ = 0.62, s.e. = 0.04, *P* = 1.14 × 10^−47^) compared with the population-based analyses (*r*_G_ = 0.76, s.e. = 0.02, *P* = 3.06 × 10^−216^). Similarly, its genetic correlations with BMI and smoking initiation were halved in the family-based (BMI *r*_G_ = −0.07 (s.e. = 0.03, *P* = 0.03) and smoking *r*_G_ = −0.05 (s.e. = 0.04, *P* = 0.23)) versus in the population-based analyses (*r*_G_ = −0.16 (s.e. = 0.02, *P* = 7.82 × 10^−12^) and *r*_G_ = −0.13 (s.e. = 0.03, *P* = 1.95 × 10^−5^), respectively). Similar attenuation was observed for the genetic correlation between MoBa educational achievement and height (*r*_G_ = 0.06 (s.e. = 0.03, *P* = 0.06) in family-based and *r*_G_ = 0.12 (s.e. = 0.02, *P* = 8.96 × 10^−7^) in population-based analyses).

For MoBa children’s height, changes in *r*_G_ estimates were less substantial, but notably, *r*_G_ estimates with external GWAS of height and BMI (*r*_G_ = 0.88 (s.e. = 0.11, *P* = 1.81 × 10^−16^) and *r*_G_ = 0.24 (s.e. = 0.05, *P* = 9.06 × 10^−6^), respectively) increased when adjusting for parental genotypes.

For depressive symptoms in MoBa children, the genetic correlation with depression was attenuated in the family-based (*r*_G_ = 0.15 (s.e. = 0.08, *P* = 0.05)) compared with the population-based analyses (*r*_G_ = 0.40 (s.e. = 0.13, *P* = 0.002)). The genetic correlations with BMI and smoking initiation were also attenuated within-family (*r*_G_ = 0.11 (s.e. = 0.07, *P* = 0.13) and *r*_G_ = 0.08 (s.e. = 0.08, *P* = 0.34)) compared with the population-based analyses (*r*_G_ = 0.22 (s.e. = 0.09, *P* = 0.01) and *r*_G_ = 0.24 (s.e. = 0.10, *P* = 0.02), respectively), whereas the genetic correlation with educational attainment was marginally higher within-family *r*_G_ = −0.26 (s.e. = 0.09, *P* = 0.004) compared with the population-based analyses *r*_G_ = −0.20 (s.e. = 0.09, *P* = 0.03).

For MoBa sleep duration, no genetic correlations with external GWAS traits were statistically significant in either the population-based or trio-based analyses (all *P* > 0.05). Estimates were modest and accompanied by large standard errors, particularly in the within-family model.

The estimates of associations of genome-wide significant variants previously reported to be associated with height and education were attenuated by 11.0% (95% confidence interval (CI) 8–15%) and 23.0% (95% CI 18–28%) after controlling for parental genotype, respectively. We also observed within-family attenuation for genetic variants associated with sleep duration and depressive symptoms, with association estimates decreasing by 29% (95% CI 9–49%) and 16% (95% CI 3–28%) from population to within-family analyses, respectively.

**Extended Data Fig. 1:**
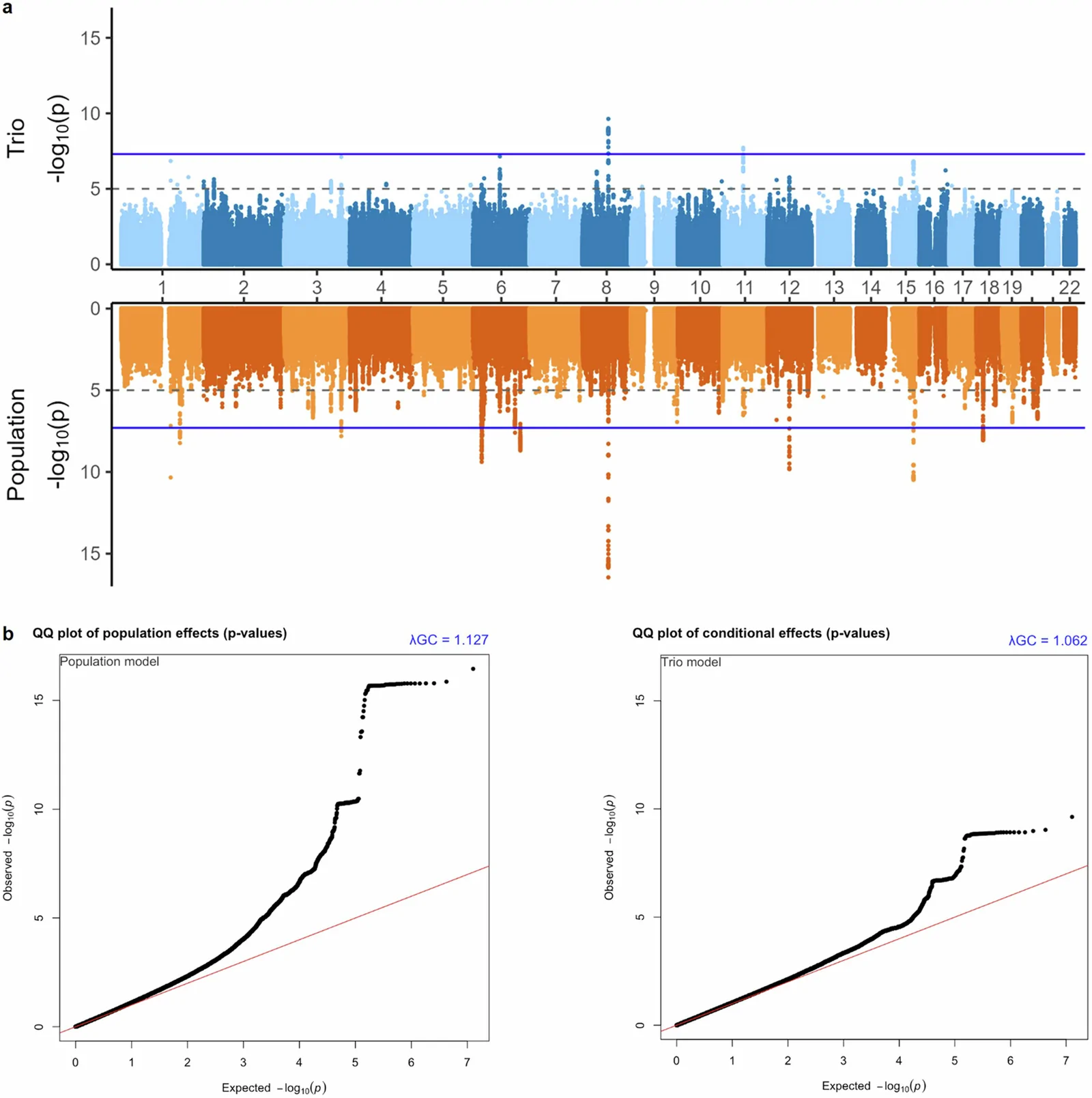
Trio- and population genome-wide association studies (GWAS) of MoBa children’s height (n = 23,786 trios). Panel **a**, Miami plot showing -log10 p-values for associations between individual genetic variants and MoBa children’s height in trio- (blue) and population-based (orange) GWAS models. The blue line is the genome-wide significance threshold (p-value < 5 × 10^−8^) for common variants in samples with genetically inferred European ancestry, while the dashed grey line is the suggestive threshold (p-value < 1 × 10^−5^); **b**. Quantile-quantile (QQ) plots showing the distribution of p-values from the population-based (left) and conditional (right) GWAS models. The red diagonal line illustrates the expected distribution of p-values under the null hypothesis of no association.

**Extended Data Fig. 2:**
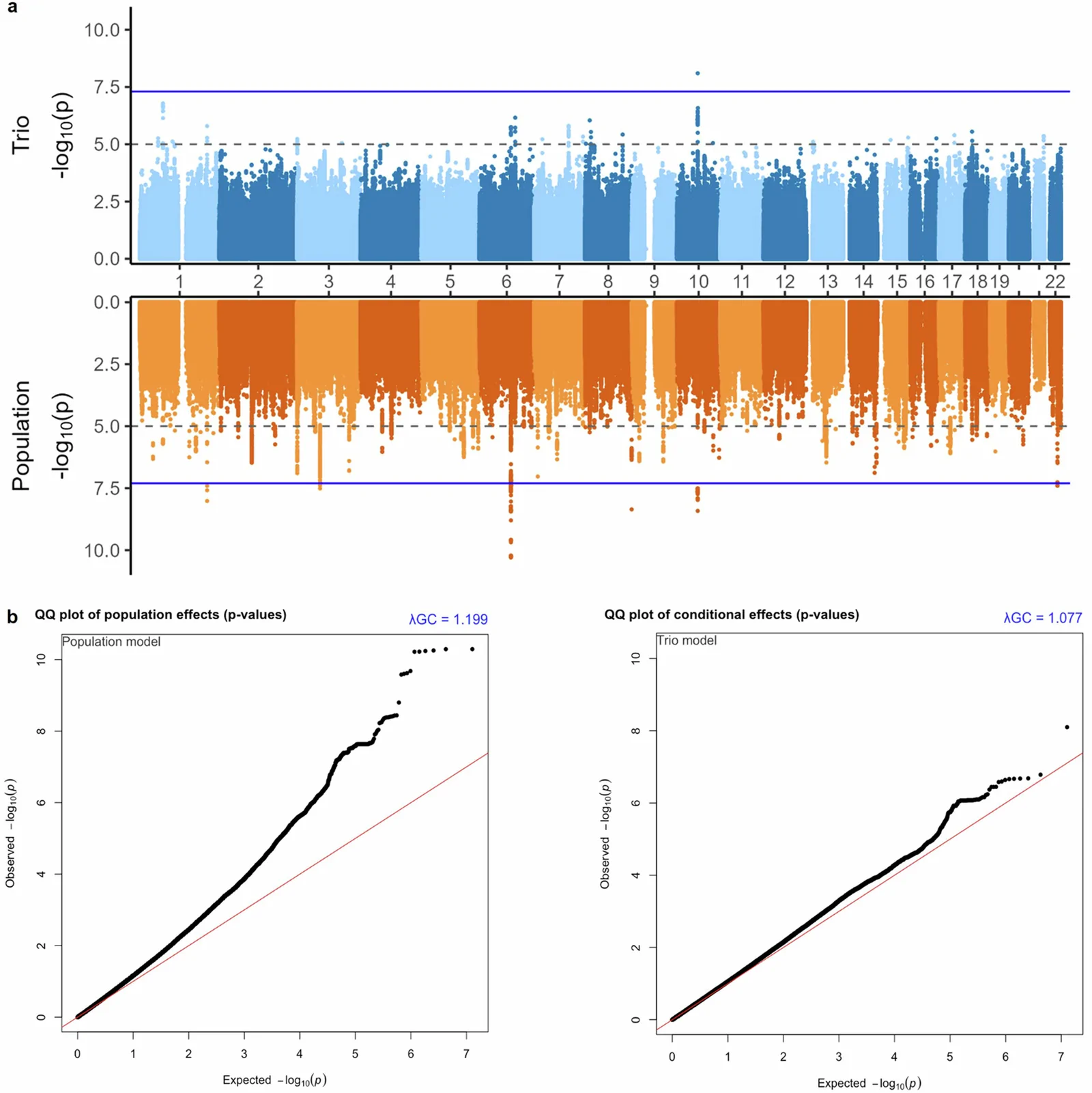
Trio- and population genome-wide association studies (GWAS) of MoBa children’s educational achievement (n = 41,761 trios). Panel **a**, Miami plot showing -log10 p-values for associations between individual genetic variants and MoBa children’s educational achievement in trio- (blue) and population-based (orange) GWAS models. The blue line is the genome-wide significance threshold (p-value < 5 × 10^−8^) for common variants in samples with genetically inferred European ancestry, while the dashed grey line is the suggestive threshold (p-value < 1 × 10^−5^); **b**. Quantile-quantile (QQ) plots showing the distribution of p-values from the population-based (left) and conditional (right) GWAS models for educational achievement. The red diagonal line illustrates the expected distribution of p-values under the null hypothesis of no association.

**Extended Data Fig. 3:**
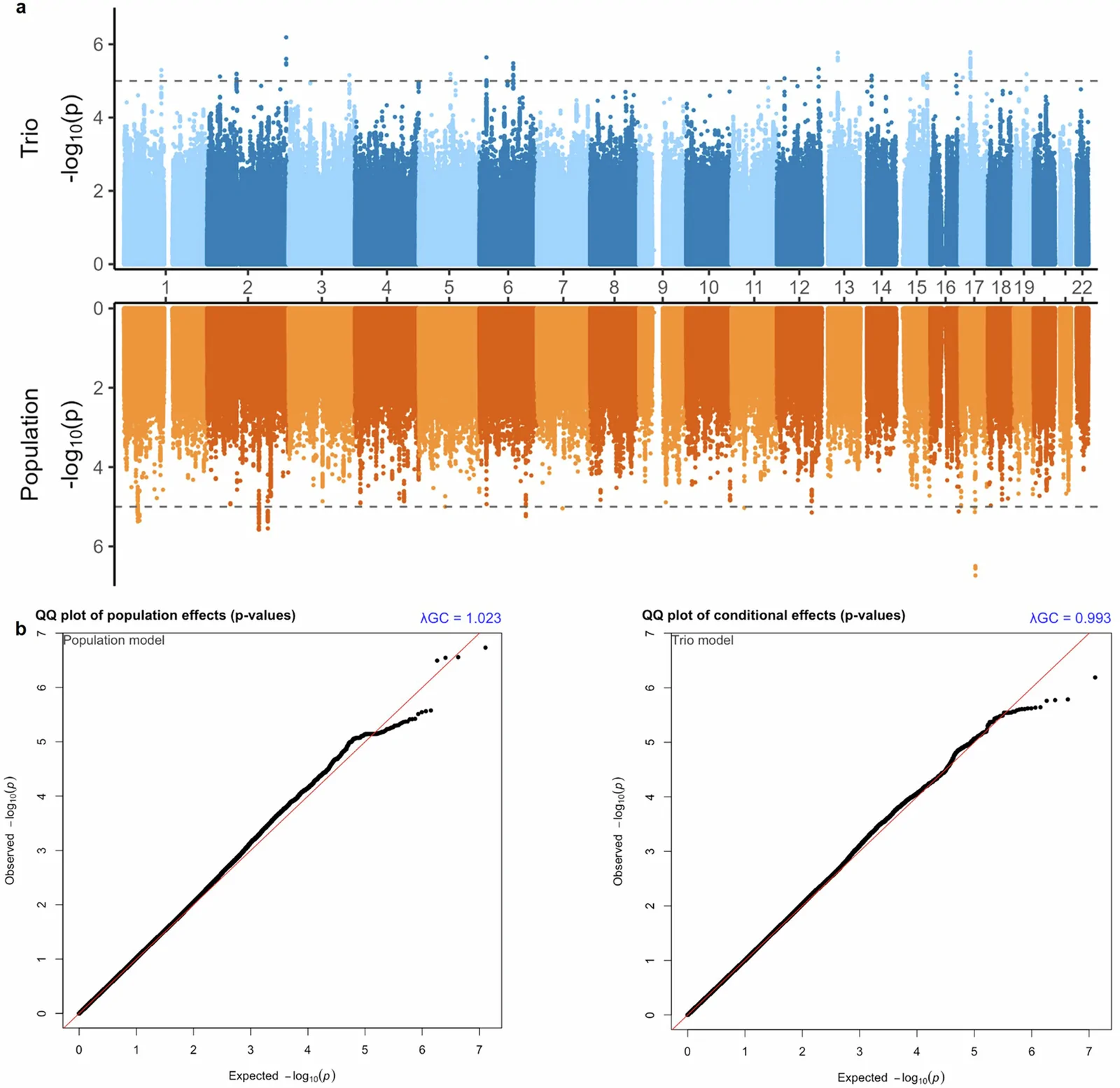
Trio- and population genome-wide association studies (GWAS) of MoBa children’s sleep duration (n = 24,376 trios). Panel **a**, Miami plot showing -log10 p-values for associations between individual genetic variants and MoBa children’s sleep duration in trio- (blue) and population-based (orange) GWAS models. The blue line is the genome-wide significance threshold (p-value < 5 × 10^−8^) for common variants in samples with genetically inferred European ancestry, while the dashed grey line is the suggestive threshold (p-value < 1 × 10^−5^); **b**. Quantile-quantile (QQ) plots showing the distribution of p-values from the population-based (left) and conditional (right) GWAS models for sleep duration. The red diagonal line illustrates the expected distribution of p-values under the null hypothesis of no association.

**Extended Data Fig. 4:**
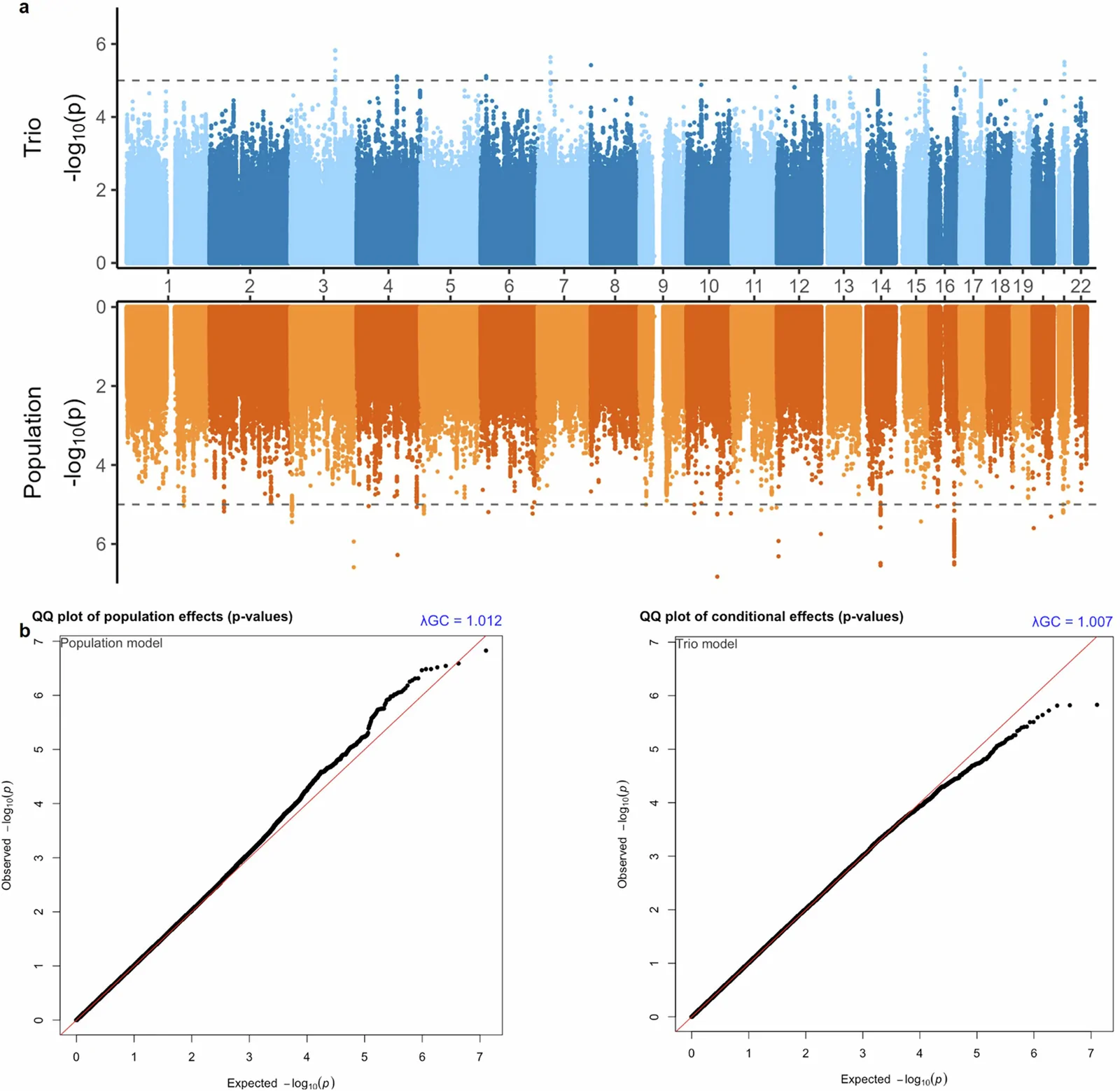
Trio- and population genome-wide association studies (GWAS) of MoBa children’s depression symptoms (n = 18,701 trios). Panel **a**, Miami plot showing -log10 p-values for associations between individual genetic variants and MoBa children’s depressive symptoms in trio- (blue) and population-based (orange) GWAS models. The blue line is the genome-wide significance threshold (p-value < 5 × 10^−8^) for common variants in samples with genetically inferred European ancestry, while the dashed grey line is the suggestive threshold (p-value < 1 × 10^−5^); **b**. Quantile-quantile (QQ) plots showing the distribution of p-values from the population-based (left) and conditional (right) GWAS models for depression symptoms. The red diagonal line illustrates the expected distribution of p-values under the null hypothesis of no association.

Our population-based GWAS identified 98 and 26 genome-wide significant (*P* < 5 × 10^−8^) offspring variants associated with height and educational achievement, respectively. Of these, five variants for height and one for educational achievement remained associated at the genome-wide significance level after adjusting for parental genotype (see Miami plot Extended Data Fig. 1a for height and Extended Data Fig. 2a for educational achievement, Extended Data Fig. 3a for sleep duration and Extended Data Fig. 4a for depressive symptoms). The within-family standard errors were on average 38% larger than the population-based standard errors across traits (height 38%, educational achievement 37%, sleep duration 38% and depression 39%), indicating lower statistical power in the within-family analyses. Quantile–quantile plots indicate significant deviation from the expected distribution in the population-based models for each phenotype (see Extended Data Fig. 1b for height, Extended Data Fig. 2b for educational achievement, Extended Data Fig. 3b for sleep duration and Extended Data Fig. 4b for depressive symptoms). This is substantially reduced in the conditional models that control for parental genotype.

Taken together, these results show that a substantial proportion of SNP-based heritability, and many reported SNP-trait and cross-trait associations in population-based GWAS, especially for educational achievement and depressive symptoms, reflect indirect effects and between-family structure rather than direct genetic effects on offspring traits. Trio-GWAS in MoBa reveals smaller but more likely causal within-family genetic effects, altered patterns of genetic overlap with other traits and fewer genome-wide significant loci, underscoring both the power and necessity of family-based designs for refining genomic discovery.

## Trio-polygenic score methods

Apart from facilitating refined genomic discovery, genetic data in family cohorts allow downstream genetic analyses to be performed in new ways. We used two family-based approaches that use polygenic scores (PGS): trio-PGS and polygenic disequilibrium transmission testing. These complementary approaches were used in MoBa to analyse within-family transmission mechanisms for height, educational achievement, sleep duration and depressive symptoms.

**Fig. 3:**
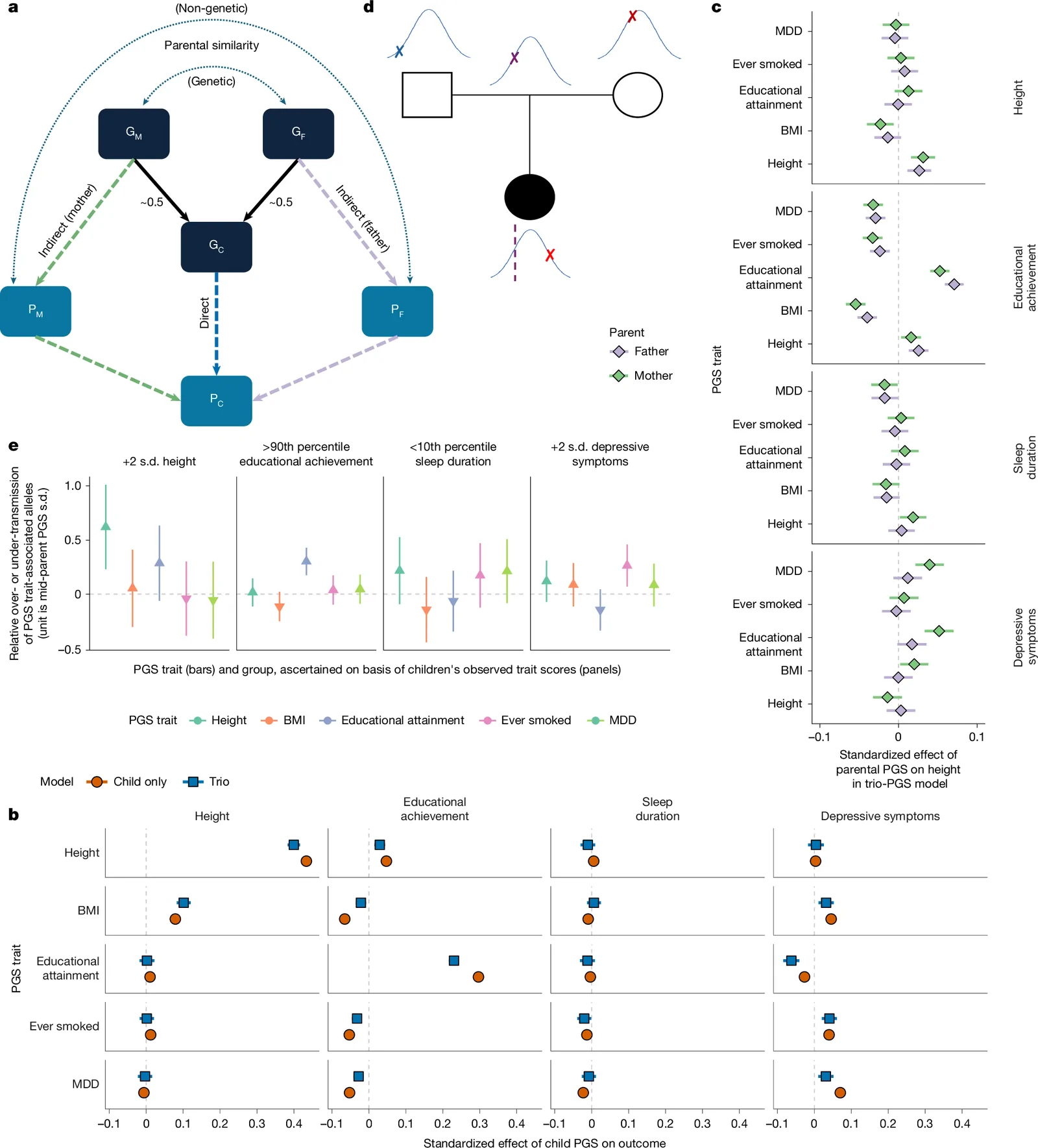
Within-family PGS-based analyses provide estimates of direct genetic effects adjusted for systematic structural confounding. **a**, Schematic of links between mothers’, fathers’ and children’s genetic liabilities (G_M__/F/C_) and phenotypes (P_M/F/C_). The solid lines indicate known relations and the dotted lines indicate unknown, but plausible relations. Trio-PGS analyses involve estimation of the direct path between G_C_ and P_C_, with control for confounding by G_M/F_ and P_M/F_. **b**, Estimates of direct genetic effects on MoBa children’s height, educational achievement, sleep duration and depressive symptoms from child-only and trio-PGS regression models. Data are presented as standardized regression model coefficients with 95% CIs. **c**, Effects of parental PGS that explain differences between direct effect estimates in the two models in **b**. Data are presented as standardized regression model coefficients with 95% CIs. **d**, Schematic of the expectation of polygenic transmission disequilibrium, in which genetic liabilities are systematically over- or under-transmitted when families are selected based on a child trait with a direct link to that genetic liability. **e**, Polygenic transmission disequilibrium (PTD), indexed by systematic differences in polygenic scores—as compared with a calculated mid-parental average polygenic score—among children selected for, respectively, height 2 standard deviations (s.d.) above the mean, educational achievement above 90th percentile, sleep duration below 10th percentile, and depressive symptoms 2 s.d. above the mean. Over- or under-transmission relative to mid-parental average polygenic score is usually interpreted as evidence of genetic influence on child trait free from structural confounding such as population stratification or assortative mating. Data are presented as means with 95% CIs. *n* trios by outcome in PGS analyses (**b**,**c**): educational achievement, 40,101; height, 21,008; sleep duration, 21,451; and depressive symptoms, 18,212. *n* trios by outcome in PTD analyses (**e**): educational achievement, 3,227; height, 527; sleep duration, 438; and depressive symptoms, 618.

**Extended Data Fig. 5:**
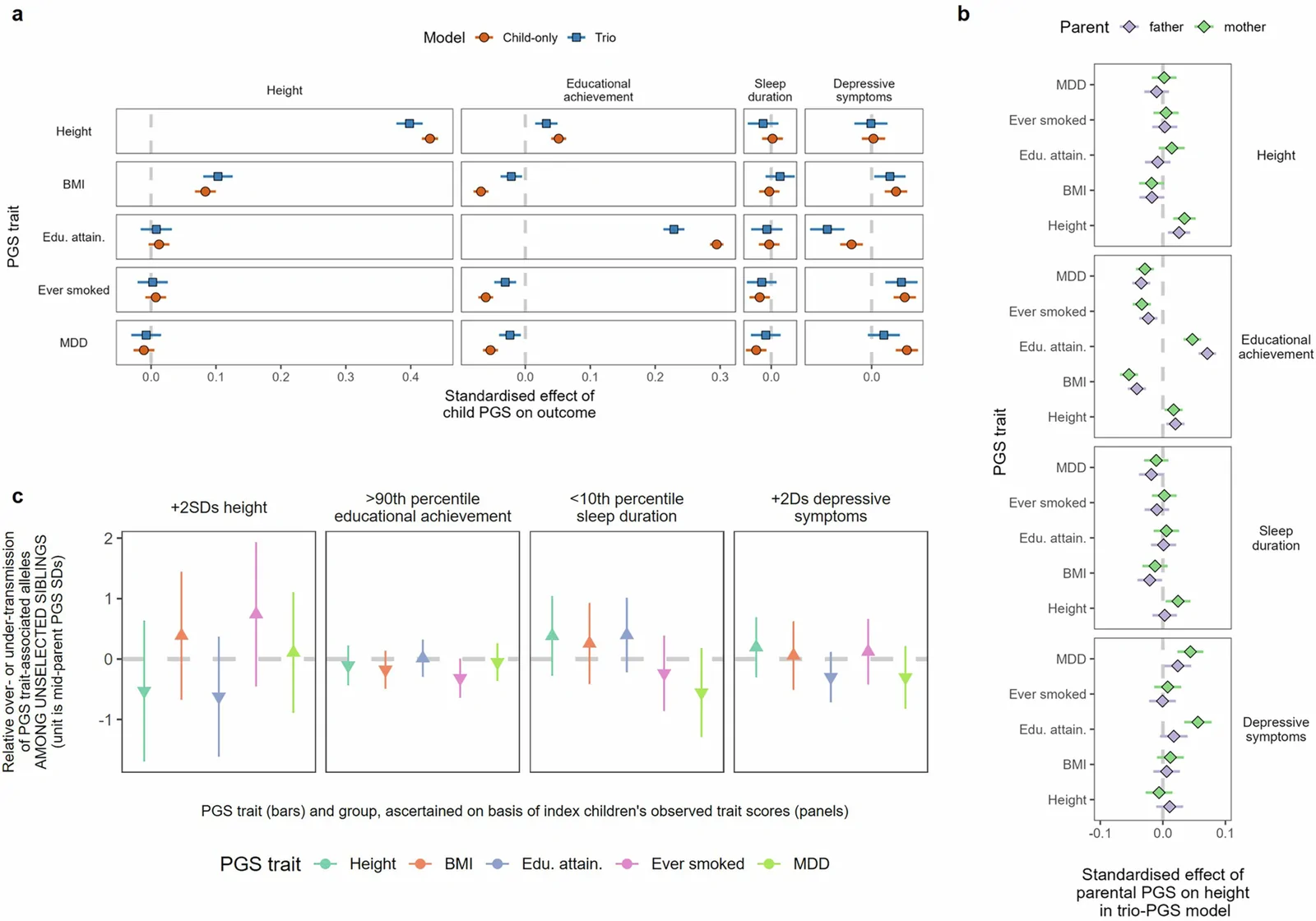
Within-family polygenic score (PGS) based sensitivity analyses. Panel **a**, Estimates of direct genetic effects on MoBa children’s height, educational achievement, sleep duration, and depressive symptoms from child only- and trio- polygenic score (PGS) models in unrelated trios. The error bars are 95% confidence intervals; **b**. Effects of parental polygenic scores (PGS) on MoBa children’s height, educational achievement, sleep duration, and depressive symptoms in trio-PGS models in unrelated trios. The error bars are 95% confidence intervals; **c**. Results of polygenic transmission disequilibrium (PTD) analysis, among the siblings of children ascertained on the basis of extreme trait scores. Data are presented as means with 95% confidence intervals. N trios by outcome in PGS analyses (panels a & b): educational achievement: 27,926; height: 14,541, sleep duration: 15,026; depressive symptoms: 12,632. N trios by outcome in PTD analyses (panel c): educational achievement: 535; height: 84, sleep duration: 78; depressive symptoms: 95.

The trio-PGS model allows the respective contributions of children’s own and each of their parents’ PGS for specific traits to explain variance in an outcome simultaneously^38^ (Fig. 3a). Using trio-PGS, we showed that the unadjusted association (*β* = 0.43, 95% CI 0.43–0.44) between a child’s PGS for height and their measured height is inflated by 9% because of the influence of parents’ PGS (Fig. 3b, left-most). Both paternal (*β* = 0.03, 95% CI 0.01–0.04) and maternal (*β* = 0.03, 95% CI 0.02–0.05) PGS independently contributed to this inflation (Fig. 3c, top). Similar evidence of parental PGS biasing estimates of children’s PGS associations with their own traits was evident across all outcomes. In most cases, unadjusted estimates were inflated, leading to seemingly stronger associations when parents’ PGS were not controlled for. However, there were notable examples of parents’ PGS suppressing associations between children’s PGS and their traits. For instance, the association between children’s PGS and educational attainment and their depressive symptoms more than doubled after adjustment for parents’ PGS (*β*_unadjusted_ = −0.03, 95% CI −0.04 to −0.02; *β*_adjusted_ = −0.06, 95% CI −0.08 to −0.04; Fig. 3b, right-most). Figure 3c (bottom) illustrates that this suppression effect, as well as the 130% inflation of the association between child PGS to depression and their own depressive symptoms in unadjusted models, were attributable to maternal, but not paternal, genetic effects. Sensitivity analyses confirmed that the inclusion of individuals related across trios was not responsible for driving the pattern of results (see Extended Data Fig. 5a for estimates of direct genetic effects and Extended Data Fig. 5b for effects of parental PGS).

Polygenic transmission disequilibrium testing (PTDT; Fig. 3d) examines whether children selected for a given phenotype, for example height, receive a higher (or lower) polygenic load for this phenotype from their parents^39^. Under Mendelian inheritance, a child’s PGS should, on average, be equal to the mid-parent PGS. If the phenotype is influenced by genetic variants in the PGS, then children selected for extreme values on that phenotype should show over-transmission of the relevant PGS (on average, the children should have higher PGS than expected based on the average of their parents’ PGS). If, instead, the PGS–phenotype association is due to structural confounding (for example, from population stratification) that also influences parental PGS, this pattern of over-transmission would be absent or attenuated. Importantly, PGS of unselected siblings should not exceed the mid-parent average—regardless of the relationship between the PGS and the phenotype of selection.

For these analyses (Fig. 3e), we used subsets of MoBa children based on extreme height (>2 s.d., above the sample mean; *n* = 527, plus *n* = 84 unselected sibling controls), educational achievement above the 90th percentile, (*n* = 3,227, *n*_siblings_ = 535), sleep duration below the 10th percentile (*n* = 438, *n*_siblings_ = 78), and extreme depressive symptoms (>2s.d. above the sample mean; *n* = 618, *n*_siblings_ = 95). Using PTDT, we observed significant over-transmission of PGS for height among extremely tall children (*P*_FDR_ < 0.001) and educational attainment among children with high educational achievement (*P*_FDR_ < 0.03). In both cases, siblings did not show evidence of similar over-transmission (Extended Data Fig. 5c), although estimates were considerably less precise. Neither sleep duration nor depressive symptoms PTDT analyses provided robust evidence for over- or under-transmission of any of the PGS. These findings suggest that, for height and educational achievement, the PGS signal reflects genuine within-family genetic transmission rather than solely between-family confounding, strengthening causal interpretation of these polygenic influences. By contrast, the lack of clear over-transmission for sleep duration and depressive symptoms suggests that current PGS for these traits either capture weaker within-family genetic effects or are more strongly influenced by confounding and measurement error, highlighting areas in which larger discovery samples and improved phenotyping are needed.

Spousal PGS Pearson correlations indicated modest within-trait assortative mating for the PGS included in our analyses, most pronounced for educational attainment (*r* = 0.14) and weaker for height, BMI, smoking and depressive symptoms (*r* = 0.02–0.06) (Extended Data Table 3).

Together, the results of the trio-PGS and PTDT analyses show that modelling parental genotypes, even in analyses using scores based on population-based GWAS, can reduce bias in the estimation of direct genetic effects. The results also show that this bias can influence PGS–phenotype associations by maternal and paternal genotypes independently, highlighting the importance of trio data for solving this problem.

## Trio-genome-wide complex trait analysis

Variance decomposition methods such as genome-wide complex trait analysis (GCTA)^49^ use observed genome-wide genetic relatedness to estimate heritability. Similar to genomic discovery and polygenic score-based methods, their estimates can be refined and improved in the context of genotyped family data. Trio-GCTA^36^ uses parent–offspring trios to estimate the genome-wide contributions of direct and indirect genetic effects. We applied trio-GCTA to the exemplar child phenotypes—height, educational achievement, sleep duration and depressive symptoms—to disentangle the relative roles of genome-wide direct and indirect genetic effects beyond trait-specific PGS.

**Fig. 4:**
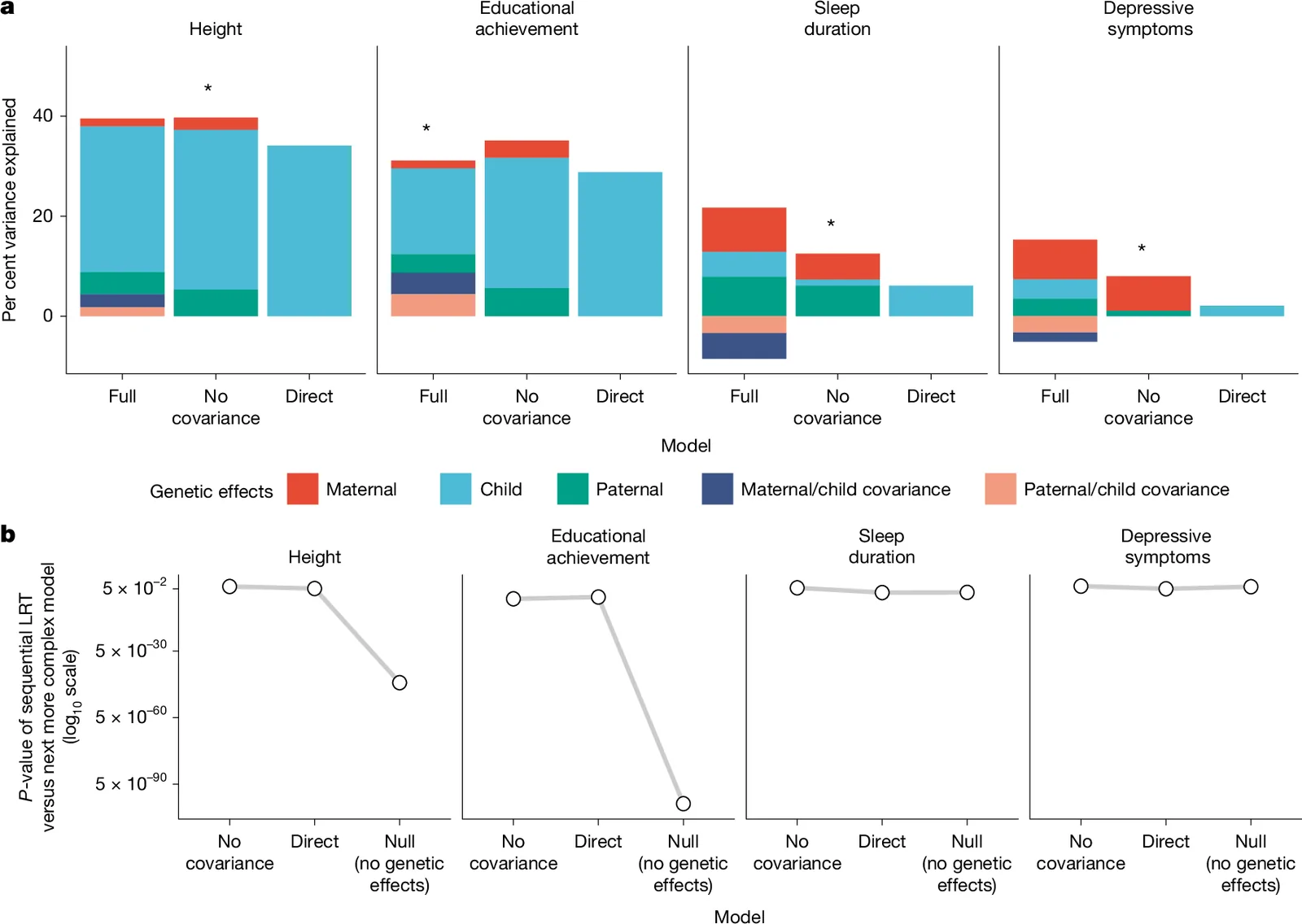
Trio-GCTA variance decomposition allows estimation of the total contribution of direct and both maternal and paternal indirect genetic effects. **a**, Estimated variance components from trio-GCTA of MoBa children’s height, educational achievement, sleep duration and depressive symptoms. The proportion of variance explained by child, parental genetic effects and positive covariance between parental and child genetic effects, which inflates the total variability in the phenotype explained by genetic effects, are shown in stacked bars above zero. Negative covariance between parental and child genetic effects is shown in stacked bars below zero as these effects will deflate the total variability in the phenotype explained by genetic effects. The best-fitting model as selected on the basis of likelihood ratio model fit comparison tests (likelihood ratio test, LRT) and Akaike information criterion values is indicated with an asterisk. Models with no genetic effects were also tested but are not shown. *P-*values for the LRT were calculated from a single chi-square distribution when covariance parameters were constrained (two-sided) and a mixture of chi-square distributions when variance parameters were constrained (one-sided); for all outcomes, a model including indirect effects were preferred—for educational achievement only, the covariance between effects was also included in the best-fitting model. **b**, LRT *P* values from trio-GCTA of height, educational achievement, sleep duration and depressive symptoms. For both height and educational achievement, the decision to reject the model without genetic effects was clear. All other decisions were marginal, indicating that genetic effects overall were minimal for sleep duration and depression and indirect effects played a limited part in explaining variation in all outcomes. *n* trios by outcome in GCTA analyses: educational achievement, 23,221; height, 12,332; sleep duration, 12,799; and depressive symptoms, 10,794.

Trio-GCTA provided clear evidence for genetic effects on height and educational achievement, and statistically detectable but smaller genetic contributions to sleep duration and depressive symptoms (Fig. 4a). For educational achievement, the best-fitting model included covariances between maternal, paternal and offspring genetic effects, whereas for the other phenotypes a simpler model without covariances sufficed. Likelihood ratio tests indicated that parental genetic variance components improve model fit for height (*P* = 0.03), sleep duration (*P* = 3.4 × 10^−4^), and depressive symptoms (*P* = 0.02), but the estimated indirect effects are modest and imprecise and the contrast between model fit statistics (Extended Data Table 4) suggests only weak evidence for indirect effects.

Direct effects were the largest contributor to genetic variance in educational achievement and height (educational achievement: 17.2% (s.e. = 2.5%); height 32.0% (s.e. = 2.8%) in the best-fitting model). Reflecting conclusions from comparison of model fit indices, depressive symptoms and sleep duration had comparatively little variance explained by genetic effects in general (Fig. 4b), with weak evidence for primarily indirect effects. Indirect effects were also found for educational achievement and, to a lesser extent, height. For these traits, in contrast to depressive symptoms and sleep duration, the indirect effects were primarily estimated to originate with fathers. For educational achievement, significant positive covariances between parental and child effects indicate gene-environment correlation acting to increase the overall variance explained by genetic effects in the phenotype.

The results indicate that when modelling genome-wide genetic effects in parent–offspring trios, children’s own genotypes (direct effects) can explain a substantial part of genetic variance for some traits (especially educational achievement and height), but not all of it. Ignoring parental genotypes can bias estimates of child genetic effects, particularly for educational achievement, for which family genetics may be especially intertwined.

Inclusion of parental genetic effects in downstream genetic analyses—whether based on specific genetic propensities indexed by PGS or based on decomposition of all genetic variance in the trait—gives new insights into genetic mechanisms. Neither approach conclusively proves why children’s traits associate with parental genotypes; so-called indirect ‘effects’ comprise many different sources of association^3,33^, but both can demonstrate that the associations between children’s own genotypes and phenotypes may be confounded. Moreover, both using specific genetic propensities (PGS) and genome-wide variation approaches can provide unbiased estimates of the direct effects of children’s own genotypes on their phenotypes.

## Conclusion

Our work demonstrates the vast potential of MoBa, a genotyped family cohort with rich phenotype data, for refining genomic discoveries, clarifying genetic overlaps and investigating intergenerational transmission. With large-scale genotyping of parents and offspring, deep longitudinal phenotyping and linkage to comprehensive health and administrative registries, MoBa provides a unique resource for family-based genetic analyses across health and functioning. Here, we have brought together and harmonized genotype data from multiple projects in MoBa and applied quality control procedures tailored to the unique characteristics of the sample.

Using these data, trio-GWAS embeds direct control for parental genotype into the genomic discovery process. This allows the most likely biologically mediated SNP–trait associations to be distinguished from those due to population stratification, genetic nurture and assortative mating. Modern population-based GWAS efforts, powered by ever-increasing sample sizes, now routinely detect hundreds of robust, independent SNP–outcome associations^18,44–46,48,50^. Our trio-GWAS results indicate that these associations cannot necessarily be attributed to the direct effects of genotype on phenotype. Moreover, we show not only inflation of heritability estimates in population-based compared with trio-GWAS but also substantial changes in the patterns of overlap with other traits. Polygenic-score-based and variance-decomposition-based approaches reveal these nuances, highlighting avenues for future investigation—such as parent-specific indirect effects, opposing effects of the same PGS in children and parents, and assortative mating. Family-based analyses and samples such as these represent a jumping-off point for the defining challenge of the post-GWAS era: explaining mechanistic links between common genetic variation and human traits. Estimates of direct genetic effects using trio data should be reasonably robust to all the sources of indirect effects, and therefore allow for prioritization of causal variants and effect sizes. However, estimates of indirect genetic effects may reflect varied components, including not only genetic nurture but also broader demographic factors, such as assortative mating, population stratification, dynastic effects and indirect genetic effects from relatives other than parents. Strategies to disentangle these factors involve triangulation across genetic designs—including those demonstrated here, and others such as mediation analyses^51,52^, extended pedigrees^53^ and assortative mating modelling^40^, as well as across multiple sources of family genetic data. Systematic investigation of assortative mating across cohorts, countries and historical periods is important for understanding how population-level mating patterns shape indirect genetic effects and their generalizability. Furthermore, as within-family GWAS incorporating samples such as MoBa become larger and more powerful, the potential to directly mitigate and control for confounding from assortative mating and population stratification in downstream within-family analyses will also increase.

Beyond its family-based structure, MoBa has many other distinctive features that can facilitate the development and application of new methods and innovative uses of genotype data. For example, MoBa features a prospective, longitudinal follow-up of more than 100,000 children from prenatal life to adulthood, allowing for comprehensive tracking and genetic analysis of health and functioning. Moreover, the 10-year recruitment period (Fig. 1a) presents opportunities to study temporal interplay between genetics and shifting environmental and societal contexts that affect health and functioning across generations. Nevertheless, no single resource or study design is without limitations. Lengthy recruitment periods and follow-up periods can present challenges as well as opportunities, particularly where measurement cannot be kept consistent either across time or age. Furthermore, the effects of non-random ascertainment and attrition represent crucial considerations for within-family analyses using such data. Finally, family-based cohorts such as MoBa are expensive to recruit and maintain, and care must be taken to ensure that prioritization of family-based cohorts in the coming years does not undermine ongoing efforts to diversify the ancestral populations included in genetic samples and ensure insights can benefit the broadest possible population. At the same time, because within-family designs are robust to population stratification, they are particularly well suited to addressing concerns about stratification in ancestrally diverse and samples with recent admixture, underscoring the importance of investing in family-based studies across a broad range of populations. Despite these limitations, a combination of widespread implementation of existing within-family approaches to new data sources and the integration of these approaches with longitudinal designs and natural experiments means that research in family-based cohorts is likely to play a central part in promoting ground-breaking discoveries in genomics in the coming years.

## Methods

### The Norwegian MoBa cohort

MoBa is a population-based pregnancy cohort study conducted by the Norwegian Institute of Public Health. Participants were recruited from all over Norway from 1999 to 2008. The women consented to participation in 41% of the pregnancies (*n* = 112,908 recruited pregnancies)^43,54^. The cohort includes approximately 114,500 children, 95,200 mothers and 75,200 fathers. The establishment of MoBa and initial data collection were based on a licence granted by the Norwegian Data Protection Agency and an approval from the Regional Committees for Medical and Health Research Ethics (REK). The MoBa cohort is currently regulated by the Norwegian Health Registry Act. The current study was approved by the Regional Committees for Medical and Health Research Ethics (14140 and 2016/1226), and all research was performed in accordance with relevant guidelines and regulations. All mothers and fathers provided written informed consent at recruitment. Mothers consented to participation on behalf of themselves and their children. At 18 years of age, the children become independent participants and receive an information letter about their rights, including how to withdraw. Participation is voluntary, and participants can withdraw their consent at any time. In accordance with REK regulations, individuals who withdraw consent are excluded. As shown in Fig. 1a, MoBa includes questionnaire data collections at many time points, including during the pregnancy, infancy, preschool-age, school-age, adolescence and beyond. Further details about the cohort representativeness and participation in specific waves of data collection are described elsewhere^43,54–56^. Detailed instrument documentation is available on the MoBa website (https://www.fhi.no/en/ch/studies/moba/). A wide range of national health and administrative registries can be linked to MoBa for further phenotyping without reliance on participant engagement in completing questionnaires (Fig. 1b).

#### Phenotypic measures

For our exemplar analyses, we included four phenotypes assessed in middle childhood (age 7–10 years), spanning physical health (height), mental health (depression symptoms) and aspects of functioning (sleep duration and educational achievement). Child height at 7 years of age was reported by mothers in the age-7 questionnaire for the question, ‘What is the child’s height and weight now at 7 years old?’ Mothers were asked to report their child’s current height in centimetres. Sleep duration at 7 years of age was maternally reported in the 7-year-questionnaire on the item ‘Approximately how many hours does the child usually sleep on a weeknight?’ with the following response categories: (1) 8 h or less, (2) 9 h, (3) 10 h, (4) 11 h and (5) 12 h or more. Depressive symptoms were measured using the 13-item Short Mood and Feelings Questionnaire^57^ reported by mothers in the 8-year-questionnaire. We prepared the questionnaire-assessed phenotypes using the phenotools R package (0.2.8) (ref. ^58^) in R 4.1.0^59^. Educational achievement at age 10 was assessed as a composite score based on performance on three national standardized tests of skills in literacy, numeracy and English, derived from the Statistics Norway Educational Registry. The national tests are administered in the autumn of grade 5 (age 10), grade 8 (age 13) and grade 9 (age 14), and are mandatory with exemptions only on application on the grounds that the results will not be useful for assessing the child’s learning due to disability or lack of knowledge of the Norwegian language. Because the raw score ranges differed across subjects and test years, the raw scores were standardized within each test year and subject test. Before standardization, outliers defined as values more than 4 standard deviations (s.d.) from the mean were dropped. The four phenotype scores were standardized for use in the trio analyses to place them on a common scale and to facilitate interpretation and comparison of effect sizes.

Parental phenotypes were not used in the analyses presented in this study. However, similar outcomes in parents to the four exemplar offspring phenotypes were defined to provide supplementary context for interpreting the offspring genetic and phenotypic results. Parental height was self-reported in centimetres in response to the question, ‘How tall are you?’ Educational achievement was derived from Statistics Norway data corresponding to the International Standard Classification of Education (ISCED)^60^ definitions. The ISCED levels from 0 to 8 corresponding to ‘early childhood education (“less than primary”)’, ‘Primary education’, ‘Lower secondary education’, ‘Upper secondary education’, ‘Post-secondary non-tertiary education’, ‘Short-cycle tertiary education’, ‘Bachelor’s or equivalent level’, ‘Master’s or equivalent level’ and ‘Doctoral or equivalent level’ were coded as 1, 7, 10, 11, 13, 14, 16, 18 and 21 years of education, respectively. Sleep problems were self-reported by mothers when the child was 14 years old and by fathers in 2015–2016, using the total score of three items modified from the Karolinska Sleep Questionnaire^61^: ‘How often do you find it difficult to get to sleep at night?’, ‘How often have you woken up repeatedly during the night?’ and ‘How often do you feel tired or sleepy during the day?’ The response options ‘Never’, ‘Less than once a week’, ‘Once per week’, ‘Twice per week’, ‘Three times per week’ and ‘Four times or more per week’, were coded from 0 to 5, respectively. Parental depression symptoms were self-reported by mothers at about week 30 of pregnancy and fathers around week 15 of pregnancy, using the depression subscale of the short eight-item Hopkins Symptoms Checklist (SCL-8)^62^.

Information from the Medical Birth Registry of Norway (MBRN)^63^, a national health registry containing information about all births in Norway from 1967 onwards, and the MoBa questionnaire data were used to identify registered sex, year of birth, multiple births (in the offspring generation) and reported parent–offspring relationships. Before genotyping quality control, all pedigrees were constructed based on the reported parent–offspring relationships for each pregnancy. Each family contained all reported relationships (these included parent–offspring, full-sibling relationships from pregnancies with single and multiple births, and half-sibling relationships). Wherever possible, registered sex was assigned using information from the MBRN. In instances where sex was not registered in the MBRN, the sex registered at birth reported in the MoBa questionnaires was used.

#### Biological data

Blood samples^64^ were collected from participating mothers and fathers at approximately the 17th week of pregnancy during the ultrasound examination. A second blood sample was taken from the mother soon after birth. The blood sample for the child was taken from the umbilical cord after birth. Biological samples were sent to the Norwegian Institute of Public Health, where deoxyribonucleic acid (DNA) was extracted by standard methods and stored^65^.

### Genotyping and quality control

Genotyping of MoBa has been conducted through multiple research projects, spanning several years. Full details about MoBa genotyping are described in Supplementary Methods 1. The MoBaPsychGen pipeline for quality control and imputation was developed to ensure the complex relationship structure and varying selection criteria, genotyping batches and genotyping arrays were handled appropriately (pre-printed^66^). Quality control was performed based on current best-practice protocols^67–71^. Throughout the pipeline, both SNP and individual-level quality control were performed; with priority given to retaining individuals over SNPs, as SNPs can be imputed. The complete pipeline, with full details about quality control, phasing, imputation and post-imputation quality control, is described module by module in Supplementary Methods 2, and a pipeline overview is shown in Supplementary Fig. 1a.

The primary software used throughout the pipeline was PLINK v.1.90b7.2 and v.1.90b6.18 (ref. ^72^) and the R Project for Statistical Computing v.4.0.5 (ref. ^59^) was used to produce figures. Moreover, LiftOver tool^73,74^, KING v.2.2.5 (ref. ^75^), FlashPCA2.0 (ref. ^76^), imputation preparation and checking script v.4.2.13 (ref. ^77^), SHAPEIT2 release 904 (ref. ^78^) with the duoHMM^79^ algorithm, IMPUTE4.1.2_r300.3 (ref. ^80^), QCTOOL v.2.0.8 and v.2.2.0 (ref. ^81^), ic tool v.1.0.8 (ref. ^82^), PLINK2 v2.00a2.3LM (ref. ^72^), IMPUTE2.3.2 (ref. ^83^), yHaplo 2.1.12 (ref. ^84^), the MitoImpute pipeline v.0.2 (refs. ^85,86^), BCFtools v.1.8 and v.1.9 (ref. ^87^), cat-bgen v.1.1.4, COSGAP v.1.0.0 containers (gwas.sif, python3.sif and r.sif from https://github.com/comorment/containers/releases/tag/v1.0.0; for details see the documentation at cosgap.readthedocs.io/en/latest/), Python 3.8.10 and perl v.5.32.1.

### Trio genetic analyses

Exemplar trio analyses were conducted using the four offspring phenotypes. All analyses were carried out on complete case data and restricted to very well-imputed (imputation quality score ≥0.95) autosomal variants available after application of the quality control pipeline. All analyses adjust for offspring age and sex, and account for effects of technical covariates (genotyping batch and first 20 principal components; PC_1–20_). Where analyses estimate or account for ‘indirect genetic effects’, these refer exclusively to variation in child outcomes associated with parents’ genotypes (that is, not those originating with siblings or other family members). The term ‘indirect genetic effects’ is used for consistency with the standards in the field, despite many methods being agnostic as to the specific mechanism, which can include indirect effects of parents, population structure (the presence of subgroups within a population that can bias genetic associations) and assortative mating (the tendency for individuals with similar traits to mate non-randomly, influencing genetic associations in the next generation) among other sources.

#### Trio-GWAS

We conducted within-family GWAS (Supplementary Note 4) using genotype data from parent–offspring trios, focusing on four offspring traits: educational achievement (*n* = 41,761), height (*n* = 23,786), sleep duration (*n* = 24,376) and depressive symptoms (*n* = 18,701). GWAS analyses involved fitting both population and within-family models to the same sample in linkage-disequilibrium-adjusted kinship v.6 (ref. ^88^). To obtain population and within-family genetic association estimates, linear regression was first performed on offspring genotype without adjusting for parental genotype, and subsequently in a mutually adjusted analysis accounting for parental genotypes. The former is synonymous with a conventional principal-component-adjusted model. The latter extends this model by including the parental genotypes as additional covariates to estimate direct genetic effects, while accounting for (and estimating) indirect parental genetic effects. Standard errors were clustered at the family level to account for offspring relatedness. The models were specified as follows (omitting technical covariates for simplicity):

Population model:

$$\mathrm{Outcome}\,=\,{\beta }_{0}+{\beta }_{1}{\mathrm{G}}_{\mathrm{child}}+{\beta }_{2}\mathrm{Sex}+{\beta }_{3}\mathrm{Age}+\varepsilon $$

Within-family model:

$$\mathrm{Outcome}\,=\,{\beta }_{0}+{\beta }_{1}{\mathrm{G}}_{\mathrm{child}}+{\beta }_{2}{\mathrm{G}}_{\mathrm{mother}}+{\beta }_{3}{\mathrm{G}}_{\mathrm{father}}\,+{\beta }_{4}\mathrm{Sex}+{\beta }_{5}\mathrm{Age}+\varepsilon $$

To quantify the attenuation of SNP associations in the within-family relative to population-based models, we compared estimates for genome-wide significant variants reported in previously published GWAS of height^44^, educational attainment^46^, sleep duration^89^ and depression^48^. For each phenotype, we extracted the set of independent genome-wide significant variants from the external GWAS and compared their association estimates in our MoBa population-based and within-family GWAS. Attenuation was calculated as the proportional decrease in SNP effect estimates between the population-based and within-family models, and 95% CIs were derived using a leave-one-out jackknife across the set of included variants. Furthermore, we illustrate the relationship between power and parental heterozygosity rate in Supplementary Fig. 2.

##### Linkage disequilibrium score regression

We used LDSC^90^ (v.1.0.1 with default parameters) to estimate SNP-based heritability (${h}_{\mathrm{SNP}}^{2}$) and genetic correlations (*r*_G_) between our population and within-family GWAS results and publicly available summary statistics from previous studies on height^44^, BMI^45^, educational attainment^46^, smoking initiation^47^ and depression^48^. We input summary-level GWAS data into LDSC, using precomputed linkage disequilibrium scores from the 1000 Genomes Project European superpopulation. For the LDSC *n* parameter, we used the effective sample size (based on standard errors) for each phenotype and model. Effective sample size was computed per SNP using the following formula:

$$\mathrm{Effective}\,n=\left(\frac{1}{{{\rm{s}}.{\rm{e}}.}^{2}}\right)\times \left(\frac{{{{\rm{s}}.{\rm{d}}.}_{{\rm{Resid}}}}^{2}}{(2\times {\rm{MAF}}\times (1-{\rm{MAF}}))}\right)$$

where s.e. is the standard error of the SNP effect estimates, MAF is the minor allele frequency, and s.d._Resid_ is the residual s.d. of the phenotype after covariate adjustment. SNP-based heritability and genetic correlations were calculated separately for the population-based and within-family GWAS models to assess the impact of adjusting for parental genotypes. We examined the attenuation of genetic correlations in within-family analyses compared with population-based models.

#### Polygenic-score-based approaches

Polygenic scores (PGSs) were calculated using LDpred2^91^, implemented in the bigsnpr package v.1.12.21 (ref. ^92^), using the ‘LDPred2-auto’ option and, following established quality control procedures^93^, based on quality control passing, well-imputed variants in MoBa that were present in an extended set of HAPMAP3+ (ref. ^94^) variants. These restrictions resulted in a list of 1,310,867 SNPs. We used a precomputed linkage disequilibrium matrix from UK Biobank as the reference linkage disequilibrium panel^94^. PGS were calculated based on the same external summary statistics used in the LDSC (height^44^, BMI^45^, educational attainment^46^ and smoking initiation^47^). All PGS were adjusted for the first 20 PCs and genotyping batch.

##### Trio-PGS

We performed trio-PGS analyses in a multiple regression framework, incorporating mothers’, fathers’ and children’s PGS for a given trait in a single model to estimate direct and indirect genetic effects on children’s height (*n* = 21,008), educational achievement (*n* = 40,101), sleep duration (*n* = 21,451) and depressive symptoms (*n* = 18,212). The models were specified as follows:

$$\begin{array}{c}\mathrm{Outcome}\,=\,{\beta }_{0}+{\beta }_{1}{\mathrm{PGS}}_{\mathrm{mother}}+{\beta }_{2}{\mathrm{PGS}}_{\mathrm{father}}\\ \,+{\beta }_{3}{\mathrm{PGS}}_{\mathrm{child}}+{\beta }_{4}\mathrm{Sex}+{\beta }_{5}\mathrm{Age}+\varepsilon \end{array}$$

Estimates were calculated with robust standard errors with clustering on maternal IDs to account for the presence of siblings in the data. In sensitivity analyses to assess the sufficiency of this clustering strategy, we restricted to unrelated trios, removing at random all but one trio from families in which children were siblings, half-siblings, or first cousins – leaving the following number of trios per outcome: 14,541 for height, 27,926 for educational achievement, 15,026 for sleep duration, and 12,632 for depressive symptoms. To estimate effects without adjustment for parental indirect genetic effects, we re-ran models without parental PGS.

##### Polygenic transmission disequilibrium testing

PTDT analyses were performed in sub-samples of MoBa families identified by selecting genotyped children with extreme values for height (>2 s.d., above the sample mean; *n* = 527) educational achievement (>90th percentile; *n* = 3,227), sleep duration (below 10th percentile; *n* = 438), and depressive symptoms (>2 s.d. above the sample mean; *n* = 618), where parental average values on equivalent measures were not similarly extreme. In PTDT, the genetic relationship between different traits or conditions is assessed based on the extent of over- or under-inheritance of genetic propensities by individuals selected based on a trait or condition^39^. This is calculated as the average deviation of selected children’s PGS from the mid-parental PGS average, scaled to the standard deviation of the mid-parental PGS. Siblings (*n* = 84 for height, 535 for educational achievement, 78 for sleep duration, and 95 for depressive symptoms) were used as negative controls in these analyses. In our PTDT analyses, we assessed the over- or under-inheritance of genetic variants associated with height^44^, BMI^45^, educational attainment^46^ and smoking initiation^47^.

#### Trio-genome-wide complex trait analysis

Trio-genome-wide complex trait analysis (trio-GCTA)^36^ was run to estimate the additive genetic variance attributable to mothers, fathers and children—across all measured SNPs—on measures of height, educational achievement, sleep duration and depressive symptoms. Genomic-related matrices were estimated for complete trios of genetically inferred European ancestry in which children had phenotypic data for at least one of the four phenotypes. In the case of more than one sibling having phenotypic data available, only one sibling was included at random. To remove individuals related across family trios a bottom-up algorithm from the OpenMendel package^95^ was used with a relatedness threshold of 0.1. The final number of trios, by outcome, was as follows: 12,332 for height, 23,221 for educational achievement, 12,799 for sleep duration and 10,794 for depressive symptoms. Four alternative models were run: (1) estimating the variance explained by the effects of all trio members and the covariance between them (full model); (2) dropping the covariance terms from the full model (no covariance model); (3) estimating the additive genetic effects of the child only (direct effects only model); and (4) estimating no genetic effects (null model). All models included child’s age (in years) at collection of the phenotype, sex, genotyping batch and parents’ first 20 PCs as fixed effect covariates. Akaike’s information criterion was primarily used to select the best model, whereas results of likelihood ratio tests (LRTs) was used to evaluate loss of fit when model parameters were removed. LRT and Bayesian information criterion were consulted for competing models with similar Akaike’s information criterion values. For the likelihood ratio test, we calculated *P*-values using the classical procedure with a single chi-square null distribution when covariance component parameters were removed. When variance component parameters were removed, we calculated *P-*values using the equation below, where $\hat{\lambda }$ denotes the test statistic, and *j* is the number of parameters removed so that the null distribution is a mixture of chi-square distributions dependent on *j* (refs. ^96,97^):

$$P={2}^{-j}\mathop{\sum }\limits_{i=0}^{j}\left(\genfrac{}{}{0ex}{}{j}{i}\right)\,\mathrm{Pr}({\chi }_{i}^{2}\ge \hat{\lambda })$$

LDSC uses GWAS summary data, meaning its power is influenced by both the heritability of the trait and the precision of the underlying GWAS. By contrast, trio-GCTA does not use any summary data and estimates heritability as part of a variance decomposition, using individual-level genetic data. Therefore, although both methods can be used to estimate SNP heritability, their properties (and the underlying data they use) differ substantially.

##### Assortative mating

Whereas trio estimates of direct genetic effects tend to be robust to assortative mating and population structure, estimates of indirect genetic effects can reflect not only genetic nurture effects but also assortative mating and population structure. To aid interpretation of the potential impact of assortative mating on the indirect genetic effects estimates, we calculated spousal Pearson correlations with 95% CIs for the four PGS used in the trio-PGS and PTDT analyses.

### Reporting summary

Further information on research design is available in the Nature Portfolio Reporting Summary linked to this article.

## Online content

Any methods, additional references, Nature Portfolio reporting summaries, source data, extended data, supplementary information, acknowledgements, peer review information; details of author contributions and competing interests; and statements of data and code availability are available at https://doi.org/10.1038/s41586-026-10926-5.

## Supplementary information

Supplementary Text This file contains Supplementary Notes 1–4, Supplementary Methods 1–15 and Supplementary References. The Supplementary Notes and Methods include the theoretical justification of the trio-GWAS approach, a description of the MoBa genotyping batches and the MoBaPsychGen pipeline methods.

Reporting Summary

Supplementary Figures Supplementary Figs. 1–11 with figure legends. Supplementary Figs. 1–3 show an overview of the MoBaPsychGen pipeline, the relationship between power and parental heterozygosity rate, and a Directed Acyclic Graph relevant to the Theoretical justification of the trio-GWAS approach, respectively. Supplementary Figs. 4–11 are from the implementation of the MoBaPsychGen pipeline

Supplementary Tables The Supplementary Tables provide an overview of the MoBa genotyping batches and the number of SNPs (autosomal, chromosome X and pseudoautosomal) and individuals passing various stages of the MoBaPsychGen pipeline

Peer Review file

## Figures and Tables

**Extended Data Table 1:**
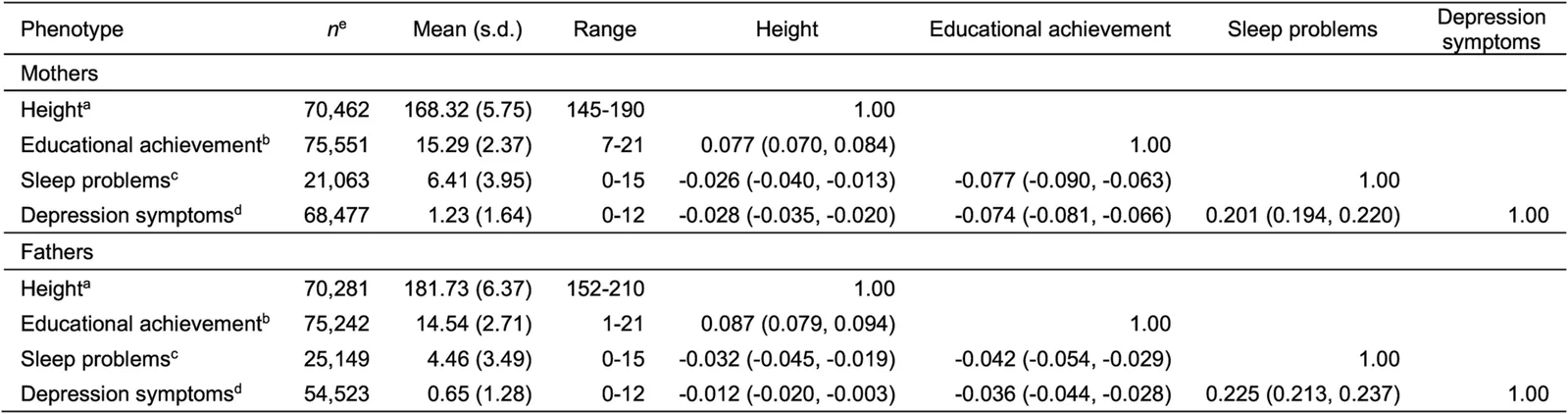
Descriptive statistics and Pearson correlation matrix (with 95% confidence interval) for similar outcomes to the four offspring phenotypes in mothers and fathers

^a^Height, in centimeters, were self-reported. ^b^Educational achievement, in years, derived from Statistics Norway data corresponding to the International Standard Classification of Education (ISCED) definitions. ^c^Sleep problems were self-reported using a three-item modified version of the Karolinska Sleep Questionnaire. ^d^Depression symptoms were self-reported using the depression subscale of the short eight-item Hopkins Symptoms Checklist (SCL-8). See Methods for full description of how phenotypes were derived. ^e^Number of parents with genotyped children.

**Extended Data Table 2:**
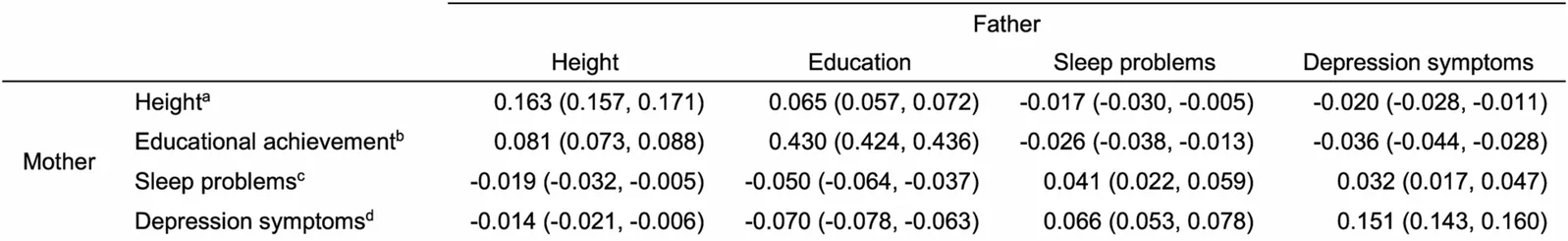
Spousal Pearson correlation matrix (with 95% confidence intervals) for similar outcomes to the four offspring phenotypes

^a^Height, in centimeters, were self-reported. ^b^Educational achievement, in years, derived from Statistics Norway data corresponding to the International Standard Classification of Education (ISCED) definitions. ^c^Sleep problems were self-reported using a three-item modified version of the Karolinska Sleep Questionnaire. ^d^Depression symptoms were self-reported using the depression subscale of the short eight-item Hopkins Symptoms Checklist (SCL-8). See methods for full description of how phenotypes were derived.

**Extended Data Table 3:**
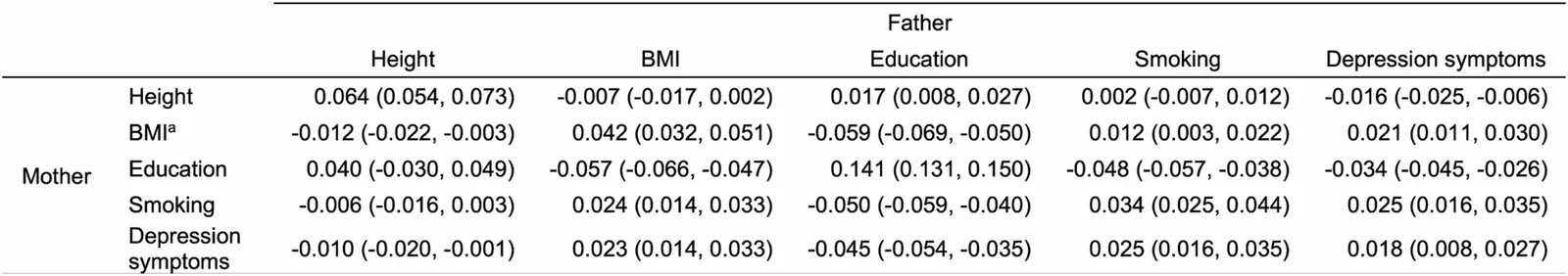
Spousal polygenic score (PGS) Pearson correlations (with 95% confidence intervals)

^a^Body mass index.

**Extended Data Table 4:**
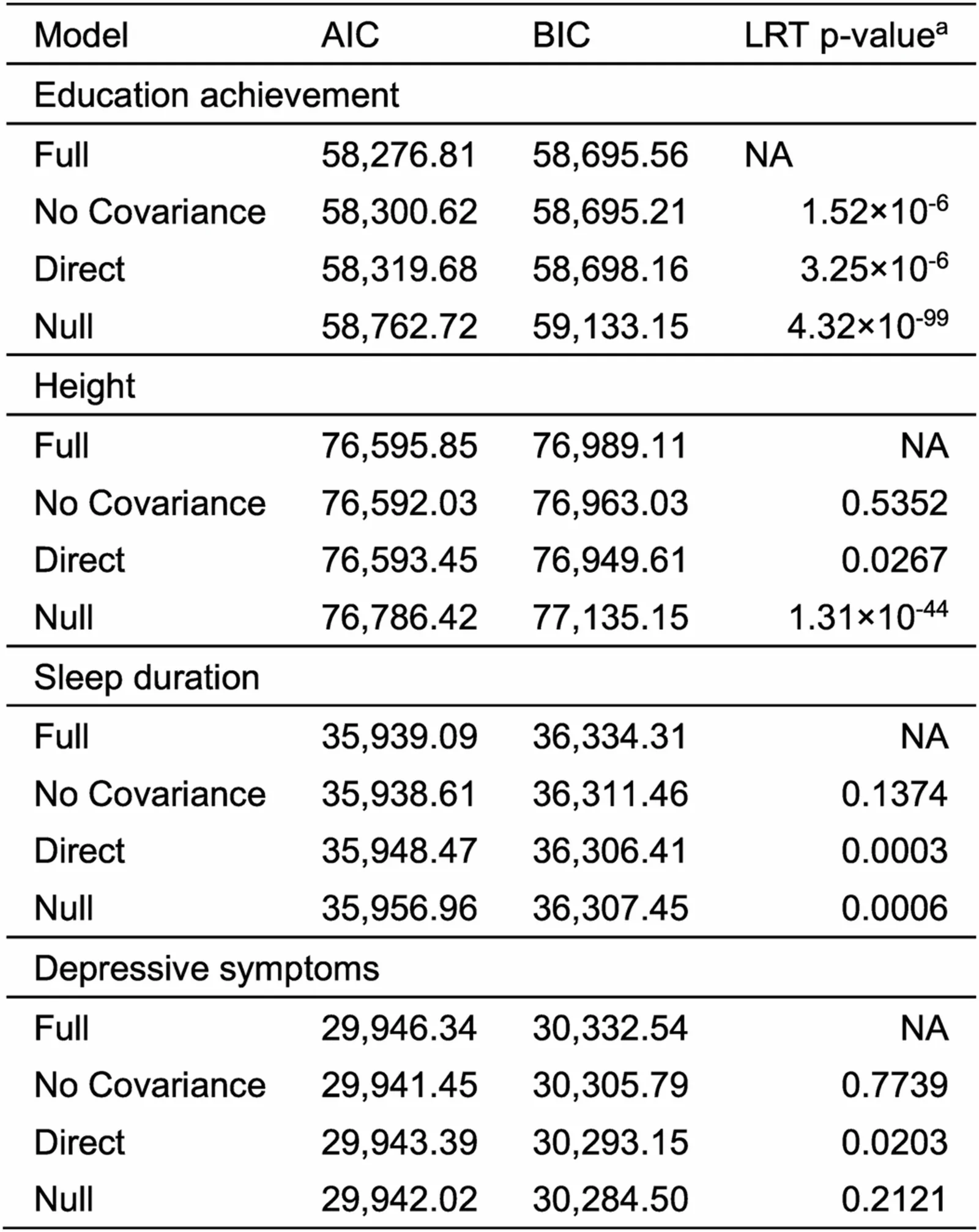
Fit statistics for the Trio-GCTA models

GCTA: genome-wide complex trait analysis. AIC: Akaike Information Criterion. BIC: Bayesian Information Criterion. LRT: likelihood ratio tests. Akaike’s Information Criterion (AIC) was primarily used to select the best model. When AIC or BIC were inconclusive LRT test was used as the deciding factor for the best fitting model. ^a^Comparison to model in previous row. When variance components removed, p-values calculated with a null distribution that is a mixture of chi-square distributions (see Methods).

## Data Availability

Data from MoBa are managed by the Norwegian Institute of Public Health. The MoBa cohort is currently regulated by the Norwegian Health Registry Act. The data that support the findings of this study are available from the Norwegian Institute of Public Health, but restrictions apply to the availability of these data. The MoBa website provides details on how to obtain access for research and the available variables (https://www.fhi.no/en/ch/studies/moba/for-forskere-artikler/research-and-data-access/). Access to individual-level data from MoBa and national registries requires approval from the Regional Committees for Medical and Health Research Ethics (REK), compliance with the General Data Protection Regulation (GDPR) and data holder approval. Researchers must apply through https://helsedata.no/en/. International researchers need to collaborate with a principal investigator employed at a Norwegian research institution—the MoBa administration team (mobaadm@fhi.no) can facilitate contact with relevant research environments. MoBa can be used for health research. The purpose of MoBa is to generate new knowledge about the causes, risk factors, courses and consequences of diseases and health outcomes, with the ultimate goal of improving prevention and treatment. The MoBa administration normally takes 3–5 weeks to reach a decision from the time a complete application has been received. The expected processing time from the decision is in place until data delivery, which is normally between 4 and 5 weeks. Delivery of linked data from health registries usually takes up to 8 weeks. Updated information about processing time is available on the MoBa website (https://www.fhi.no/en/ch/studies/moba/for-forskere-artikler/research-and-data-access/). Information on how to access the MoBaPsychGen post-imputation quality-controlled data is available by contacting the MoBa administration and on the following website: https://www.fhi.no/en/more/research-centres/psychgen/access-to-genetic-data-after-quality-control-by-the-mobapsychgen-pipeline-v/. All summary statistics from GWAS conducted in this study will be available on the MoBa website for GWAS summary statistics: https://www.fhi.no/en/ch/studies/moba/for-forskere-artikler/gwas-data-from-moba/. Participant consent does not allow individual-level data storage in repositories or journals. Moreover, the following external reference panels and linkage disequilibrium resources were used in the MoBaPsychGen pipeline and exemplar trio analyses: the publicly available (access controlled) European Genome–Phenome Archive (Study ID EGAS00001001710) Haplotype Reference Consortium (HRC) release 1.1 (https://ega-archive.org/studies/EGAS00001001710, https://ega-archive.org/access/request-data/how-to-request-data/); the Genome Aggregation Database (gnomAD) v.3.1.2 (https://gnomad.broadinstitute.org/downloads); the MitoImpute reference panel restricted to SNPs with minor allele frequency above 0.1% (https://github.com/sjfandrews/MitoImpute/tree/master); the 1000 Genomes Project Phases 1 and 3 (https://www.internationalgenome.org/data/, https://www.cog-genomics.org/plink/2.0/resources#phase3_1kg); pre-computed linkage disequilibrium scores from the 1000 Genomes Project European superpopulation (https://data.broadinstitute.org/alkesgroup/LDSCORE/eur_w_ld_chr.tar.bz2); and pre-computed linkage disequilibrium matrix from UK Biobank (https://figshare.com/articles/dataset/LD_reference_for_HapMap3_/21305061).

## Code availability

Scripts used throughout the MoBaPsychGen pipeline are available on GitHub: https://github.com/psychgen/MoBaPsychGen-QC-pipeline. Similarly, the analytic code for the exemplar trio analyses is available in an additional GitHub repository: https://github.com/psychgen/moba-trio-analyses.
