# Supplementary material for: Family genetic designs in MoBa provide insights into health and functioning
Source: Nature. 2026 Aug 19;658(8136):707–15. doi: 10.1038/s41586-026-10926-5 (PMC13668695; doi:10.1038/s41586-026-10926-5)
Supplement: Supplementary file 1 [file 41586_2026_10926_MOESM1_ESM.pdf]

nature portfolio

https://doi.org/10.1038/s41586-026-10926-5

Supplementary information

Family genetic designs in MoBa provide insights into health and functioning

In the format provided by the authors and unedited

Supplementary Note 1 – Rationale for MoBaPsychGen pipeline development

As the field has progressed, scripted open-source quality control (QC) and imputation pipelines for genotype data, such as the Rapid Imputation and Computational PipeLIne for Genome-Wide Association Studies (RICOPILI)[68] and BIGwas[98], have been developed. These pipelines are designed to be arbitrarily used by cohorts or studies and are typically updated to include the most up-to-date best-practice procedures[67,69]. Despite continued improvements in QC practices, handling complex relatedness in processing of genotyped samples remains a major challenge; and harmonising across multiple batches when complex relatedness exists between batches is not always addressed. Furthermore, robustly performing QC in cohorts with diverse ancestries (i.e., participants clustering with multiple reference such as HapMap[99], 1000 Genomes Project[100], Human Genome Diversity Project[101] superpopulations: African, East Asian, South Asian, European, Admixed American). A basic framework has been developed for performing QC in cohorts with diverse ancestral backgrounds[102], however, it doesn't address the challenge of preforming QC in cohorts with large imbalances in superpopulation sizes.

Many of the largest longitudinally studied pregnancy and birth cohorts available today consist of related individuals. A key benefit of cohorts with related individuals is the ability to perform complex genetic analyses, by explicitly modelling the relatedness. Cohorts with large numbers of related individuals can be used to address unique research questions, which would not be possible in cohorts comprising only unrelated individuals. These include investigations of genetic and environmental mechanisms of intergenerational transmission[36], and interrogations of the role of familial confounding in observational associations[2].

However, to exploit the novel analytical opportunities available using increasingly complex family cohorts, family-based QC protocols need to be performed, otherwise the researcher risks inducing a bias in results. To our knowledge, the Pedigree Imputation Consortium Pipeline (PICOPILI)[66] is the most used scripted open-source family-based QC pipeline available. PICOPILI offers scripts for pre-imputation QC, phasing, imputation, post-imputation QC, and genome-wide association studies (GWASs) of family data with arbitrary pedigrees, alongside tools for imputation and family-based analyses. However, some of the software used in PICOPILI is not designed to handle large sample sizes. Furthermore, generic open-source pipelines such as PICOPILI are – by design – not equipped to handle idiosyncrasies of specific cohorts or studies. As such, other studies have also had to adapt in house protocols tailored to meet the cohort specific characteristics. For example, QC pipelines optimized to account for high levels of recent admixture and population diversity[103,104]. We were unable to use a pre-existing pipeline for the Norwegian Mother, Father, and Child Cohort Study (MoBa) due to (1) the cohort size, (2) the complex interrelatedness, and (3) the idiosyncrasies of MoBa (e.g., inclusion of within and across batch duplicates and the large imbalance in number of individuals clustering with reference superpopulations).Therefore, we developed the MoBaPsychGen pipeline to manually inspect and benchmark performance of the various QC stages in the context of a very large sample size with imbalanced batches, population structure, and relatedness. Future work is required to automate the process based on the empirical learning from this process to meet strict standards such as re-running of various stages following consent withdrawals.

Supplementary Note 2 – Additional results information

The MoBaPsychGen pipeline (Supplementary Methods 2, Supplementary Figure 1a) was applied to genotyped individuals in MoBa for which genotyping has been conducted across multiple research projects spanning several years, in batches selected based on varying criteria and processed using different genotyping arrays at different genotyping centres (Supplementary Note 3).

The 26 MoBa genotyping batches comprised 234,505 successfully genotyped samples with valid informed consent as of January 2019. Approximately 95% of genotyped participants clustered with the 1000 Genomes Project European superpopulation, illustrating MoBa is a quite homogenous cohort. The large imbalance of individuals clustering with the 1000 Genomes Project European superpopulation resulted in small numbers of individuals clustering with the African, Admixed American, East Asian, and South Asian 1000 Genomes Project superpopulations, which were spread across the 26 genotyping batches. This made it incredibly challenging to apply ancestry adjusted QC approaches to MoBa, given some genotype batches included <5 individuals clustering with specific superpopulations. After post-imputation QC 6,981,748 autosomal single nucleotide polymorphisms (SNPs) and 207,569 unique individuals remained (corresponding to 90% of the unique individuals genotyped). Of these SNPs, 777,816 were directly genotyped in at least one imputation batch, with 94% SNPs imputed, on average, in each batch (Supplementary Figure 1b) and high imputation quality in all batches (Supplementary Figure 1c). Across the six imputation batches, a total of 76,577 children, 53,358 fathers, and 77,634 mothers were genotyped (Supplementary Figure 1d).

Meanwhile, after post-imputation QC 174,462 chromosome X and 3,200 pseudoautosomal region (PAR) SNPs (of which 17,954 and 377, respectively, were directly genotyped) were available in a subset of these individuals (N = 204,913 and 135,593, respectively). An overview of the number of SNPs and individuals passing the various QC modules is available in Supplementary Tables 2-3.

Reported parent-offspring relationships and relatedness checks performed throughout the pipeline using genetic data allowed us to identify within-generation and across-generation zeroth-degree (monozygotic twins), first-degree (full-siblings, parent-offspring), second-degree (half-siblings, avuncular pairs, grandparent-grandchild), and third-degree (first cousins) relationships (Supplementary Figure 1e). The 207,569 individuals passing autosomal post-imputation QC comprised 64,471 nuclear, blended (including biological and step-relatives), and extended families ranging in size from singletons to large families with greater than 30 unique individuals (singletons are included as families as other family members may not have been genotyped, imputed, or passed post-imputation QC). The identified family sizes illustrate the complexity of the pedigrees in MoBa.

The mitochondrial haplogroup for each individual was inferred using the script from the MitoImpute pipeline. In the Trøndelag Health Study (HUNT), a population-based cohort from Nord-Trøndelag county in Norway, the most prevalent mitochondrial haplogroups were H, U, and J, constituting 40%, 17%, and 15% of the sample, respectively[105]. In line with this, in MoBa, we identified the H haplogroup as the most prevalent (41% of the total sample), followed by the U (16%), and J (11%) haplogroups.

The chromosome Y haplogroup for each individual was inferred using the yHaplo software. The most prominent haplogroups identified in MoBa were R1b (31%), I1 (30.8%), and R1a (25.7%). In a previous study of Norwegian individuals[106], based on five variants and therefore only able to distinguish broader haplogroup categories, the most prevalent haplogroups were reported as BR(xDE,J,N3,P) – which includes I1, P*(xR1a) – which includes R1b, and R1a (using YCC 2002[107] nomenclature), with frequencies of 37%, 31%, and 26%, respectively. In MoBa, using the same broader haplogroups as the previous study, we found these broader haplogroups to be present at 37.4%, 32.6%, and 25.7%, respectively.

Supplementary Note 3 – Additional pipeline discussion

We developed and applied a QC and imputation pipeline capable of handling genotyping data from large population-based samples of individuals with complex interrelatedness. The MoBaPsychGen pipeline was designed for, and applied to MoBa, a cohort with a highly complex pattern of relatedness and for which genotyping has been conducted across multiple research projects spanning several years, in batches selected based on varying criteria, and processed using different genotyping arrays at different genotyping centres. Individuals passing QC with the MoBaPsychGen pipeline represented 90% of the unique originally genotyped samples, with information on approximately seven million SNPs available for individuals in a final merged dataset.

Most currently available QC pipelines are structured to handle genotype data from unrelated individuals only or samples with a simple relatedness structure (e.g., nuclear families). As genotyping continues to decrease in cost and processing time, increasing numbers of people will be genotyped for research – including both in existing cohort samples with complex interrelatedness by design, and in convenience samples where complex relationships become inevitable as sample size increases (e.g., communities or small countries). Instead of excluding these valuable relationships, they can be leveraged in analyses to provide not only larger sample sizes, but also important discoveries, provided the data is processed in such a way that complex interrelatedness is accounted for and does not have the potential to bias results.

In developing the MoBaPsychGen pipeline, we focused on ensuring comprehensive coverage of key QC steps with particular importance for the MoBa sample. Firstly, the duplicate QC, which provides an indication of the heterogeneity within and between both genotyping batches and imputation batches. Through the removal of discordant SNPs, based on the relatively high number of duplicate or triplicate genotype samples in MoBa, we ensure that genotyping errors are identified and addressed. Secondly, the relatedness check, which corrects inaccurate pedigree assignment. With a high number of known relationships in cohort samples such as MoBa, it is possible to identify any sample mix-ups or unexpected duplicates – but doing so requires specific, manually-overseen steps in the QC pipeline. Furthermore, having as many relationships as possible coded in the pedigree is also important because: (1) only founders are used in some of the basic SNP QC steps (e.g., minor allele frequency [MAF] and Hardy-Weinberg equilibrium [HWE]) and (2) without correct pedigree information, the estimated haplotypes and resulting imputed genetic data will be affected, resulting in higher likelihood of switch errors in the haplotype estimation and Mendelian errors (ME) in the imputed data[79]. It is particularly important that these key steps are performed accurately throughout the QC pipeline to ensure the highest quality data.

The MoBaPsychGen pipeline can be adapted to other cohorts containing related individuals. Conducting quality control procedures on a cohort of genetically related individuals comes with inherent challenges. Added to this, additional complexities including duplicate samples, distinct genotyping batches of varying sizes based on varying selection criteria and processed on different genotyping arrays are idiosyncratic characteristics of the MoBa sample that present challenges for the QC process but which, nonetheless, may feature to varying degrees in other cohorts. The steps taken to meet these challenges in the MoBaPsychGen pipeline should provide an informative basis for those carrying out quality control in other large-scale population-based cohorts, particularly those with family data. Therefore, as individuals are recruited into cohorts in larger numbers, the prevalence of relatedness between participants and other complexities, such as having multiple genotyping batches, will increase. The MoBaPsychGen pipeline provides an example of how to handle these complexities.

The challenges and intricacies of using genomic data from samples with complex interrelatedness do not end with QC. Indeed, it is important that users understand the implications and decisions of the QC process and interact correctly with its outputs according to their analytic needs. To this end, for the MoBa genetic data to which the MoBaPsychGen pipeline was applied in this paper, and which is available in post-imputation QC form to MoBa researchers on request (see https://www.fhi.no/en/more/research-centres/psychgen/access-to-genetic-data-after-quality-control-by-the-mobapsychgen-pipeline-v/), we have developed an R package, *genotools* (https://github.com/psychgen/genotools) as a companion to the QCed genetic data. This package aims to facilitate accurate, efficient, and reproducible use of MoBa genetic data, and currently has functionality to help users work with genetic datafiles alongside supporting identification, linkage, and covariates files, identifying relationships based on analysis types, creating and adjusting polygenic scores, and will continue to be developed to help researchers maximize the potential of analyses using MoBa genetic data processed via the MoBaPsychGen pipeline.

Many novel methods can make use of genetic data from related individuals to gain additional insights into the nature of genetic effects that are not accessible using population-based data. These include variations of GWASs (e.g., within-family GWAS, sibling GWAS, and trio-GWAS analyses), genomic-relatedness matrix-based approaches (e.g., Trio-GCTA, M-GCTA[108]), polygenic score-based approaches (e.g., trio polygenic score analyses, transmitted and non-transmitted allele polygenic score analyses, rGenSi modelling[40], polygenic transmission disequilibrium testing) and others (e.g., within-family Mendelian Randomization). It is likely that methodological development in this space will continue at a high rate over the coming years. Appropriately preparing genomic data in relevant samples for inclusion in analyses using this wide array of approaches is an essential upstream component of research into a range of important topics, including familial aggregation of risk for health problems, putative causal risk factors (including parental factors) in children's environments, comorbidity between health problems, factors influencing early life neurodevelopment and many more. Furthermore, knowledge about the specific degrees of relatedness within a cohort allows for contribution to larger consortia of certain family types, for example, sibling, twin, and children of twin consortia.

Every effort was taken to ensure the MoBaPsychGen pipeline adhered to the current best-practice protocols for preforming QC while appropriately handling the idiosyncratic

characteristics of the MoBa genotyping data. However, there are a few limitations of the pipeline. Firstly, the Global Screening Array (GSA) genotyping batches were not phased and imputed in a single imputation batch because the genotyping batches were received at varying timepoints after imputation of other GSA batches had already been performed. Secondly, the usage of 1000 Genomes Project phase 1 reference, with visual inspection. However, the use of supervised clustering in MoBa using 1000 Genomes Project phase 3 as the reference revealed minimal differences in identification of individuals clustering with the European superpopulations. Thirdly, since the majority of MoBa participants are of European genetic ancestry, the analyses were restricted to this homogenous ancestry group. Multi-ancestry QC and imputation should be implemented for future studies.

Furthermore, for transparency and reproducibility purposes, detailed documentation of our protocols and scripts used to perform QC, imputation and downstream analyses have been shared exactly as originally used, including hard-coded paths (see "Code Availability" statement). This approach was taken in alignment with other large studies, which have provided excellent documentation but have not released a general open-source software tool(s) that can be readily applied to other datasets[12,14,15]. We understand and acknowledge, that this is a limitation from a portability perspective. However, given the MoBaPsychGen pipeline is a tailored in-house protocol with many elements bespoke to MoBa, rather than as a generic open-source software pipeline, we do not anticipate other cohorts applying the pipeline in its current form.

The MoBaPsychGen pipeline handles complex relatedness and other specific challenges in the process of quality controlling genotype data from the MoBa cohort. The pipeline has potential utility in similar cohorts, and MoBa genetic data processed via the pipeline is available and well-suited to a wide range of highly informative downstream genetic analyses - including those that make particular use of the complex relatedness in such a sample.

Supplementary Note 4 – Theoretical justification of the Trio-GWAS approach

Family-based designs have a long history in statistical genetics. Abecasis et al. (2000) made an important contribution, deriving a QTDT test – a quantitative transmission disequilibrium test (QTDT)[109]. This test examines whether a quantitative (continuous) trait is associated with the transmission of genetic variants from parents to offspring, using either parental genotypes or sibships. In the case of trio data being available, the QTDT estimation model for the outcome includes the child and parental genotypes. They formally derived within and between family components, and argued that the random transmission of variants from parents to offspring can be used to overcome population stratification. Gauderman (2003) describes similar models in the context of candidate gene studies in which the offspring genetic effect is conditioned on the parental genotype[110]. Wheeler and Cordell (2007) extend these methods to quantitative traits in case-control samples[111]. Each of these methods exploits the random transmission of genetic variants from parents to offspring to obtain estimates of the direct effects of genetic variants.

Here, we use within-family trio-GWAS estimators which is estimated using the following model:

$$y_i = \alpha + \Gamma_{1,j} g_{i,j} + \Gamma_{2,j} g_{im,j} + \Gamma_{3,j} g_{if,j} + \boldsymbol{\beta} \boldsymbol{X_i} + e_{i,j}$$

This was described in Brumpton et al. (2020)[112], and is very similar in form to the QTDT model, and other recent papers[113]. Where the offspring phenotype is indicated by y_i, the offspring, mother and father genotype at locus j is indicated by $g_{i,j}$, $g_{im,j}$, and $g_{if,j}$, respectively. The residual or error term is indicated by $e_{i,j}$ which is modelled using cluster-robust standard errors allowing for within-family residual dependence. The matrix of covariates $\boldsymbol{X}$ include genetic principal components. Note, these are included to increase the efficiency of the estimator and control for bias in the parental effect estimates from population stratification, not to control for bias in the estimated direct effect of offspring genotype. The direct effect tagged by the genotype is estimated by $\Gamma_{1,j}$. The indirect effects of the maternal and paternal genotypes are indicated by $\Gamma_{2,j}$ and $\Gamma_{3,j}$, respectively. They may be biased by residual confounding from population stratification after attempting to adjust for genetic principal components. As with QTDT above, the association of the offspring genotype with the phenotype, conditional on parental genotypes, can provide an unbiased estimate of the direct effects of the genotype, absent other forms of bias (e.g. incomplete linkage disequilibrium with the tagged causal variant, selection/collider bias). This follows from the random transmission of genetic variants from parents to offspring and can be demonstrated using the following Directed Acyclic Graphs (Supplementary Figure 3)[112,114].

There are three causes of offspring genotype, $g_{i,j}$, mother and father's genotypes $g_{im,j}$ and $g_{if,j}$ and chance due to random Mendelian inheritance. Hence, once parental genotype are controlled for via statistical adjustment in a linear model, assuming no measurement error in the parental genotype measures, the confounding paths from parental genotype to the phenotype, any backdoor path that acts via parental genotype, which includes population structure, assortative mating and dynastic effects, are blocked. Hence, absent other mechanisms (e.g. selection bias) adjusting for parental genotype allows for unbiased estimation of the tagged direct genetic effects. However, the interpretation of the coefficients on the parental genotypes is more complex, as any factor that affects parental genotype and the outcome can induce associations. Thus, the coefficients on the parental genotype may not merely reflect genetic nurture, but could also reflect bias from residual population stratification.

Supplementary Methods 1 – Genotyping of MoBa

Three research projects (HARVEST, SELECTIONpreDISPOSED, and NORMENT) provided genotype data to MoBa Genetics (https://github.com/folkehelseinstituttet/mobagen) while PsychGen pipeline was being developed and run. In total, 238,001 MoBa samples were sent to be genotyped in 24 genotyping batches with varying selection criteria, genotyping arrays, and genotyping centres (Supplementary Table 1). Two genotyping batches were split into sub-batches for technical reasons; the 26 batches and sub-batches are henceforth referred to as batches. The 26 MoBa genotyping batches comprised 235,412 successfully genotyped samples, comprising 80.6% of the individuals in MoBa.

As a quality control measure, 397 individuals were deliberately genotyped more than once. Furthermore, genotyping efforts were conducted independently, which resulted in numerous individuals being genotyped multiple times. In total, there were 4,035 individuals genotyped multiple times, of which 3,958 were genotyped twice and 77 genotyped three times.

Genotyping in the HARVEST and SELECTIONpreDISPOSED projects

Two HARVEST batches, comprising approximately 11,000 full mother, father, and child trios (approximately 33,000 individuals), were genotyped in 2014 and 2015. Genotyping, following the Illumina Infinium HD ultra-protocol with scanning performed using the Illumina iScan, and the conversion of scanned data to genotype calls, using GenomeStudio_v2011.1 with GenTrain_v2, was conducted at the Genomics Core Facility, Trondheim, Norway. Two Illumina HumanCoreExome (HCE) arrays were used to genotype the batches. The HARVEST12 batch was genotyped using the Illumina HCE12v1.1 array. The HARVEST24 batch was genotyped using the Illumina HCE24v1.0 array. Because of a difference in signal intensities, the HARVEST12 batch was re-clustered into two sub-batches: HARVEST12a and HARVEST12b. Additionally, the HapMap sample NA12878 was genotyped twice as an external control.

An additional 6,000 full trios were genotyped in 2017 (ROTTERDAM1 batch) and a further 3,000 trios were genotyped in 2018 (ROTTERDAM2 batch). Collectively, these two batches comprised approximately 9,000 full mother, father, and child trios (approximately 27,000 individuals). Genotyping using the Illumina GSA MD v.1.0 array and the conversion of scanned data to genotype calls using GenomeStudio was conducted at the Erasmus MC, Rotterdam, Netherlands.

The full trios were selected randomly, while minimizing the number of trays taken out of the freezer, but were excluded if: (1) the pregnancy resulted in a stillbirth; (2) a sample from the trio was deceased (at the time the sample list was created); (3) the pregnancy resulted in a multiple birth (multiple births in the parent generation were not excluded); (4) data for the pregnancy is missing in the Medical Birth Registry of Norway (MBRN) data; (5) missing anthropometric measurements at birth in the MBRN; (6) not included in the mother-reported first questionnaire (Q1) (as a proxy for higher fallout rate); and (7) missing DNA samples.

Genotyping in the NORMENT project

Genotyping of 20 batches with varying selection criteria and genotype arrays, and GenomeStudio conversion of scanned data to genotype calls were conducted at deCODE Genetics in Reykjavik, Iceland.

ADHD batches

Two data selections with the same criteria were conducted for the Common and rare genetic risk factors for attention-deficit/hyperactivity disorder (ADHD) subproject. The initial selection criteria were: (1) data from the MBRN available for the index pregnancy (the pregnancy with an ADHD diagnosis); (2) DNA available for the index pregnancy; (3). index pregnancy not stillborn or deceased; and (4) DNA available for the parents. Additional criteria for: (1) case children–ADHD diagnosis (International Classification of Diseases version 10 code F90) from Norwegian Patient Registry (NPR) which contains all diagnosis from specialist health service in Norway from 2008 onwards; (2) control children–no ADHD diagnosis in NPR plus birth year, registered sex, and birth hospital or county matched with case children; (3) mothers of case children–DNA available from mother at any time point, including from another pregnancy; and (4) fathers of case children–DNA available from father at any time point, might come from another pregnancy, and independent of DNA availability from the mother. The ADHD1 batch included 5,818 individuals, which were sent for genotyping using the

Illumina Infinium OmniExpress (OMNI) 24v1.2 array. The ADHD2 batch included 2,502 individuals, which were sent for genotyping using the Illumina GSA MD v.1.0 array.

NORMENT parent only batches

The NORMENT-Jan2015 and NORMENT-Jun2015 batches comprised 6,040 unrelated parent samples, which were sent to be genotyped as population controls for planned analyses, using the Illumina Human OMNI 24v1.0 array. The only selection criteria was that individuals were excluded if they were part of a multiple birth.

NORMENT trio batches

The NORMENT-May16 batch included approximately 6,452 full trios, which were sent for genotyping using the Illumina Infinium OMNI 24v1.2 array. Similarly, NORMENT-Feb18 batch included approximately 3,280 full trios, which were sent for genotyping using the Illumina GSA MD v.1.0 array. Almost identical selection criteria were used for the NORMENT trio batches as the HARVEST and SELECTIONpreDISPOSED projects, with the exception that the mother did not answer the first questionnaire (Q1) the trio was not excluded.

NORMENT remaining MoBa samples

From 2020 onwards, the genotyping batches contained as many individuals from the same families as possible. However, the primary goal was to efficiently genotype the remaining samples from the MoBa cohort, with no selection criteria enforced other than having already been genotyped (meaning stillborn and deceased pregnancies or deceased individuals will be present in these batches). Therefore, the percentage of relatedness within the batches decreased with time.

The NORMENT-Feb2020 batch included 17,930 individuals with a moderate percentage of first-degree relatives, which was sent for genotyping using the Illumina GSA. Due to the availability of BeadChips the NORMENT-Feb2020 batch was genotyped using two versions of the array and was therefore separated into two sub-batches: NORMENT-Feb2020_v1 and NORMENT-Feb2020_v3. The majority (13,505) of samples were genotyped using the Illumina GSA MD v.1.0, forming the NORMENT-Feb20-v1 sub-batch. However, a quarter of the samples (4,418) were genotyped using the Illumina GSA MD v.3.0, forming the NORMENT-Feb20-v3 sub-batch.

All remaining batches were genotyped using the Illumina GSA MD v.3.0 array and contained only a small percentage of first-degree relatives, with the last batch containing unrelated individuals only. The remaining 13 batches, NORMENT-Aug2020_996, NORMENT-Aug2020_1029, NORMENT-Nov2020_1066, NORMENT-Nov2020_1077, NORMENT-Nov2020_1108, NORMENT-Nov2020_1109, NORMENT-Nov2020_1135, NORMENT-Nov2020_1146, NORMENT-Mar2021_1273, NORMENT-Mar2021_1409, NORMENT-Mar2021_1413, NORMENT-Mar2021_1531, NORMENT-Mar2021_1532, comprised 25,004, 25,004, 25,002, 4,700 4,794, 5,640, 4,606, 5,264, 5,450, 2,703, 5,639, 1,974, and 219 samples sent for genotyping, respectively.

Supplementary Methods 2 – Implementation of the family-based MoBaPsychGen genotype QC pipeline

Module 0: Harmonize genotype data

Harmonization of the genotype files was conducted (as needed for each batch) using PLINK[115] binary file format, including (1) updating SNP alleles (convert from Illumina A/B to the genetic A, C, T, and G alleles on the plus strand); (2) excluding problematic SNPs previously reported by the Psychiatric Genomics Consortium (PGC)[116]; (3) updating SNP names to rsIDs; and (4) converting chromosomal positions to match the genome build of the phasing and imputation reference panel (GRCh37/hg19), using the LiftOver tool[73,74]. The fam files were then updated to include the reported pedigree and registered sex.

Initially, the 234,505 genotyped samples (230,436 unique individuals) were assembled into 90,861 nuclear and blended families constructed based on the reported parent-offspring relationships for each pregnancy. The initial nuclear and blended family size based on reported parent-offspring relationships ranged from singletons (where the other family members were not genotyped due to missing blood samples, genotyping failures, etc.) to eight unique individuals. The initial nuclear and blended families only included across generation parent-offspring relationships for each pregnancy, which enabled the identification of full-sibling or half-sibling in the child generation based on having the same reported parent (i.e., does not include relationships inferred using genetic data).

Module 1: Classify MoBa individuals against 1000 Genomes Project ancestral populations

To perform reliable genotype QC and manage confounding bias due to population stratification (systematic differences in MAF between populations[117]), one convention is to perform analyses within major ancestral groups. We took the following steps to cluster MoBa individuals against Asian, European, and African super-populations as characterised in the 1000 Genomes Project phase 1 reference dataset[100] (Supplementary Methods 3). SNPs were temporarily removed from the complete genotype data based on the following criteria: (1) call rate<95%; (2) out of HWE at p-value<1.00×10^{-3} (within founders only to account for relatedness); and (3) MAF<1% (within founders only to account for relatedness). Individuals were temporarily removed with call rate<95%. (permanent SNP and individual removal were performed in module 2). The MoBa genotype batch was merged with 1000 Genomes Project phase 1[100] (N=1,083 unrelated individuals) and principal component analysis (PCA) was performed on the combined dataset (Supplementary Methods 4). To perform the PCA, linkage disequilibrium (LD) pruning was performed using the PLINK indep-pairwise parameter with a window size of 3,000, step size of 1,500, and r^2 threshold of 0.1. Additionally, SNPs in long-range high LD[118] regions were removed. Principal components (PCs) were first estimated within founders (based on reported information). The non-founders, all individuals with at least one reported parent, were then projected into the PC space of founders.

Individuals were assigned to ancestry populations based on visual inspection using the first seven PCs, with boundaries drawn and individuals far from population clusters removed due

to the complexity of imputing admixed individuals There is on-going discussion about how to define and manage genetic ancestry in epidemiological analyses practicably and accurately[119], and classification into discrete ancestral groups is used as a convenient simplification here as the vast majority of MoBa participants clustered with a single reference superpopulation. As new approaches emerge to appropriately handle the continuous nature of ancestry in cohorts with large imbalance of individuals clustering with a single reference superpopulation we will update our QC procedures.

Module 2: QC of ancestry populations

Pre-imputation QC of the ancestry populations was performed in multiple rounds to ensure the validity of checks performed as individuals and SNPs were removed throughout the pipeline. For MoBa, three rounds were enough to ensure a robust QC was performed with only SNPs and samples with reliable quality metrics passing through the pipeline. Module 2 was performed separately for each of the major ancestry populations in MoBa.

Basic QC

Initially a basic QC was performed where SNPs and individuals were removed based on the following filters: (1) SNPs with MAF<0.5% (within founders only to account for relatedness); (2) SNPs with call rate<95%; (3) SNPs with call rate<98% (Supplementary Methods 5); (4) SNPs and individuals with a call rate<98%; and (5) SNPs out of HWE at p-value<1.00×10^{-6} (within founders only to account for relatedness). Heterozygosity filtering was then performed with individual outliers removed if they were ±3 standard deviations (SDs) from the mean heterozygosity across all individuals using only autosomes (Supplementary Methods 6).

Sex check

A check to remove potential errors in sample identification was performed by identifying discrepancies between registered sex and imputed sex, including the following steps: (1) ensuring the pseudo-autosomal region is coded as a separate XY chromosome; (2) check registered sex against those imputed from X chromosome genotype calls; (3) identify females with inbreeding coefficient (F)>0.2 and males with F<0.8; and (4) remove individuals with discordant sex—females with F>0.5 and males with F<0.5.

Duplicate QC

Duplicate QC included the following steps: (1) confirming expected duplicates (from phenotype information) are true duplicates, based on genetic data using identity-by-descent (IBD) analysis with an estimated proportion of the genome shared IBD (PI_HAT)>0.98; (2) removing all discordant non-missing SNPs, based on SNP concordance analysis in true duplicates; and (3) removing the individual with the lowest call rate from each true duplicate pair. Expected duplicates not confirmed in the IBD analysis were resolved in the relatedness check.

Relatedness checks

A pedigree build and relatedness check was performed using KING version 2.2.5[75] (Supplementary Methods 7), whereby genetic data was used to confirm reported and infer unreported relationships. Any identified within-family errors or between-family issues were

investigated. Plausible relationships inferred from the genetic data were updated in the pedigree. Meanwhile, individuals or families were removed if the reported or inferred relationship was erroneous or implausible (for example, unexpected duplicates). An in-depth description of how each relationship type was confirmed is detailed below.

Inferred monozygotic twin pairs were confirmed based on whether they had the same registered sex and year of birth. Furthermore, the relationships of identified monozygotic twin pairs with other family members were also examined to ensure they were as expected. When a pair of individuals were inferred as a duplicate/monozygotic twin pair, and reported information indicated the pair were unexpected duplicates (based on reported information it was impossible for the pair to be an monozygotic twin pairs, i.e., different registered sex, year of birth, parents), the inferred family relationships were used to identify the individual to exclude from the study. Within-generation parent-offspring relationships and across-generation full-siblings or half-siblings relationships were confirmed together. The within-generation parent-offspring relationship was initially confirmed based on an age difference of at least 15 years, followed by confirmation that all other family relationships were as expected, including the across-generation full-siblings or half-siblings relationship. If the relationships could not be confirmed, unexpected family relationships were used to identify the individual(s) to remove. Full-siblings in the parent generation were incorporated if all relationships between the families were as expected. Full-siblings and half-siblings in the child generation were incorporated if the reported parent-offspring relationships were as expected and one or both parents were unreported. Inferred parent-offspring relationships where the parent was not reported were confirmed if all other expected relationships were as expected. When parent-offspring errors occurred where the expected mother was unrelated to the child, the family was removed as blood was taken from the mother and child at the same time. Meanwhile, for parent-offspring errors where an expected father was unrelated to the child, the parent-offspring relationship was broken and both individuals were kept in the family. The individuals were not removed or placed into separate families, because it is plausible (in contrast to unrelated mothers, described above) that the individual from whom the blood sample was collected as the father was not the biological father of the child but is acting as parental figure for the child, therefore, it is important to keep the individual in the dataset for gene-environment studies.

IBD analysis was used to confirm the relationships inferred by KING. Unrelated individuals who were not members of a family and had an estimated PI_HAT>0.15 were removed (approximately corresponding to the lower threshold for coding second-degree relatives or the upper threshold for coding third-degree relatives in KING).

An excess cryptic relatedness check was performed by, (1) computing a sum of kinship coefficients>2.5%; (2) plotting the summed kinship for each individual; and (3) removing outliers based on visual inspection.

After within-family and between-family relationships were confirmed by genetic data, a check for ME was performed, including families with one or two parents present in the data. Families with >5% errors and SNPs with >1% errors were removed. The remaining ME were set to missing.

Ancestry and plate effect checks

Within the core ancestry populations identified in module 1, additional ancestry outliers were removed based on PCA with the 1000 Genomes Project phase 1 unrelated data[100]. As described earlier, PCs were first estimated within founders only, after LD pruning (including removing SNPs in long range high LD regions[118] and merging with the 1000 Genomes Project dataset. The non-founders were then projected into the PC space of the founders (Supplementary Methods 4). Ancestry outliers were removed based on visual inspection, using pairwise plots for the first seven PCs.

Similarly, PCA was used to identify substructure within each ancestry population of all MoBa batches. PCs were first estimated in founders only, after LD pruning (including removing SNPs in long range high LD regions[118]). The non-founders were projected into the PC space of the founders. Outliers were removed based on visual inspection, using pairwise plots for the first ten PCs.

Plate effect checks were performed, whereby: (1) the variation in the first four PCs were investigated by creating box and whisker plots grouped by genotyping plate; (2) scatter plots were created for PC1 vs PC2 and PC3 vs PC4 coloured by genotyping plate to ensure clustering of plates did not occur; (3) Analysis of Variance (ANOVA) was performed to determine if there was a difference between genotyping plates within the first 10 PCs; and (4) the Cochran-Mantel-Haenszel test, in founders only, was used to assess the association of SNPs with genotyping plates (using the mh2 function with registered sex as the phenotype). SNPs associated with genotyping plates at a p-value<0.0001 were removed.

Autosomal chromosomes

The QC was restricted to autosomal chromosomes at the beginning of the third round of the ancestry population QC (module 2). Therefore, a sex check was not performed in the remainder of the autosome pipeline.

Chromosome X and pseudoautosomal regions (PAR)

QC, phasing, imputation, and post-imputation QC of chromosome X and PAR SNPs was performed separately from the autosomes. First, individuals were restricted to those passing module 9 post-imputation QC of the autosomal chromosomes. Then, the sex chromosome data was separated by chromosome (X, Y, and PAR) and by registered sex for chromosome X and Y.

Basic QC was performed where SNPs and individuals were removed based on the following filters: (1) SNPs with MAF < 0.5% (within founders only to account for relatedness); (2) SNPs with call rate < 95%; (3) SNPs with call rate < 98% (Supplementary Methods 5); (4) SNPs and individuals with a call rate < 98%; and (5) chromosome X SNPs in females only and PAR SNPs out of HWE at $p < 1.00\times10^{-6}$ (within founders only to account for relatedness). A heterozygosity check was performed in PAR SNPs only. As heterozygosity was already checked in the autosomal data, individuals were only removed if the distribution is non-normal, given the small size of PAR.

Sex checks were then performed by first limiting to chromosome X and Y SNPs passing basic QC in both males and females, plus the PAR SNPs. Then a sex check was performed using (1) the chromosome X only and (2) chromosome Y only. First, the sex assignments

reported at birth were checked against those imputed from X chromosome genotype calls and individuals with discordant sex were removed (females F > 0.2 and males < 0.8). Then the biological sex assignments were checked against the number of non-missing Y chromosome genotype calls.

A ME check was then performed in families with one or two parents present in the data. Families with > 5% errors and SNPs with > 1% errors were removed. The remaining ME were set to missing.

Finally, a Cochran-Mantel-Haenszel test, in founders only, was used to assess the association of SNPs with genotyping plates (using the PLINK mh2 function with shuffled biological sex as the phenotype). SNPs associated with genotyping plates at a p-value<0.0001 were removed.

Module 3: Merge by genotyping array

Genotyping batches were merged by genotyping array (or genotyping arrays with significant overlap). Only SNPs passing three rounds of ancestry population QC in all the batches genotyped on the same array were kept. As multiple families were merged in each of the genotyping batches, the pedigree was updated to ensure the across-batch reported relationships were accurately coded in the merged datasets.

Chromosome X and PAR

Genotyping batches were merged by genotyping array (or genotyping arrays with significant overlap). Only SNPs passing the ancestry population QC in all batches genotyped on the same array were kept.

Module 4: QC of ancestry populations in merged genotyping array datasets

Ancestry population QC was carried out in each merged genotyping dataset (release1-HCE, release1-OMNI, release1-GSA, relsease2, release3, release4). The duplicate QC was performed as previously described, except both individuals from a true across-batch duplicate pair were kept, allowing this check to be performed in the post-imputation QC. All PCA were run using FlashPCA2.0[76] to accommodate the increased number of individuals, with relatedness addressed as previously described. Whereby, PCs were first estimated in founders only, after LD pruning (including removing SNPs in long range high LD regions[118]). The non-founders were then projected into the PC space of the founders. The plate effect check was replaced with a batch effect check, whereby: (1) the variation in the first four PC was investigated by creating box and whisker plots grouped by genotyping batch; (2) scatter plots were created for PC1 vs PC2 and PC3 vs PC4 coloured by genotyping batch to ensure clustering of batches did not occur; (3) ANOVA was performed to determine if there was a difference in the first 10 PCs between genotyping batches; and (4) the Cochran-Mantel-Haenszel test, in founders only, was used to assess the association of SNPs with genotyping batches. SNPs associated with genotyping batches at a p-value<0.0001 were removed (Supplementary Methods 8).

Chromosome X and PAR

The ancestry population QC was then carried out in each merged genotyping dataset as described in module 2. The plate effect check was replaced with a batch effect check, whereby the Cochran-Mantel-Haenszel test, in founders only, was used to assess the association of SNPs with genotyping batches. SNPs associated with genotyping batches at a p-value<0.0001 were removed.

Module 5: Pre-phasing QC

A pre-phasing check was performed using the publicly available (access controlled) European Genome-Phenome Archive (Study ID EGAS00001001710) Haplotype Reference Consortium (HRC) release 1.1[120] Imputation preparation and checking script version 4.2.13[77], whereby, SNPs were removed if they were: (1) duplicates; (2) indels; (3) strand ambiguous (A/T and C/G); (4) not present among HRC sites; (5) >0.2 MAF differences between the merged array MoBa dataset (within founders only to account for relatedness) and HRC; and (6) alleles different to HRC. To match the data present in HRC, where needed, the following additional steps were implemented: (1) SNP IDs were updated to match the SNP IDs in HRC; (2) SNP alleles were flipped to match the strand of HRC; and (3) allele 1 / allele 2 assignments were changed to match the assignment in HRC.

Module 6 & 7: Phasing and imputation

The publicly available (access controlled) European Genome-Phenome Archive HRC release 1.1[120] data was used as the reference panel for both phasing and imputation. Whole-chromosome phasing[121] was performed using SHAPEIT2 release 904[78] software with the duoHMM[79] algorithm to incorporate the pedigree information into the haplotype estimates. Imputation was performed using IMPUTE4.1.2_r300.3[80] (Supplementary Methods 9), an implementation of IMPUTE2[83] re-coded to run faster with more memory efficiency, in chunks of up to 5,000 individuals (with all individuals from the same family kept in the same chunk). Imputation was conducted in five megabase chunks, with one megabase buffer on each side (meaning three megabases from each chunk were used for analyses). Chunks were defined from the first SNP passing pre-phasing QC on each chromosome. Imputation quality scores (INFO) were calculated by QCTOOL version 2.0.8[81]. To evaluate the quality of imputation we generated figures showing dependency of INFO scores on MAF (Supplementary Figures 7-9) for all SNPs. Initial, post-imputation checks were performed following the steps outlined in the ic tool version 1.0.8[82].

Chromosome X and PAR

The PAR (PAR1 and PAR2) were phased and imputed following the same protocols as those used for the autosomes. For the non-PAR of the X chromosome, phasing was performed without application of the duoHMM algorithm, and imputation was carried out using IMPUTE2.3.2[83]. INFO scores for this region were calculated using QCTOOL version 2.2.0. The rest of the processes mirrored the procedures applied to the autosomes.

Module 8: Post-imputation QC

Post-imputation QC was performed in a single round following the QC by genotype array protocol for each imputation batch. Initially, dosage data was converted to best-guess, hard-call genotype data using a certainty threshold 0.7 (Supplementary Methods 10). SNP and individual QC were then performed. The following thresholds were used for SNP removal: (1) INFO≤0.8; (2) MAF<1% (within founders only to account for relatedness); (3) call rate<95% (Supplementary Methods 11); (4) HWE p-value<1.00×10^{-6} (within founders only to account for relatedness); (5) discordant (concordance rate<97%) in true duplicates (expected duplicates confirmed by genetic data PI_HAT>0.95); (6) >1% ME (with remaining ME set to missing); and (7) association with genotype batch at a p-value<5×10^{-8}. The following thresholds were used for individual removal: (1) call rate<98%; (2) ±3 SDs from the mean heterozygosity across all individuals; (3) the individual from each true duplicate pair with the lowest call rate (Supplementary Methods 12); (4) all relatedness checks described in module 2 (Supplementary Methods 13); (5) cryptic relatedness; (6) ME>5% in families; (7) ancestry outliers (PCA using imputed SNPs of MoBa & 1000 Genomes Project[100]); and (8) ancestry population outliers (PCA using imputed SNPs of MoBa). Additionally, assessment of the number of ME within full trios was conducted with respect to INFO and MAF (Supplementary Methods 14).

Chromosome X and PAR

Post-imputation QC was performed in a single round following the QC by genotype array protocol for each imputation batch. Initially, the X and PAR dosage data was converted to best-guess, hard-call genotype data using a certainty threshold 0.7 (Supplementary Methods 10). The chromosome X data was then separated by registered sex. Then the following thresholds were used for SNP removal: (1) SNPs with MAF <1% (within founders only to account for relatedness); (2) call rate <95% (Supplementary Methods 11); (3) HWE at p-value<1.00×10^{-6} (within founders only to account for relatedness) in PAR and females only for chromosome X; (4) >1% ME (with remaining ME set to missing); and (5) association with imputation batch at a p-value<5×10^{-8}. The following thresholds were used for individual removal: (1) call rate <98%; (2) non-normal heterozygosity distribution of PAR; (3) ME>5% in families; and (4) discordant sex (females F>0.5 and males F<0.5).

Module 9: Post-imputation QC of merged imputation batches

Post-imputation QC of the merged imputation batches is required for cohorts with multiple imputation batches to confirm expected cross-batch relationships and ensure inferred across-imputation relationships are accurately coded in extended families (Supplementary Methods 15). The imputation batches were merged keeping only overlapping SNPs passing post-imputation QC in all batches. The pedigree was updated to ensure the across-imputation reported relationships were accurately coded in the merged dataset. The post-imputation SNP and individual QC procedures, including relatedness checks of updated across-imputation batch relationships, were performed as described in module 8. The batch effect check was replaced with an imputation effect check, whereby: (1) the variation in the first four PCs were investigated by creating box and whisker plots grouped by imputation batch; (2) scatter plots were created for PC1 vs PC2 and PC3 vs PC4 coloured by imputation batch to ensure clustering of imputation batches did not occur; (3) ANOVA was performed to

determine if there was a difference in the first 10 PCs between imputation batches; and (4) the Cochran-Mantel-Haenszel test, in founders only, was used to assess the association of SNPs with imputation batches. SNPs associated with imputation batches at a p-value<5×10^{-8} were removed.

Chromosome X and PAR

The imputation batches were merged keeping only overlapping SNPs passing post-imputation QC in all imputation batches. The post-imputation SNP and individual QC procedures were then performed as described in module 8. The batch effect check was replaced with an imputation effect check, whereby the Cochran-Mantel-Haenszel test, in founders only, was used to assess the association of SNPs with imputation batches. SNPs associated with imputation batches at a p-value<5×10^{-8} were removed.

Mitochondrial DNA haplotype estimation

QC

QC and imputation of mitochondrial SNPs was performed separately from the autosomes and sex chromosomes. OMNI array does not contain probes tagging mitochondrial SNPs, therefore, the four batches (NORMENT-Jan-15, NORMENT-Jun-15, NORMENT-May-16 and ADHD1) that were genotyped using OMNI arrays were not processed. Additionally, we excluded three batches (NORMENT-Feb-18, ADHD2 and NORMENT-Feb-20-V1) genotyped on GSA v.1.0 array due to very sparse coverage of mitochondrial genome, with less than 80 genotyped mitochondrial SNPs available after processing of raw intensity (.idat) files. After these exclusions we ended up with 19 batches which underwent pre-imputation QC and imputation, one batch at a time.

Pre-imputation QC was done using PLINK v1.90b7.2 (11 Dec 2023)[115]. For the three batches (HARVEST12good, HARVEST12bad, HARVEST24) genotyped using the HCE array, alleles were converted from Illumina A/B alleles to the genetic A, C, G, T alleles on the plus strand. Only SNPs with A/C/G/T alleles were retained for further processing. We further removed SNPs with duplicated position and alleles (keeping the first occurrence for each group of duplicates), as well as monomorphic SNPs and variants with genotype missingness >99%. Individuals with genotype missingness >99% were excluded.

Imputation

For imputation of mitochondrial SNPs we deployed MitoImpute pipeline[85,86]. MitoImpute provides several versions of imputation reference panels. We used the MitoImpute reference panel restricted to SNPs with minor allele frequency above 0.1%, containing 1,874 SNPs. Following MitoImpute guidelines, we ensured that chromosomes and positions are given according to the revised Cambridge Reference Sequence (rCRS)[122], sex of all samples was changed to male and PLINK was used to convert genotype data into Oxford-format. IMPUTE2 v.2.3.2 was then applied with "-chrX -iter 2 -burnin 1 -k_hap 500" arguments. Treating all samples as males and applying "-chrX" argument instructs IMPUTE2 software[83] to use an imputation model adjusted for haploid genomes. The "-k_hap" argument controls the number of haplotypes from the reference panel selected by IMPUTE2 to be used as templates when imputing missing genotypes. The "-iter" argument defines the total number of Markov chain Monte Carlo interations to be performed and the "-burnin" argument specifies

how many of these iterations at start will not contribute to the final imputation probabilities. We use IMPUTE2 argument values recommended by the MitoImpute protocol[85].

Chromosome Y haplotype estimation

QC

QC of the chromosome Y data followed similar procedures as those used for other chromosomes up until module 5. Before imputation, duplicate SNPs, indels, non-A/C/G/T alleles, and ambiguous SNPs were excluded. The dataset was then filtered to include only male samples, determined based on the count of variants on the Y chromosome. Additional filtering removed variants with more than 2% missingness and monomorphic variants.

The genomic coordinates were aligned with the hg38 reference genome using LiftOver[73,74] to ensure compatibility with the Genome Aggregation Database (gnomAD) v3.1.2 reference panel[123]. To maintain consistency with gnomAD, several modifications were made: SNP IDs were revised to match those in gnomAD, SNP alleles were flipped to align with the gnomAD strand orientation, and the designations of Allele 1 and Allele 2 were adjusted to match gnomAD assignments.

Imputation

Since both the Y chromosome and mitochondrial DNA are non-recombining, a similar imputation procedure was applied to the Y chromosome using IMPUTE2 v.2.3.2[83] with the arguments "-chrX -iter 2 -burnin 1 -k_hap 500." Due to the low density of genotyped markers, only the first 26 megabase of the Y chromosome could be imputed. To optimally use computational resources during imputation, 5 megabase chunks with a 1 megabase buffer on each side were created, each accommodating up to 5,000 individuals. After merging the chunks, INFO scores were calculated using QCTOOL version 2.2.0[81].

Following imputation, the genomic coordinates were lifted back to the hg19 reference genome before proceeding with haplogroup estimation. Haplogroups were then estimated using yHaplo[84], which employs the ISOGG Y-DNA tree 2016 version (https://isogg.org/tree/2016/index16.html) with default settings, based on genotyped and imputed variants with INFO scores greater than 0.8.

Supplementary Methods 3 – Identifying ancestry by clustering

To quantify the potential difference in ancestry group assignment between visual and automated clustering we used the ancestry inference implemented in the following script: https://github.com/MRCIEU/ukb-rap-utils/blob/main/scripts/ancestry/king_ancestry.r. This approach performs supervised clustering, whereby 1000 Genomes Project phase 3[100] data was used as a reference to identify individuals clustering with the reference superpopulations. Results from this analysis showed considerable overlap, with 98.5% identified as clustering with the European superpopulation in both the automated and visual clustering (Supplementary Figure 4).

Supplementary Methods 4 – PCA projection methodology

Using PLINK1.9 we conducted a comparison of the PCs estimated in (1) 1000 Genomes Project with MoBa projected into the PC space (2) 1000 Genomes Project plus MoBa founders with MoBa non-founders projected into the PC space shows. The first five PCs were highly correlated; in the NORMENT_Nov2020_1146 batch, the Pearson correlations for PC1, PC2, PC3, PC4, and PC5 were 0.9998, -0.9994, 0.9944, 0.9854, and 0.9301, respectively.

Recent methodological advancements in PCA projection[124,125], including the development of relatively computationally efficient software packages, have enabled improved identification and adjustment of fine-scale population structure in homogenous samples. In particular, the online augmentation, decomposition and Procrustes transformation (OADP) is a projection method, which is relatively computationally efficient[124]. In the UK Biobank, lower scaled mean-squared differences (MSD) were reported compared to simple projection (SP; as implemented in PLINK1.9 and FlashPCA2.0) in European populations (OADP MSD=0.100 vs SP MSD=0.360), while negligible differences were reported for diverse populations (OADP MSD=0.156 vs SP MSD=0.156)[124].

Unfortunately, the relatively computationally efficient OADP software was not available until after the MoBaPsychGen QC protocol was established, finalized, and QC analyses had commenced. Therefore, to maintain methodological consistency across all genotyping batches, particularly considering the estimated computational runtimes were not negligible we decided to consistently apply the SP method throughout the MoBaPsychGen protocol. Given the continuous improvement of methodology, software efficiency, and imputation reference panels, we anticipate that the protocol will be updated in the future to incorporate these advancements, however, it is outside the scope of the current project.

Supplementary Methods 5 – Call rate filtering

When PLNK is run including a call rate filter for both SNPs (--geno) and individuals (--mind) in the same command the individuals filtering is performed first. To try to keep as many samples as possible, the SNP call rate filtering with a threshold of 98% was performed twice, firstly in a PLINK command alone to remove SNPs with low call rate, and secondly, in a PLINK command with the individual call rate filter; with the second command initially removing and individuals with a low call rate before ensuring no SNPs have a low call rate after individuals with high missingness have been removed.

Supplementary Methods 6 – Heterozygosity outlier filtering

Heterozygosity filtering was performed using a threshold of ±3 SDs from the mean heterozygosity across all individuals rather than an F statistic to ensure the heterozygosity distribution was normally distributed in all genotyping batches. In large genotyping batches the F statistic plots were normally distributed, however, in small genotyping batches large tails were observed (Supplementary Figure 5), however, limited or no outliers were removed. Therefore, filtering using ±3 SDs from the mean heterozygosity across all individuals was used to ensure heterozygosity outliers were removed.

Supplementary Methods 7 – Pedigree build and relatedness check software

In the MoBaPsychGen pipeline, we used a similar strategy to the UK Biobank[15] to investigate the relatedness structure within MoBa. This involved first checking and inferring relationships using KING (kinship coefficient and proportion of SNPs with zero identical-by-state; IBS0 estimates) before confirming the relationships using the PLINK genome function (estimates of PI_HAT and probability of 0, 1, and 2 alleles are shared IBD; Z0, Z1, and Z2, respectively) to ensure the pairwise relationships were as expected for the types of relationships in MoBa (including distinguishing full-siblings from parent-offspring pairs). KING was used to perform the initial pedigree build and relatedness checks instead of PLINK because (1) in the presence of admixture KING can accurately infer zeroth-degree (monozygotic twin or duplicate pairs; kinship coefficient >0.3540), first-degree (parent-offspring, full sibling, dizygotic twin pairs; kinship coefficient range 0.1770-0.3540), second-degree (half-siblings, grandparent-grandchild, avuncular pairs; kinship coefficient range 0.0884–0.1770), and third-degree (first cousins; kinship coefficient range 0.0442–0.0884) relationships; and (2) preforms a pedigree build and outputs files to easily update family and parental IDs, whereas using PLINK the pedigree build would need to be manually curated and would likely introduce errors in such a large cohort.

This is the same strategy used to investigate the relatedness structure within UK Biobank. An additional advantage for MoBa was that we were able to construct an initial pedigree of expected parent-offspring relationships within MoBa. As such the main goal of the relatedness checks were to confirm reported relationships before infer unreported relationships (primarily in the parent generation).

PC-relate[126] is a newer method than that improves on KING for admixed and diverse cohorts. Given this is not the case in MoBa, with the vast majority of individuals in MoBa clustering with the 1000 Genomes Project European superpopulation, concern for population structure bias in kinship estimates is minimal.

Supplementary Methods 8 – Batch effect checks

The two ADHD batches contained child cases and their parents, plus matched control children. Furthermore, seven batches were genotyped as full (mother-father-child) trios. Therefore, a concern of the batch effect check is that some variants associated with ADHD or trio-ness could be removed via selection biases. However, the batch effect check was performed in founders only, and the number of SNPs removed due to association with batch was small (0.10-0.22% of SNPs removed). Furthermore, looking at the PC1 vs PC2 and PC3 vs PC4 plots colored by batch the SNP removal is driven by large variation in sample size rather than clustering of specific batches (Supplementary Figure 6).

Supplementary Methods 9 – Imputation software

IMPUTE4[80] was used for imputation as it uses the methodology implemented in IMPUTE2[83], which can handle both related and unrelated individuals, with more computational efficiency, as it was specifically designed for the imputation of large datasets. IMPUTE5[127] was not

considered as we were unsure whether the software can appropriately account for varying levels of relatedness during imputation.

Supplementary Methods 10 – Genotype certainty threshold

The certainty threshold of 0.7 was used to balance the high missingness that results from using the PLINK default of 0.9 and with the level of uncertainty that is added to the dataset by lowering the threshold.

Supplementary Methods 11 – Post-imputation QC SNP call rate filter

The post-imputation QC SNP call rate filter was reduced to 95% as researchers may have specific reasons for investigating SNPs with slightly lower SNP call rate threshold. Therefore, we have applied a lower SNP call rate threshold of 95% during the post-imputation QC. If researchers require the higher SNP call rate threshold of 98%, they can easily apply this to the data using plink.

Supplementary Methods 12 – Post-imputation QC duplicate removal

Genotyping batch was not used as the deciding factor to identify which individual from each duplicate pair to remove, because the varying selection criteria of genotyping batches prevented all nuclear and blended family members being genotyped in the same genotyping batch. Meaning it is not possible to preserve the integrity of genotyping array within families. Therefore, we chose to use call rate as the deciding factor to identify the duplicate to remove to prevent introducing biases on which family members to have genotyped using the same array.

Supplementary Methods 13 – Filtering imputed SNPs for KING analyses

For the KING relatedness checks, temporary SNP removal was required due to computational resource limitations: SNPs with call rate<98%, MAF<5% (within founders only to account for relatedness), and pairs of SNPs with squared correlation greater than 0.4 were temporarily removed.

KING website (https://www.kingrelatedness.com/manual.shtml) recommends: "Please do not prune or filter any good SNPs that pass QC prior to any KING inference, unless the number of variants is too many to fit the computer memory, in which case rare variants can be filtered out. LD pruning is not recommended in KING". However, this is referring to directly genotyped SNPs. To get enough SNP filtering, a really high MAF threshold had to be used (>30% within founders only to account for relatedness). Therefore, given the SNPs were imputed, light LD pruning was used to ensure that the SNPs were distributed across the genome, rather than INFO score filtering, which is correlated with MAF, and would have led

to an uneven distribution of SNPs across the genome (with SNPs in high LD with directly genotyped SNPs likely to have higher INFO scores).

Supplementary Methods 14 – Assessment of ME within full trios

Within each imputation batch, we used full trios (release1-HCE=6,748, release1-OMNI=5,033, release1-GSA=7,764, release2=4,124, release3=1,609, release4=230) to assess the QC by stratifying the number of ME by INFO score and MAF in the SNPs passing post-imputation QC. As seen in Supplementary Figure 10 for SNPs passing post-imputation QC the pattern of ME is similar across all INFO and MAF thresholds within each imputation batch, proportional to the number of full trios within the batch. The HCE imputation batch exhibits slightly elevated numbers, as expected due to the lower number of common genotyped SNPs.

Supplementary Methods 15 – Merge imputation batches

MoBa was genotyped through multiple projects with varying selection criteria. As a result, the reported nuclear and blended families were not genotyped as complete families. Therefore, to ensure whole families can be analysed together and MoBa can be analysed as a complete cohort, the imputation batches were merged. Merging only overlapping SNPs passing post-imputation QC in all imputation batches ensures a single distribution of power and non-centrality parameter across all SNPs. Furthermore, a central assumption of standard meta-analysis approaches is that effect sizes are independent. Methodologies are being developed to conduct meta-analyses of results with non-independent effect estimates, however, there are significant limitations to these methods[128]. Furthermore, we have described below the considerations we made that resulted in six imputation batches, that necessitated the merging of imputation batches.

Given MoBa is a family cohort, we decided that the protocol needed to place importance on the impact to haplotype estimation in addition to imputation quality and bias, plus performance of polygenic scores. During the QC process we discussed with Vivek Appadurai the best approach to limit phasing and imputation errors and biases in the MoBa genotyping data. His recent paper preforming benchmarking work, that investigates both phasing, imputation, and polygenic score performance, based on four data integration protocols for on how phasing and imputation were performed in batches genotyped with different arrays[129]. The four data integration protocols evaluated were: (1) “separate”, where the batches were phased and imputed separately; (2) “intersection”, where batches are merged and phasing and imputation are performed together based only on SNPs genotyped in both batches; (3) “union” where batches are merged and phasing and imputation are performed together based SNPs genotyped on either batches; and (4) “two stage” where phasing, and imputation are performed separately by batch before merging the “union” SNPs and performing phasing and imputing of the merged dataset (similar to the approach for combining datasets with varying technology, i.e., array-based genotyping and whole genome sequencing[130]). The benchmarking results showed that due to the specific genotyping data available (e.g., multiple genotyping batches, low SNP overlap between genotyping batches), trade-off situations arise whereby educated decisions need to be made. We strongly believe that is the case in MoBa.

This work replicates previous work showing that taking an intersection approach when incorporating samples genotyped on multiple arrays leads to a loss of imputation accuracy compared to the separate imputation approach[131]. Furthermore, the new benchmarking work showed that taking an intersection approach, with two arrays overlapping at approximately 180,000 markers, leads to increased Switch Error Rates (and attenuation in variance explained from polygenic score analyses) compared to the separate approach.

Combined, we made the educated decision that for MoBa the trade-off of performing separate phasing and imputation was important due to the reduction of Switch Error Rates. However, while establishing the MoBaPsychGen QC protocol, we determined the SNP overlaps of 252,110 for HCE-OMNI pair, 105,360 for HCE-GSA pair, and 137,341 for OMNI-GSA pair. We further determined that after pre-imputation QC, there were less than 200,000 overlapping SNPs when the three batches genotyped with HCE and four batches genotyped with an OMNI were merged. This low intersection was determined to impact the quality of imputation, potentially deteriorating performance of polygenic risk scores. Therefore, we performed phasing and imputation separately for the HCE and OMNI batches. Meanwhile, the batches genotyped using the GSA array were not phased and imputed in a single imputation batch because the genotyping batches were received at varying timepoints after imputation and post-imputation QC of other GSA batches had already been performed.

At this point, we determined that considerable investment of time and resources would be required to re-impute all the GSA batches together or perform a second round of imputation using well imputed SNPs from all imputation batches for minor improvement in imputation accuracy. Further to this, we have performed analyses to see the amount of inflation potentially introduced into genome-wide association analyses by performing separate phasing and imputation and determined that adjusting for genotyping batch negates this bias.

These analyses performing 12 genome-wide association analyses (adjusted for age, registered sex, and the first 20 PCs) to test for association with imputation batch. This follows the method previously described for identifying batch effects[69], with the modification of assigning an additional 10% of cases randomly selected from the control imputation batches, to allow for testing as to whether adjusting for genotyping batch negates any induced bias (Supplementary Figure 11). The SNP-based heritability estimates for the analyses without adjustment were: -0.0876 (SE=0.0087, intercept=1.8539) for release1-GSA, -0.4856 (SE=0.0182, intercept=2.0027) for release1-HCE, -0.1911 (SE=0.0078, intercept=1.1251) for release1-OMNI, -0.0225 (SE=0.0033, intercept=1.2358) for release2, -0.0284 (SE=0.0044, intercept=1.2262) for release3, and -0.0428 (SE=0.0105, intercept=1.1826) for release4. Meanwhile, the SNP-based heritability estimates for the analyses with adjustment for genotyping batch were: 0.0037 (SE=0.0037, intercept=0.999) for release1-GSA, 0.0046 (SE=0.0039, intercept=0.9997) for release1-HCE, -0.0022 (0.0048, intercept=1.0031) for release1-OMNI, -0.0002 (SE=0.0026, intercept=1.0006) for release2, -0.0016 (SE=0.0031, intercept=1.007) for release3, and -0.0028 (SE=0.0073, intercept=1.002) for release4.

Furthermore, we performed simulation analyses which show that adjusting for genotyping batch does not induce collider bias (https://explodecomputer.github.io/lab-book/posts/2024-03-27-collider-gwas/).

Supplementary Text References

2 Howe, L. J. *et al.* Within-sibship genome-wide association analyses decrease bias in estimates of direct genetic effects. *Nature Genetics* (2022). https://doi.org/10.1038/s41588-022-01062-7

12 Ziyatdinov, A. *et al.* Genotyping, sequencing and analysis of 140,000 adults from Mexico City. *Nature* **622**, 784-793 (2023). https://doi.org/10.1038/s41586-023-06595-3

14 Kurki, M. I. *et al.* FinnGen provides genetic insights from a well-phenotyped isolated population. *Nature* **613**, 508-518 (2023). https://doi.org/10.1038/s41586-022-05473-8

15 Bycroft, C. *et al.* The UK Biobank resource with deep phenotyping and genomic data. *Nature* **562**, 203-209 (2018). https://doi.org/10.1038/s41586-018-0579-z

36 Eilertsen, E. M. *et al.* Direct and indirect effects of maternal, paternal, and offspring genotypes: Trio-GCTA. *Behavior Genetics* **51**, 154-161 (2021). https://doi.org/10.1007/s10519-020-10036-6

40 Torvik, F. A. *et al.* Modeling assortative mating and genetic similarities between partners, siblings, and in-laws. *Nature Communications* **13**, 1108 (2022). https://doi.org/10.1038/s41467-022-28774-y

66 Pedigree Imputation Consortium Pipeline (PICOPILI) (https://github.com/Nealelab/picopili, 2016).

67 Anderson, C. A. *et al.* Data quality control in genetic case-control association studies. *Nature Protocols* **5**, 1564-1573 (2010). https://doi.org/10.1038/nprot.2010.116

68 Lam, M. *et al.* RICOPILI: Rapid Imputation for COnsortias PIpeLIne. *Bioinformatics* **36**, 930-933 (2020). https://doi.org/10.1093/bioinformatics/btz633

69 Turner, S. *et al.* Quality control procedures for genome-wide association studies. *Current Protocols in Human Genetics* **68**, 1.19.11-11.19.18 (2011). https://doi.org/https://doi.org/10.1002/0471142905.hg0119s68

73 LiftOver (https://genome.sph.umich.edu/wiki/LiftOver#Lift_genome_positions).

74 Hinrichs, A. S. *et al.* The UCSC Genome Browser Database: update 2006. *Nucleic Acids Research* **34**, D590-D598 (2006). https://doi.org/10.1093/nar/gkj144

75 Manichaikul, A. *et al.* Robust relationship inference in genome-wide association studies. *Bioinformatics* **26**, 2867-2873 (2010). https://doi.org/10.1093/bioinformatics/btq559

76 Abraham, G., Qiu, Y. & Inouye, M. FlashPCA2: principal component analysis of Biobank-scale genotype datasets. *Bioinformatics* **33**, 2776-2778 (2017). https://doi.org/10.1093/bioinformatics/btx299

77 HRC or 1000G Imputation Preparation and Checking v. 4.2.13 (https://www.well.ox.ac.uk/~wrayner/tools/).

78 Delaneau, O., Marchini, J. & Zagury, J.-F. A linear complexity phasing method for thousands of genomes. *Nature Methods* **9**, 179 (2011). https://doi.org/10.1038/nmeth.1785

79 O'Connell, J. *et al.* A general approach for haplotype phasing across the full spectrum of relatedness. *PLOS Genetics* **10**, e1004234 (2014). https://doi.org/10.1371/journal.pgen.1004234

80 IMPUTE4 v. 1.2_r300.3 (https://jmarchini.org/software/#impute-4).

81 QCTOOL v. 2.08 (https://www.well.ox.ac.uk/~gav/qctool_v2/).

82 ic: A post-imputation data checking program v. 1.0.8 (https://www.well.ox.ac.uk/~wrayner/tools/Post-Imputation.html).

83 Howie, B. N., Donnelly, P. & Marchini, J. A flexible and accurate genotype imputation method for the next generation of genome-wide association studies. *PLOS Genetics* **5**, e1000529 (2009). https://doi.org/10.1371/journal.pgen.1000529

84 Poznik, G. D. Identifying Y-chromosome haplogroups in arbitrarily large samples of sequenced or genotyped men. *bioRxiv*, 088716 (2016). https://doi.org/10.1101/088716

85 McInerney, T. W. *et al.* A globally diverse reference alignment and panel for imputation of mitochondrial DNA variants. *BMC Bioinformatics* **22**, 417 (2021). https://doi.org/10.1186/s12859-021-04337-8
86 sjfandrews/MitoImpute: v0.2 v. v0.2 (Zenodo, 2020).
98 Kässens, J. C., Wienbrandt, L. & Ellinghaus, D. BIGwas: Single-command quality control and association testing for multi-cohort and biobank-scale GWAS/PheWAS data. *GigaScience* **10**, giab047 (2021). https://doi.org/10.1093/gigascience/giab047
99 Gibbs, R. A. *et al.* The International HapMap Project. *Nature* **426**, 789-796 (2003). https://doi.org/10.1038/nature02168
100 Auton, A. *et al.* A global reference for human genetic variation. *Nature* **526**, 68-74 (2015). https://doi.org/10.1038/nature15393
101 Wong, K. H. Y. *et al.* Towards a reference genome that captures global genetic diversity. *Nature Communications* **11**, 5482 (2020). https://doi.org/10.1038/s41467-020-19311-w
102 Peterson, R. E. *et al.* Genome-wide Association Studies in Ancestrally Diverse Populations: Opportunities, Methods, Pitfalls, and Recommendations. *Cell* **179**, 589-603 (2019). https://doi.org/10.1016/j.cell.2019.08.051
103 Conomos, Matthew P. *et al.* Genetic Diversity and Association Studies in US Hispanic/Latino Populations: Applications in the Hispanic Community Health Study/Study of Latinos. *The American Journal of Human Genetics* **98**, 165-184 (2016). https://doi.org/https://doi.org/10.1016/j.ajhg.2015.12.001
104 Jordan, E. *et al.* Genetic Architecture of Dilated Cardiomyopathy in Individuals of African and European Ancestry. *JAMA* **330**, 432-441 (2023). https://doi.org/10.1001/jama.2023.11970
105 Børte, S. *et al.* Mitochondrial genome-wide association study of migraine – the HUNT Study. *Cephalalgia : an international journal of headache* **40**, 625-634 (2020). https://doi.org/10.1177/0333102420906835
106 Dupuy, B. M., Stenersen, M., Lu, T. T. & Olaisen, B. Geographical heterogeneity of Y-chromosomal lineages in Norway. *Forensic Science International* **164**, 10-19 (2006). https://doi.org/https://doi.org/10.1016/j.forsciint.2005.11.009
107 Y Chromosome Consortium. A nomenclature system for the tree of human Y-chromosomal binary haplogroups. *Genome research* **12**, 339-348 (2002). https://doi.org/10.1101/gr.217602
108 Qiao, Z. *et al.* Introducing M-GCTA a software package to estimate maternal (or paternal) genetic effects on offspring phenotypes. *Behavior Genetics* **50**, 51-66 (2020). https://doi.org/10.1007/s10519-019-09969-4
109 Abecasis, G. R., Cardon, L. R. & Cookson, W. O. C. A General Test of Association for Quantitative Traits in Nuclear Families. *The American Journal of Human Genetics* **66**, 279-292 (2000). https://doi.org/https://doi.org/10.1086/302698
110 Gauderman, W. J. Candidate gene association analysis for a quantitative trait, using parent-offspring trios. *Genetic Epidemiology* **25**, 327-338 (2003). https://doi.org/https://doi.org/10.1002/gepi.10262
111 Wheeler, E. & Cordell, H. J. Quantitative trait association in parent offspring trios: Extension of case/pseudocontrol method and comparison of prospective and retrospective approaches. *Genetic Epidemiology* **31**, 813-833 (2007). https://doi.org/https://doi.org/10.1002/gepi.20243
112 Brumpton, B. *et al.* Avoiding dynastic, assortative mating, and population stratification biases in Mendelian randomization through within-family analyses. *Nature communications* **11**, 1-13 (2020).
113 Guan, J. *et al.* Family-based genome-wide association study designs for increased power and robustness. *Nature Genetics* **57**, 1044-1052 (2025). https://doi.org/10.1038/s41588-025-02118-0
114 Feeney, T., Hartwig, F. P. & Davies, N. M. How to use directed acyclic graphs: guide for clinical researchers. *BMJ* **388**, e078226 (2025). https://doi.org/10.1136/bmj-2023-078226

115 Purcell, S. *et al.* PLINK: A tool set for whole-genome association and population-based linkage analyses. *The American Journal of Human Genetics* **81**, 559-575 (2007). https://doi.org/10.1086/519795
116 Demontis, D. *et al.* Discovery of the first genome-wide significant risk loci for attention deficit/hyperactivity disorder. *Nature Genetics* **51**, 63-75 (2019). https://doi.org/10.1038/s41588-018-0269-7
117 Byun, J. *et al.* Ancestry inference using principal component analysis and spatial analysis: A distance-based analysis to account for population substructure. *BMC Genomics* **18**, 789 (2017). https://doi.org/10.1186/s12864-017-4166-8
118 Price, A. L. *et al.* Long-Range LD can confound genome scans in admixed populations. *The American Journal of Human Genetics* **83**, 132-135 (2008). https://doi.org/https://doi.org/10.1016/j.ajhg.2008.06.005
119 Coop, G. Genetic similarity versus genetic ancestry groups as sample descriptors in human genetics. *arXiv preprint arXiv:2207.11595* (2022).
120 McCarthy, S. *et al.* A reference panel of 64,976 haplotypes for genotype imputation. *Nature genetics* **48**, 1279-1283 (2016). https://doi.org/10.1038/ng.3643
121 Howie, B., Fuchsberger, C., Stephens, M., Marchini, J. & Abecasis, G. R. Fast and accurate genotype imputation in genome-wide association studies through pre-phasing. *Nature Genetics* **44**, 955 (2012). https://doi.org/10.1038/ng.2354
122 Andrews, R. M. *et al.* Reanalysis and revision of the Cambridge reference sequence for human mitochondrial DNA. *Nature Genetics* **23**, 147-147 (1999). https://doi.org/10.1038/13779
123 Chen, S. *et al.* A genomic mutational constraint map using variation in 76,156 human genomes. *Nature* **625**, 92-100 (2024). https://doi.org/10.1038/s41586-023-06045-0
124 Zhang, D., Dey, R. & Lee, S. Fast and robust ancestry prediction using principal component analysis. *Bioinformatics* **36**, 3439-3446 (2020). https://doi.org/10.1093/bioinformatics/btaa152
125 Privé, F., Luu, K., Blum, M. G. B., McGrath, J. J. & Vilhjálmsson, B. J. Efficient toolkit implementing best practices for principal component analysis of population genetic data. *Bioinformatics* **36**, 4449-4457 (2020). https://doi.org/10.1093/bioinformatics/btaa520
126 Conomos, Matthew P., Reiner, Alexander P., Weir, Bruce S. & Thornton, Timothy A. Model-free Estimation of Recent Genetic Relatedness. *The American Journal of Human Genetics* **98**, 127-148 (2016). https://doi.org/https://doi.org/10.1016/j.ajhg.2015.11.022
127 Rubinacci, S., Delaneau, O. & Marchini, J. Genotype imputation using the Positional Burrows Wheeler Transform. *PLOS Genetics* **16**, e1009049 (2020). https://doi.org/10.1371/journal.pgen.1009049
128 Cheung, M. W. L. A guide to conducting a meta-analysis with non-independent effect sizes. *Neuropsychology Review* **29**, 387-396 (2019). https://doi.org/10.1007/s11065-019-09415-6
129 Appadurai, V. *et al.* Accuracy of haplotype estimation and whole genome imputation affects complex trait analyses in complex biobanks. *Communications Biology* **6**, 101 (2023). https://doi.org/10.1038/s42003-023-04477-y
130 Mathur, R. *et al.* GAWMerge expands GWAS sample size and diversity by combining array-based genotyping and whole-genome sequencing. *Communications Biology* **5**, 806 (2022). https://doi.org/10.1038/s42003-022-03738-6
131 Johnson, E. O. *et al.* Imputation across genotyping arrays for genome-wide association studies: assessment of bias and a correction strategy. *Human Genetics* **132**, 509-522 (2013). https://doi.org/10.1007/s00439-013-1266-7
