# Supplementary material for: Family genetic designs in MoBa provide insights into health and functioning
Source: Nature. 2026 Aug 19;658(8136):707–15. doi: 10.1038/s41586-026-10926-5 (PMC13668695; doi:10.1038/s41586-026-10926-5)
Supplement: Supplementary file 2 [file 41586_2026_10926_MOESM2_ESM.pdf]

nature portfolio

Corresponding author(s): Alexandra Havdahl and Elizabeth Corfield

Last updated by author(s): Jul 24, 2026

Reporting Summary

Nature Portfolio wishes to improve the reproducibility of the work that we publish. This form provides structure for consistency and transparency in reporting. For further information on Nature Portfolio policies, see our Editorial Policies and the Editorial Policy Checklist.

Statistics

For all statistical analyses, confirm that the following items are present in the figure legend, table legend, main text, or Methods section.

n/a	Confirmed	
☐	☒	The exact sample size (*n*) for each experimental group/condition, given as a discrete number and unit of measurement
☐	☒	A statement on whether measurements were taken from distinct samples or whether the same sample was measured repeatedly
☐	☒	The statistical test(s) used AND whether they are one- or two-sided<br>*Only common tests should be described solely by name; describe more complex techniques in the Methods section.*
☐	☒	A description of all covariates tested
☐	☒	A description of any assumptions or corrections, such as tests of normality and adjustment for multiple comparisons
☐	☒	A full description of the statistical parameters including central tendency (e.g. means) or other basic estimates (e.g. regression coefficient) AND variation (e.g. standard deviation) or associated estimates of uncertainty (e.g. confidence intervals)
☐	☒	For null hypothesis testing, the test statistic (e.g. *F*, *t*, *r*) with confidence intervals, effect sizes, degrees of freedom and *P* value noted<br>*Give P values as exact values whenever suitable.*
☒	☐	For Bayesian analysis, information on the choice of priors and Markov chain Monte Carlo settings
☒	☐	For hierarchical and complex designs, identification of the appropriate level for tests and full reporting of outcomes
☒	☐	Estimates of effect sizes (e.g. Cohen's *d*, Pearson's *r*), indicating how they were calculated

Our web collection on statistics for biologists contains articles on many of the points above.

Software and code

Policy information about availability of computer code

Data collection	No software was used for data collection. Only secondary data analysis was performed.
Data analysis	All software used is described in the methods section and in in code, available on GitHub: https://github.com/psychgen/MoBaPsychGen-QC-pipeline & https://github.com/psychgen/moba-trio-analyses.<br>MoBaPsychGen pipeline:<br>The primary software used throughout the pipeline was PLINK v1.90b7.2 & v1.90b6.18 (https://www.cog-genomics.org/plink/) and The R Project for Statistical Computing (R 4.0.5; https://www.r-project.org/) was used to produce figures. Additionally, LiftOver tool (https://genome.ucsc.edu/store.html), KING version 2.2.5 (https://www.kingrelatedness.com/history.shtml#2.2.5), FlashPCA2.0 (https://github.com/gabraham/flashpca/releases/tag/v2.0), imputation preparation and checking script version 4.2.13 (https://www.chg.ox.ac.uk/~wrayner/tools/index.html), SHAPEIT2 release 904 with the duoHMM algorithm (https://mathgen.stats.ox.ac.uk/genetics_software/shapeit/shapeit.html), IMPUTE4.1.2_r300.3(https://jmarchini.org/software/#impute-4), QCTOOL version 2.0.8 & 2.2.0 (https://www.chg.ox.ac.uk/~gav/qctool/documentation/download.html), ic tool version 1.0.8 (https://www.chg.ox.ac.uk/~wrayner/tools/Post-Imputation.html), PLINK2 v2.00a2.3LM (https://www.cog-genomics.org/plink/2.0/), IMPUTE2.3.2 (https://mathgen.stats.ox.ac.uk/impute/impute_v2.html), yHaplo 2.1.12 (https://github.com/23andMe/yhaplo), and the MitoImpute pipeline v0.2 (https://github.com/sjfandrews/MitoImpute), BCFtools v 1.8 &1.9 (https://github.com/samtools/bcftools/), cat-bgen v1.1.4 (https://enkre.net/cgi-bin/code/bgen/doc/trunk/doc/wiki/cat-bgen.md), COSGAP v1.0.0 containers (gwas.sif, python3.sif, r.sif from https://github.com/comorment/containers/releases/tag/v1.0.0; for details see the documentation), python 3.8.10 (https://www.python.org/downloads/release/python-3810/), & perl v5.32.1 (https://perldoc.perl.org/perl5321delta).<br>Trio analyses:<br>The software used throughout the exemplar trio analyses were: phenotools R package 0.2.8 (https://doi.org/10.17605/OSF.IO/6G8BJ), R 4.1.0, (https://www.r-project.org/), linkage disequilibrium adjusted kinship (LDAK) v6 (https://dougspeed.com/), Linkage Disequilibrium Score

Regression89 (LDSC) version 1.0.1 (https://github.com/bulik/ldsc), LDpred2 (https://privefl.github.io/bigsnpr/articles/LDpred2.html) implemented in the bigsnpr package version 1.12.21 (https://github.com/privefl/bigsnpr), Trio-GCTA (https://github.com/espenmei/trio), and OpenMendel package (https://openmendel.github.io/).

For manuscripts utilizing custom algorithms or software that are central to the research but not yet described in published literature, software must be made available to editors and reviewers. We strongly encourage code deposition in a community repository (e.g. GitHub). See the Nature Portfolio guidelines for submitting code & software for further information.

Data

Policy information about availability of data

All manuscripts must include a data availability statement. This statement should provide the following information, where applicable:

- Accession codes, unique identifiers, or web links for publicly available datasets
- A description of any restrictions on data availability
- For clinical datasets or third party data, please ensure that the statement adheres to our policy

Data from the Norwegian Mother, Father and Child Cohort Study (MoBa) is managed by the Norwegian Institute of Public Health. The MoBa cohort is currently regulated by the Norwegian Health Registry Act. The data that support the findings of this study are available from the Norwegian Institute of Public Health, but restrictions apply to the availability of these data. The MoBa website provides details on how to obtain access for research and the available variables (https://www.fhi.no/en/ch/studies/moba/for-forskere-artikler/research-and-data-access/). Access to individual-level data from MoBa and national registries requires approval from the Regional Committees for Medical and Health Research Ethics (REK), compliance with General Data Protection Regulation (GDPR), and data holder approval. Researchers must apply via https://helsedata.no/en/. International researchers need to collaborate with a principal investigator employed at a Norwegian research institution - the MoBa administration team (mobaadm@fhi.no) can facilitate contact with relevant research environments. MoBa can be used for health research. The purpose of MoBa is to generate new knowledge about the causes, risk factors, courses, and consequences of diseases and health outcomes, with the ultimate goal of improving prevention and treatment. The MoBa administration normally uses 3–5 weeks to reach a decision from the time a complete application has been received. The expected processing time from decision is in place until data delivery is normally between 4 and 5 weeks. Delivery of linked data from health registries usually takes up to 8 weeks. Updated information about processing time is available on the MoBa website (https://www.fhi.no/en/ch/studies/moba/for-forskere-artikler/research-and-data-access/).

Information on how to access the MoBaPsychGen post-imputation quality-controlled data is available by contacting the MoBa administration and on the following website: https://www.fhi.no/en/more/research-centres/psychgen/access-to-genetic-data-after-quality-control-by-the-mobapsychgen-pipeline-v/. All summary statistics from genome-wide association studies (GWASs) conducted in this study will be available on the MoBa website for GWAS summary statistics: https://www.fhi.no/en/ch/studies/moba/for-forskere-artikler/gwas-data-from-moba/. Participant consent does not allow individual-level data storage in repositories or journals.

Additionally, the following external reference panels and LD resources were used in in the MoBaPsychGen pipeline and exemplar trio analyses. The publicly available (access controlled) European Genome-Phenome Archive (Study ID EGAS00001001710) Haplotype Reference Consortium (HRC) release 1.1 (https://ega-archive.org/studies/EGAS00001001710, https://ega-archive.org/access/request-data/how-to-request-data/). The Genome Aggregation Database (gnomAD) v3.1.2 (https://gnomad.broadinstitute.org/downloads). The MitoImpute reference panel restricted to SNPs with minor allele frequency above 0.1% (https://github.com/sjfandrews/MitoImpute/tree/master). The 1000 Genomes Project Phase 1 and Phase 3 (https://www.internationalgenome.org/data/, https://www.cog-genomics.org/plink/2.0/resources#phase3_1kg). Pre-computed linkage disequilibrium (LD) scores from the 1000 Genomes Project European data (https://data.broadinstitute.org/alkesgroup/LDSCORE/eur_w_ld_chr.tar.bz2). Pre-computed LD matrix from UK Biobank (https://figshare.com/articles/dataset/LD_reference_for_HapMap3_/21305061).

Research involving human participants, their data, or biological material

Policy information about studies with human participants or human data. See also policy information about sex, gender (identity/presentation), and sexual orientation and race, ethnicity and racism.

Reporting on sex and gender	Sex as registered in the national Medical Birth Registry of Norway.
Reporting on race, ethnicity, or other socially relevant groupings	The trio analyses are restricted to individuals of genetically inferred European ancestry. Population descriptives are available in the MoBa cohort profile papers (https://doi.org/10.1093/ije/dyl170 & https://doi.org/10.1093/ije/dyw029 & https://doi.org/10.1093/ije/dyaf139).
Population characteristics	MoBa is a population-based pregnancy cohort. The cohort includes approximately 114,500 children, 95,200 mothers and 75,200 fathers. The children were born between 1999 and 2009. Currently children are aged 17-27, while parents are 34-88 years old. More detailed information is provided in the MoBa Cohort profiles (https://doi.org/10.1093/ije/dyl170 & https://doi.org/10.1093/ije/dyw029 & https://doi.org/10.1093/ije/dyaf139).
Recruitment	The MoBa cohort recruited women and their partners at the universal ultrasound assessment in pregnancy.
Ethics oversight	The establishment of MoBa and initial data collection was based on a license granted from the Norwegian Data Protection Agency and an approval from The Regional Committees for Medical and Health Research Ethics (REK). The MoBa cohort is currently regulated by the Norwegian Health Registry Act. The current study was approved by The Regional Committees for Medical and Health Research Ethics (14140 and 2016/1226), and all research was performed in accordance with relevant guidelines and regulations. All mothers and fathers provided written informed consent at recruitment. Mothers consented to participation on behalf of themselves and their children. At age 18 years the children become independent participants and receive an information letter about their rights, including how to withdraw. Participation is voluntary and participants can

withdraw their consent at any time. In accordance with REK regulations, individuals who withdraw consent are excluded.

Note that full information on the approval of the study protocol must also be provided in the manuscript.

Field-specific reporting

Please select the one below that is the best fit for your research. If you are not sure, read the appropriate sections before making your selection.

☐ Life sciences ☒ Behavioural & social sciences ☐ Ecological, evolutionary & environmental sciences

For a reference copy of the document with all sections, see nature.com/documents/nr-reporting-summary-flat.pdf

Behavioural & social sciences study design

All studies must disclose on these points even when the disclosure is negative.

Study description	In this quantitative study, we use family trio designs in the MoBa cohort to demonstrate advantages of familial cohorts with genotype data
Research sample	Up to 234,000 mothers, fathers and children participating in the Norwegian Mother, Father, and Child Cohort Study (MoBa) with available genotype data. The children were born between 1999 and 2009. Currently children are aged 17-27, while parents are 34-88 years old. More detailed information on the cohort is available in the MoBa Cohort profiles (https://doi.org/10.1093/ije/dyl170 & https://doi.org/10.1093/ije/dyw029 & https://doi.org/10.1093/ije/dyaf139). Information about the representativeness of MoBa is available here: Nilsen et al. Self-selection and bias in a large prospective pregnancy cohort in Norway. Paediatr Perinat Epidemiol 2009; 23: 597-608. Biele et al. Bias from self-selection and loss to follow-up in prospective cohort studies. Eur J Epidemiol 2019 34(10), 927–938. Vejrup et al. Lost to follow-up in the Norwegian mother, father and child cohort study. Paediatr Perinat Epidemiol 2021;00:1–10.
Sampling strategy	MoBa is a population-based cohort with national recruitment at the universal ultrasound assessment in 1999-2009. The analyses in this manuscript relied on existing data, and we used the maximum available sample available after quality control. We did not conduct any a priori sample size calculation.
Data collection	The data had already been collected as part of MoBa and linked national registries. No blinding was conducted as this is an observational study. For our exemplar analyses, we included four phenotypes assessed in middle childhood (age 7-10 years), spanning physical health (height), mental health (depression symptoms) and aspects of functioning (sleep duration and educational achievement). Child height at age 7 years was reported by mothers in the age-7 questionnaire for the question “What is the child’s height and weight now at 7 years old?”. Mothers were asked to report their child’s current height in centimetres. Sleep duration at age 7 years was maternally reported in the 7-year-questionnaire on the item “Approximately how many hours does the child usually sleep on a weeknight?” with response categories 1) 8 hours or less, 2) 9 hours, 3) 10 hours, 4) 11 hours, 5) 12 hours or more. Depressive symptoms were measured using the 13-item Short Mood and Feelings Questionnaire57 (SMFQ) reported by mothers in the 8-year-questionnaire. We prepared the questionnaire-assessed phenotypes using the phenotools R package (0.2.8)58 in R 4.1.0 (R Core Team, 2022). Educational achievement at age 10 was assessed as a composite score based on performance on three national standardized tests of skills in literacy, numeracy and English, derived from the Statistics Norway Educational Registry. The national tests are administered in the autumn of grade 5 (age 10), 8 (age 13) and 9 (age 14), and are mandatory with exemptions only upon application on the grounds that the results will not be useful for assessing the child's learning due to disability or lack of knowledge of the Norwegian language. Because the raw score ranges differed across subjects and test years, the raw scores were standardized within each test year and subject test. All the four phenotype scores were standardized for use in the trio analyses to place them on a common scale and to facilitate interpretation and comparison of effect sizes.
Timing	MoBa recruited pregnant mothers and their partners from July 1999 to December 2008.
Data exclusions	At the beginning of the project, 234,505 successfully genotyped samples with valid informed consent were available, corresponding to 230,436 unique individuals. Throughout the implementation of the MoBaPsychGen pipeline, 22,867 individuals were excluded due to failure to meet one or more of the pre-specified exclusion criteria and/or withdrawal of consent, as described in the Supplementary Information. Following implementation of the MoBaPsychGen pipeline, 207,569 unique individuals passed all pre-specified exclusion criteria and were available for downstream analyses.<br>Exemplar trio analyses were conducted in subsets of these QC-passed individuals with complete genotyped mother–father–child trios and available phenotype data. Pre-specified exclusion criteria for trio analyses included incomplete trios, missing phenotype data, phenotype outliers (defined as values more than 4 standard deviations from the mean), and withdrawal of consent during implementation of analyses.<br>For trio-GWAS analyses, 41,761 trios were included for educational achievement (N individuals excluded = 82,286), 23,786 for height (N individuals excluded = 136,211), 24,376 for sleep duration (N individuals excluded = 134,441), and 18,701 for depressive symptoms (N individuals excluded = 151,466).<br>For trio-GCTA analyses, an additional exclusion criterion was applied to retain only one sibling per family, resulting in 23,221 trios for educational achievement (N individuals excluded = 137,906), 12,332 for height (N individuals excluded = 170,573), 12,799 for sleep duration (N individuals excluded = 169,172), and 10,794 for depressive symptoms (N individuals excluded = 175,187).<br>For trio-PGS analyses, 40,101 trios were included for educational achievement (N individuals excluded = 87,266), 21,008 for height (N individuals excluded = 144,545), 21,451 for sleep duration (N individuals excluded = 143,216), and 18,212 for depressive symptoms (N individuals excluded = 152,933).<br>PTDT analyses were conducted in a subset of MoBa families identified by selecting genotyped children with extreme phenotype values: height (>2 SD above the mean; N = 528), educational achievement (>90th percentile; N = 3,230), sleep duration (<10th percentile; N = 438), and depressive symptoms (>2 SD above the mean; N = 618). Individuals were included only if parental average

	phenotype values were not similarly extreme and complete genotype and phenotype data were available. Sibling controls (N = 84, 535, 78, and 95 for height, educational achievement, sleep duration, and depressive symptoms, respectively) were included in the analyses. All inclusion criteria were pre-specified.
Non-participation	All pregnant women attending the universal ultrasound assessment from July 1999 to December 2009 were invited; 41% participated
Randomization	No randomisation was conducted since this is an observational study.

Reporting for specific materials, systems and methods

We require information from authors about some types of materials, experimental systems and methods used in many studies. Here, indicate whether each material, system or method listed is relevant to your study. If you are not sure if a list item applies to your research, read the appropriate section before selecting a response.

Materials & experimental systems

n/a	Involved in the study
☒	☐ Antibodies
☒	☐ Eukaryotic cell lines
☒	☐ Palaeontology and archaeology
☒	☐ Animals and other organisms
☒	☐ Clinical data
☒	☐ Dual use research of concern
☒	☐ Plants

Methods

n/a	Involved in the study
☒	☐ ChIP-seq
☒	☐ Flow cytometry
☒	☐ MRI-based neuroimaging

Plants

Seed stocks	NA
Novel plant genotypes	NA
Authentication	NA
