# Supplementary material for: Family genetic designs in MoBa provide insights into health and functioning
Source: Nature. 2026 Aug 19;658(8136):707–15. doi: 10.1038/s41586-026-10926-5 (PMC13668695; doi:10.1038/s41586-026-10926-5)
Supplement: Supplementary file 3 [file 41586_2026_10926_MOESM3_ESM.pdf]

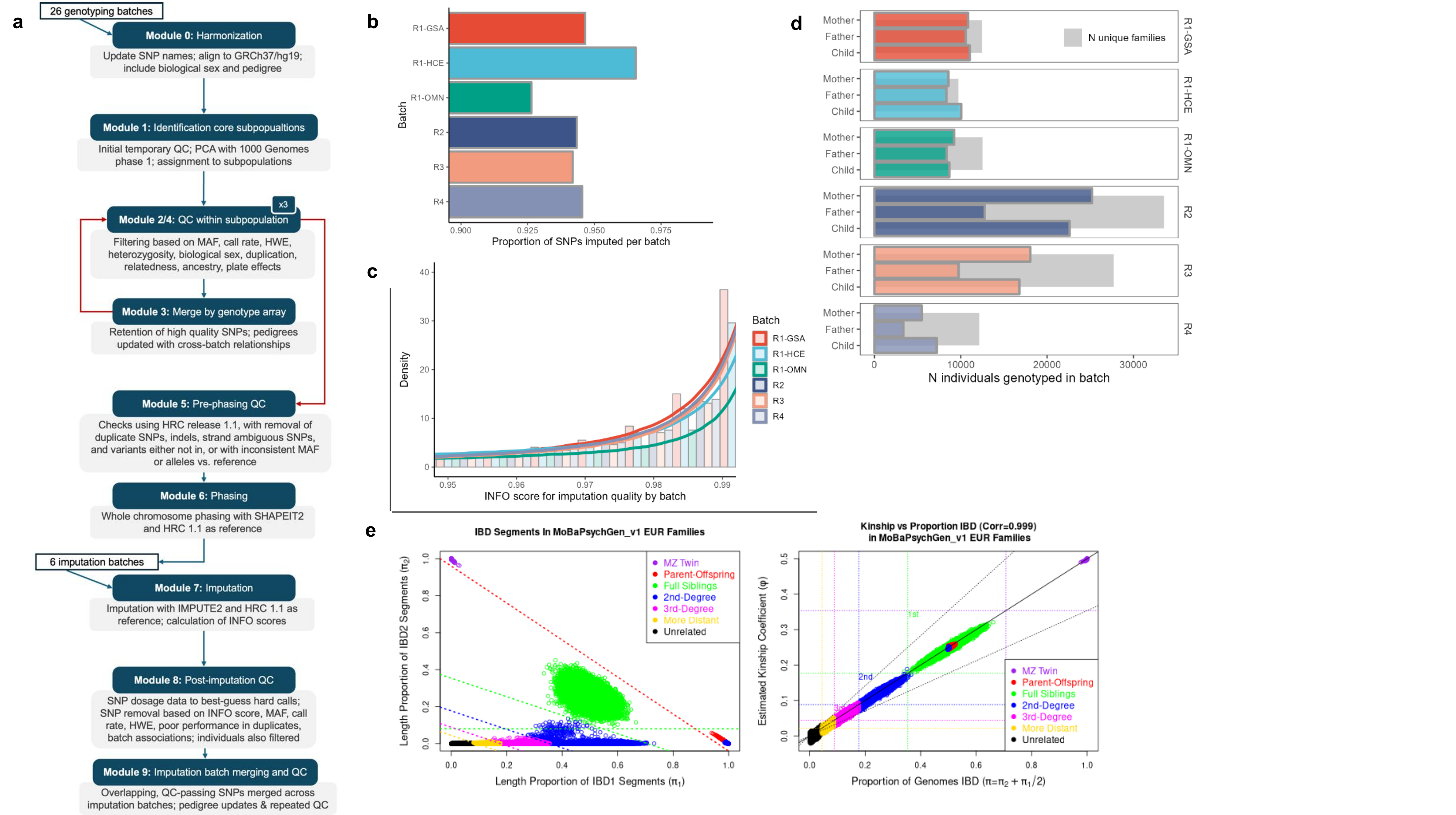

Supplementary Figure 1. Genotyping and quality control (QC) of the Norwegian Mother, Father, and Child Cohort Study (MoBa) makes n=44,017 trios available for genetic analyses. a, Overview of the modules comprising the MoBaPsychGen QC pipeline applied to the n=234,505 samples of genotype data provided by MoBa participants. **b**, Batch-wise imputation rates and, **c**, Imputation quality, showing all batches with >90% of SNPs imputed, and high imputation quality for most SNPs across all batches. **d**, Individuals and unique families included in each imputation batch, showing more trios in earlier batches and more unrelated individuals and mother-child duos in later batches. **e**, Inferred pedigree relationships in MoBa as a function of the proportion of alleles identical by descent (IBD) and estimated kinship coefficients.

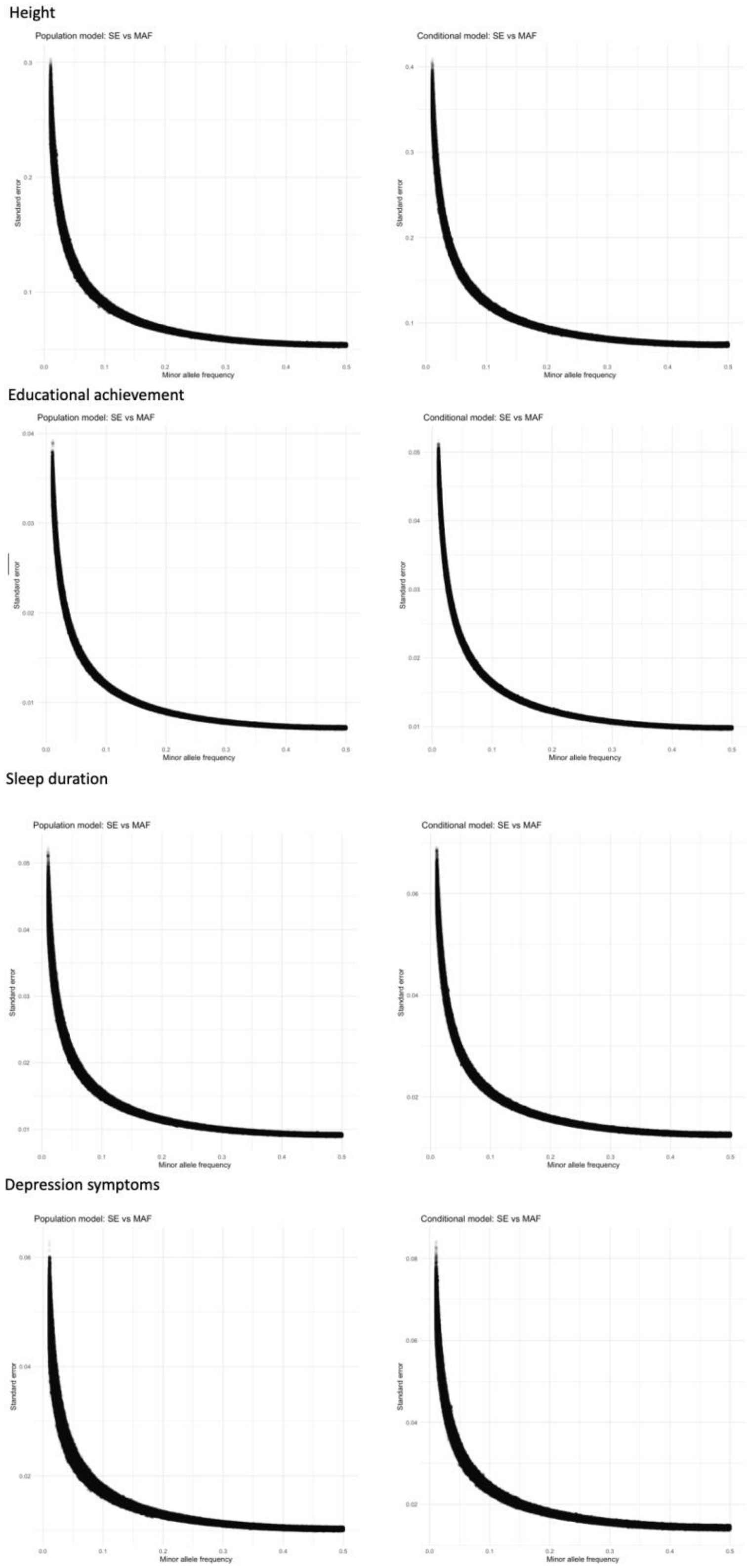

Supplementary Figure 2. Two-by-two scatter plots illustrating the relationship between the minor allele frequency (MAF) in the parents (proxied by offspring MAF, which by definition will equal the MAF in the parents on average) and the standard error of the population-based (left) and within-family (right) estimates. Plots from top to bottom are height (n=23,786 samples), educational achievement (n=41,761 samples), sleep duration (n=24,376 samples), and depression symptoms (18,701 samples), respectively.

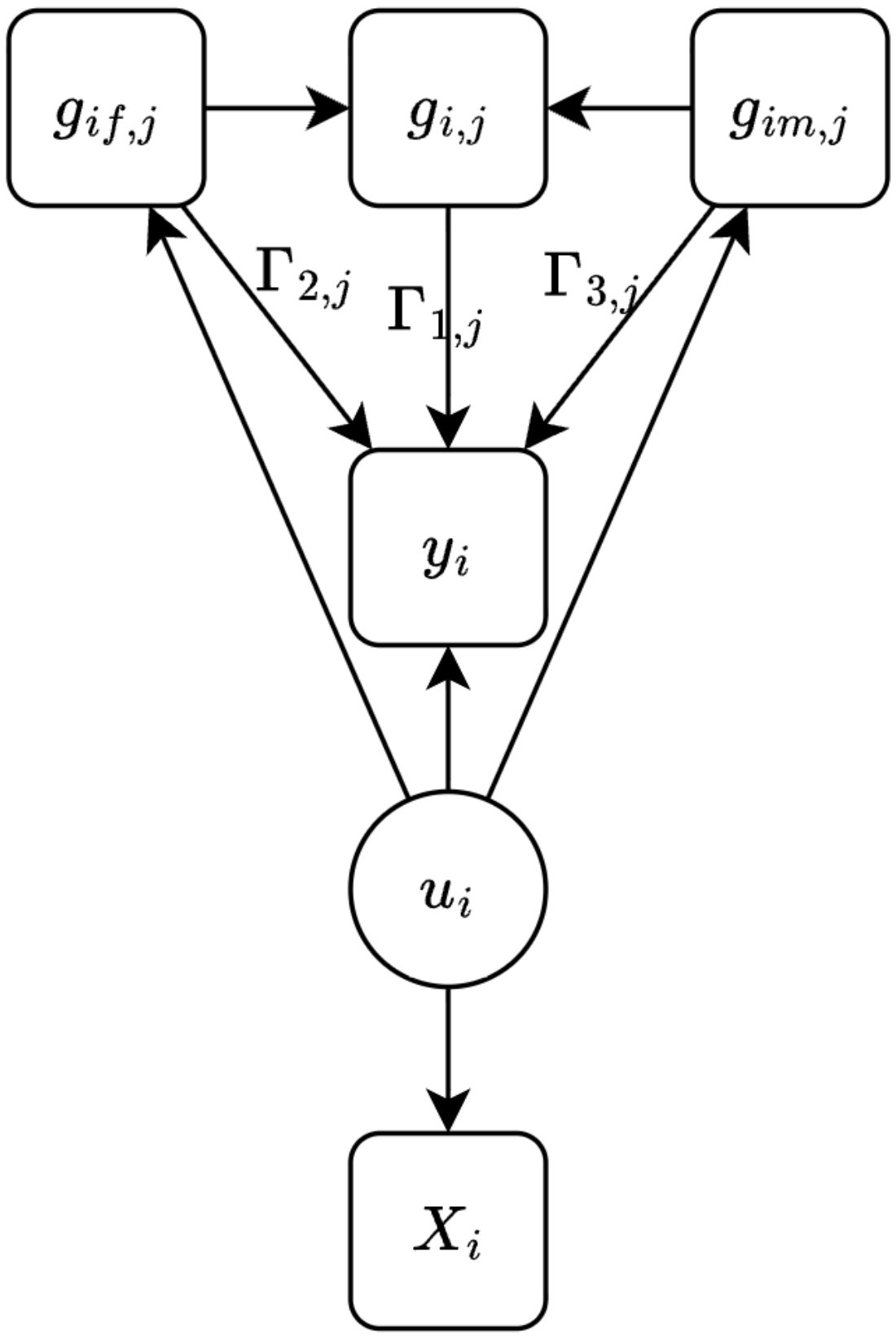

Supplementary Figure 3. A Directed Acyclic Graph illustrating the relationships between offspring i at variant $g_{i,j}$, maternal genotype $g_{im,j}$, paternal genotype $g_{if,j}$ and offspring phenotype y_i. It represents the estimation model described in text. The unobserved confounding term $u_{i,j}$ influences the outcome and the exposure, for example, via population structure, which also influences the allele frequencies of the parental genotypes. In practice, many population-based studies attempt to capture this bias by using genetic principal components (X_i), but there is no guarantee of the PCs completely controlling this confounding. Alternatively, if the parents' genotypes are controlled for, then all confounding paths between the offspring genotype and the offspring phenotype are blocked, and the only remaining path in this graph is the direct causal effect from the offspring genotype to the offspring phenotype.

Population estimation model:

$$y_i = \alpha + \Gamma_{1,j} g_{i,j} + \boldsymbol{\beta} \boldsymbol{X_i} + e_i$$

where y_i is the offspring phenotype for individual i, α is an intercept term, $\Gamma_{1,j}$ represents the direct effect of the jth variant $g_{i,j}$ on the phenotype, $\boldsymbol{X_i}$ is a vector of covariates including age, sex registered at birth, genotyping batch and the first twenty genetic principal components for the individual with corresponding vector of effects $\boldsymbol{\beta}$ on the phenotype, and e_i is the residual error.

Within-family estimation model:

$$y_i = \alpha + \Gamma_{1,j} g_{i,j} + \Gamma_{2,j} g_{if,j} + \Gamma_{3,j} g_{im,j} + \boldsymbol{\beta} \boldsymbol{X_i} + e_i$$

where $g_{if,j}$ and $g_{im,j}$ are the parental genotypes at variant j for mother and father of the offspring i, and $\Gamma_{2,j}$ and $\Gamma_{3,j}$ represent their respective indirect effects on the offspring phenotype y_i.

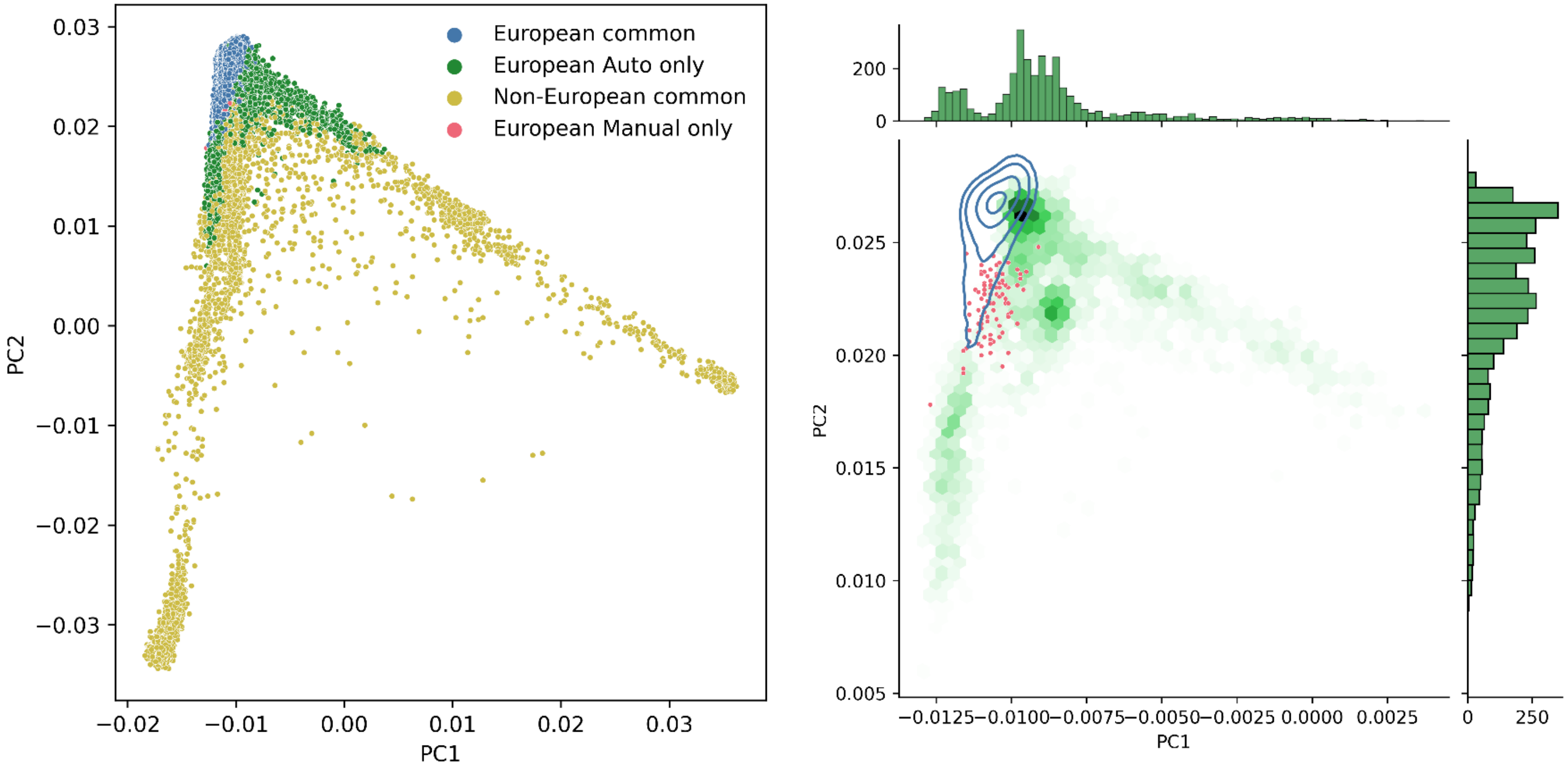

Supplementary Figure 4. PC1 vs PC2 plots from testing of visual clustering using 1000 Genomes phase 1 data verses automated clustering supervised using 1000 Genomes phase 3 data. The plot on the left shows the results from all 26 MoBa genotyping batches colored blue for individuals identified as clustering with the European superpopulation in both clustering methods (n=224,353 samples), green for individuals identified as clustering with the European superpopulation in automated clustering only (n=3,351 samples), red for individuals identified as clustering with the European superpopulation in the visual only (n=95 samples), and yellow for individuals identified as not clustering with the European superpopulation in both automated and visual clustering (n=5,769 samples). The plot on the right shows the blue contour lines correspond to proportion levels: 0.01, 0.05, 0.25 and 0.75, with 99% of individuals identified as clustering with the European superpopulation common for both automatic and visual clustering within the outer-most contour line, 95% within the second outer contour line, 75% within the third contour line, and 25% within the inner-most contour line. Marginal green histograms show counts of samples identified as clustering with the European superpopulation in automatic clustering but not in visual clustering and correspond to the green hexagons showing 2D density of the same samples. The red points are the individuals identified as clustering with the European superpopulation from visual clustering only.

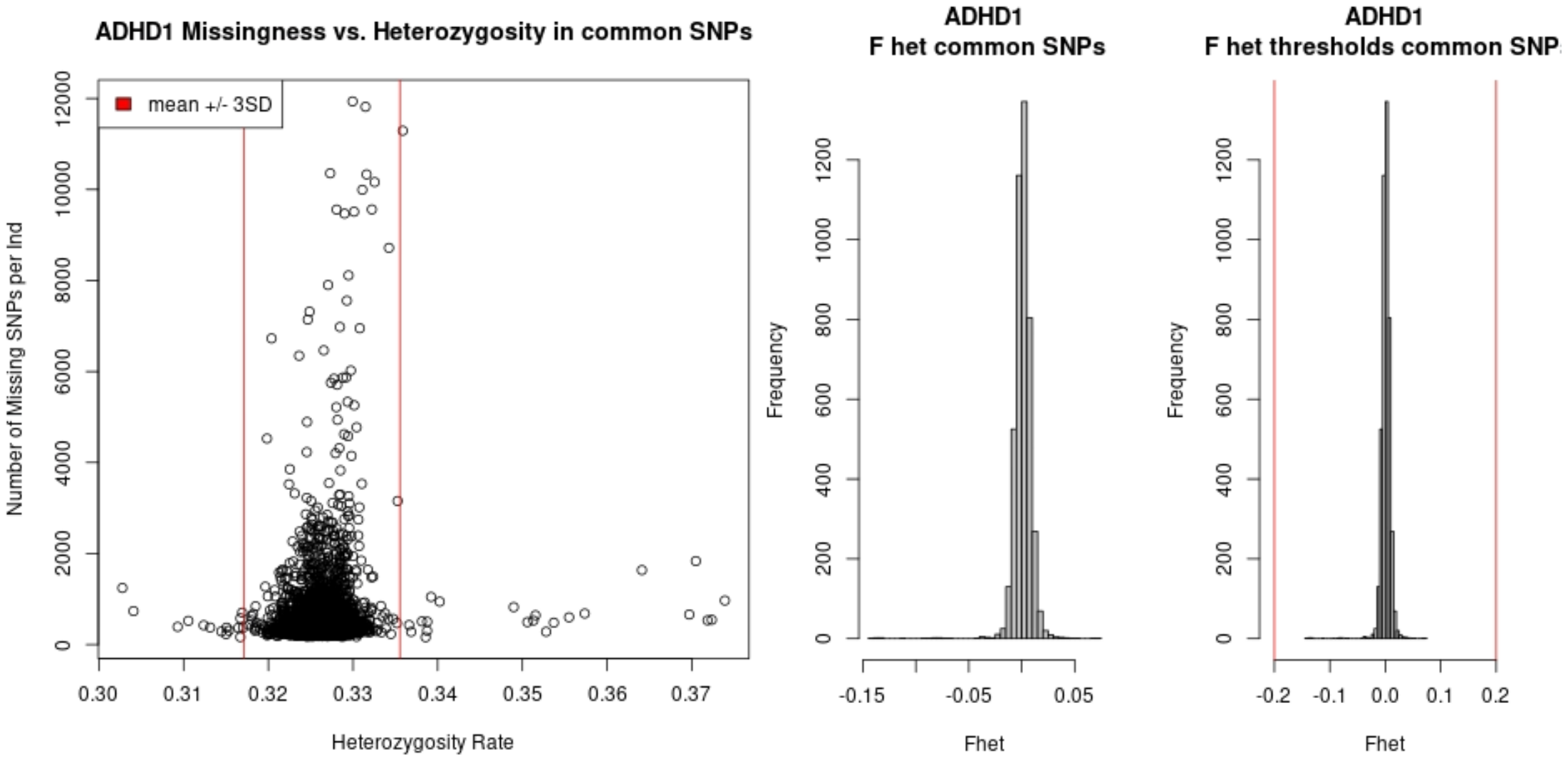

Supplementary Figure 5. Heterozygosity plots from the ADHD1 batch (n=4,407 samples). Left plot shows the heterozygosity rate vs number of missing SNPs, with red mean ±3 SD thresholds. Middle plot shows the Fhet vs Frequency plot. Right plot shows the Fhet vs Frequency plot with red Fhet |0.2| thresholds.

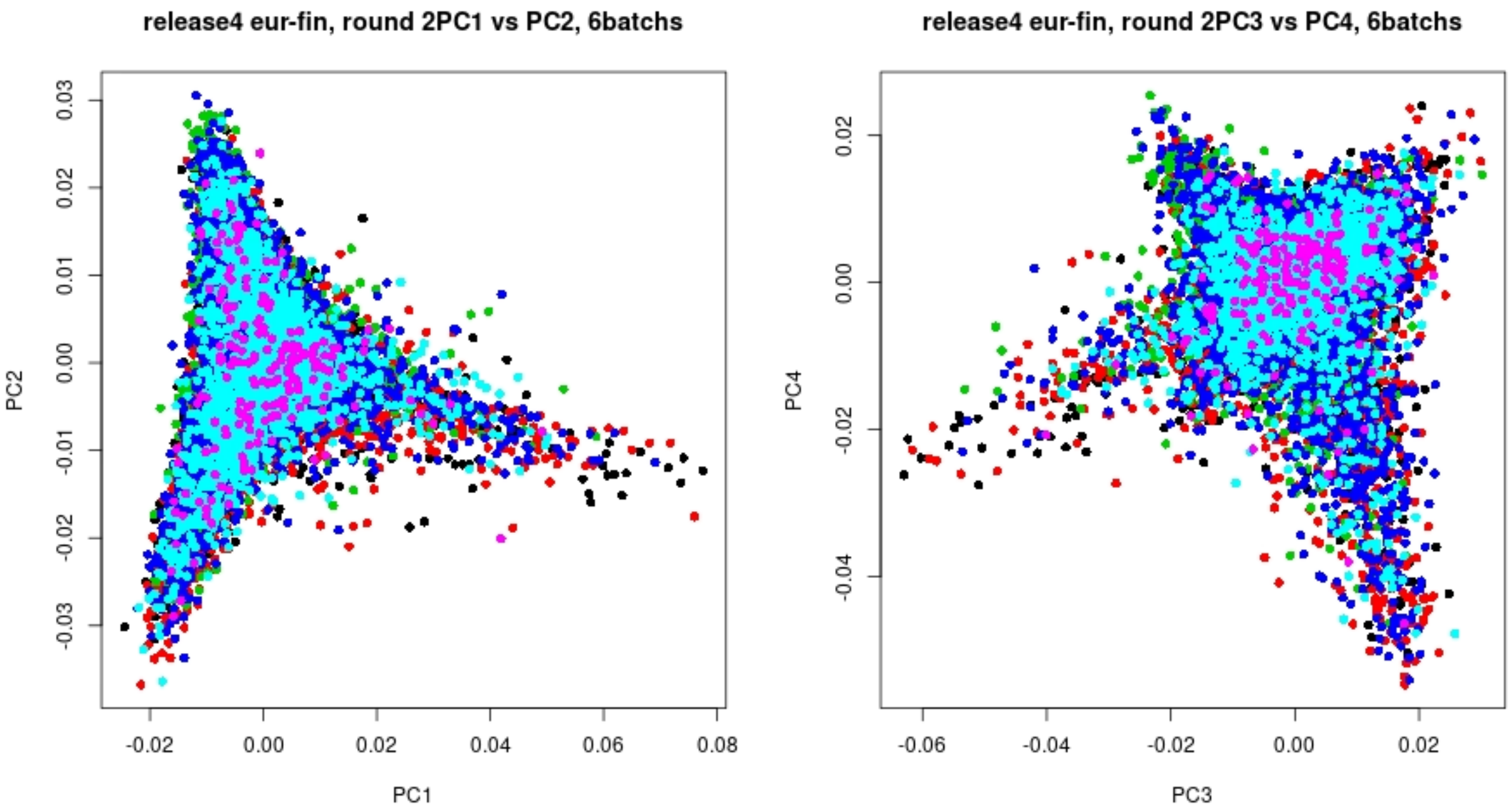

Supplementary Figure 6. PC1 vs PC2 and PC3 vs PC4 plots colored by batch. Black: ADHD2 n=2,198 samples, red: NORMENT_Mar2021_1273 n=4,997 samples, dark blue: NORMENT_Mar2021_1409 n=2,501 samples, green: NORMENT_Mar2021_1413 n=5,178 samples, light blue: NORMENT_Mar2021_1531 n=1,795 samples, pink: NORMENT_Mar2021_1532 n=195 samples.

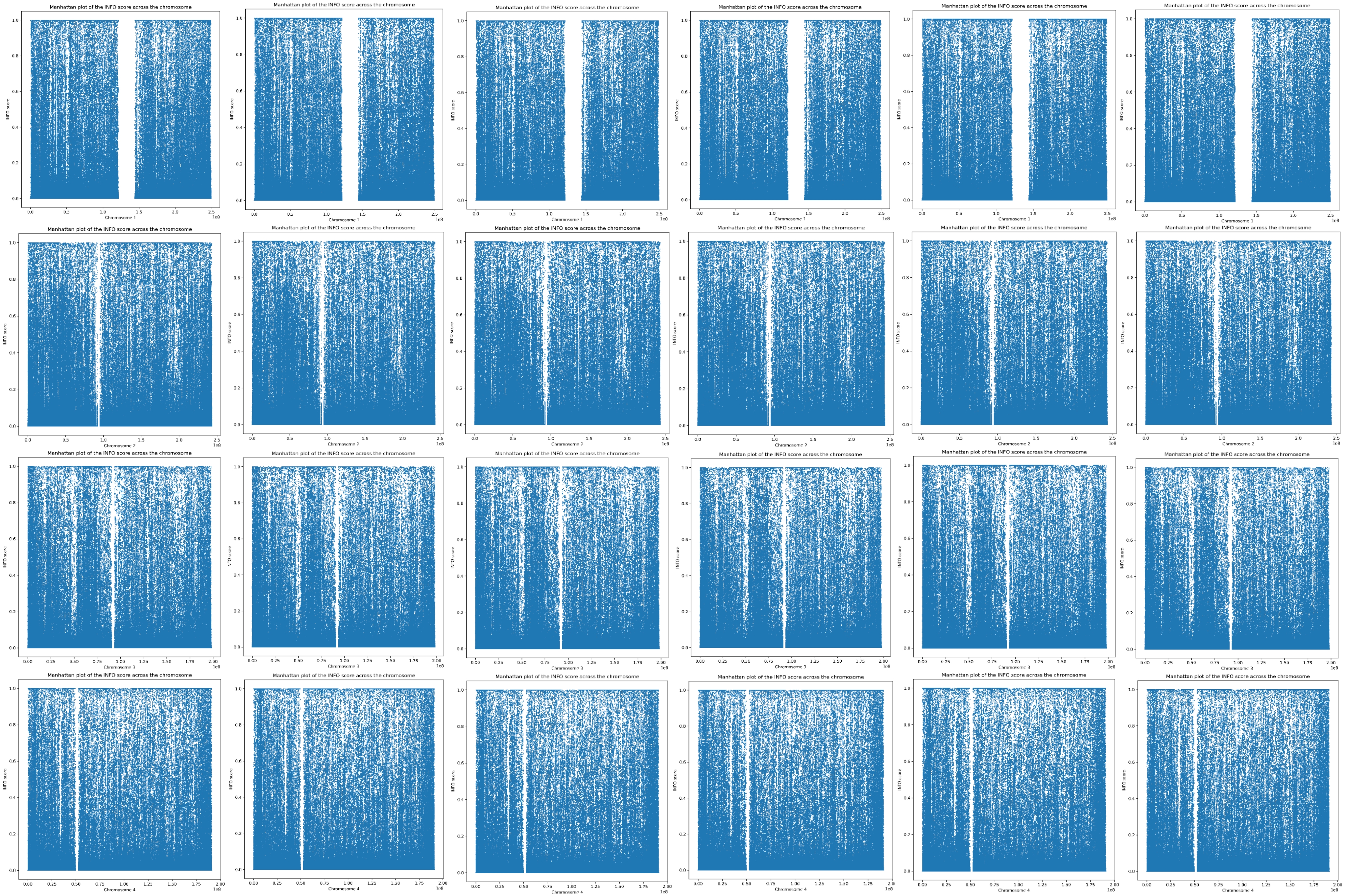

Manhattan plot of the INFO score across the chromosome
INFO score
Chromosome 1
Chromosome 2
Chromosome 3
Chromosome 4

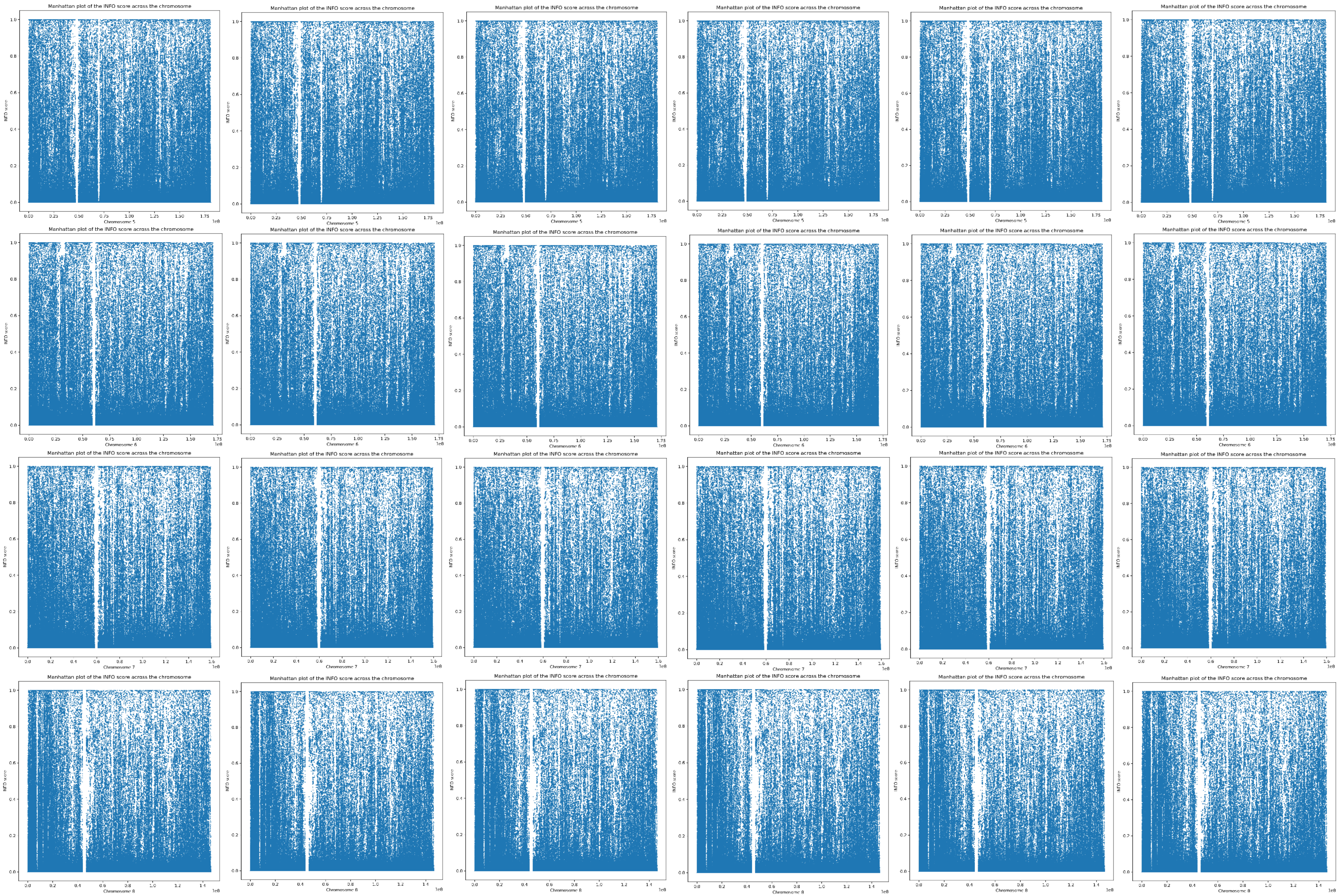
Manhattan plot of the INFO score across the chromosome
INFO score
Chromosome 5
Chromosome 6
Chromosome 7
Chromosome 8
1e8

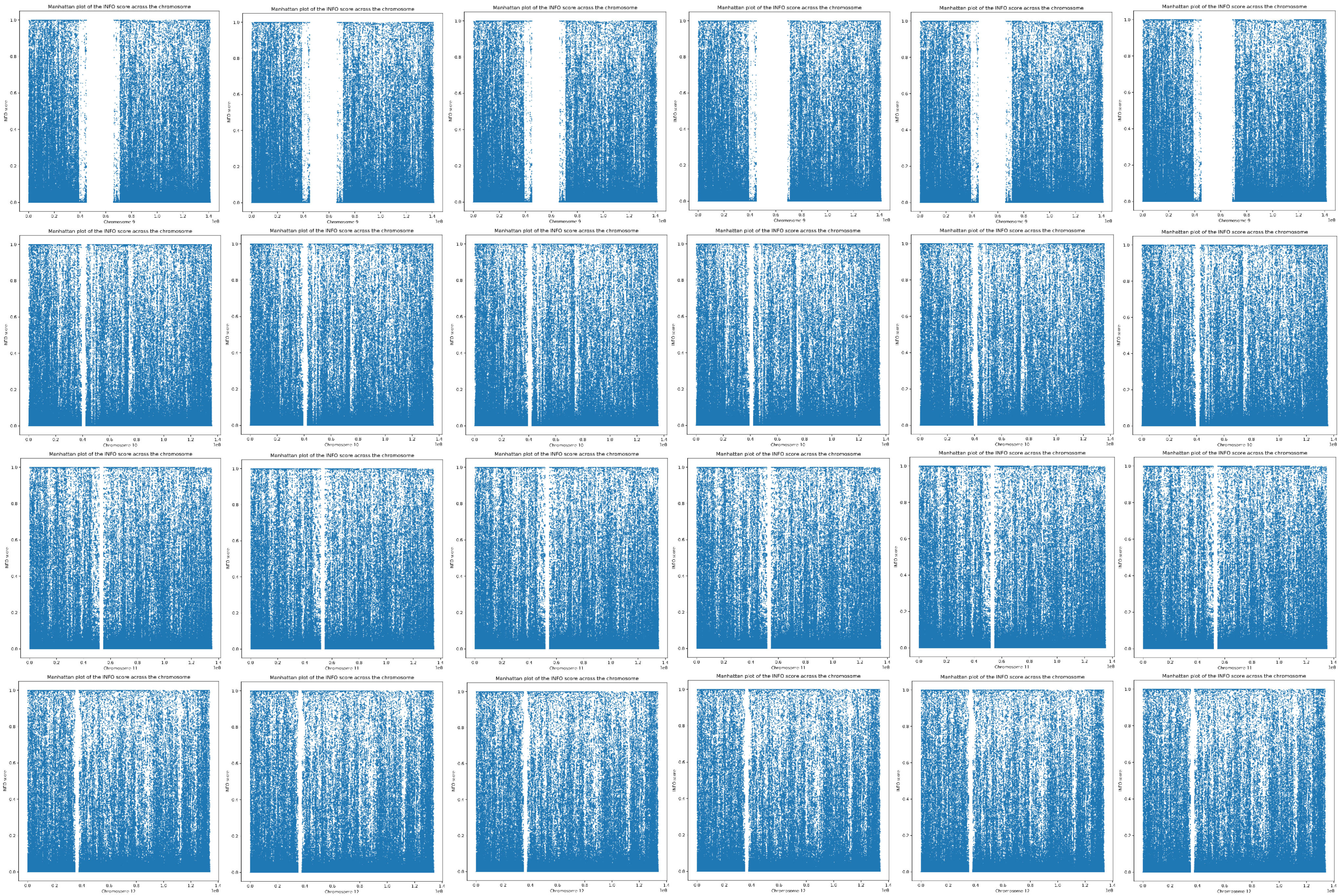
Manhattan plot of the INFO score across the chromosome
INFO score
Chromosome 9
Chromosome 10
Chromosome 11
Chromosome 12
1e8

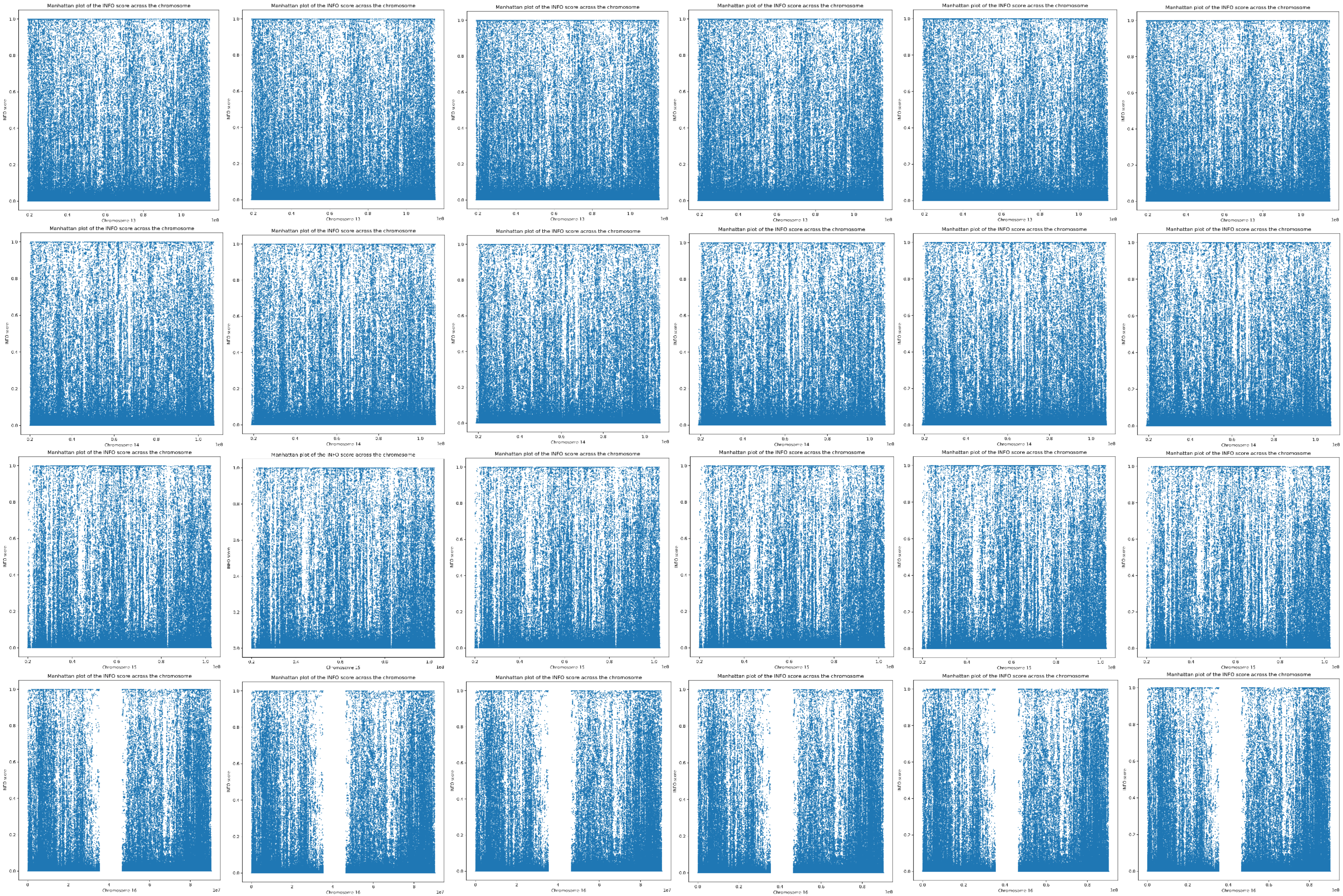
Manhattan plot of the INFO score across the chromosome
INFO score
Chromosome 13
Chromosome 14
Chromosome 15
Chromosome 16
1e8
1e7

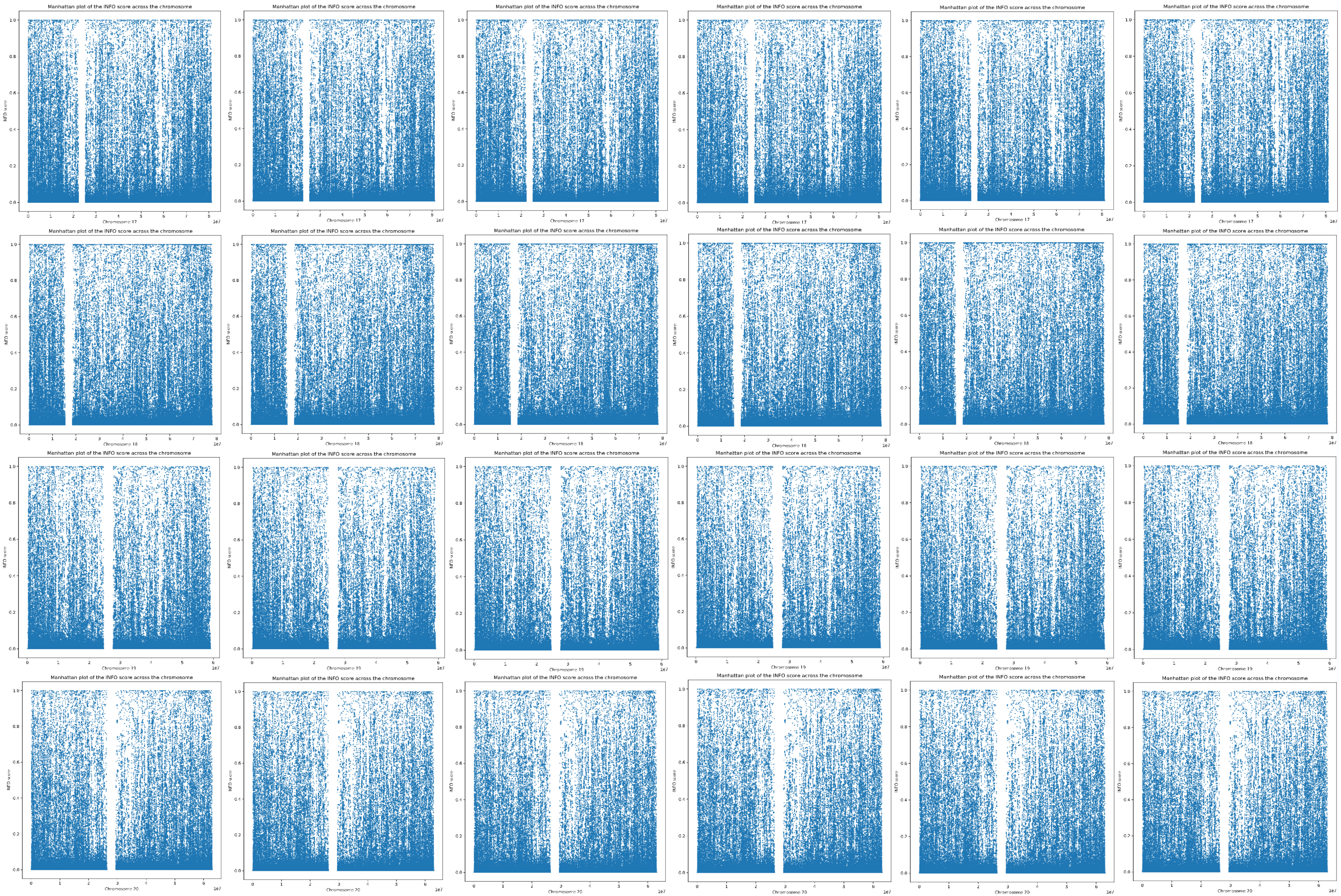
Manhattan plot of the INFO score across the chromosome
INFO score
Chromosome 17
Chromosome 18
Chromosome 19
Chromosome 20
1e7

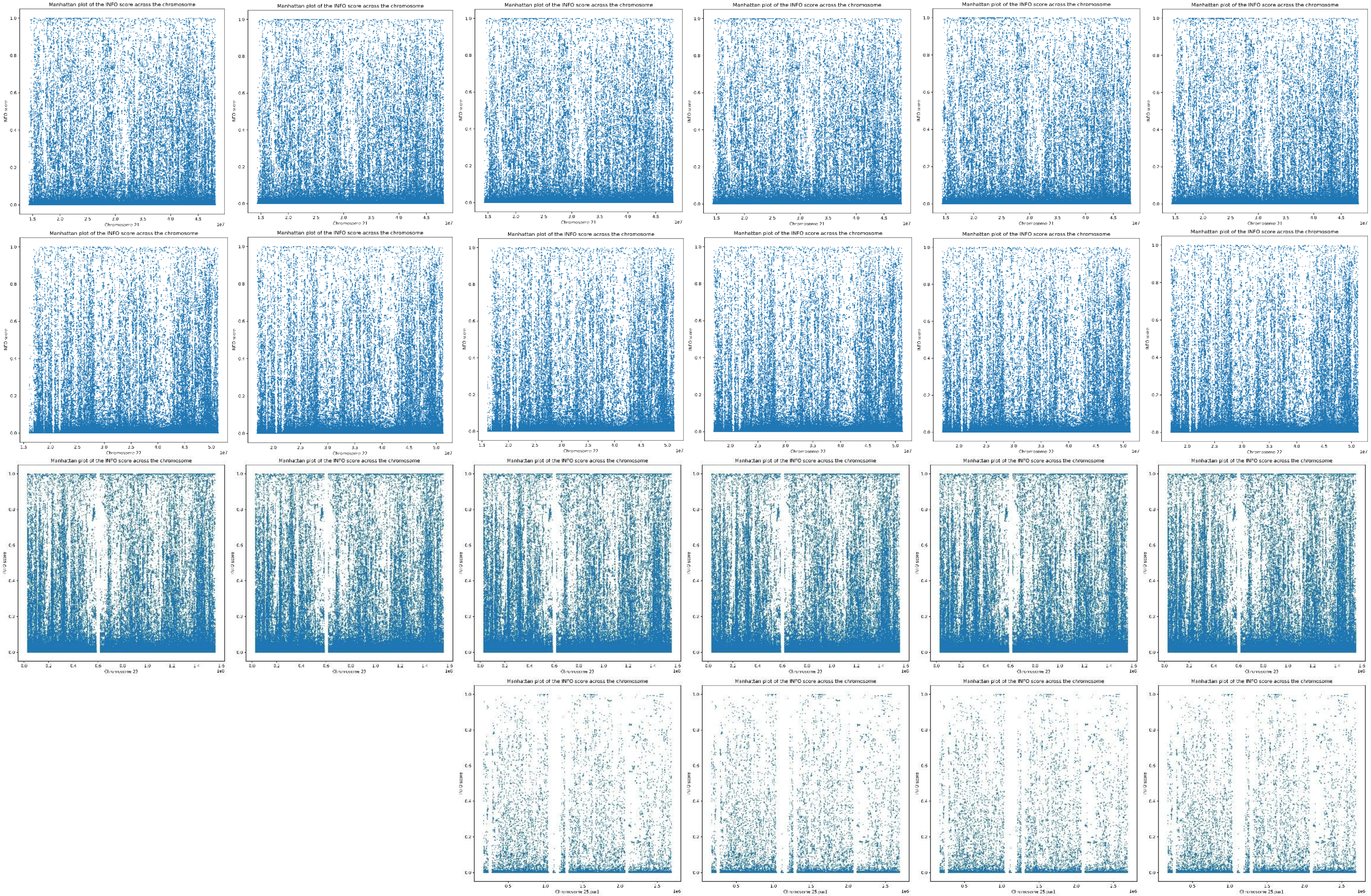
Manhattan plot of the INFO score across the chromosome
INFO score
Chromosome 21
Chromosome 22
Chromosome 23

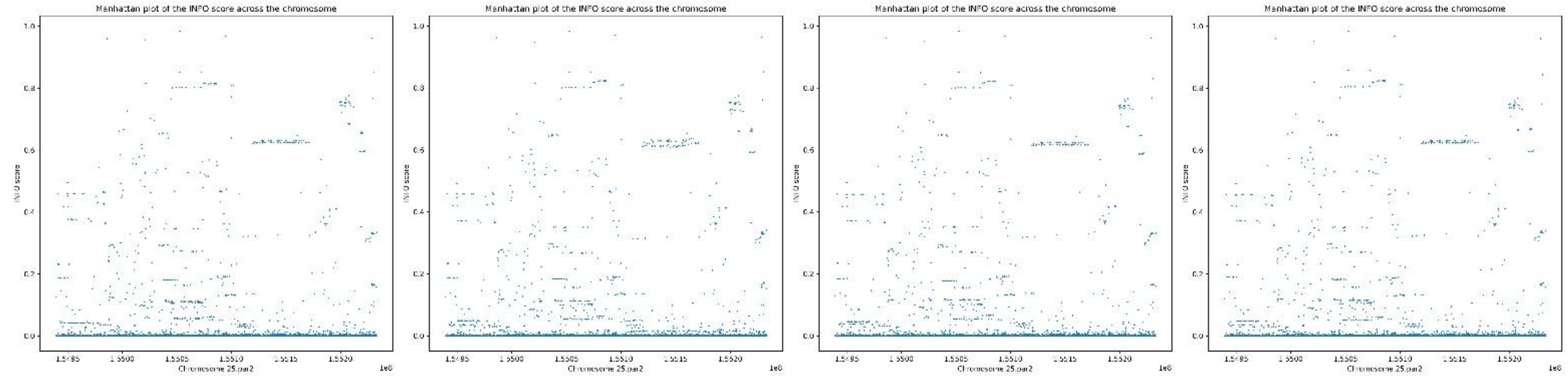

Supplementary Figure 7. Imputation quality plots showing chromosome position vs imputation quality scores (INFO) for each chromosome and MoBa imputation batch. Plots from left to right are Imputation batch HCE, OMNI, GSA1, GSA2, GSA3, and GSA4. Plots from top to bottom are chromosome 1-22 (HCE n=30,859 samples, OMNI n=26,891 samples, GSA1 n=33,893 samples, GSA2 n=62,181 samples, GSA3 n=45,624 samples, and GSA4 n=16,768 samples), chromosome X (HCE n=26,421 samples, OMNI n=26,004 samples, GSA1 n=32,289 samples, GSA2 n=60,163 samples, GSA3 n=44,139 samples, and GSA4 n=15,897 samples), and pseudoautosomal region 1 and 2 (HCE n=NA samples, OMNI n=NA samples, GSA1 n=29,213 samples, GSA2 n=60,163 samples, GSA3 n=44,139 samples, and GSA4 n=15,897 samples).

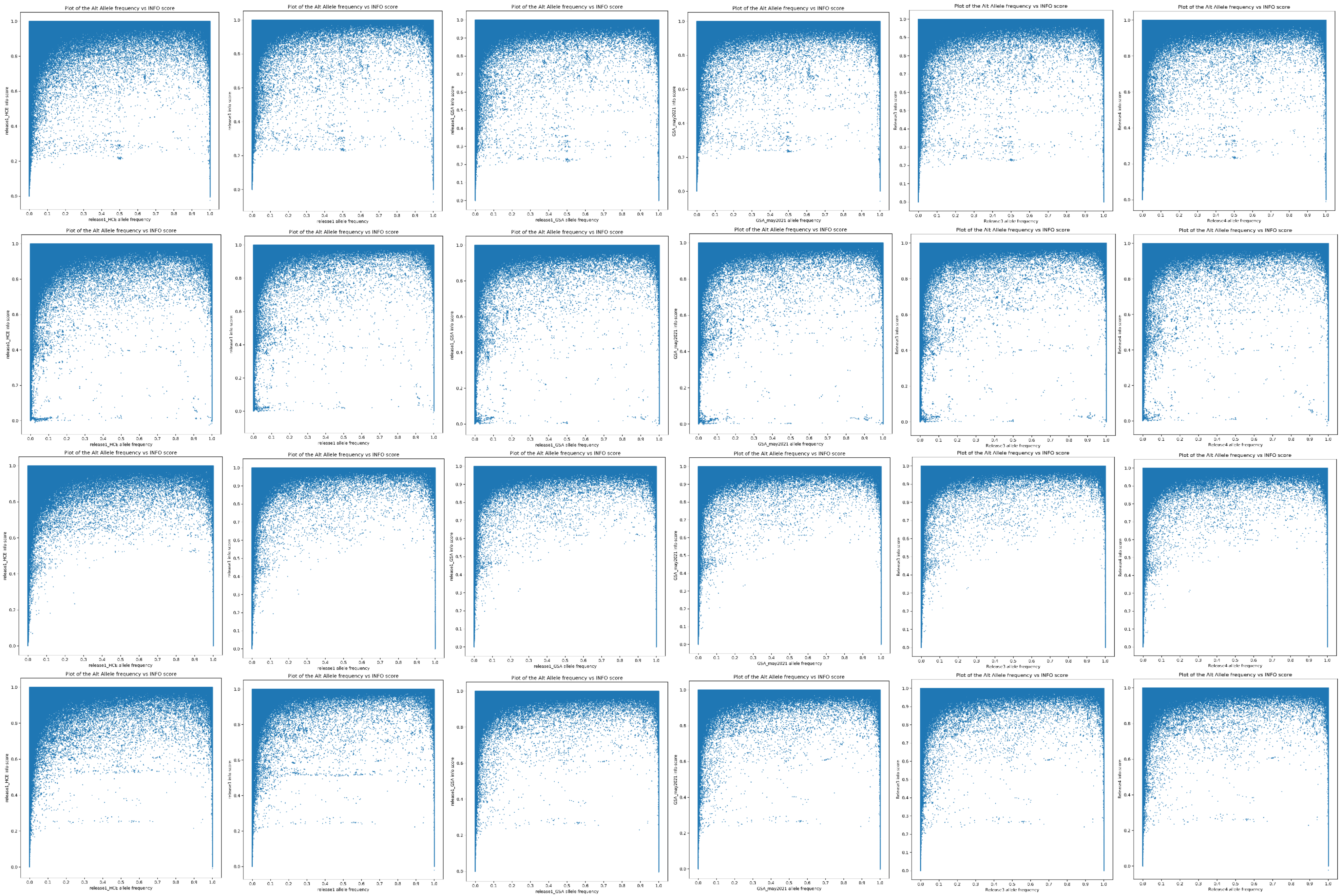
Plot of the Alt Allele frequency vs INFO score
release1_HCE allele frequency
release1_HCE info score
release1 allele frequency
release1 info score
release1_GSA allele frequency
release1_GSA info score
GSA_may2021 allele frequency
GSA_may2021 info score
Release3 allele frequency
Release3 info score
Release4 allele frequency
Release4 info score

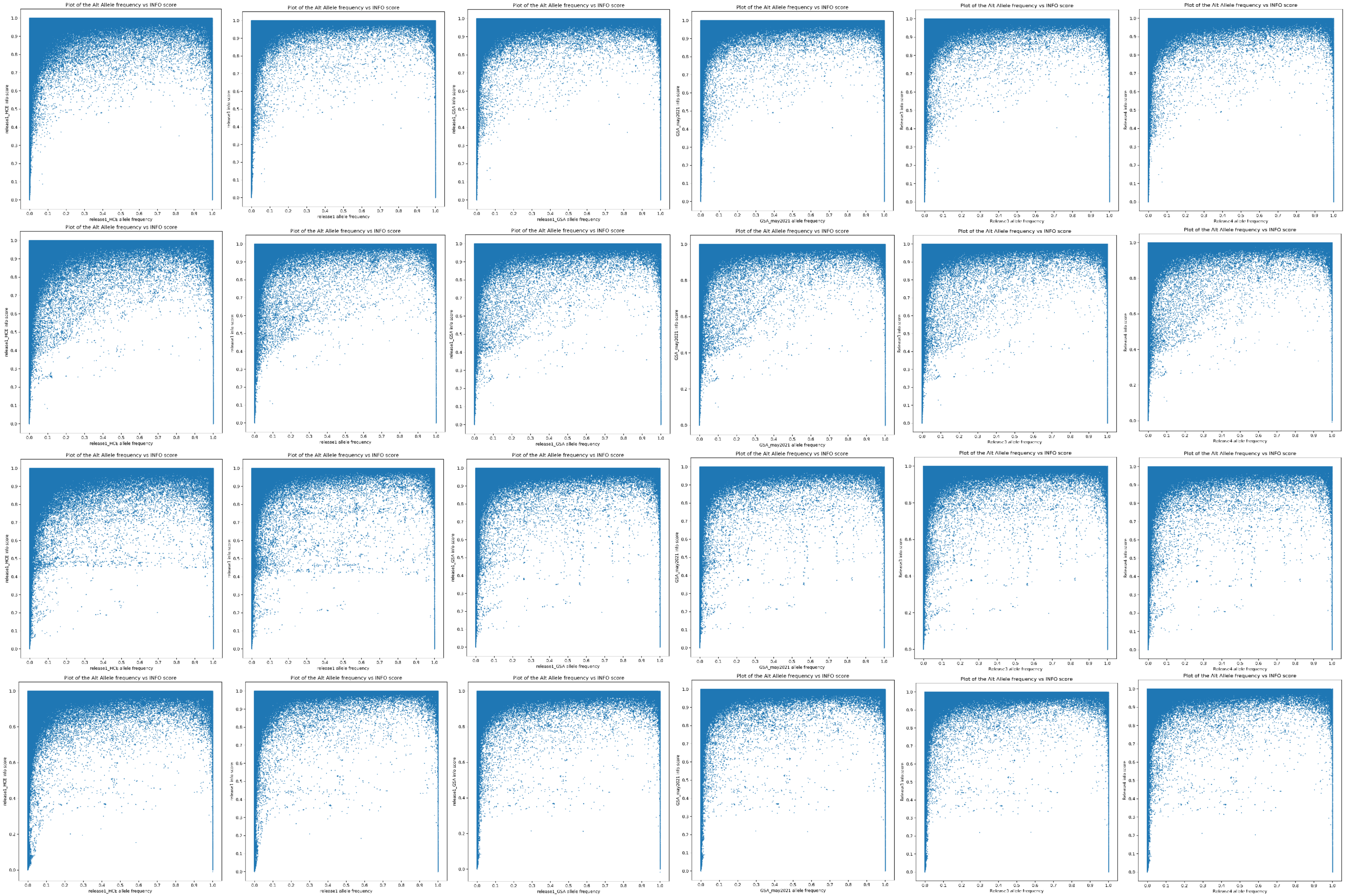
Plot of the Alt Allele frequency vs INFO score
release1_HCE info score
release1_HCE allele frequency
release1 info score
release1 allele frequency
release1_GSA info score
release1_GSA allele frequency
GSA_may2021 info score
GSA_may2021 allele frequency
Release3 info score
Release3 allele frequency
Release4 info score
Release4 allele frequency

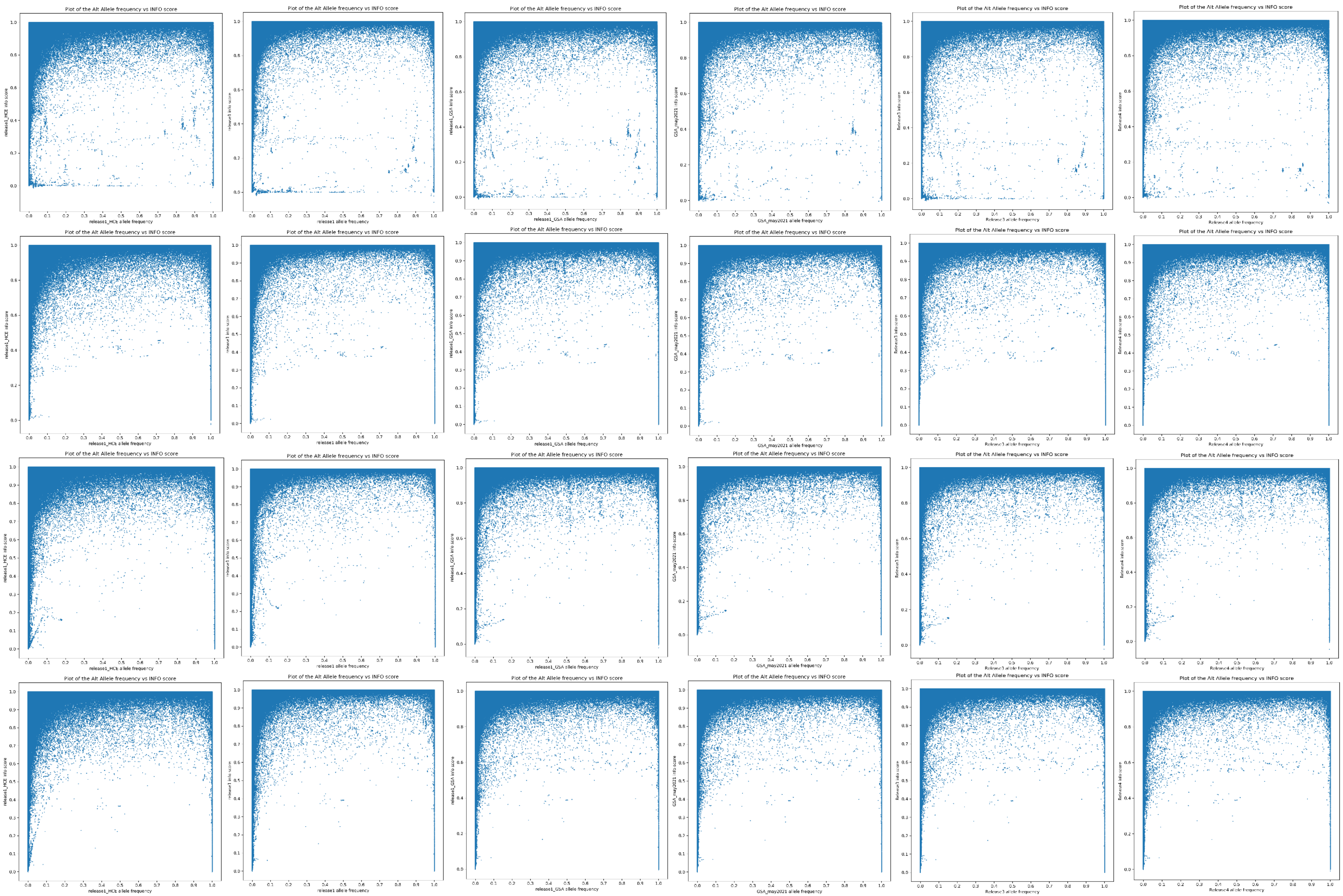
Plot of the Alt Allele frequency vs INFO score
release1_HCE allele frequency
release1_HCE info score
release1 allele frequency
release1 info score
release1_GSA allele frequency
release1_GSA info score
GSA_may2021 allele frequency
GSA_may2021 info score
Release3 allele frequency
Release3 info score
Release4 allele frequency
Release4 info score

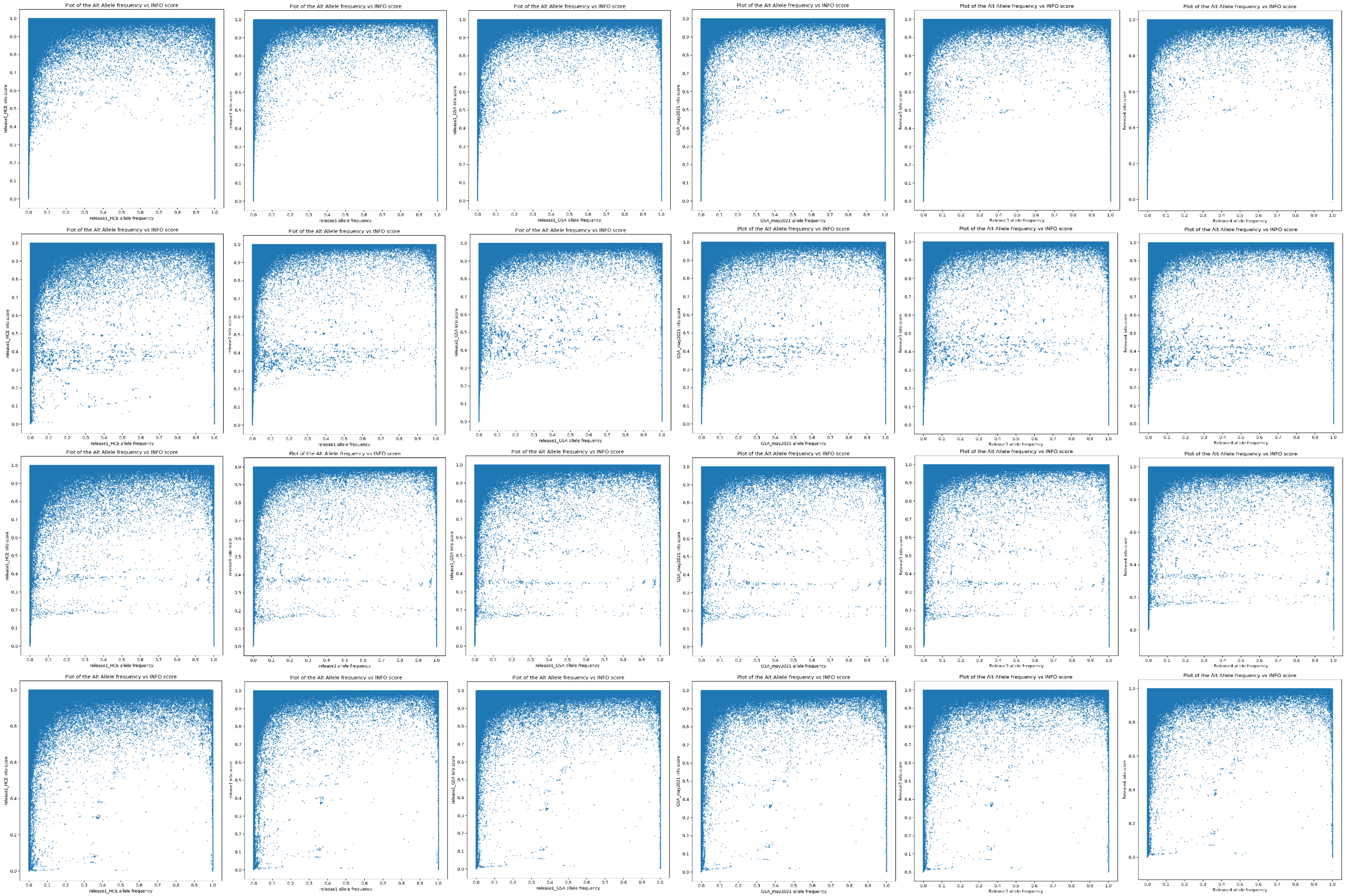
Plot of the Alt Allele frequency vs INFO score
release1_HCE allele frequency
Plot of the Alt Allele frequency vs INFO score
release1 allele frequency
Plot of the Alt Allele frequency vs INFO score
release1_GSA allele frequency
Plot of the Alt Allele frequency vs INFO score
GSA_may2021 allele frequency
Plot of the Alt Allele frequency vs INFO score
Release3 allele frequency
Plot of the Alt Allele frequency vs INFO score
Release4 allele frequency
Plot of the Alt Allele frequency vs INFO score
release1_HCE allele frequency
Plot of the Alt Allele frequency vs INFO score
release1 allele frequency
Plot of the Alt Allele frequency vs INFO score
release1_GSA allele frequency
Plot of the Alt Allele frequency vs INFO score
GSA_may2021 allele frequency
Plot of the Alt Allele frequency vs INFO score
Release3 allele frequency
Plot of the Alt Allele frequency vs INFO score
Release4 allele frequency
Plot of the Alt Allele frequency vs INFO score
release1_HCE allele frequency
Plot of the Alt Allele frequency vs INFO score
release1 allele frequency
Plot of the Alt Allele frequency vs INFO score
release1_GSA allele frequency
Plot of the Alt Allele frequency vs INFO score
GSA_may2021 allele frequency
Plot of the Alt Allele frequency vs INFO score
Release3 allele frequency
Plot of the Alt Allele frequency vs INFO score
Release4 allele frequency
Plot of the Alt Allele frequency vs INFO score
release1_HCE allele frequency
Plot of the Alt Allele frequency vs INFO score
release1 allele frequency
Plot of the Alt Allele frequency vs INFO score
release1_GSA allele frequency
Plot of the Alt Allele frequency vs INFO score
GSA_may2021 allele frequency
Plot of the Alt Allele frequency vs INFO score
Release3 allele frequency
Plot of the Alt Allele frequency vs INFO score
Release4 allele frequency

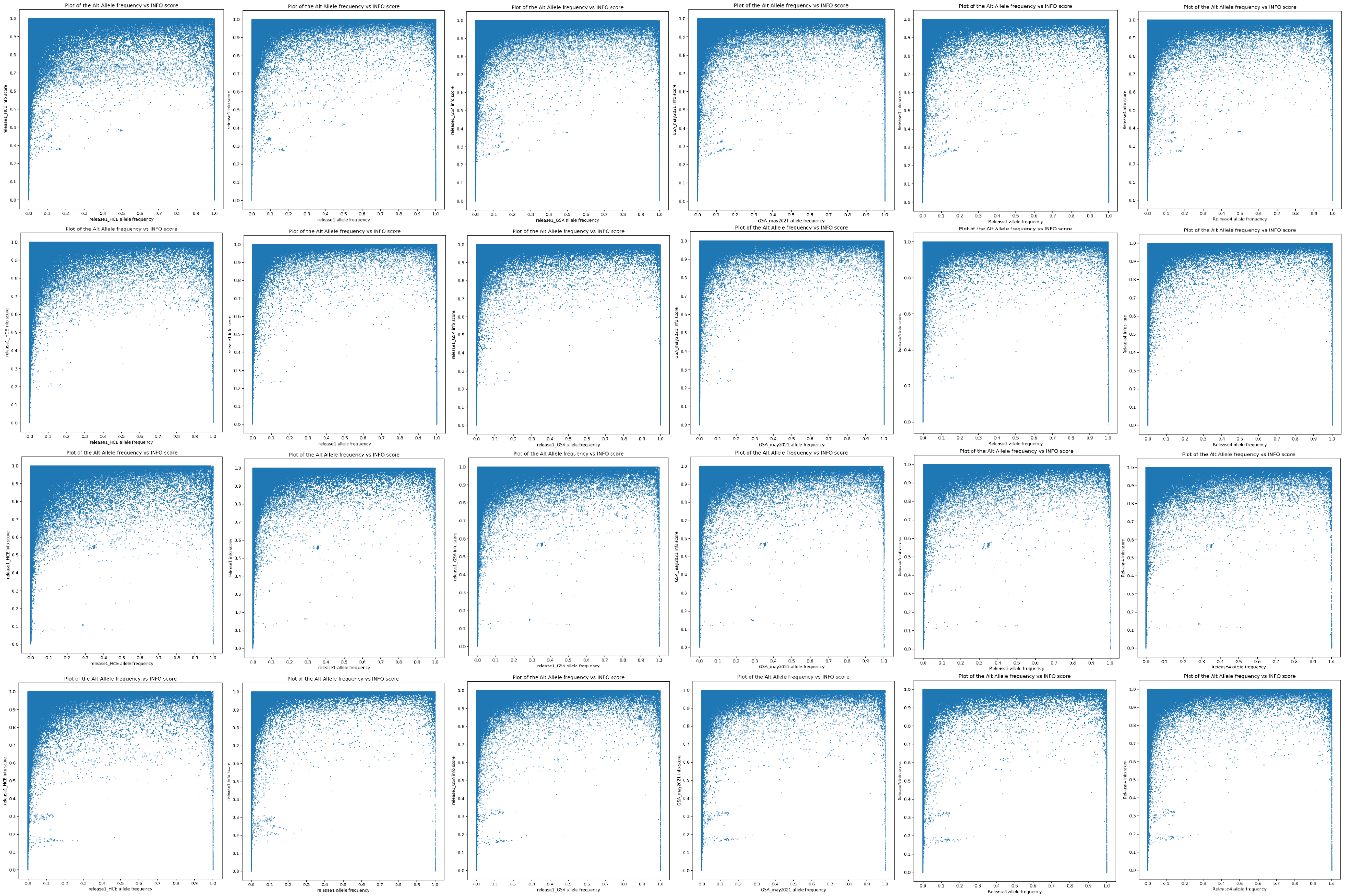
Plot of the Alt Allele frequency vs INFO score
release1_HCE allele frequency
release1_HCE info score
release1 allele frequency
release1 info score
release1_GSA allele frequency
release1_GSA info score
GSA_may2021 allele frequency
GSA_may2021 info score
Release3 allele frequency
Release3 info score
Release4 allele frequency
Release4 info score

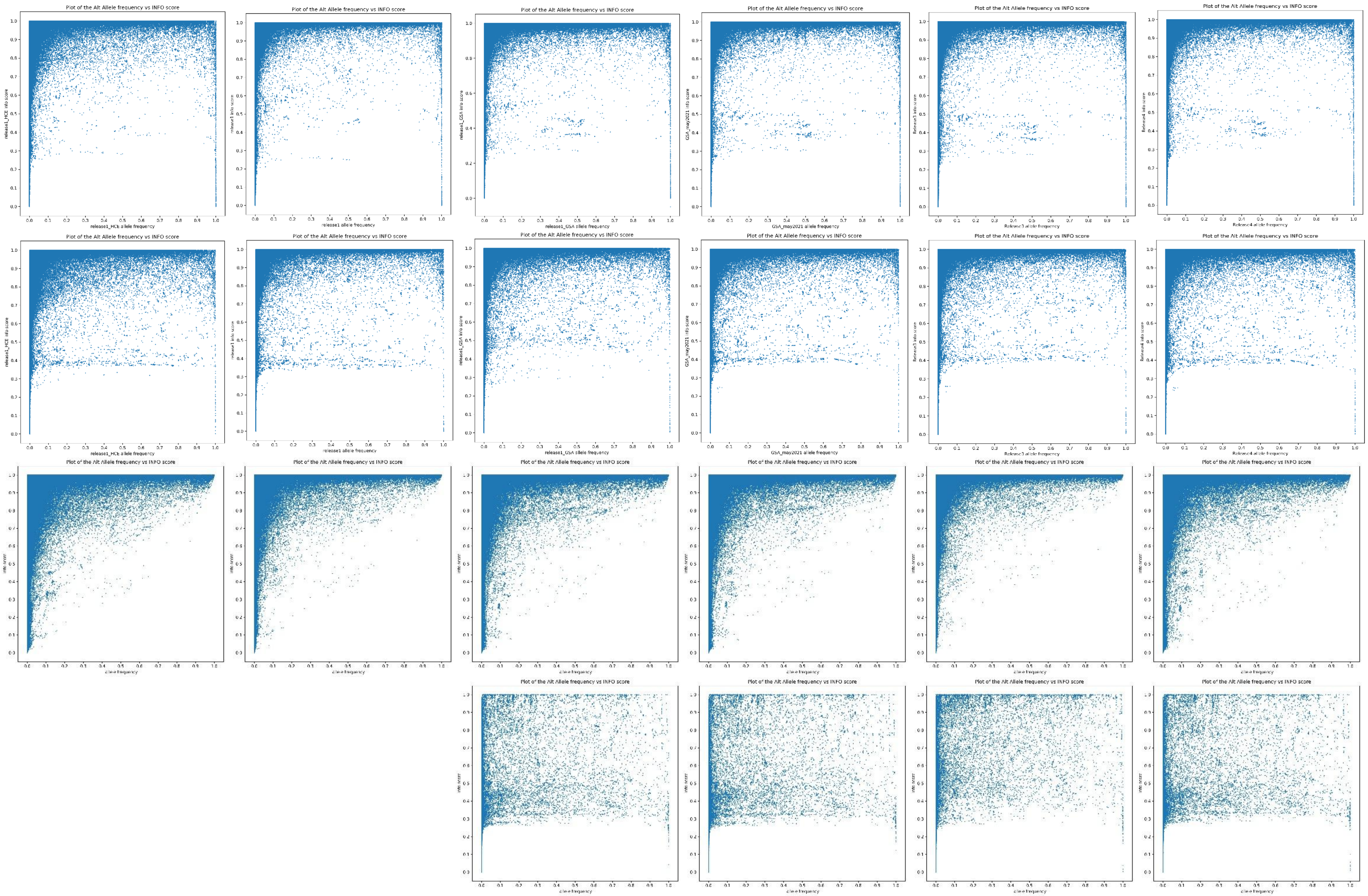
Plot of the Alt Allele frequency vs INFO score
release1_HCE allele frequency
release1_HCE info score
release1 allele frequency
release1 info score
release1_GSA allele frequency
release1_GSA info score
GSA_may2021 allele frequency
GSA_may2021 info score
Release3 allele frequency
Release3 info score
Release4 allele frequency
Release4 info score
allele frequency
info score

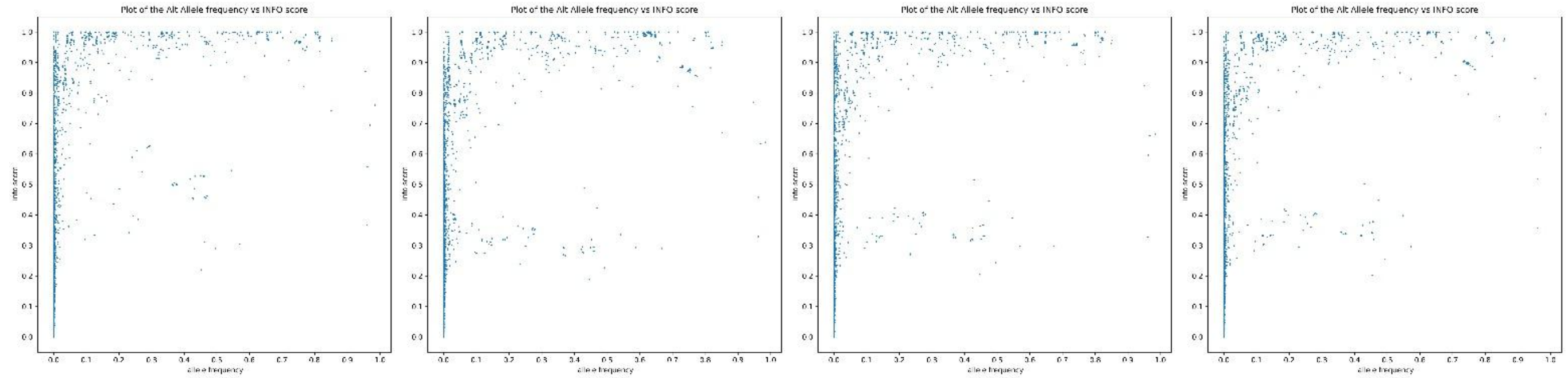

Supplementary Figure 8. Imputation quality plots showing minor allele frequency (MAF) in MoBa imputed data vs imputation quality scores (INFO) for each chromosome and MoBa imputation batch. Plots from left to right are Imputation batch HCE, OMNI, GSA1, GSA2, GSA3, and GSA4. Plots from top to bottom are chromosome 1-22 (HCE n=30,859 samples, OMNI n=26,891 samples, GSA1 n=33,893 samples, GSA2 n=62,181 samples, GSA3 n=45,624 samples, and GSA4 n=16,768 samples), chromosome X (HCE n=26,421 samples, OMNI n=26,004 samples, GSA1 n=32,289 samples, GSA2 n=60,163 samples, GSA3 n=44,139 samples, and GSA4 n=15,897 samples), and pseudoautosomal region 1 and 2 (HCE n=NA samples, OMNI n=NA samples, GSA1 n=29,213 samples, GSA2 n=60,163 samples, GSA3 n=44,139 samples, and GSA4 n=15,897 samples).

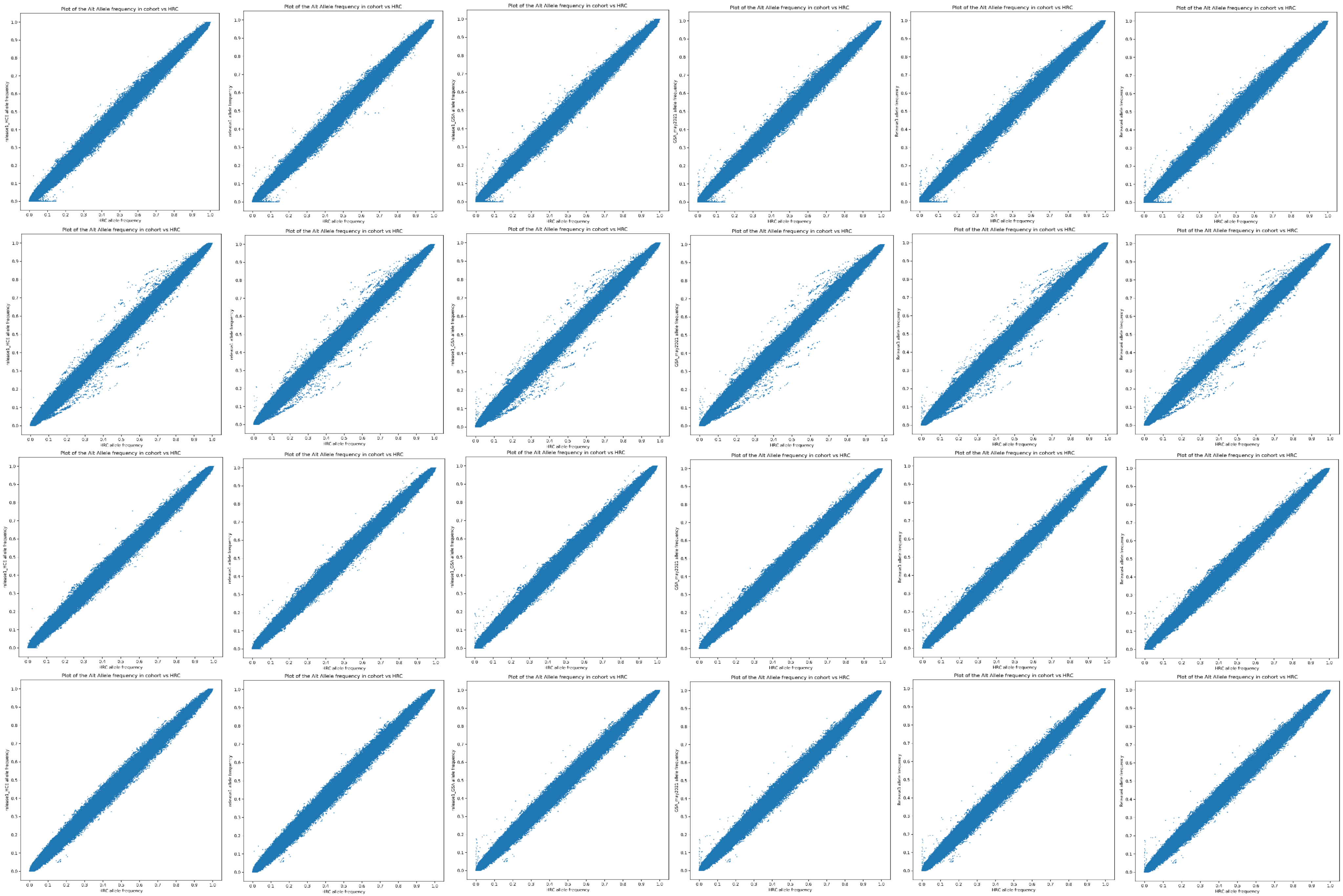
Plot of the Alt Allele frequency in cohort vs HRC
HRC allele frequency

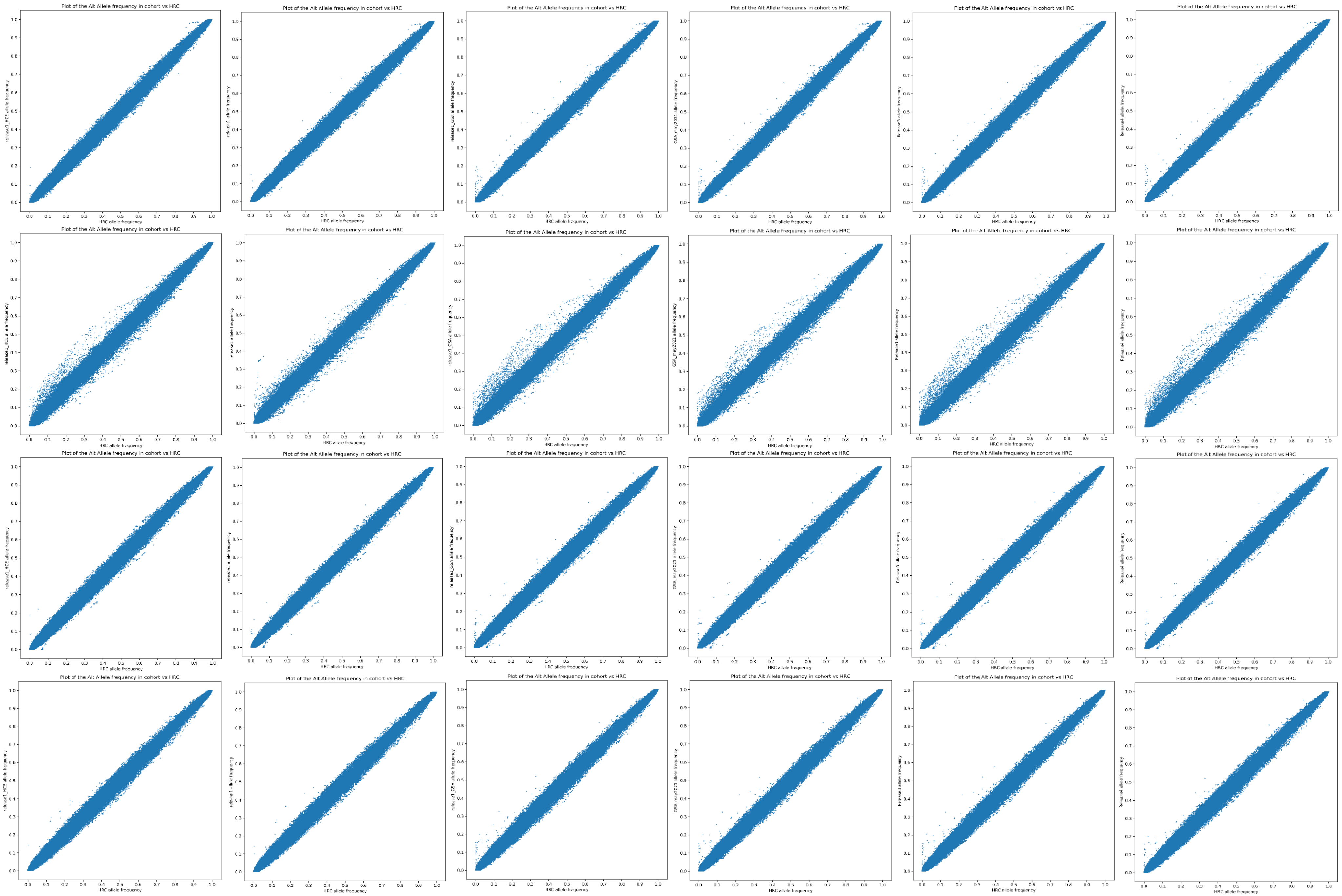
Plot of the Alt Allele frequency in cohort vs HRC
HRC allele frequency

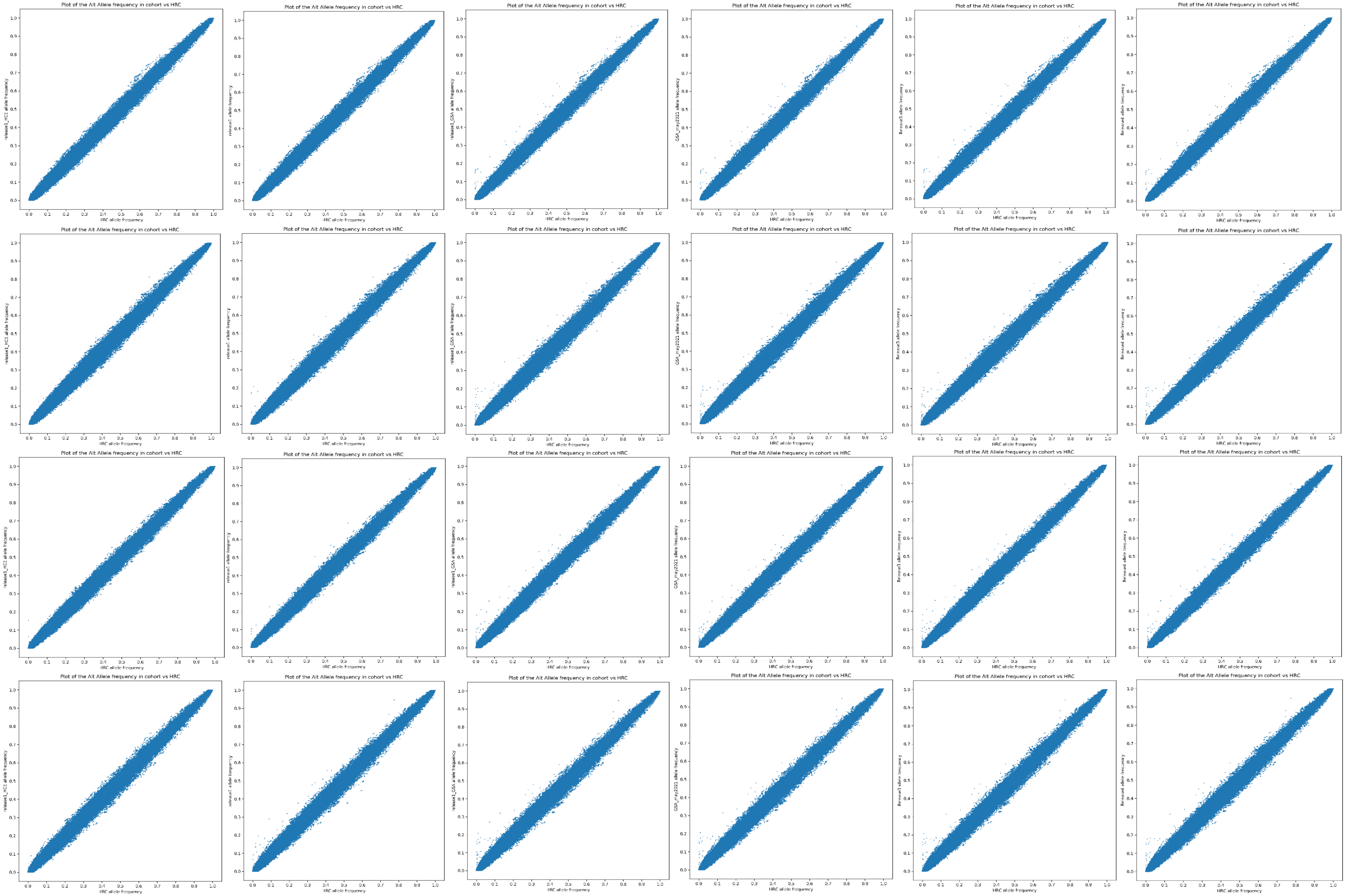
Plot of the Alt Allele frequency in cohort vs HRC
HRC allele frequency

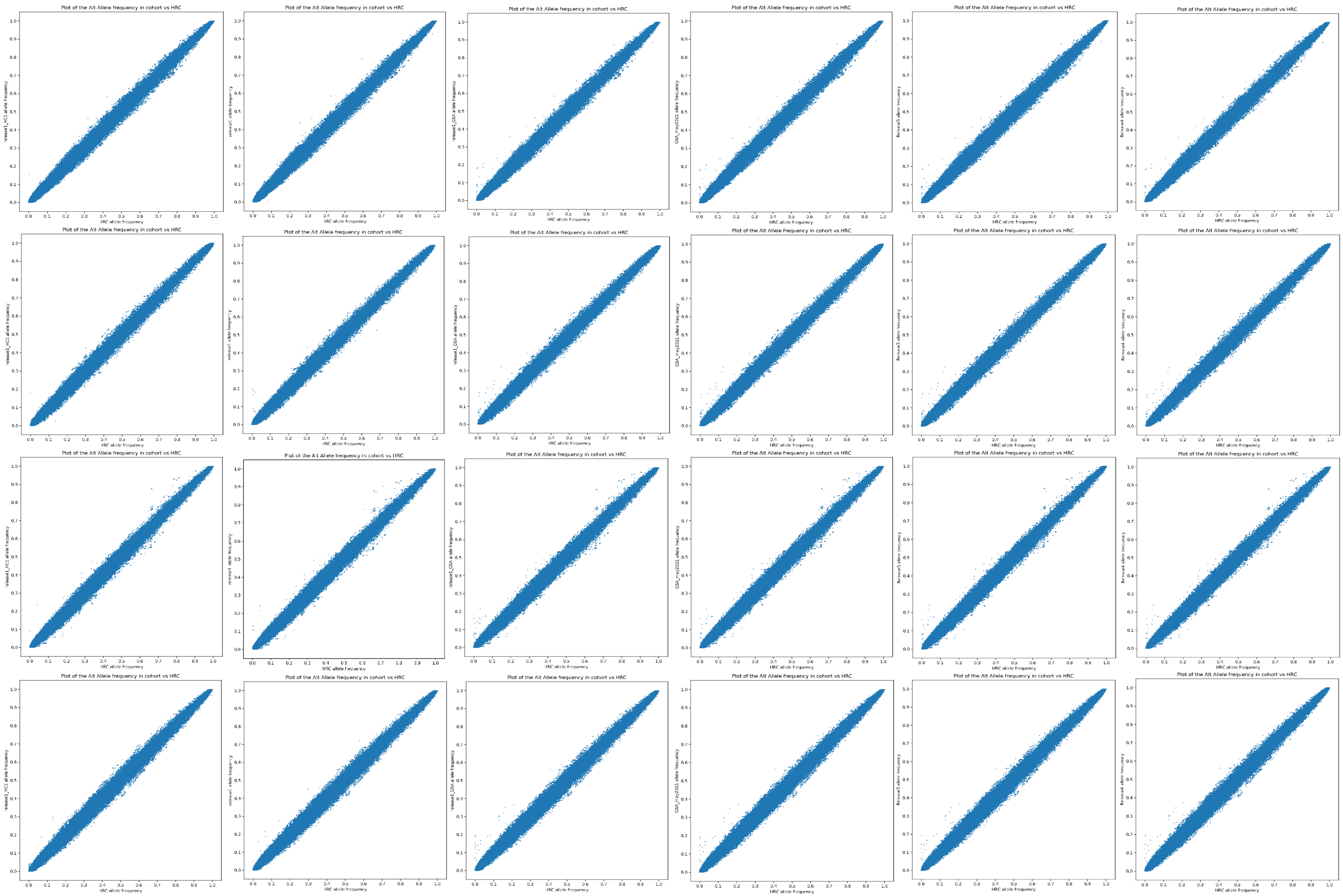
Plot of the Alt Allele frequency in cohort vs HRC
HRC allele frequency

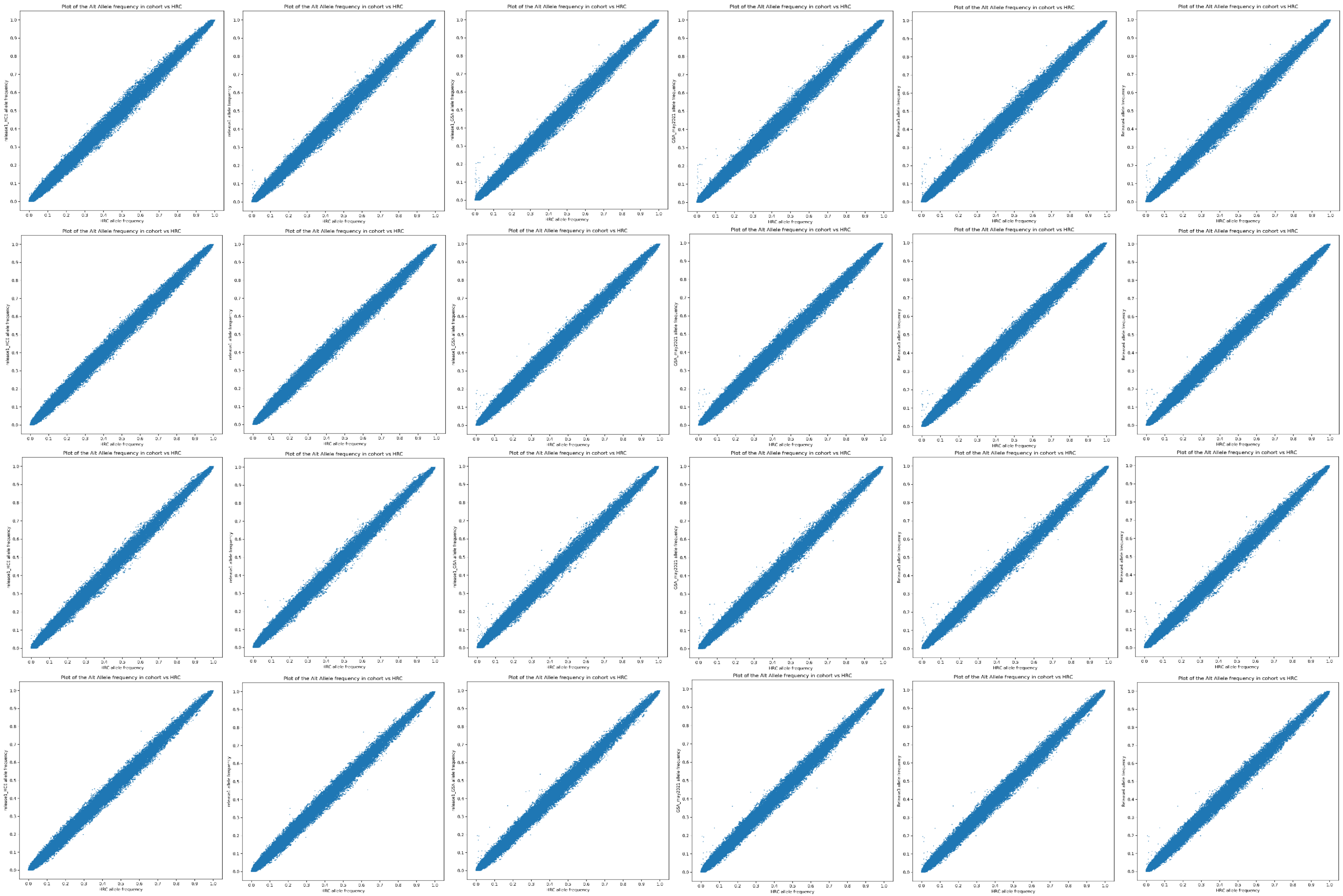
Plot of the Alt Allele frequency in cohort vs HRC
HRC allele frequency

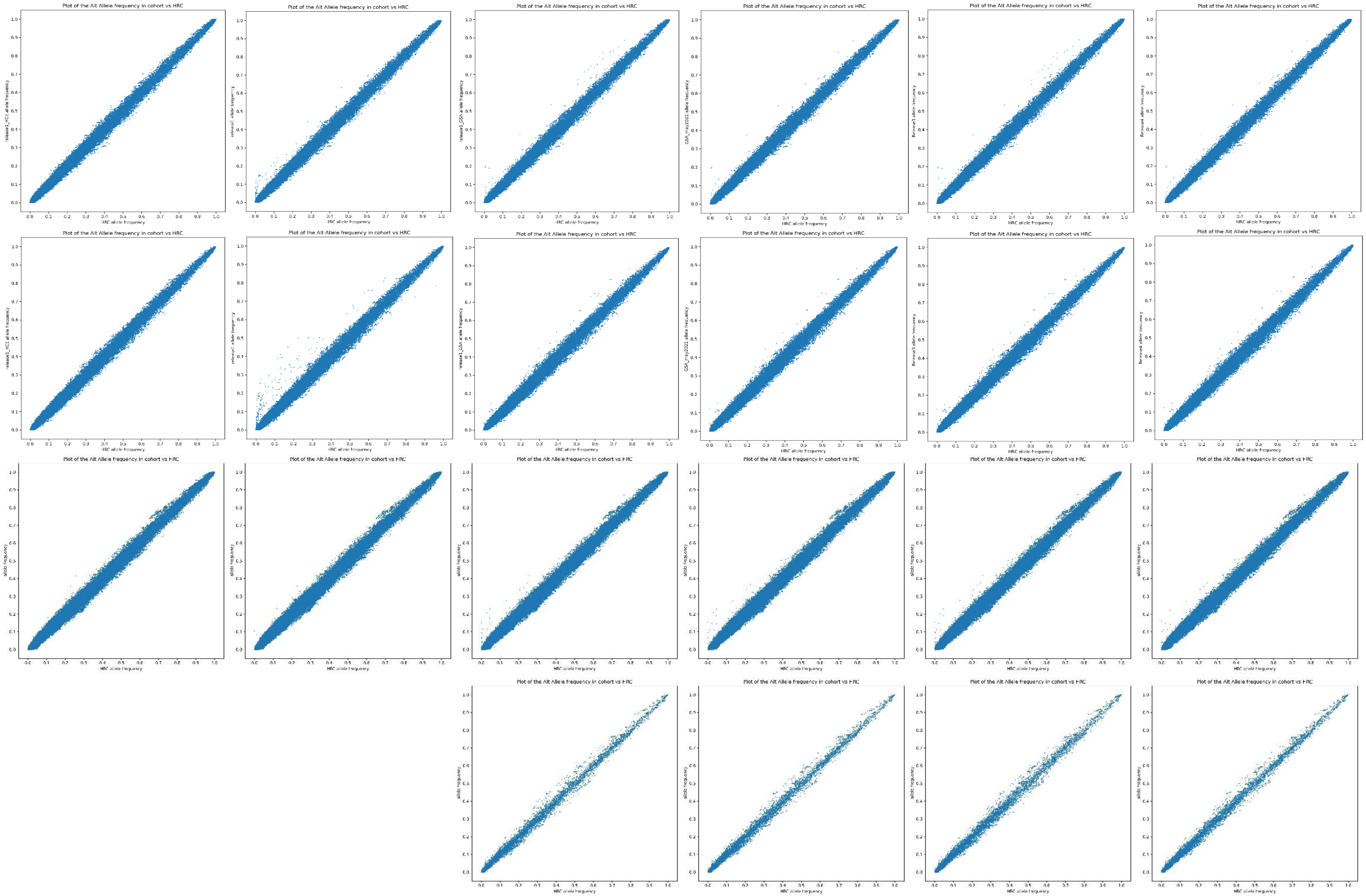
Plot of the Alt Allele frequency in cohort vs HRC
HRC allele frequency

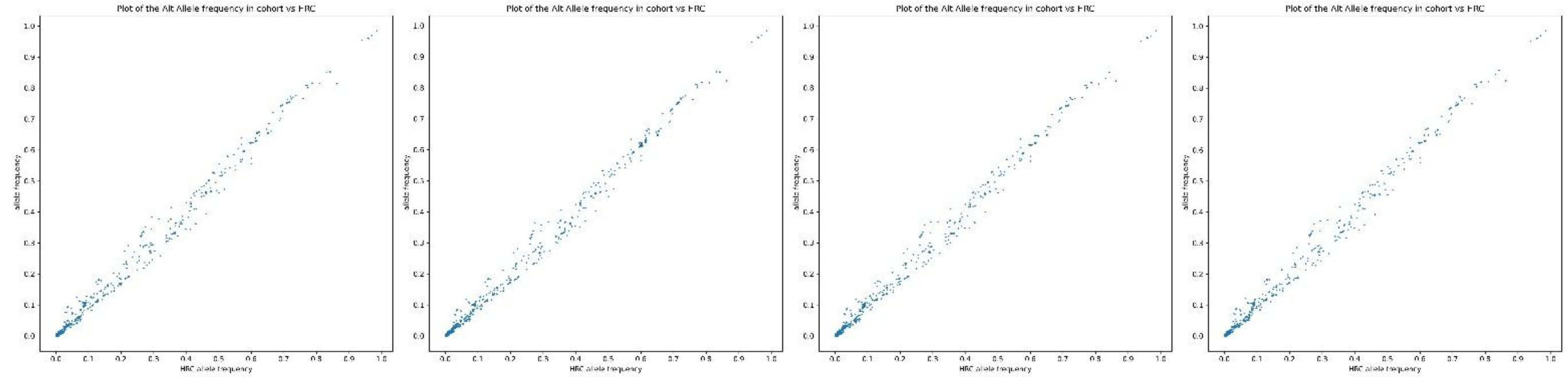

Supplementary Figure 9. Imputation quality plots showing minor allele frequency (MAF) in the Haplotype Reference Consortium vs MAF in MoBa imputed data for each chromosome and MoBa imputation batch. Plots from left to right are Imputation batch HCE, OMNI, GSA1, GSA2, GSA3, and GSA4. Plots from top to bottom are chromosome 1-22 (HCE n=30,859 samples, OMNI n=26,891 samples, GSA1 n=33,893 samples, GSA2 n=62,181 samples, GSA3 n=45,624 samples, and GSA4 n=16,768 samples), chromosome X (HCE n=26,421 samples, OMNI n=26,004 samples, GSA1 n=32,289 samples, GSA2 n=60,163 samples, GSA3 n=44,139 samples, and GSA4 n=15,897 samples), and pseudoautosomal region 1 and 2 (HCE n=NA samples, OMNI n=NA samples, GSA1 n=29,213 samples, GSA2 n=60,163 samples, GSA3 n=44,139 samples, and GSA4 n=15,897 samples).

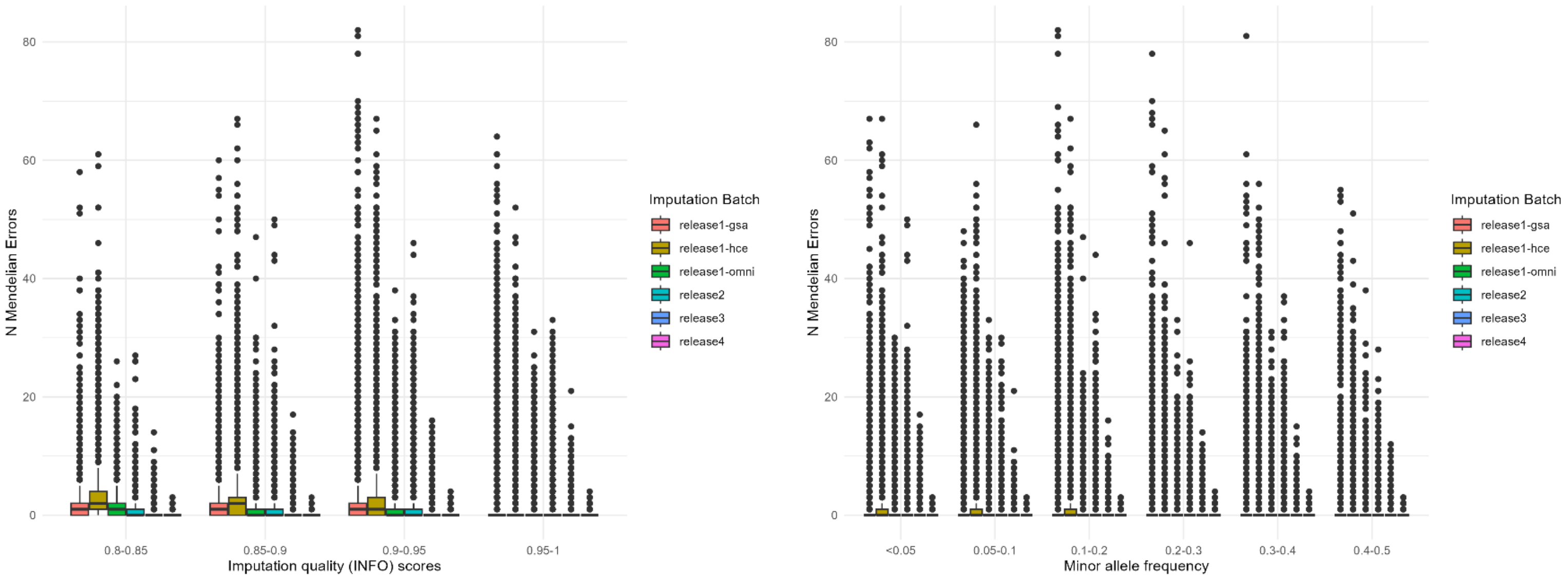

Supplementary Figure 10. Box plots showing the number of Mendelian errors (ME) in full trios for each of the 6 imputation batches. Yellow: release1-HCE n=6,748 samples, green: release1-OMNI n=5,033 samples, red: release1-GSA n=7,764 samples, cyan: release2 n=4,124 samples, blue: release3 n=1,609 samples, magenta: release4 n=230 samples. The plot on the left shows the number of ME in variants with imputation quality scores of 0.80-0.85, 0.85-0.90, 0.90-0.95, and 0.95-1. The plot on the right shows the number of ME in variants with MAF <5%, 5-10%, 10-20%, 20-30%, 30-40% and >40%.

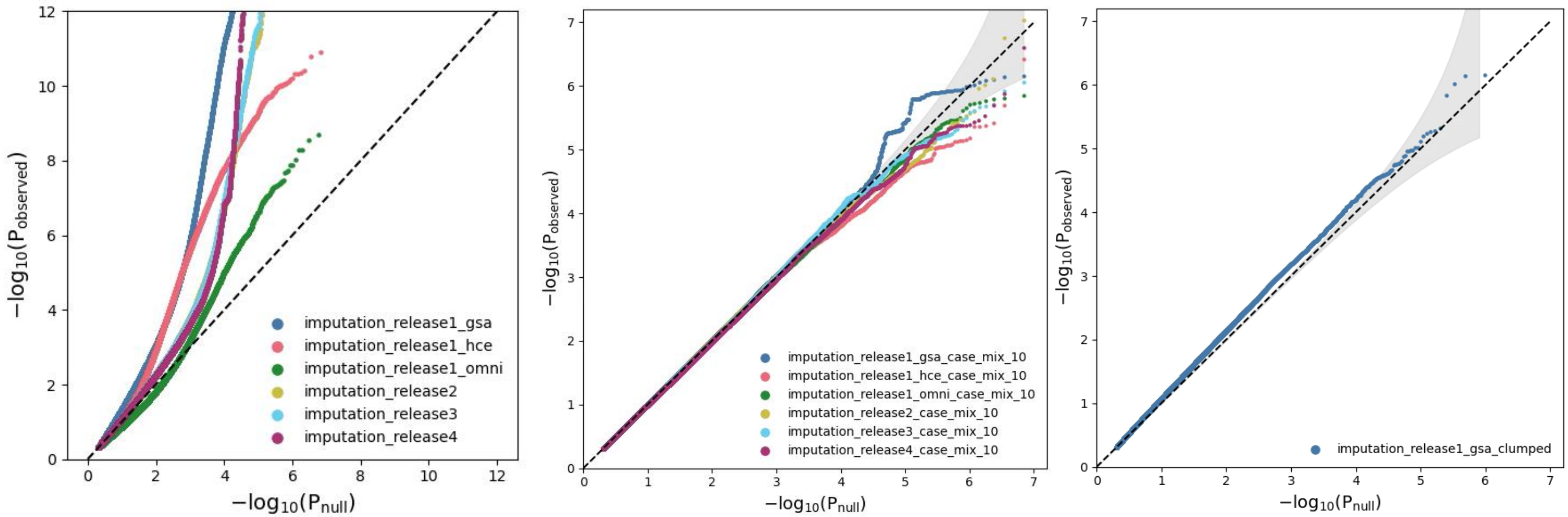

Supplementary Figure 11. Quantile-quantile (QQ) plots from six genome-wide association analyses assigning each imputation batch as cases and the remaining imputation batches as controls. The left plot shows the models without adjustment for genotyping batch (Imputation_release1_gsa n=32,535 cases & 173,423 controls, Imputation_release1_hce n=27,020 cases & 178,938 controls, Imputation_release1_omni n=26,305 cases & 179,653 controls, Imputation_release2 n=60,511cases & 145,447 controls, Imputation_release3 n=44,521 cases & 161,437 controls, imputation_release4 n=15,066 cases & 190,892 controls), while the middle plot shows the results with adjustment for genotyping batch. The right plot shows the QQ plot for the association analysis of release1-GSA imputation batch as cases adjusted for genotyping batch and pruned for linkage disequilibrium (LD) showing that the deviation from the null in the is the result of high LD. In all panels the dashed diagonal line illustrates the expected distribution of p-values under the null hypothesis of no association, while the shaded gray area shows the 95% confidence interval.
