# Supplementary material for: Family genetic designs in MoBa provide insights into health and functioning
Source: Nature. 2026 Aug 19;658(8136):707–15. doi: 10.1038/s41586-026-10926-5 (PMC13668695; doi:10.1038/s41586-026-10926-5)
Supplement: Supplementary file 4 [file 41586_2026_10926_MOESM5_ESM.pdf]

nature portfolio

Peer Review File

Family genetic designs in MoBa provide insights into human health and functioning

Corresponding Author: Professor Alexandra Havdahl

Any redactions in this file are there to maintain patient confidentiality, the confidentiality of unpublished data, or to remove third-party material.

This file contains all reviewer reports in order by version, followed by all author rebuttals in order by version.

Version 1:

Reviewer comments:

Referee #1

(Remarks to the Author)
Comment 1:
One of the strengths of MoBa, and a key message of the paper, is the advantage of using trios for genetic analysis. However, to my understanding, trios have not been used to assess the performance of the QC steps—another key aspect of the paper. I suggest that the authors evaluate the quality of imputation and QC steps by examining Mendelian violations (i.e., counting alleles that are implausible given the parental genotypes). Stratifying these violations by MAF, INFO score, etc., could provide deeper insights into the effectiveness of the QC pipeline.

Comment 2:
The reduction in heritability for height (Figure 2a) appears larger than expected compared to findings from previous studies on adult height, such as the MedRxiv preprint (https://www.medrxiv.org/content/10.1101/2024.10.01.24314703v1.full.pdf) and the Nature Genetics paper (https://www.nature.com/articles/s41588-022-01062-7). Could the authors provide an explanation for these differences?

Comment 3:
For Figure 3b, I find it challenging to interpret the impact of the polygenic score (PGS) for height on BMI. Given that BMI is a function of both height and weight, this makes it a somewhat unusual trait to highlight in this context. What is the clinically relevant question the authors aim to address here?

Comment 4:
This paper will likely become a key reference for MoBa. However, the current manuscript has limited focus on the phenotypes available in MoBa. I suggest adding a supplementary table or figure summarizing the key phenotypes in MoBa, especially highlighting those that are not available in other large biobanks such as the UK Biobank and FinnGen.

Comment 5:
The manuscript states: "The within-family SEs were on average 38% and 37% larger than the population-based SEs for height and educational achievement, respectively, indicating lower statistical power in the within-family analyses". It is not obvious that under the same sample size, why power for direct effect detection from family studies would be different. The author could add some discussion to explain this result. Was this comparison performed using the same number of children? If the only difference between the two analyses is that one includes parents and the other does not, why we should see reduction in power? See also this paper for discussion: https://www.nature.com/articles/s41586-024-07721-5

Comment 6:
Figure 2: I find that a Miami plot, which visualizes p-values, may not be the most informative way to compare trio-based versus population-based GWAS results. The trio design is specifically intended to detect direct genetic effects, and p-values from the trio GWAS are theoretically influenced by the heterozygosity rate in parents (see: https://pmc.ncbi.nlm.nih.gov/articles/PMC10680648/; https://pmc.ncbi.nlm.nih.gov/articles/PMC11008796/), which makes the comparison with population-based GWAS less straightforward. I suggest presenting the comparisons based on effect sizes

for independent loci instead of p-values. Additionally, consider including a plot to visualize the relationship between power and parental heterozygosity rate.

Comment 7:
I recommend adding a discussion about the discrepancy between the heritability estimate from LDSC and the variance component analysis from trio-GCTA. It may not be immediately obvious that LDSC is more sensitive to GWAS power than individual-level methods like GCTA.

Comment 8:
Considering the power of trio-based GWAS, I suggest exploring the method presented in https://www.nature.com/articles/s41588-022-01062-7#Sec9 to calculate effective sample sizes as input for LDSC, rather than using the raw sample size.

Additionally, there are some issues with the legend in Figure 4.

(Remarks on code availability)

Referee #2

(Remarks to the Author)
I co-reviewed this manuscript with one of the reviewers who provided the listed reports.

(Remarks on code availability)

Referee #3

(Remarks to the Author)
This manuscript describes an important innovation in the conduct of genetic association studies, perhaps especially of behavioral traits. The majority of human genetic studies were for a long time based on families. With technological advances and knowledge of linkage disequilibrium, the population genetic study became feasible and powerful. As the present paper describes, population studies have been valuable but have limitations that can be addressed at least partially with a returned focus on families.

Leading the way in this space is the cohort described here, the Norwegian Mother, Father, and Child Cohort Study. (What does MoBa stand for?) This sample is contributing in valuable ways to a wide variety of important questions that simply cannot be addressed in population datasets, including mating patterns and parsing so-called “direct” from “indirect” genetic effects. Some of these advances are illustrated in the present paper.

The manuscript is straightforward. It describes briefly the cohort, quality control procedures as they may apply to a family dataset. Then, for two example phenotypes, the authors estimate a handful of genetic estimates (e.g., heritability, polygenic score effects) for two child phenotypes (height at age 7, standardized educational test results at age 10). Then re-estimates them again in the context of an analytical design that includes the parents. I am extremely partial to the claim that large genotyped family cohorts are a new frontier with major potential in the study of medicine and human behavior. So I certainly agree with the premise of this paper. As a reviewer, I was left with several questions, which I outline here, all of which are relatively minor.

Could the authors define or characterize what are “direct” and what are “indirect” genetic effects? They have mathematical definitions in the context of the models employed here, but I would hope there is some conceptual scientific guidance available for the reader to help them understand how the math relates to the concepts. In particular, how do such analyses pin down specific causal environments? An indirect effect could be anything correlated with the parent genotypes, including stratification, parenting, occupational success, etc. Does the improved estimate of the direct effect allow one to prioritize genetic variants more or less likely to be causally interpretable?

Could the authors provide an explanation of why they find such large indirect effects, for height especially? It would seem their models assume assortative mating is zero (perhaps in addition to other assumptions). High assortative mating, expected for education and somewhat for height, would I expect result in overestimation of indirect effects and actually accidentally control for direct effects. In fact, it would seem assortative mating is an important consideration for many if not all the findings reported here. Although AM is to me a very complex issue and Norwegian scientists working in MoBa are making among the most important recent contributions (e.g., Torvik et al. 2022 Nat Comm, 1108). Also specific to the polygenic scoring work, please see: https://www.biorxiv.org/content/10.1101/2023.07.10.548458v1.full

This paragraph is quite minor and perhaps a reach and I am not requesting new analyses, but in the PTDT analyses the standardized test selection is made on the phenotypic value of a child (in the highest 90% only, with no consideration of the lowest 10%), who then is expected to have higher PGS than their mid-parent. The sibling is expected to have a PGS no

higher than the mid-parent. But if the sibling's educational attainment affects the child's, and the child is selected based on phenotype, then wouldn't one expect the sibling to have higher than mid-parent PGS as well? The effect here may admittedly be vanishingly small. Additionally, in the PTDT analyses is it possible that ascertainment and retention affects the results? If higher-achieving siblings are retained and lower-achieving siblings drop out, then aren't the siblings selected for higher educational attainment PGS, and lower smoking PGS (as they will be correlated)? Generally, some information on retention would be valuable for readers to understand this cohort and feasibility of similar efforts. On a search, I have trouble finding retention rates in MoBa other than the 2016 cohort update in Int J Epi, but I recall they are around 40-50% over a five-year follow-up.

Following references 47, 48, and 49 to understand where the polygenic scores came from I wonder if there is an error. Reference 49 did not produce smoking summary statistics. They used existing summary statistics from Liu et al. (2019) Association studies of up to 1.2 million individuals… Nature Genetics, 51, 237-244.

If space is a problem for any revisions, I would think the extensive genotype QC information could be left to supplementary materials.

No single study design is a panacea and no group of scientists is in a better position to describe limitations of family designs like MoBa than the authors of this manuscript. Could a brief discussion of strengths and limitations be provided for the curious reader? For example, parent-child designs may not be suitable for phenotypes with age of onset after mid-adulthood because parents will be censored (e.g., deceased). Perhaps more broadly, any phenotype where etiology or measurement differs as a function of age or birth year may pose problems given developmental or generational changes (e.g., years of education in parents versus standardized tests of 10-year-olds).

After reading this impressive effort, my main reaction was that I found myself wondering what the primary purpose of this manuscript was. It was a description of the cohort but may benefit from important details, such as brief descriptions of catchment, consent rates, retention, representativeness, extent of phenotyping. As an illustration of the utility of families it explores findings for only two child phenotypes, height and standardized test results, showing that estimates may differ in singleton versus trio designs, but does not really attempt to interpret these findings or characterize their scientific import in detail. A strength of MoBa are the large families, including extended families with sibling, avuncular, mating, and cousin relationships, but the utility of these is not explored. Because there are only two phenotypes, I was left wondering to what extent the family design is relevant across many more phenotypic domains. MoBa is probably the single best place to explore such issues.

Thank you for the opportunity to read and comment on this fine manuscript. I hope the above comments are somewhat helpful, I wish you the best in publishing this important work, and look forward to having this study in print.

I am in the habit of signing my reviews.

Scott Vrieze
University of Minnesota

(Remarks on code availability)

Referee #4

(Remarks to the Author)
Summary
=======
In this manuscript, Corfield et al. present the Norwegian Mother, Father, and Child Cohort Study (MoBa) genomic data processing pipeline and use the resulting data to illustrate the benefits of family-based studies for identifying direct genetic effects that may be confounded in population-based studies. With respect to the MoBa pipeline, genomic data processing pipelines addressing relatedness arising by design have already been proposed in other studies (e.g., DOI: 10.1016/j.ajhg.2015.12.001). This study's pipeline does not incorporate some state-of-the-art methods from the preceding reference or more recent ones, although the steps seemed generally reasonable. I would note that the division of the genomic data pipeline description between the Methods and Supplementary Methods made for confusing reading, with key pieces of information scattered across two locations. I would highly recommend putting all these details into the Supplementary Methods if space is an issue. There are also several points where clarification is required, including some areas of concern detailed in specific comments below.

In terms of the second major objective of this study, pointing out that large-scale family-based studies have unique utility for estimating direct genetic effects in the era of population-based biobanks is important, and the authors provide interesting examples from MoBa. However, the authors provide rather limited descriptions of the theoretical justification for each family-based approach used, and the references were not always apropos. Moreover, there are some methodological clarifications and issues in these analyses that need to be addressed detailed in the specific comments below.

Specific Comments
=================

MobaPsychGen Pipeline

- Module 0: Was conversion of A/B alleles done to obtain A, C, T, or G *on the plus strand*?
- Module 1
-- How was relatedness accounted for in HWE testing? The PCA methods seemed to account for relatedness by performing PCA in 1000 Genomes and founders only, for example.
-- For PCA, it was unclear which method was used for projection of relatives onto the PC space learned from 1000 Genomes and founders, although a modern approach would use Online Augmentation, Decomposition, and Procrustes (OADP; DOIs: 10.1093/bioinformatics/btaa152, 10.1093/bioinformatics/btaa520) to eliminate centroid bias. Please specify the method used and justify if it was not OADP or ADP.
-- Improved reference callsets, including the 1000 Genomes phase 3 callset with ~2500 individuals, have been available since 2015. Why was 1000 Genomes phase 1 used for ancestry inference?
-- The use of visual clustering for ancestry group assignment seems arbitrary. There are automated approaches to do this based on defining hyperellipsoids in the ancestry PC space (DOI: 10.1016/j.ajhg.2015.12.001), and there are also approaches that use continuous ancestry for ancestry-adjusted QC (DOI: 10.1016/j.ajhg.2015.11.022, authors' reference 15, DOI: 10.1001/jama.2023.11970), which would obviate the need to define these groups.
- Module 2
-- The authors are incorrect in stating that heterozygosity QC cannot be performed with continuous ancestry adjustment; they should see the supplement to reference 15 for details on how to adjust heterozygosity for continuous ancestry PCs. A similar approach has been used by others with diverse cohorts including family members (DOI: 10.1001/jama.2023.11970).
-- A more robust approach than KING for relatedness QC might be applying the newer PC-Relate technique (DOI: 10.1016/j.ajhg.2015.11.022) to the entire sample. This technique improves on KING for admixed samples and provides an estimate of P(IBD=0) to distinguish full siblings from parent-offspring pairs.
- Supplementary Methods 19: Were PCA and other analyses conducted again on imputed SNPs? If not, it is unclear how the checks based on PCs would reveal batch effects arising from imputation.
- General
-- What was the rationale for imputing separately by batch? This might create batch-specific variation due to variants being typed in some batches but fully imputed in others. Others have found that using the intersection of SNPs on all arrays is necessary to avoid bias in imputation (DOI: 10.1007/s00439-013-1266-7), and some studies have recommended two rounds of imputation in the context of combining array-based and WGS genotypes (DOI: 10.1038/s42003-022-03738-6).
-- For Y and MT, I can see the rationale for having completely separate descriptions of the QC pipeline. However, I would recommend including additional steps specific for the X chromosome and PAR in the description of each module for continuity. Having these separate makes it challenging to follow along with Figure 1a.
-- In Figure 1e, there seem to be many unrelated pairs with estimated IBD well above expectation. Could the authors explain this observation or perhaps remedy the overplotting to make these easier to read? Also, the methods for the IBD segment length analysis were not described, and the authors should clarify that the right panel is comparing the KING estimate of phi on the y axis to the IBD segment-based estimate of 2*phi on the x axis.

Trio-GWAS

- Supplementary Methods 23 is missing.
- Were the parent-offspring trios selected such that offspring could be considered independently sampled from a larger population? This would not be the case if, for example, a sibship with K siblings were split into K trios. If selection was done to render offspring independent of one another, the method for doing so should be described. If not, linear regression with model-based standard errors does not appropriately account for relatedness; empirical standard errors or mixed models should be used instead (as was done for Trio-PGS).
- Reference 2 uses a within-between decomposition based on sibship genotypes, which is a bit different from the Trio-GWAS approach used here. The Trio-GWAS approach is most similar in spirit to the QTDT with parental genotypes (DOI: 10.1086/302698), which was not cited, but also uses a within-between decomposition to establish its properties. Because the authors do not provide a reference that clearly describes the Trio-GWAS approach and its properties, it would be useful for them to provide a more detailed theoretical development here.

Trio-PGS

- I assume that the same model specifications from Trio-GWAS were used with the genotype terms replaced by the PGS. It would be helpful to provide explicit equations for both this and all other models so that the included covariates and effect interpretations are clear.
- The use of robust (empirical) standard errors in this analysis is entirely appropriate (see my comment in Trio-GWAS), but using only the mother ID to define clusters is probably not reasonable as it treats half-siblings with the same father as independent. More generally, the comment above about trio selection applies as there could be complex relatedness between trios drawn from the same extended family (e.g., child in one trio is a parent in another, or first cousins who do not share mother or father IDs). The safest approach would be to estimate the kinship matrix with an ancestry-adjusted approach like PC-Relate and define clusters based on groups of individuals with pairwise kinship exceeding a certain threshold. How to set this threshold would depend on whether the model was believed to capture all genetic factors contributing to phenotype (i.e., residual clustering due only to shared environment) or not; these assumptions should be specified. The authors could also consider using the approach used in the Trio-GCTA analysis to get a subset of unrelated trios.

Trio-GCTA

- Likelihood ratio tests for variance components are known to have non-standard null distributions (i.e., mixtures of chi square distributions with varying degrees of freedom) because the null is on the boundary of the parameter space. See DOI 10.2307/2289471 for the seminal paper on this topic or documentation for SAS PROC GLIMMIX available on the web. It is unclear from the manuscript or accompanying code whether the appropriate null distribution was used. The authors should specify the null distribution used for each nested likelihood ratio test.

Minor Comments

- Monozygotic twins are zeroth-degree relatives (100% IBD) rather than first-degree relatives (50% IBD).
- Please make sure to expand all acronyms at first use (e.g., MBRN).

Data Availability Statement

- Please specify the repository in which the summary statistics (presumably from Trio-GWAS) will be made available.

Data Reporting

- Figures 2a, 2b, 3b, 3d, 4b; Supplementary Figures 5, 6: Error bars were not defined (SD? SE? CI?).
- Figure 2c, Supplementary Figure 2: Reference lines were not defined (one appears to be for 5e-8 genome-wide significance).

(Remarks on code availability)
I looked through the psychgen/MoBaPsychGen-QC-pipeline and psychgen/moba-trio-analyses GitHub repositories from the perspective of someone who might want to use or adapt the code. Because I do not have access to the underlying data, I cannot test run the code to verify that I can replicate the findings presented. Both repositories are useful for providing more detailed documentation of what was done than can be provided in a manuscript, but I would find it very challenging to use the code to replicate the analysis with the same data or to adapt the code for other purposes in its current state. Specific issues include:
- The top-level READMEs do not give the user any indication of how to start using the codebase. A good example to follow is at https://github.com/PGScatalog/pgsc_calc, which gives specifics on the required execution environment, installation, testing, and running as well as links to further documentation.
- A highly motivated user could probably piece the pipeline together using the written workflow in psychgen/MoBaPsychGen-QC-pipeline/qc-modules, but the authors should provide a single shell script or nextflow pipeline for each module as well as usage instructions regarding the required execution environment, inputs, and outputs. The READMEs in scripts/{R_scripts,python_scripts} of this repository are good examples of what should be provided elsewhere for the main scripts.
- Many of the scripts are not portable and use hardcoded paths to key files that would need to be changed by a downstream user. A portable script would accept required paths and other user-specific parameters as arguments.
- Many scripts are heavily dependent on the execution environment but do not provide adequate instructions for configuring it. For example, the m1_qc.sh script above assumes that plink and R are available in PATH and does not state the versions required. A standard way to ensure a reproducible execution environment would be providing a Conda environment recipe with pinned versions/builds or using a Singularity/Apptainer image, as was done in some other scripts.
- It is unclear where to obtain required Singularity/Apptainer images or recipes to produce them.

Version 2:

Reviewer comments:

Referee #1

(Remarks to the Author)
In Figure 4a the bars for Sleep duration and depression full model variance explained seem to be a bit off.

I have no futher comments.

(Remarks on code availability)

Referee #2

(Remarks to the Author)
I co-reviewed this manuscript with one of the reviewers who provided the listed reports.

(Remarks on code availability)

Referee #3

(Remarks to the Author)
Thank you for the opportunity to read this revision. All of my positive comments from my review of the initial submission stand and I will not repeat them here.

I remain torn on the purpose of this manuscript. On the one hand it describes an exciting resource that will be of interest to a wide variety of scientists from many fields. This cannot be overstated in my opinion. But this description is fairly limited to genetic data and quality control information. Important, to be sure, but probably not of interest to the general scientist, versus the specialist. On the other hand, it describes a series of analyses that intend to "demonostrate… the vast potential of genotyped family cohorts like MoBa for refining genomic discoveries, clarifying genetic overlaps, and investigating intergenerational transmission".

Throughout the manuscript I felt like this goal was not quite achieved. The family design certainly provides exciting new avenues for investigation and analysis. But for each of the four phenotypes I was left wondering what we had learned about, say, intergenerational transmission? Maybe it is just a matter of telling the reader what they should take away from each of the analyses. My hunch is that the manuscript is currently limited by its laser focus on genetic information and analysis (perhaps because the paper began as a description of a genetic data resource), without consideration of phenotypic information and analysis crucial to interpretation of the genetic findings.

I commend the authors on a significant and responsive revision, including responding to each of the ideas that I raised in my review. I found the actual changes to the manuscript itself to be quite minimal on several points about which I remain curious.

1. The issue of assortative mating. At the very least, as a reader I would expect a discussion of AM in the methods and/or supplement, along with estimates of spousal phenotypic correlations (and, if the authors think it important, the correlation of polygenic scores) and how the models are robust (or not) to AM.

2. The measurement/etiology issue, and how it is affected by development (age) and cohort (generation). I have two suggestions.

2a. First, please add information about how the measurements were taken for the four phenotypes. Currently, one has to dig to figure out that "educational achievement" is actually GPA in fifth grade (versus years of education on which the PGS is based) and what the exam is that is used for the PDTD analysis. As another example, are the children reporting on their own sleep and depressive symptoms? It was not until page 7 (line 224) that I found it mentioned that the mother is reporting on the child's symptoms, but this is not stated elsewhere. This complicates interpretation. I recommend adding a section dedicated to describing all measures used.

2b. Second, please provide more information at the phenotypic (versus genetic) level. The genetic analysis is interesting, but without more information about the phenotypes (means and variances for children and parents [and others if relevant] pairwise relative correlations, etc.) they become quite difficult to interpret and understand. I would suggest that integrating phenotypic information directly into the current text and figures is crucial, versus punting it deep within the supplement. Or, if there is some strong scientific reason for completely divorcing the genetic and phenotypic information in this manuscript, it would be helpful to explain that to the reader.

Finally, some very minor suggestions you may consider.
3. Page 3 lines 41 to 45. This is a great definition of indirect and direct effects. On my read it tends to conflate direct effects with proximal effects and indirect effects with distal effects. For example, either could operate through environmentally- or socially-mediated pathways. (Also - do indirect effects not also routinely include population stratification.)
4. Reference 47, Karlsson-Linner did not conduct a GWAS of smoking. They used results from an existing study. I presume you then must have used results from this existing study: Liu et al. (2019) Association studies of up to 1.2 million individuals… Nature Genetics, 51, 237-244.
5. This is a minor stylistic question, but I became confused throughout by what is meant by "genetic propensities". In some places it seems to simply mean "score on the PGS", but there are some places where both terms are used in the same sentence and they seem to be used to mean different things. Is there an important distinction to be made here?
6. Consider revising the description in the main text and the legend and axis labels of Figure 3 of the PTDT method. It is difficult to follow and no interpretation is given. At the end of the paragraph (page 7 line 205) can you tell the reader why these results are useful or interesting? As it is now, the reader is left to figure that out on their own.
7. You are using three fit indices in the Trio-GCTA, and these fit indices disagree. I find that is not uncommon. If you can find a way to show the three index values for all the models you considered that I would think provides more useful information than what is currently in Figure 4b, which is a small slice of the model selection space considered here.
8. Is there inertia in the title (and abstract)? No strong opinions here but "Health and development" do not seem to be core features of the current text. Although I suppose Nature editors will make title suggestions upon acceptance.

Thank you again for considering the above.

I am in the habit of signing my reviews,

Scott Vrieze
University of Minnesota

(Remarks on code availability)
I commend the authors for posting their code and encourage them if they haven not already to making sure the code runs, perhaps with example toy datasets.

Version 3:

Reviewer comments:

Referee #3

(Remarks to the Author)
This is a third review of this manuscript. I very much appreciate the care with which the authors have continued to humor my suggestions. Thank you kindly. I apologize for the delay in providing this review.

I have no new suggestions or concerns. I continue to believe that the addition of information about phenotypic similarity among relatives is crucial to interpret the genetic analyses. But I do not see it as my role as reviewer to insist on such a thing so will decline any further review request related to this issue.

Many thanks for your work on this important research enterprise. I am convinced it will fuel important work in multiple disciplines, including medicine, psychology, human genetics, and methodological development.

I am in the habit of signing my reviews.

Collegially,

Scott Vrieze
University of Minnesota

(Remarks on code availability)
I have not reviewed the code base.

Open Access This Peer Review File is licensed under a Creative Commons Attribution 4.0 International License, which permits use, sharing, adaptation, distribution and reproduction in any medium or format, as long as you give appropriate credit to the original author(s) and the source, provide a link to the Creative Commons license, and indicate if changes were made.
In cases where reviewers are anonymous, credit should be given to 'Anonymous Referee' and the source.
The images or other third party material in this Peer Review File are included in the article’s Creative Commons license, unless indicated otherwise in a credit line to the material. If material is not included in the article’s Creative Commons license and your intended use is not permitted by statutory regulation or exceeds the permitted use, you will need to obtain permission directly from the copyright holder.
To view a copy of this license, visit https://creativecommons.org/licenses/by/4.0/

Editor comments:

1. You will see that there's generally appreciation for the study and its potential value, however, the reviewers have also pointed out that this value has not been sufficiently *demonstrated*. We ask that in a revised manuscript you include further vignettes (along the lines suggested by reviewers) that shows how this dataset can be used for answering questions that could previously not be addressed.

Response: In line with your guidance, we have included additional vignettes to demonstrate how the MoBa genomic resource can be used to address questions that could not be previously answered. Specifically, we have revised the manuscript to clarify its twin aims of a) illustrating the importance of genotyped family cohort data for progressing genomic research and b) highlighting and exemplifying the MoBa cohort as a valuable resource in this regard. Furthermore, we have included an overview of the extensive phenotyping of family members, spanning more than 25 years of ongoing data collection. We have expanded the vignettes to illustrate how trio designs can offer novel insights into diverse traits, including height, educational achievement, depressive symptoms, and sleep duration. We selected the additional traits to exemplify the breadth of phenotyping in MoBa, including behavioral traits not available in many biobanks with mostly binary diagnostic data. This selection of vignettes achieves a greater breadth without compromising the high-level, illustrative nature of the analyses. Going either too broad or too deep with our analyses and interpretations would undermine the accessibility of the paper for its target audience. We hope the changes suitably address the nuanced point raised and demonstrate the value of MoBa as a family-based genomic resource for advancing our understanding of within-family transmission of human traits. These changes are detailed below in our response to reviewers.

2. We further ask that the data availability statement is made more detailed, and also states if any restrictions are in place (i.e. are the data for general research use?).

Response: We have added detail to the data availability statement, as shown below. The MoBa data is for general research use.
"Data from the Norwegian Mother, Father and Child Cohort Study (MoBa) is managed by the Norwegian Institute of Public Health. The MoBa website provides details on the available data and how to obtain access for research (https://www.fhi.no/en/ch/studies/moba/for-forskere-artikler/research-and-data-access/). Access to individual-level data from MoBa and national registries requires approval from the Regional Committees for Medical and Health Research Ethics (REK), compliance with General Data Protection Regulation (GDPR), and data holder approval. Researchers must apply via https://helsedata.no/en/. International researchers need to collaborate with a principal investigator employed at a Norwegian research institution - the MoBa administration team (mobaadm@fhi.no) can facilitate contact with relevant research environments. Information on how to access the MoBaPsychGen post-imputation quality controlled data is available here: https://www.fhi.no/en/more/research-centres/psychgen/access-to-genetic-data-after-quality-control-by-the-mobapsychgen-pipeline-v/. All summary statistics from genome-wide association studies (GWASs) conducted in this study will be available on the MoBa website for GWAS summary statistics: https://www.fhi.no/en/ch/studies/moba/for-forskere-artikler/gwas-data-from-moba/. Participant consent does not allow individual-level data storage in repositories or journals."

3. Finally, we ask that the manuscript title is revised to make it more informative.

Response: We have revised the title to make it more informative: Family genetic designs in the MoBa cohort provide insights into human health and development.

Response to the reviewers' comments:

Referee #1: genetic epidemiology, biobanks

1. One of the strengths of MoBa, and a key message of the paper, is the advantage of using trios for genetic analysis. However, to my understanding, trios have not been used to assess the performance of the QC steps—another key aspect of the paper. I suggest that the authors evaluate the quality of imputation and QC steps by examining Mendelian violations (i.e., counting alleles that are implausible given the parental genotypes). Stratifying these violations by MAF, INFO score, etc., could provide deeper insights into the effectiveness of the QC pipeline.

Response: We have now examined the number of Mendelian errors within full trios in each imputation batch stratified by imputation quality score (INFO) and minor allele frequency (MAF). The results of this investigation are now included in Supplementary Methods 15 – Assessment of ME within full trios, which states: "Within each imputation batch, we used full trios (release1-HCE=6,748, release1-OMNI=5,033, release1-GSA=7,764, release2=4,124, release3=1,609, release4=230) to assess the QC by stratifying the number of ME by INFO score and MAF in the SNPs passing post-imputation QC. As seen in Supplementary Figure 18 for SNPs passing post-imputation QC, the pattern of ME is similar across all INFO and MAF thresholds within each imputation batch, proportional to the number of full trios within the batch. The HCE imputation batch exhibits slightly elevated numbers, as expected due to the lower number of common genotyped SNPs.

2. The reduction in heritability for height (Figure 2a) appears larger than expected compared to findings from previous studies on adult height, such as the MedRxiv preprint (https://www.medrxiv.org/content/10.1101/2024.10.01.24314703v1.full.pdf) and the Nature Genetics paper (https://www.nature.com/articles/s41588-022-01062-7). Could the authors provide an explanation for these differences?

Response: We thank the reviewer for this helpful observation and for their other suggestion (comment 8) to calculate effective sample sizes for input into LDSC. In our initial submission, we had used the total offspring sample size, which can bias within-family SNP-heritability downward. This is because, in within-family GWAS, the effective sample size for each SNP is lower relative to the raw total number of offspring, depending on parental heterozygosity. Following the reviewer's advice, we computed per-SNP effective sample sizes and re-ran the LDSC analyses.

With this correction, the discrepancy between the population-based and within-family (trios-based) heritability estimates for height is now more consistent with previous reports (e.g. in the Nature Genetics paper by Howe et al., 2022[1]: population h^2_{SNP}=41%; within-sibship h^2_{SNP}=34%). The updated results are now included in the main manuscript text (page 4 line 108-115), which states "In the population models, SNP-based heritability (h^2_{SNP}) for height and educational achievement was h^2_{SNP}=35% (standard error; SE=3%) and h^2_{SNP}=31% (SE=2%), respectively. Estimates were lower for sleep duration (h^2_{SNP}=5%, SE=2%) and depressive symptoms (h^2_{SNP}=6%, SE=3%). In the trio models, which conditioned the effects of children's genotype on parental genotypes, we saw the heritability estimates for height and education attenuate to h^2_{SNP}=22% (5%) and h^2_{SNP}=24% (3%), respectively (Figure 2a). Heritability for sleep duration was reduced to near zero within-families (h^2_{SNP}=1%, SE=4%), while the within-family estimate for depressive symptoms was higher (h^2_{SNP}=11%, SE=5%).".

3. For Figure 3b, I find it challenging to interpret the impact of the polygenic score (PGS) for height on BMI. Given that BMI is a function of both height and weight, this makes it a somewhat unusual trait to highlight in this context. What is the clinically relevant question the authors aim to address here?

Response: Our findings indicate that when parental genotypes are not accounted for, this can lead to an underestimation of the association between children's genetic predisposition to higher BMI and

their height, illustrating a suppression effect. Our intention was to demonstrate that neglecting to account for parental genotypes can lead to both inflated and reduced effect estimates. However, we acknowledge that the relationship between height and BMI is atypical, which may detract from the clarity of our argument. In response to your comments, we have added two additional traits to provide a broader context for the utility of within-family designs in the MoBa genomic resource. We now highlight a clearer example of a suppression effect: the impact of maternal educational attainment genetic propensity on depressive symptoms.

Amended text on page 6, line 175-183: "However, there were notable examples of parental genetic propensities *suppressing* associations between children's genetic propensities and their traits. For instance, the association between children's genetic propensity to educational attainment and their depressive symptoms more than doubled after adjustment for parental genotypes ($\beta_{unadjusted}$=-0.03, 95% CI: -0.04,-0.02; $\beta_{adjusted}$=-0.06, 95% CI: -0.08,-0.04; Figure 3b**,** right-most panel). Figure 3c (bottom panel) illustrates that this suppression effect, as well as the 130% inflation of the association between child genetic propensity to depression and their own depressive symptoms in unadjusted models, were attributable to maternal, but not paternal, genetic effects."

4. This paper will likely become a key reference for MoBa. However, the current manuscript has limited focus on the phenotypes available in MoBa. I suggest adding a supplementary table or figure summarizing the key phenotypes in MoBa, especially highlighting those that are not available in other large biobanks such as the UK Biobank and FinnGen.

Response: Thank you, we have followed your useful suggestion and added a figure providing an overview of the extensive longitudinal phenotyping in MoBa, including a wide array of behavioral and environmental measures not available in other large biobanks such as the UKBB and FinnGen. Accompanying text in the updated first section (page 4, lines 77-100) of the manuscript (which previously focused more on the QC for the genetic data, now in the supplement), entitled "MoBa: a family-based genetic resource with extensive longitudinal phenotyping", reads: "MoBa represents a uniquely powerful resource for family-based genetic analyses, offering a wide range of phenotypic measures on mothers, fathers, and offspring collected from early pregnancy and spanning more than 25 years. Within the completed data collections covering the first fourteen years of life alone, MoBa has amassed observations on neurodevelopment, mental health, physical health, health behaviours and medication use, social and environmental exposures and more (Figure 1). Ongoing waves continue to follow participants through adolescence and into early adulthood, while numerous sub-studies have collected diverse biological samples and additional phenotypes, further expanding the potential for deriving insights into human health and development using family-based genetic analyses[2]. This potential is underpinned by the availability of genotype data collected on 77,634 mothers, 53,358 fathers, and 76,577 children in MoBa. The considerable number of variously interrelated families in the sample, combined with complexities in the way genotyping was carried out necessitated the implementation of a quality control (QC) pipeline bespoke to MoBa. Therefore, we developed and applied a family-based QC and imputation pipeline that verifies and accounts for diverse types of relatedness from large population-based samples of individuals while appropriately handling the differences resulting from a varied genotyping strategy (Supplementary Note 1). The relationships identified during the QC (Supplementary Note 2) included 287 monozygotic twin pairs, 22,884 full-siblings, 117,006 parent-offspring pairs, 23,299 second-degree relative pairs, and 10,828 third-degree relative pairs. This included 44,017 full father-mother-child trios, which form the basis for refined genomic discovery and enhanced downstream genetic analyses in the sample.".

5. The manuscript states: "The within-family SEs were on average 38% and 37% larger than the population-based SEs for height and educational achievement, respectively, indicating lower statistical power in the within-family analyses". It is not obvious that under the same sample size, why power for direct effect detection from family studies would be different. The author could add some discussion to explain this result. Was this comparison performed using the same number of children? If the only difference between the two analyses is that one includes

parents and the other does not, why we should see reduction in power? See also this paper for discussion: https://www.nature.com/articles/s41586-024-07721-5

Response: Thank you for suggesting adding an explanation for the difference in power. The population-based estimate reflects the total association of the child's genotype and the phenotype. This includes direct and all indirect sources of association. Whereas, in the within-family estimate, which conditions on the parental genotypes, we estimate only the direct effect of the child's genotype on the phenotype, excluding most indirect paths. Children and parents' genotypes are highly correlated (0.5), and hence the conditional variation in the child genotype is much lower. This means that the within-family SEs will almost certainly be higher for the same sample size. Intuitively, each parent's genotype correlates 0.5 with the child's genotype and will independently explain 25% of the variance in the child's genotype. Jointly the parents' genotype will explain 50% of the variance in the children's genotype. Thus, the conditional variance in the children's genotypes will be 50% smaller. This implies a theoretical Variance Inflation Factor of $VIF = \frac{1}{(1-R^2)} = 2$, and an inflation of the standard errors of $SE\ inflation = \sqrt{2} = 1.414$, which is approximately what we see empirically.

In Davies et al. (2024)[3], we made a subtly different argument, that there was very modest loss in power for estimating population-based associations using samples of trios, if the phenotype is measured in all individuals (children, mothers and fathers). Whereas, we noted that within-family estimates of direct effects are substantially less precise than population-based estimates of the total association. However, the two analyses (population-based and within-family) are estimating two different parameters, the total child genotype-phenotype association and the direct effect of the child's genotype on the phenotype.

In this revision, we additionally present the average percentage increase in SEs for sleep duration and depressive symptoms, with average increase across all four phenotypes examined ranging from 37-39% (Page 5, line 150-153): "The within-family SEs were on average 38% larger than the population-based SEs across traits (height: 38%, education achievement: 37%, sleep duration: 38%, depression: 39%), indicating lower statistical power in the within-family analyses."

6. Figure 2: I find that a Miami plot, which visualizes p-values, may not be the most informative way to compare trio-based versus population-based GWAS results. The trio design is specifically intended to detect direct genetic effects, and p-values from the trio GWAS are theoretically influenced by the heterozygosity rate in parents (see: https://pmc.ncbi.nlm.nih.gov/articles/PMC10680648/;https://pmc.ncbi.nlm.nih.gov/articles/PMC11008796/), which makes the comparison with population-based GWAS less straightforward. I suggest presenting the comparisons based on effect sizes for independent loci instead of p-values. Additionally, consider including a plot to visualize the relationship between power and parental heterozygosity rate.

Response: With the addition of two further vignettes in the analyses in this revision, we have opted to move the Miami plots to the supplement and present only the comparison SNP heritabilities and rGs from the population and trio-based GWAS in Figure 2. This is because we do agree, in part, that the Miami plot was not the most informative part of the figure. However, we have retained these plots in the supplement and would like to elaborate on our reasoning for using this plot type for comparing the two sets of GWAS results.

Veller et al. (2024)[4] raise an interesting point about the interpretation of estimates from within-family studies. They make two arguments: first, that if there is effect heterogeneity, then within-family estimates represent a local average treatment effect (LATE), borrowing terminology from the instrumental variables literature in econometrics. Second, the LATE is the effect of the exposure in individuals whose exposure value was altered by the instrument – the effect among compliers. For example, in a randomised controlled trial with non-compliance, there are four types of individuals:

those who comply with treatment (i.e., take the treatment they are allocated to), those who never take treatment (never-takers), those who always take treatment (always-takers), and those who defy the treatment (defiers). Studies typically assume that there are no defiers, and thus, the LATE reflects the effect among the compliers (the only group whose exposure is affected by the instrument). When applied to genetic inheritance, this implies that within-family estimates reflect the effects of inheriting an allele in those with at least one heterozygous parent. Individuals with parents who were homozygous at that locus would always inherit the same variants; they would have no variation in their exposure and thus not contribute to the estimated LATE (they would be never takers or always takers). If the effects of the variant differ between those with homozygous and heterozygous parents, then the LATE will not equal the average treatment effect. This is theoretically interesting, however as noted by Veller et al. (2024)[4] there is currently little evidence that the effects of variants do differ between the offspring of homozygous and heterozygous parents:

"The consequence is that if there is heterogeneity in effects between the children of homozygous and heterozygous parents, family studies will generally result in a biased estimate of the average effect of an allele in a population. In the case of the effect size estimated by a family GWAS for a single locus, the estimate can nonetheless be viewed as a LATE for the children of heterozygotes, and thus has internal validity for a well-defined subset of families."

Furthermore, this limitation applies to population-based estimates as well, which in addition can be confounded. Therefore, within-family estimates are likely to produce a less biased estimate of the direct causal effect than population-based estimates, because while both may be biased by hypothetical effect heterogeneity, population-based estimates can also be biased by confounding.

Veller and Coop (2024)[5] argue that cis-linkage disequilibrium (LD) within a chromosome or haplotype, and trans-LD across parental haplotypes can induce bias into within-family estimates and that estimates of direct effects from siblings can be biased by indirect effects of the siblings. Variants in cis and trans-LD with the locus are a potential source of bias in the direct effect of each locus; however, this bias also applies to population-based estimates, which must, in addition, overcome the confounding factors outlined in Veller and Coop (2024)[5] and many other papers. Furthermore, Veller and Coop (2024)[5] provide little indication of the extent or impact of these hypothetical biases, or compelling empirical examples to demonstrate that these biases are likely to meaningfully impact empirical results. Regarding sibling indirect effects, we present estimates from a trio model, which will not be affected by bias, as, conditional on parental genotype, each sibling's genetic inheritance will be independent of the variants inherited by their sibling.

Finally, regarding the relationship between power and parental heterozygosity rate, we have followed your suggestion to illustrate the relationship between power and parental heterozygosity rate (new Supplementary Figure 10, referenced in the Methods section on page 19, line 534). Below we provide two-by-two scatter plots illustrating the relationship between the minor allele frequency in the parents (proxied by offspring MAF, which by definition will equal the MAF in the parents on average) and the standard error of the population-based and within-family estimates.

Height

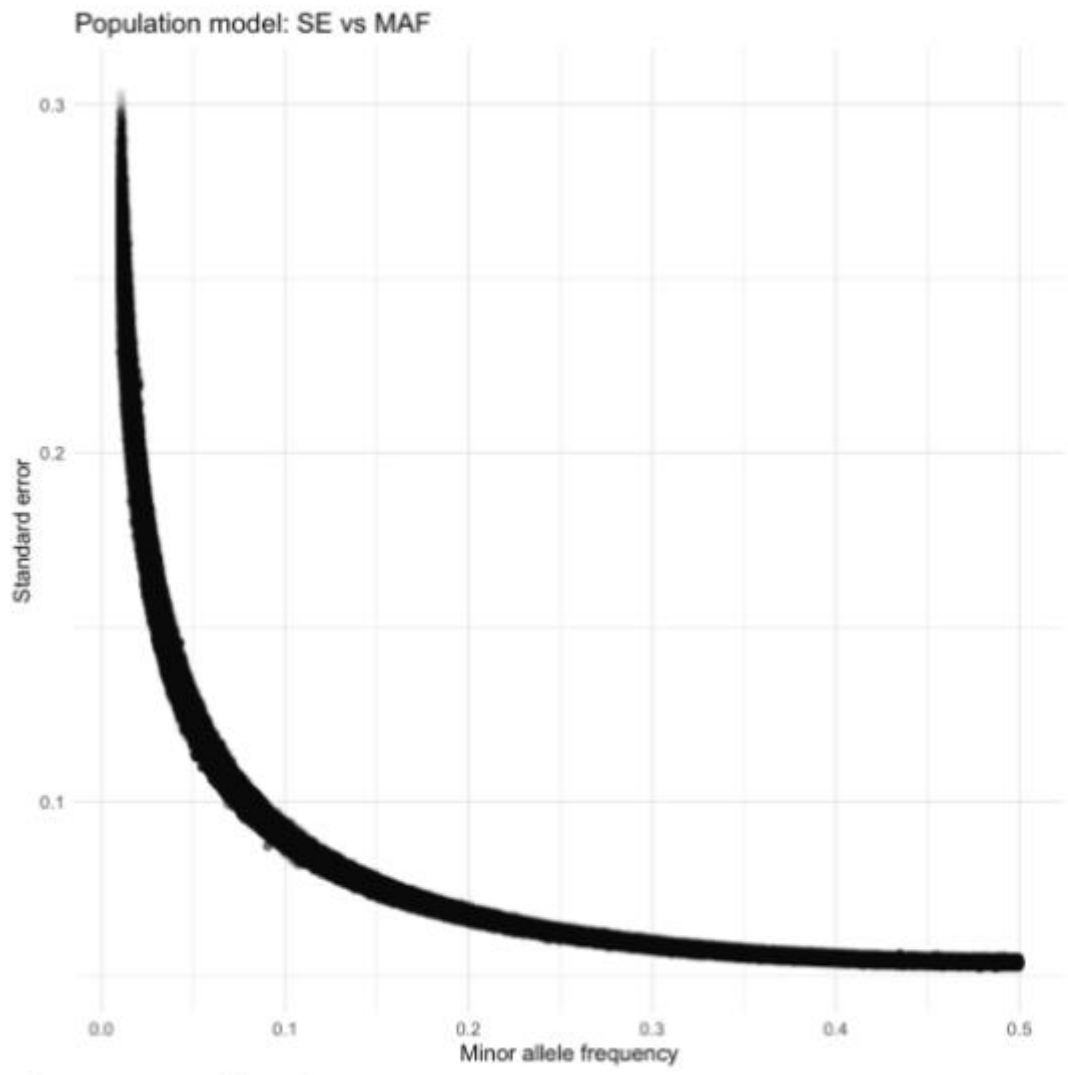

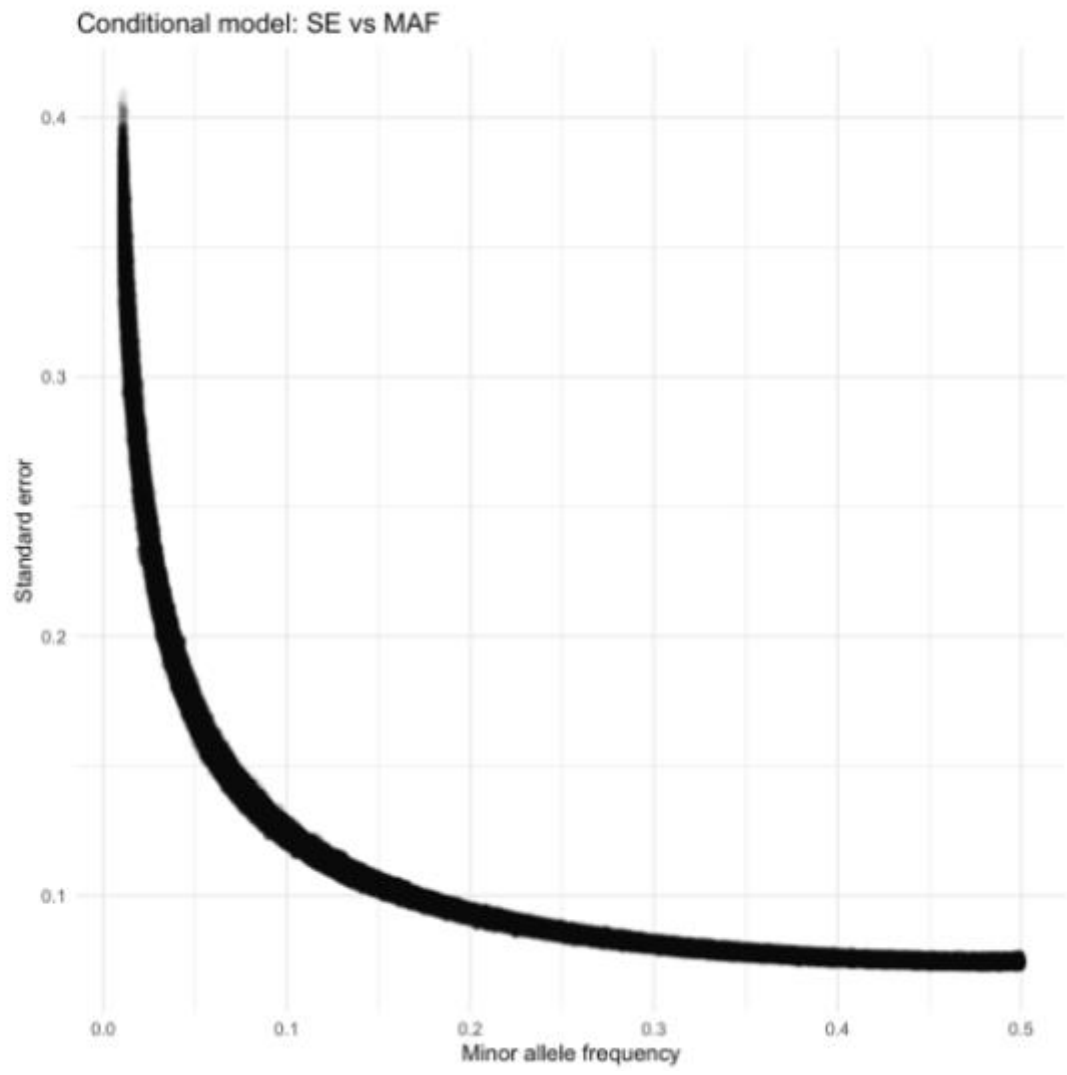

Educational achievement

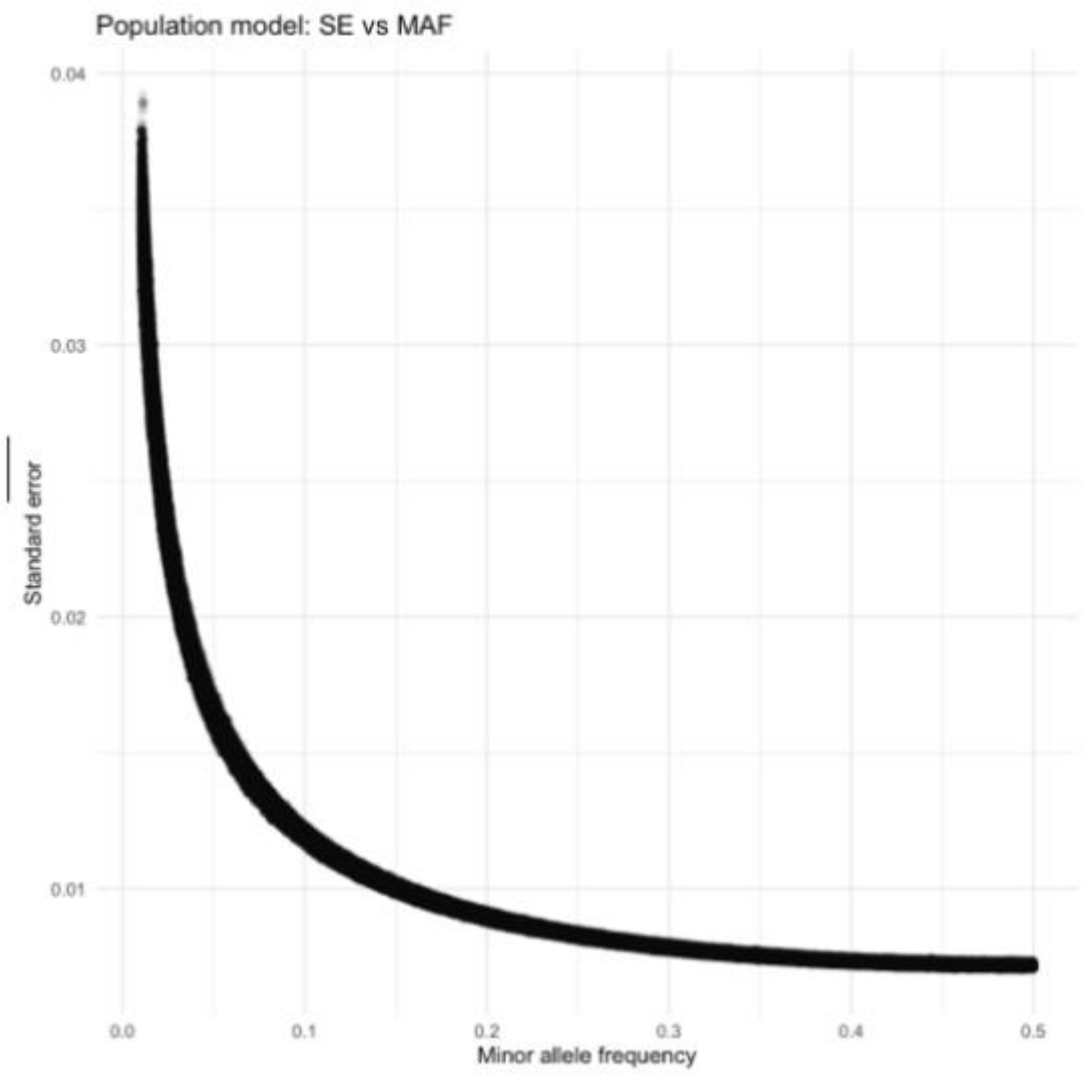

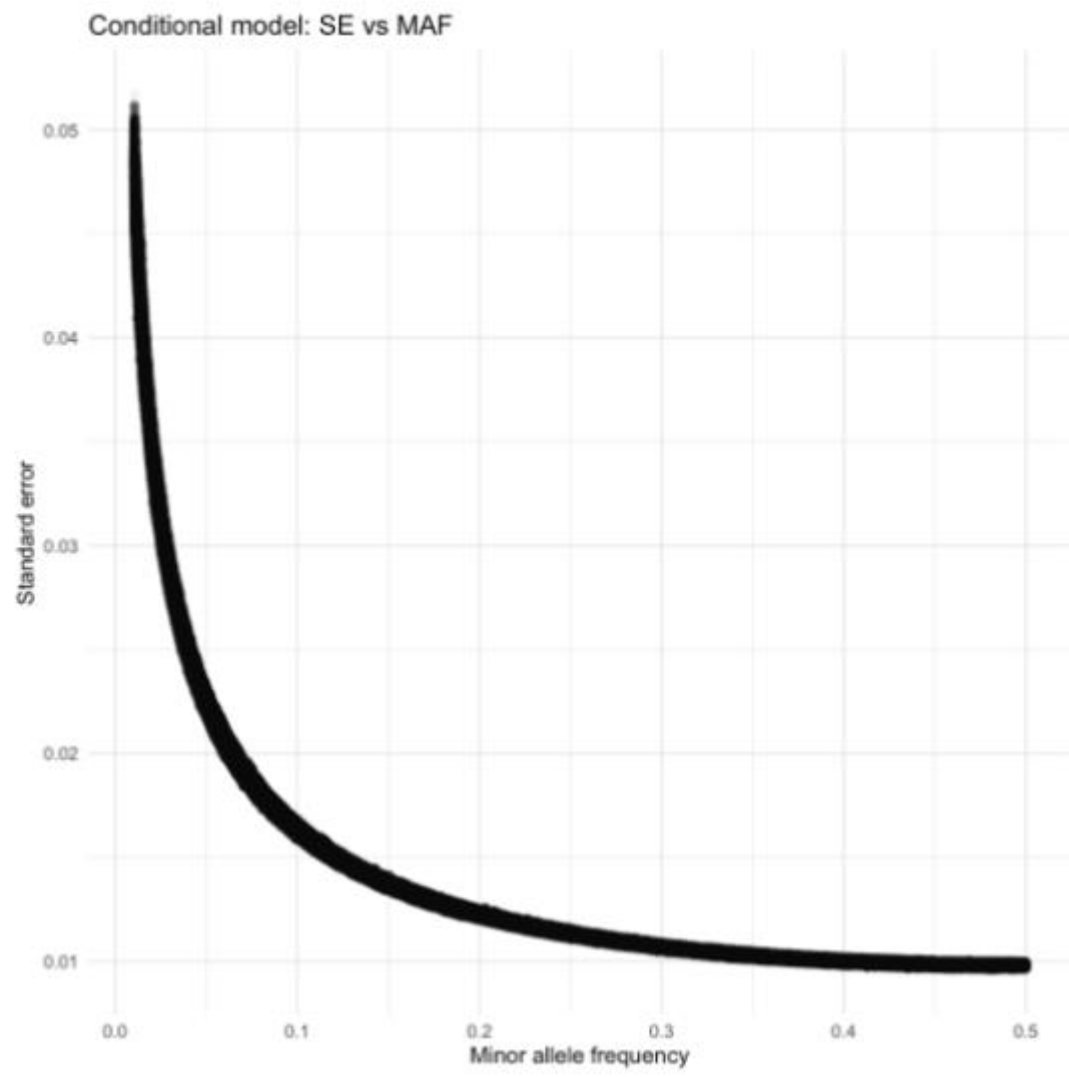

Sleep duration

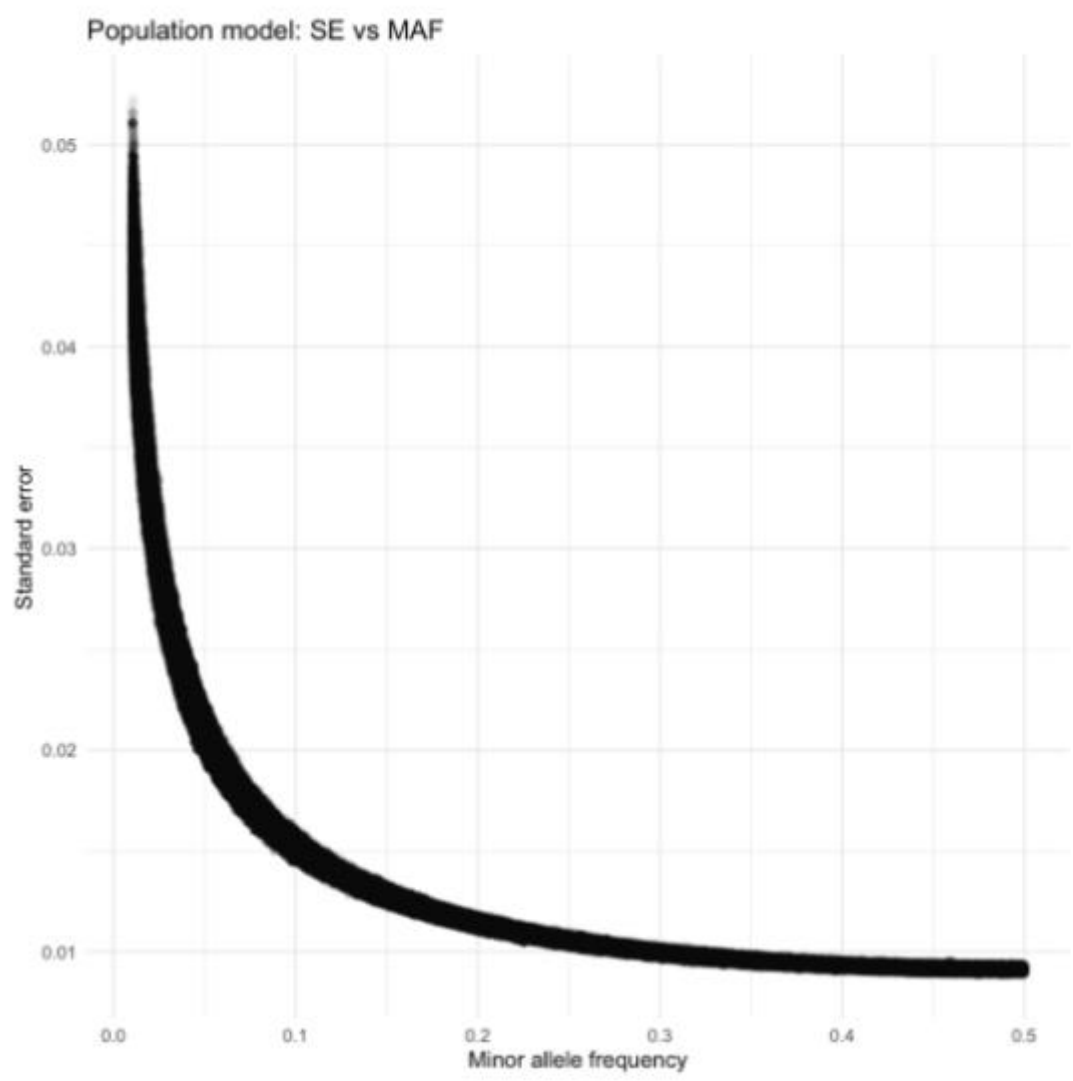

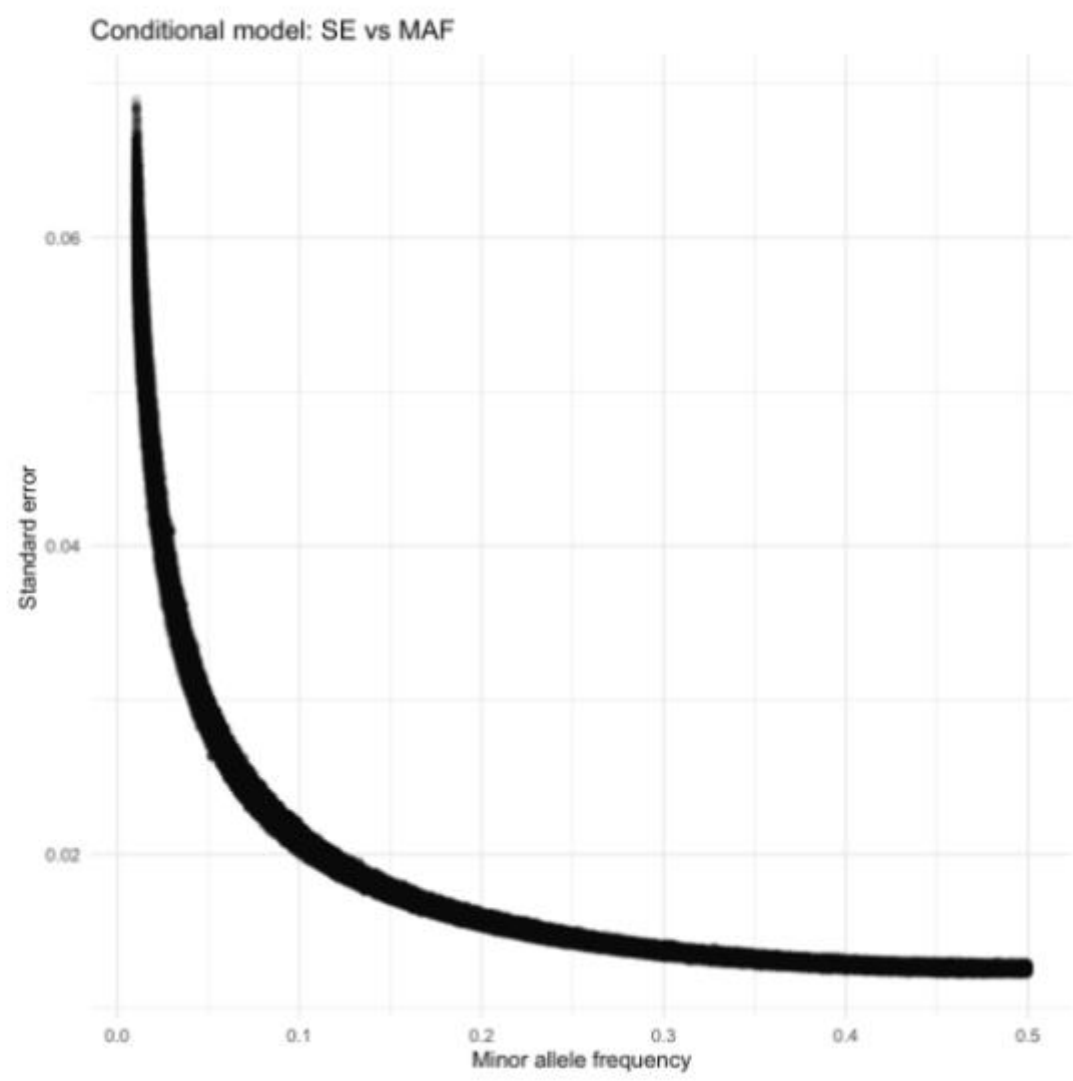

Depression symptoms

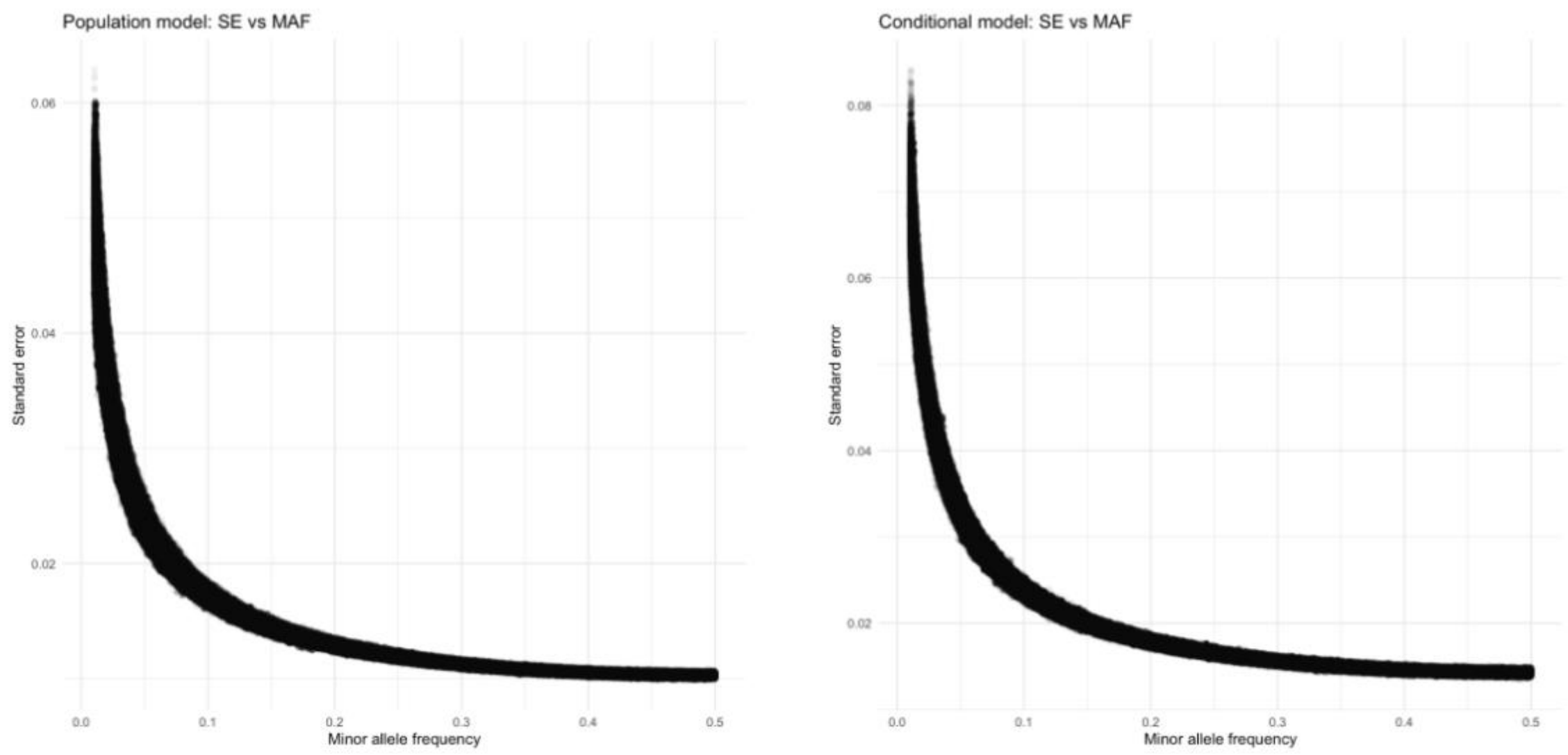

7. I recommend adding a discussion about the discrepancy between the heritability estimate from LDSC and the variance component analysis from trio-GCTA. It may not be immediately obvious that LDSC is more sensitive to GWAS power than individual-level methods like GCTA.

Response: We have added this point in the methods section on trio-GCTA (page 21, line 610-614): "LDSC uses GWAS summary data, meaning its power is influenced by both the heritability of the trait and the precision of the underlying GWAS. In contrast, trio-GCTA does not use any summary data and estimates heritability as part of a variance decomposition, using individual-level genetic data. Therefore, while both methods can be used to estimate SNP heritability, their properties (and, typically, the underlying data they use) differ substantially."

8. Considering the power of trio-based GWAS, I suggest exploring the method presented in https://www.nature.com/articles/s41588-022-01062-7#Sec9 to calculate effective sample sizes as input for LDSC, rather than using the raw sample size.

Response: Thank you for this helpful suggestion. We have now calculated effective sample sizes for each phenotype and model as input for LDSC. The procedure applied is described in the methods (page 19 line 542-547) "For the LDSC N parameter, we used the effective sample size (based on standard errors) for each phenotype and model. Effective sample size was computed per SNP using the following formula:

$$Effective\ N = \left(\frac{1}{SE^2}\right) \times \left(\frac{{SD_{Resid}}^2}{(2\ \times MAF\ \times\ (1 - MAF))}\right)$$

where SE is the standard error of the SNP effect estimates, MAF is the minor allele frequency, and SD_{Resid} is the residual SD of the phenotype after covariate adjustment.".

9. Additionally, there are some issues with the legend in Figure 4.

Response: Thanks for noting this – Figure 4 has been reproduced in this revision, with additional results included, and we have checked that all legends are displaying correctly.

Referee #2

1. I co-reviewed this manuscript with one of the reviewers who provided the listed reports.

Response: Thank you for taking the time to co-review our manuscript. We appreciate your involvement in the review process.

Referee #3 behavioural genetics

1. This manuscript describes an important innovation in the conduct of genetic association studies, perhaps especially of behavioral traits. The majority of human genetic studies were for a long time based on families. With technological advances and knowledge of linkage disequilibrium, the population genetic study became feasible and powerful. As the present paper describes, population studies have been valuable but have limitations that can be addressed at least partially with a returned focus on families.

   Leading the way in this space is the cohort described here, the Norwegian Mother, Father, and Child Cohort Study. (What does MoBa stand for?) This sample is contributing in valuable ways to a wide variety of important questions that simply cannot be addressed in population datasets, including mating patterns and parsing so-called "direct" from "indirect" genetic effects. Some of these advances are illustrated in the present paper.

   The manuscript is straightforward. It describes briefly the cohort, quality control procedures as they may apply to a family dataset. Then, for two example phenotypes, the authors estimate a handful of genetic estimates (e.g., heritability, polygenic score effects) for two child phenotypes (height at age 7, standardized educational test results at age 10). Then re-estimates them again in the context of an analytical design that includes the parents. I am extremely partial to the claim that large genotyped family cohorts are a new frontier with major potential in the study of medicine and human behavior. So I certainly agree with the premise of this paper. As a reviewer, I was left with several questions, which I outline here, all of which are relatively minor.

Response: Thank you for taking the time to read and review our manuscript. The acronym MoBa comes from the first 2 letters of each of the Norwegian words for mother "Mor" and child "Barn". We have responded to each of your questions below.

2. Could the authors define or characterize what are "direct" and what are "indirect" genetic effects? They have mathematical definitions in the context of the models employed here, but I would hope there is some conceptual scientific guidance available for the reader to help them understand how the math relates to the concepts. In particular, how do such analyses pin down specific causal environments? An indirect effect could be anything correlated with the parent genotypes, including stratification, parenting, occupational success, etc. Does the improved estimate of the direct effect allow one to prioritize genetic variants more or less likely to be causally interpretable?

Response: We have added key term definitions in the second paragraph of the main text introduction (page 3, line 41-52): "increasing evidence suggests that genetic associations estimated from unrelated samples reflect not only direct genetic effects - where an individual's genetic variants causally influence their own phenotype - but also correlated factors, such as indirect genetic effects on an individual's phenotype arising from the genetic influence of another individual through environmental or social processes [6,7]. Overcoming confounding of direct genetic effect estimates is very challenging in samples of unrelated individuals[3,8]. Family-based analyses can directly control for the parental genotypes and exploit the random inheritance of genetic variation from parents to offspring to preclude many forms of environmental confounding. Therefore, using large samples of genotyped related individuals[9-16] is one of the most compelling ways to overcome environmental confounding and estimate the contributions of direct and indirect effects of both genotypes and phenotypes."

Further description of direct and indirect genetic effects is included on page 7, line 231-238: "Inclusion of parental genetic effects in downstream genetic analyses – whether based on specific genetic propensities indexed by PGS, or based on decomposition of all genetic variance in trait, gives new insights into genetic mechanisms. Neither approach conclusively proves why children's traits associate with parental genotypes, so-called *indirect 'effects'* comprise many different sources of association[3,8]), but both can demonstrate that the children's own genotypes and phenotypes are likely confounded. Moreover, for specific genetic propensities and genome-wide variation respectively, both approaches can provide unbiased estimates of the direct effects of children's own genotypes on their phenotypes.".

We also added as a future direction in the conclusions section (page 8, line 258-269): "Estimates of direct genetic effects using trio data allow for prioritization of variants that are more likely to be causal. However, estimates of *indirect* genetic effects – those linking parental genotypes to children's traits – may still be confounded by broader demographic factors, such as assortative mating, population stratification, dynastic effects, and indirect genetic effects from relatives other than parents. Strategies to disentangle these factors will involve triangulation across genetic designs – including those demonstrated here, and others such as mediation analyses (e.g.,Cheesman et al. 2020[17]; Huang et al., 2024[18]), extended pedigrees (e.g., Nivard et al., 2024[19]), and assortative mating modelling (e.g., Torvik et al., 2022[20]), as well as across multiple sources of family genetic data. Furthermore, as within-family GWAS incorporating samples like MoBa become larger and more powerful, the potential to directly mitigate and control for confounding from assortative mating and population stratification in downstream within-family analyses will also increase.".

3. Could the authors provide an explanation of why they find such large indirect effects, for height especially? It would seem their models assume assortative mating is zero (perhaps in addition to other assumptions). High assortative mating, expected for education and somewhat for height, would I expect result in overestimation of indirect effects and actually accidentally control for direct effects. In fact, it would seem assortative mating is an important consideration for many if not all the findings reported here. Although AM is to me a very complex issue and Norwegian scientists working in MoBa are making among the most important recent contributions (e.g., Torvik et al. 2022 Nat Comm, 1108). Also specific to the polygenic scoring work, please see: https://www.biorxiv.org/content/10.1101/2023.07.10.548458v1.full

Response: Regarding an explanation for our results for child height: to summarise, our Trio-GCTA analyses find: direct effects 32.0% [SE: 2.8%], maternal indirect: 2.4% [SE: 2.4%], paternal indirect: 5.3% [SE: 2.5%]. Adding these genetic components together, the total variance explained by genetic effects is 39.7%. Of this, the proportion attributable to direct genetic effects is 80.6%, with 19.4% percent attributable to combined indirect genetic effects. The Trio-GCTA estimate of direct genetic effects for height is lower than previous studies that have indicated that approximately 90% of the SNP heritability for height can be attributed to direct genetic effects, with limited or negligible indirect effects (e.g., Kong et al., 2018[6]; Young, 2023[21]). However, our estimate for direct genetic effects is fairly consistent with other recent studies, at only 2-3% higher than in the Family GWAS by Tan et al., 2024[22] and the sibling GWAS in Howe et al., 2023[1], with overlapping standard errors.

Notwithstanding the relative consistency of the results for height with other recent studies, we acknowledge and appreciate your insightful feedback regarding the need to consider assortative mating. We fully agree that assortative mating represents an important consideration in interpreting our findings, and accordingly we have now noted it as a limitation in the discussion (page 8, line 259-262): "However, estimates of *indirect* genetic effects – those linking parental genotypes to children's traits – may still be confounded by broader demographic factors, such as assortative mating, population stratification, dynastic effects, and indirect genetic effects from relatives other than parents." Assortative mating is complex to model, and significant methodological development is

needed to do this appropriately for all the various methods we employ here. We feel that delving into the complexities of assortative mating will have the effect of detracting from our broader goal of presenting the potential of within-family genetic analyses in the MoBa resource to a diverse audience.

We think that the key point here is to ensure that readers get clarity about what can and cannot be inferred on the basis of indirect effect estimates from analyses such as these – as per your previous comment. As noted in response to that comment, we have added to the discussion (page 8 lines 262-266) that disaggregating the contributions of different sources of variation – included assortative mating – to the "indirect genetic effects" parameter(s) in these models is one of the key future directions in this field, to which MoBa can contribute: "Strategies to disentangle these factors will involve triangulation across genetic designs – including those demonstrated here, and others such as mediation analyses (e.g., Cheesman et al. 2020[17]; Huang et al., 2024[18]), extended pedigrees (e.g., Nivard et al., 2024[19]), and assortative mating modelling (e.g., Torvik et al., 2022[20]), as well as across multiple sources of family genetic data." We welcome collaborations and we hope that this resource paper will provide the foundations for future projects based on more sophisticated approaches.

Notwithstanding our conviction that the best approach in this context is to go only as far as highlighting the assortative mating issue for readers, for your information we checked the partner PGS correlations and note that only educational achievement (r=0.14) has an observed spousal correlation above 0.06 in the sample – indicating that AM likely plays a relatively limited role in the results at least for the PGS analyses.

4. This paragraph is quite minor and perhaps a reach and I am not requesting new analyses, but in the PTDT analyses the standardized test selection is made on the phenotypic value of a child (in the highest 90% only, with no consideration of the lowest 10%), who then is expected to have higher PGS than their mid-parent. The sibling is expected to have a PGS no higher than the mid-parent. But if the sibling's educational attainment affects the child's, and the child is selected based on phenotype, then wouldn't one expect the sibling to have higher than mid-parent PGS as well? The effect here may admittedly be vanishingly small. Additionally, in the PTDT analyses is it possible that ascertainment and retention affects the results? If higher-achieving siblings are retained and lower-achieving siblings drop out, then aren't the siblings selected for higher educational attainment PGS, and lower smoking PGS (as they will be correlated)? Generally, some information on retention would be valuable for readers to understand this cohort and feasibility of similar efforts. On a search, I have trouble finding retention rates in MoBa other than the 2016 cohort update in Int J Epi, but I recall they are around 40-50% over a five-year follow-up.

Response: Thank you for this comment, in which you raise a number of important points. First, it is correct that the model assumes no sibling interaction effects, though we agree with the speculation that these would likely be small. In fact, control for "indirect effects" in all of our analyses applies only to *parental* indirect effects, and we now note this in the methods section (under heading 'Trio genetic analyses' [page 18 lines 497-505], new text: "Where analyses estimate or account for "indirect genetic effects" these, refer exclusively to variation in child outcomes associated with parents' genotypes (i.e., not those originating with siblings or other family members). The term "indirect genetic effects" is used for consistency with the standards in the field, despite all methods being agnostic as to the reasons for this covariation, which can include: indirect effects of parents, population structure (the presence of subgroups within a population that can bias genetic associations), and assortative mating (the tendency for individuals with similar or dissimilar traits to mate non-randomly, influencing genetic associations in the next generation) among other sources.".

Second, ascertainment and attrition-related biases are important considerations in all analyses presented here, and we now note this explicitly in the main text "the effects of non-random ascertainment and attrition represent crucial considerations for within-family analyses using such data." (page 8, lines 280-281). References to papers describing representativeness and retention rates have been added to the sample description of the methods section (page 17, added text in italics lines 452-453):

"Participants were recruited from all over Norway from 1999 to 2008. The women consented to participation in 41% of the pregnancies (N=112,908 recruited pregnancies)[2,23]. The cohort includes approximately 114,500 children, 95,200 mothers and 75,200 fathers. Additional information about MoBa establishment is contained in Supplementary Methods 1. *Further details about the cohort representativeness and participation in specific waves of data collection are described elsewhere*[2,23-25]."

5. Following references 47, 48, and 49 to understand where the polygenic scores came from I wonder if there is an error. Reference 49 did not produce smoking summary statistics. They used existing summary statistics from Liu et al. (2019) Association studies of up to 1.2 million individuals... Nature Genetics, 51, 237-244.

Response: Thank you for alerting us to this issue. We have now included the appropriate reference:

Karlsson Linnér R, Biroli P, Kong E, Meddens SFW, ... Benjamin DJ, Koellinger PD, Beauchamp JP. Genome-wide association analyses of risk tolerance and risky behaviors in over 1 million individuals identify hundreds of loci and shared genetic influences. Nat Genet. 2019 Feb;51(2):245-257. doi: 10.1038/s41588-018-0309-3. Epub 2019 Jan 14. PMID: 30643258; PMCID: PMC6713272.

6. If space is a problem for any revisions, I would think the extensive genotype QC information could be left to supplementary materials.

Response: Thank you for pointing out this solution – we have now moved all of the QC methods to the Supplementary Methods and the majority of the QC information from the main text to Supplementary Note 2.

7. No single study design is a panacea and no group of scientists is in a better position to describe limitations of family designs like MoBa than the authors of this manuscript. Could a brief discussion of strengths and limitations be provided for the curious reader? For example, parent-child designs may not be suitable for phenotypes with age of onset after mid-adulthood because parents will be censored (e.g., deceased). Perhaps more broadly, any phenotype where aetiology or measurement differs as a function of age or birth year may pose problems given developmental or generational changes (e.g., years of education in parents versus standardized tests of 10-year-olds).

Response: We agree that readers will benefit from an expanded and more balanced discussion of the merits and limitations of MoBa and related samples. We are somewhat limited in the scope of this by space, but have expanded the final paragraph of the discussion to incorporate a couple of your suggested limitations (and one other), as well as strengths of the family cohort design (pages 8-9, lines 270-289):

"Beyond their family-based structure, cohorts such as MoBa have many other distinctive features that can facilitate the development and application of new methods and innovative uses of genotype data. For example, MoBa features a prospective, longitudinal follow-up of more than 100,000 children from prenatal life to adulthood, allowing for comprehensive tracking and genetic analysis of developmental trajectories. Additionally, the 10-year recruitment period presents opportunities to study temporal interplay between genetics and shifting environmental and societal contexts that impact health and

behaviour across generations. Nevertheless, no single resource or study design is without limitations. Lengthy recruitment periods and follow-up periods can present challenges as well as opportunities, particularly where measurement cannot be kept consistent either across time or age. Furthermore, the effects of non-random ascertainment and attrition represent crucial considerations for within-family analyses using such data. Finally, family-based cohorts such as MoBa are expensive to recruit and maintain, and care must be taken to ensure that prioritisation of family-based cohorts in the coming years does not undermine ongoing efforts to diversify genetic samples and ensure insights can benefit the broadest possible population. Despite these limitations, a combination of widespread implementation of existing within-family approaches to new data sources and the novel integration of these approaches with longitudinal designs and natural experiments, means that research in family-based cohorts is likely to play a central role in promoting groundbreaking discoveries in genetics in the coming years.".

8. After reading this impressive effort, my main reaction was that I found myself wondering what the primary purpose of this manuscript was. It was a description of the cohort but may benefit from important details, such as brief descriptions of catchment, consent rates, retention, representativeness, extent of phenotyping. As an illustration of the utility of families it explores findings for only two child phenotypes, height and standardized test results, showing that estimates may differ in singleton versus trio designs, but does not really attempt to interpret these findings or characterize their scientific import in detail. A strength of MoBa are the large families, including extended families with sibling, avuncular, mating, and cousin relationships, but the utility of these is not explored. Because there are only two phenotypes, I was left wondering to what extent the family design is relevant across many more phenotypic domains. MoBa is probably the single best place to explore such issues.

Response: Thank you for this comment. We have revised the manuscript to clarify its twin aims of a) illustrating the importance of genotyped family cohort data in advancing genomic research in the coming years and b) highlighting and exemplifying the MoBa cohort as one such resource. Further, we have added an overview of the extensive phenotyping of family members over the more than 25 years of ongoing data collection, and expanded the exemplar phenotypes to include depressive symptoms and sleep duration. We selected these additional traits to allow us to more comprehensively illustrate the utility of the family design and exemplify the breadth of phenotyping in MoBa, but – crucially – without compromising the high-level, illustrative nature of the analyses. Going either too broad or too deep with our analyses and interpretations would undermine the accessibility of the paper for its target audience. We hope the changes suitably address the nuanced point you raised and that you agree that the paper is now both more informative and better balanced.

Revised paragraph on the study aims (pages 3-4 lines 66-76): "Despite the valuable insights possible from studying genotyped families, few cohorts or biobanks have implemented large-scale family-based sampling[9-16]. Here, we emphasise the critical role genotyped family cohorts will play in advancing genomic research on human traits in the years ahead, highlighting the Norwegian Mother, Father, and Child Cohort Study (MoBa) as a prime resource. MoBa combines extensive family-based genetic data with rich prospective and longitudinal follow-up of ~100,000 children from prenatal life into adulthood.[2] By applying a range of trio-based methods to analyse MoBa children's height (age 7 years), educational achievement (age 10 years), sleep duration (age 7 years), and depressive symptoms (age 8 years), we demonstrate the valuable insights that can be gained by incorporating maternal and paternal genotypes in investigation of genetic effects across diverse phenotypic domains."

We have rewritten the second half of the first paragraph of the conclusion to better connect our examples to the question of the importance of incorporating genotyped family cohort data in order to move genomic research forward in the coming years (page 8, line 249-257): "Our trio-GWAS results clearly illustrate how the nature of these associations cannot be assumed. Moreover, we show not only inflation of heritability estimates in population-based *versus* trio-GWAS, but also substantial

changes in the patterns of overlap with other traits. Downstream, polygenic score- and variance decomposition-based approaches draw out these nuances further, highlighting avenues for future investigation — such as parent-specific indirect effects and opposing effects of the same genetic propensities in children and parents. Analyses and samples such as these represent a jumping-off point for the defining challenge of the post-GWAS era: the elucidation of *mechanistic* links between common genetic variation and human traits.".

To better highlight the unique possibilities to use MoBa for family studies, we have added a new figure (now Figure 1) illustrating the breadth and scale of available data on relevant traits across family members and time in the cohort. Added on page 4 (lines 79-84): "MoBa represents a uniquely powerful resource for family-based genetic analyses, offering a wide range of phenotypic measures on mothers, fathers, and offspring collected from early pregnancy and spanning more than 25 years. Within the completed data collections covering the first fourteen years of life alone, MoBa has amassed observations on neurodevelopment, mental health, physical health, health behaviours and medication use, social and environmental exposures and more (Figure 1).".

Regarding the suggestion to include brief descriptions of catchment, consent rate, and retention, further information has been added as noted in our response to your comment 4.

9. Thank you for the opportunity to read and comment on this fine manuscript. I hope the above comments are somewhat helpful, I wish you the best in publishing this important work, and look forward to having this study in print.

   I am in the habit of signing my reviews.

   Scott Vrieze
   University of Minnesota

Response: Thank you, Scott, for taking the time to read and review our manuscript. Your suggestions and comments have indeed been very useful.

Referee #4: biostatistics, family design

1. In this manuscript, Corfield et al. present the Norwegian Mother, Father, and Child Cohort Study (MoBa) genomic data processing pipeline and use the resulting data to illustrate the benefits of family-based studies for identifying direct genetic effects that may be confounded in population-based studies.
   With respect to the MoBa pipeline, genomic data processing pipelines addressing relatedness arising by design have already been proposed in other studies (e.g., DOI: 10.1016/j.ajhg.2015.12.001). This study's pipeline does not incorporate some state-of-the-art methods from the preceding reference or more recent ones, although the steps seemed generally reasonable.

Response: Thank you for taking the time to read and review our manuscript. We have systematically gone through and responded to your concerns below.

We greatly appreciate the work already done to establish pipelines to process genomic data, and more specifically family-based genotype data, as noted in Supplementary Note 1 & 3. In this text we state that the Pedigree Imputation Consortium Pipeline (PICOPILI), was the basis for the QC, phasing, imputation, and post-imputation QC process utilised by MoBaPsychGen. Furthermore, we list the reasons why we were unable to use a pre-existing pipeline for MoBa, namely: (1) the cohort size, (2) the complex interrelatedness, and (3) the idiosyncrasies of MoBa (e.g., inclusion of within and across batch duplicates and the large imbalance in number of individuals clustering with reference

superpopulations). We have added the following text in Supplementary Note 1 (page 27, lines 855-866; new text in italics):
"PICOPILI offers scripts for pre-imputation QC, phasing, imputation, post-imputation QC, and genome-wide association studies (GWASs) of family data with arbitrary pedigrees, alongside tools for imputation and family-based analyses. However, some of the software used in PICOPILI is not designed to handle large sample sizes. Furthermore, generic open-source pipelines such as PICOPILI are – by design – not equipped to handle idiosyncrasies of specific cohorts or studies. *As such, other studies have also had to adapt in house protocols tailored to meet the cohort specific characteristics. For example, QC pipelines optimized to account for high levels of recent admixture and population diversity*[26,27]. *We were unable to use a pre-existing pipeline for the Norwegian Mother, Father, and Child Cohort Study (MoBa) due to (1) the cohort size, (2) the complex interrelatedness, and (3) the idiosyncrasies of MoBa (e.g., inclusion of within and across batch duplicates and the large imbalance in number of individuals clustering with reference superpopulations).*".

2. I would note that the division of the genomic data pipeline description between the Methods and Supplementary Methods made for confusing reading, with key pieces of information scattered across two locations. I would highly recommend putting all these details into the Supplementary Methods if space is an issue. There are also several points where clarification is required, including some areas of concern detailed in specific comments below.

Response: Thank you for pointing out this solution – we have now moved all the QC methods text and the majority of the QC information from the main text to the supplementary materials. Furthermore, we have responded to the points raised in the specific comments below.

3. In terms of the second major objective of this study, pointing out that large-scale family-based studies have unique utility for estimating direct genetic effects in the era of population-based biobanks is important, and the authors provide interesting examples from MoBa. However, the authors provide rather limited descriptions of the theoretical justification for each family-based approach used, and the references were not always apropos. Moreover, there are some methodological clarifications and issues in these analyses that need to be addressed detailed in the specific comments below.

Response: We have expanded the range of examples, the discussion of results, and the theoretical justification of the approaches in this revision, as well as checking and updating references throughout. We hope to have satisfactorily provided the requested clarifications in response to the specific comments below.

4. **MobaPsychGen Pipeline**

   - Module 0: Was conversion of A/B alleles done to obtain A, C, T, or G *on the plus strand*?

Response: The conversion of A/B alleles was performed to obtain A, C, T, G alleles on the top strand. We have added "on the plus strand" in text (page 35 line 1167) as suggested.

Module 1
-- How was relatedness accounted for in HWE testing? The PCA methods seemed to account for relatedness by performing PCA in 1000 Genomes and founders only, for example.

Response: PLINK by default only uses founders for allele frequency estimation and HWE filtering. We used this method of only including founders to account for relatedness (as described in Supplementary Note 3 [page 29 lines 941-942]), in both MAF and HWE filtering. To make this clear we have added "(within founders only to account for relatedness)" in the MoBaPsychGen methods description in all instances that MAF and HWE filtering is described.

-- For PCA, it was unclear which method was used for projection of relatives onto the PC space learned from 1000 Genomes and founders, although a modern approach would use Online Augmentation, Decomposition, and Procrustes (OADP; DOIs: 10.1093/bioinformatics/btaa152, 10.1093/bioinformatics/btaa520) to eliminate centroid bias. Please specify the method used and justify if it was not OADP or ADP.

Response: In the MoBaPsychGen protocol, PLINK 1.9 and FlashPCA2.0, which both use simple projection were used to project related individuals onto the PC space derived from the 1000 Genomes Project referent and MoBa founders. We agree with the reviewer that the more recent Augmented Decomposition Projection (ADP) and Online Augmentation, Decomposition, and Procrustes transformation (OADP) offer methodological advantages over simple projection.
However, relatively computationally efficient tools implementing these approaches that are suitable for large samples were published (or made available as pre-prints) after the MoBaPsychGen QC protocol was established, finalized, and QC analyses had started being conducted. Therefore, to ensure continuality, and timely release of the data, we decided to consistently apply the same methodology across all genotyping batches. We acknowledge that this is a limitation and have included the following text in Supplementary Methods 5 (page 42-43 lines 1474-1490) to recognise this:

“Recent methodological advancements in PCA projection[28,29], including the development of relatively computationally efficient software packages, have enabled improved identification and adjustment of fine-scale population structure in homogenous samples. In particular, the online augmentation, decomposition and Procrustes transformation (OADP) is a projection method, which is relatively computationally efficient[28]. In the UK Biobank, lower scaled mean-squared differences (MSD) were reported compared to simple projection (SP; as implemented in PLINK1.9 and FlashPCA2.0) in European populations (OADP MSD=0.100 vs SP MSD=0.360), while negligible differences were reported for diverse populations (OADP MSD=0.156 vs SP MSD=0.156)[28].

Unfortunately, the relatively computationally efficient OADP software was not available until after the MoBaPsychGen QC protocol was established, finalized, and QC analyses had commenced. Therefore, to maintain methodological consistency across all genotyping batches, particularly considering the estimated computational runtimes were not negligible we decided to consistently apply the SP method throughout the MoBaPsychGen protocol. Given the continuous improvement of methodology, software efficiency, and imputation reference panels, we anticipate that the protocol will be updated in the future to incorporate these advancements, however, it is outside the scope of the current project.”.

-- Improved reference callsets, including the 1000 Genomes phase 3 callset with ~2500 individuals, have been available since 2015. Why was 1000 Genomes phase 1 used for ancestry inference?

-- The use of visual clustering for ancestry group assignment seems arbitrary. There are automated approaches to do this based on defining hyperellipsoids in the ancestry PC space (DOI: 10.1016/j.ajhg.2015.12.001), and there are also approaches that use continuous ancestry for ancestry-adjusted QC (DOI: 10.1016/j.ajhg.2015.11.022, authors’ reference 15, DOI: 10.1001/jama.2023.11970), which would obviate the need to define these groups.

Response: We respond to these two comments together below.

At the inception of the study, the choice to use 1000 Genomes phase 1 data was motivated by the fact that some of the batches were genotyped using Illumina HumanCoreExome12v1.1 and Illumina HumanCoreExome24v1.0 arrays in 2014 and 2015, prior to the release of the 1000 Genomes phase 3 data. Since 1000 Genomes phase 1 data was used in the design and selection of variants for these

arrays. To maintain consistency throughout the release, we continued to use Phase 1 data throughout the QC process.

We agree with the reviewer that using the 1000 Genomes phase 1 instead of phase 3 is a limitation of the QC protocol and, as noted in our previous response, anticipate that with improvements in methodology and reference panels the protocol will be updated in the future. However, we demonstrate in the new analysis described below and in Supplementary Methods 4 (page 42 lines 1460-1467) that the differences between 1000 Genomes phase 1 and phase 3 data for the purpose of identifying individuals clustering with reference superpopulations in MoBa are minimal.

Indeed, the fact that 95% of genotyped MoBa participants cluster with the 1000 Genomes, phase 1, European superpopulation illustrates MoBa is a quite homogenous cohort. Norway's population, including Indigenous (Sami) and minority (Kvens/Norwegian Finns, Forest Finns, Roma, Romani/Taters, and Jewish) populations, are not represented, in either the 1000 Genomes phase 1 or phase 3 reference callsets. Therefore, using the 1000 Genomes phase 3 reference callset is unlikely to offer substantial improvement in identifying individuals clustering with reference superpopulations in MoBa. These are the reasons that visual inspection, which we agree would seem arbitrary for a diverse cohort, was used in the MoBaPsychGen protocol instead of automated clustering and is supported by the comparative analysis described below.

In the Module 1 methods text (page 35-36 lines 1202-1205), we stated "There is on-going discussion about how to define and manage genetic ancestry in epidemiological analyses practicably and accurately[30], and classification into discrete ancestral groups is used as a convenient simplification here". We have expanded this sentence to include "as the vast majority of MoBa participants clustered with a single reference superpopulation." (pages 36 lines 1205-1206). We further state "As new approaches emerge to appropriately handle the continuous nature of ancestry in cohorts with large imbalance of individuals clustering with a single reference superpopulation we will update our QC procedures." (page 36 lines1206-1208).

To quantify the potential difference in ancestry group assignment, we used 1000 Genomes, phase 3 data to perform supervised clustering with support vector machines using the following script: https://github.com/MRCIEU/ukb-rap-utils/blob/main/scripts/ancestry/king_ancestry.r. Results from this analysis showed considerable overlap, with 98.5% identified as clustering with the European superpopulation in both the automated and visual clustering. The results from this investigation have been included in Supplementary Methods 4, in addition to expanding the limitations section in Supplementary Note 3, which now states: "Secondly, the usage of 1000 Genomes phase 1 reference, with visual inspection. However, the use of supervised clustering in MoBa using 1000 Genomes phase 3 as the reference revealed minimal differences in identification of individuals clustering with the European superpopulations. Thirdly, since the majority of MoBa participants are of European genetic ancestry, the analyses were restricted to this homogenous ancestry group. Multi-ancestry QC and imputation should be implemented for future studies." (pages 30-31 lines 997-1002).

The large imbalance of individuals clustering with the 1000 Genomes European superpopulation resulted in small numbers of individuals clustering with the African, Admixed American, East Asian, and South Asian 1000 Genomes superpopulations, which were spread across the 26 genotyping batches. This made it incredibly challenging to apply ancestry adjusted QC approaches to MoBa, given some genotype batches included <5 individuals clustering with specific superpopulations.

- Module 2

-- The authors are incorrect in stating that heterozygosity QC cannot be performed with continuous ancestry adjustment; they should see the supplement to reference 15 for details on how to adjust heterozygosity for continuous ancestry PCs. A similar approach has been used by others with diverse cohorts including family members (DOI: 10.1001/jama.2023.11970).

Response: We apologize for our inaccurate statement regarding heterozygosity QC. We have reviewed the supplement to reference 15, as well as cited the Jordan et al. (2023) study[27]. Based on this we have removed the original statement from the manuscript.

-- A more robust approach than KING for relatedness QC might be applying the newer PC-Relate technique (DOI: 10.1016/j.ajhg.2015.11.022) to the entire sample. This technique improves on KING for admixed samples and provides an estimate of P(IBD=0) to distinguish full siblings from parent-offspring pairs.

Response: We thank the reviewer for the suggestion of applying the newer PC-Relate[31] technique to the entire sample. The main improvement of PC-Relate compared to KING is for admixed and diverse cohorts. Given this is not the case in MoBa, with the vast majority of individuals in MoBa clustering with the 1000 Genomes European superpopulation, concern for population structure bias in kinship estimates is minimal. As such, we are confident in the pedigree we constructed for MoBa.

An additional advantage for MoBa was that we were able to construct an initial pedigree of expected parent-offspring relationships within MoBa. As such the main goal of the relatedness checks was to confirm reported relationships before inferring unreported relationships (primarily in the parent generation). We were also able to use year of birth information ensure inferred relationships were possible, for example parent-offspring relationships within the parent generation.

Supplementary Methods 8 has been expanded to include the following details: "In the MoBaPsychGen pipeline, we used a similar strategy to the UK Biobank[32] to investigate the relatedness structure within MoBa. This involved first checking and inferring relationships using KING (kinship coefficient and proportion of SNPs with zero identical-by-state; IBS0 estimates) before confirming the relationships using the PLINK genome function (estimates of PI_HAT and probability of 0, 1, and 2 alleles are shared IBD; Z0, Z1, and Z2, respectively) to ensure the pairwise relationships were as expected for the types of relationships in MoBa (including distinguishing full-siblings from parent-offspring pairs)" (page 43 lines 1509-1515).

We further state: "PC-relate[31] is a newer method than that improves on KING for admixed and diverse cohorts. Given this is not the case in MoBa, with the vast majority of individuals in MoBa clustering with the 1000 Genomes European superpopulation, concern for population structure bias in kinship estimates is minimal." (page 44 lines 1530-1533).

- Supplementary Methods 19: Were PCA and other analyses conducted again on imputed SNPs? If not, it is unclear how the checks based on PCs would reveal batch effects arising from imputation.

Response: Yes, the PCA and other analyses were conducted using imputed SNPs. To make this clearer, we have added "using imputed SNPs" to the post-imputation QC text where PCA is described (page 41 lines1416 and 1417).

-- What was the rationale for imputing separately by batch? This might create batch-specific variation due to variants being typed in some batches but fully imputed in others. Others have found that using the intersection of SNPs on all arrays is necessary to avoid bias in imputation

(DOI: 10.1007/s00439-013-1266-7), and some studies have recommended two rounds of imputation in the context of combining array-based and WGS genotypes (DOI: 10.1038/s42003-022-03738-6).

Response: We thank the reviewer for raising this concern as we spent a great deal of time deciding on how to appropriately handle imputing the 26 MoBa genotyping batches.

Given MoBa is a family cohort, we decided that the protocol needed to place importance on the impact to haplotype estimation in addition to imputation quality and bias, plus performance of polygenic scores. During the QC process we discussed with Vivek Appadurai the best approach to limit phasing and imputation errors and biases in the MoBa genotyping data. His recent paper[33] performing benchmarking work, that investigates phasing, imputation, and polygenic score performance, based on four data integration protocols for on how phasing and imputation were performed in batches genotyped with different arrays. The four data integration protocols evaluated were: (1) “separate”, where the batches were phased and imputed separately; (2) “intersection”, where batches are merged and phasing and imputation are performed together based only on SNPs genotyped in both batches; (3) “union” where batches are merged and phasing and imputation are performed together based SNPs genotyped on either batches; and (4) “two stage” where phasing, and imputation are performed separately by batch before merging the “union” SNPs and performing phasing and imputing of the merged dataset. The benchmarking results showed that due to the specific genotyping data available (e.g., multiple genotyping batches, low SNP overlap between genotyping batches), trade-off situations arise whereby educated decisions need to be made. We strongly believe that is the case in MoBa.

Appadurai V et al (2023)[33] replicates the results of Johnson et al (2013)[34], showing that taking an intersection approach when incorporating samples genotyped on multiple arrays leads to a loss of imputation accuracy compared to the separate imputation approach. Furthermore, Appadurai V et al (2023)[33] showed that taking an intersection approach, with two arrays overlapping at approximately 180,000 markers, leads to increased Switch Error Rates (and attenuation in variance explained from polygenic score analyses) compared to the separate approach.

While establishing the MoBaPsychGen QC protocol, we determined the SNP overlaps of 252,110 for HCE-OMNI pair, 105,360 for HCE-GSA pair, and 137,341 for OMNI-GSA pair. We further determined that after pre-imputation QC, there were less than 200,000 overlapping SNPs when the three batches genotyped with HCE and four batches genotyped with an OMNI were merged. This low intersection was determined to impact the quality of imputation, potentially deteriorating performance of polygenic risk scores. Therefore, we performed phasing and imputation separately for the HCE and OMNI batches. Meanwhile, the batches genotyped using the GSA array were not phased and imputed in a single imputation batch because the genotyping batches were received at varying timepoints after imputation and post-imputation QC of other GSA batches had already been performed.

At this point, we determined that considerable investment of time and resources would be required to re-impute all the GSA batches together or perform a second round of imputation using well imputed SNPs from all imputation batches for minor improvement in imputation accuracy. Further to this, we have performed analyses to see the amount of inflation potentially introduced into genome-wide association analyses by performing separate phasing and imputation and determined that adjusting for genotyping batch negates this bias (Supplementary Methods 16).

In Supplementary Note 3 (page 30 lines 993-996), we had acknowledged “However, there are a few limitations of the pipeline. Firstly, the Global Screening Array (GSA) genotyping batches were not

phased and imputed in a single imputation batch because the genotyping batches were received at varying timepoints after imputation of other GSA batches had already been performed.". Further to this, we have now expanded Supplementary Methods 16 to include the considerations we took, as described above, in deciding not to perform a second round of imputation and the testing that we performed investigating imputation biases in MoBa.

Furthermore, we performed simulation analyses which show that adjusting for genotyping batch does not induce collider bias (https://explodecomputer.github.io/lab-book/posts/2024-03-27-collider-gwas/).

> -- For Y and MT, I can see the rationale for having completely separate descriptions of the QC pipeline. However, I would recommend including additional steps specific for the X chromosome and PAR in the description of each module for continuity. Having these separate makes it challenging to follow along with Figure 1a.

Response: We have incorporated the chromosome X and PAR specific QC steps into the main MoBaPsychGen QC protocol description. Hopefully, this is now easier to follow along with Supplementary Figure 9a (which was previously Figure 1a).

> -- In Figure 1e, there seem to be many unrelated pairs with estimated IBD well above expectation. Could the authors explain this observation or perhaps remedy the overplotting to make these easier to read? Also, the methods for the IBD segment length analysis were not described, and the authors should clarify that the right panel is comparing the KING estimate of phi on the y axis to the IBD segment-based estimate of 2*phi on the x axis.

Response: Thank you for pointing this out, there was an error in the original R script used to identify pairwise relatedness types, which led to a large proportion of individuals inaccurately identified as unrelated. We have corrected the script and updated Supplementary Figure 9e (previously Figure 1e) with the accurate relationship types.

We utilized three KING relationships inference algorithms in the MoBaPsychGen QC protocol, namely, build, related, and ibs. From the KING outputs, the primary values that we used to confirm relationship types were the estimated kinship coefficient and the proportion of SNPs with zero alleles identical-by-state (IBS0). Therefore, the only instance in the pipeline where IBD segment inference estimate is used is in Supplementary Figure 9e (previously figure 1e), which is produced by KING when the rplot flag is specified. Unfortunately, the KING IBD segment inference, which is an integrated part of the related analysis, is yet to be published (as mentioned in the KING manual: https://www.kingrelatedness.com/manual.shtml#IBDSEG).

The KING related analysis output includes columns entitled "Phi", which is the "Kinship coefficient as specified by the provided pedigree data", "Kinship", which is the "Estimated kinship coefficient (ϕ) from the SNP data", and "PropIBD", which is the "Proportion of genomes shared identical-by-descent, estimated by IBD2Seg + IBD1Seg/2, estimate of $\pi=\pi_2+\pi_1/2$" (https://www.kingrelatedness.com/manual.shtml#RELATED). We have confirmed that the value plotted in the right panel plot x-axis is the PropIBD estimate while the Kinship estimate is plotted on the y-axis.

5. **Trio-GWAS**

> ---------
>
> - Supplementary Methods 23 is missing.

Response: Apologies for the confusion, there is no Supplementary Method 23 (this text was incorporated into the methods section). Therefore, we have removed the reference to Supplementary Method 23.

> Were the parent-offspring trios selected such that offspring could be considered independently sampled from a larger population? This would not be the case if, for example, a sibship with K siblings were split into K trios. If selection was done to render offspring independent of one another, the method for doing so should be described. If not, linear regression with model-based standard errors does not appropriately account for relatedness; empirical standard errors or mixed models should be used instead (as was done for Trio-PGS).

Response: As with Howe et al. (2022)[1], we included siblings and allowed for within-family clustering of standard errors across sibships using a cluster-robust sandwich estimator (this is automatically applied by LDAK when calling --trios).

> - Reference 2 uses a within-between decomposition based on sibship genotypes, which is a bit different from the Trio-GWAS approach used here. The Trio-GWAS approach is most similar in spirit to the QTDT with parental genotypes (DOI: 10.1086/302698), which was not cited, but also uses a within-between decomposition to establish its properties. Because the authors do not provide a reference that clearly describes the Trio-GWAS approach and its properties, it would be useful for them to provide a more detailed theoretical development here.

Response: We have the below theoretical justification of the Trio-GWAS approach in Supplementary Note 4 (pages 31-32 lines 1018-1062):

"Family-based designs have a long history in statistical genetics. Abecasis et al. (2000) made an important contribution, deriving a QTDT test – a quantitative transmission disequilibrium test (QTDT)[35]. This test examines whether a quantitative (continuous) trait is associated with the transmission of genetic variants from parents to offspring, using either parental genotypes or sibships. In the case of trio data being available, the QTDT estimation model for the outcome includes the child and parental genotypes. They formally derived within and between family components, and argued that the random transmission of variants from parents to offspring can be used to overcome population stratification. Gauderman (2003) describes similar models in the context of candidate gene studies in which the offspring genetic effect is conditioned on the parental genotype[36]. Wheeler and Cordell (2007) extend these methods to quantitative traits in case-control samples[37]. Each of these methods exploits the random transmission of genetic variants from parents to offspring to obtain estimates of the direct and indirect effects of genetic variants.

Here, we use within-family trio-GWAS estimators which is estimated using the following model:

$$y_i = \beta_0 + \Gamma_1 g_{i,j} + \Gamma_2 g_{im,j} + \Gamma_3 g_{if,j} + \boldsymbol{\beta X} + v_{i,j}$$

This was described in Brumpton et al. (2020)[38], and is very similar in form to the QTDT model, and other recent papers[39]. Where the offspring phenotype is indicated by y, the offspring, mother and father genotype at loci l is indicated by $g_{i,j}$, $g_{im,j}$, and $g_{if,j}$, respectively. The residual or error term is indicated by $v_{i,j}$ which is modelled using cluster-robust standard errors, making no assumptions about the distribution of the phenotype, and allow for clustering by family. The matrix of covariates $\boldsymbol{X}$ include genetic principal components. Note, these are included to increase the efficiency of the estimator and not to control for bias in the estimated direct effect from population stratification. The direct effect of the genotype is estimated by Γ_1. The indirect effects of the maternal and paternal genotypes are indicated by Γ_2 and Γ_3, respectively They may be biased by residual confounding from population stratification after attempting to adjust for genetic principal components. As with QTDT

above, the association of the offspring genotype with the phenotype, conditional on parental genotypes, can provide an unbiased estimate of the direct effects of the genotype, absent other forms of bias (pleiotropy, linkage, and selection/collider bias). This follows from the random transmission of genetic variants from parents to offspring and can be demonstrated using the following Directed Acyclic Graphs (Supplementary Figure 11)[38,40].

Supplementary Figure 11. A Directed Acyclic Graph illustrating the relationships between offspring, maternal and paternal genotype and a phenotype. It represents the estimation model described in text; however, the unobserved confounding term $u_{i,j}$ influences the outcome and, if it represents, for example, population structure, can also influence the allele frequencies of the parental genotypes. In practice, many population-based studies attempt to capture this bias by using genetic principal components, but there is no guarantee of the PCs completely control this confounding. Alternatively, if the parents' genotypes are controlled for then all confounding paths from the offspring genotype to the phenotype are blocked, and the only remaining path in this graph is the direct causal effect from the genotype to the phenotype.

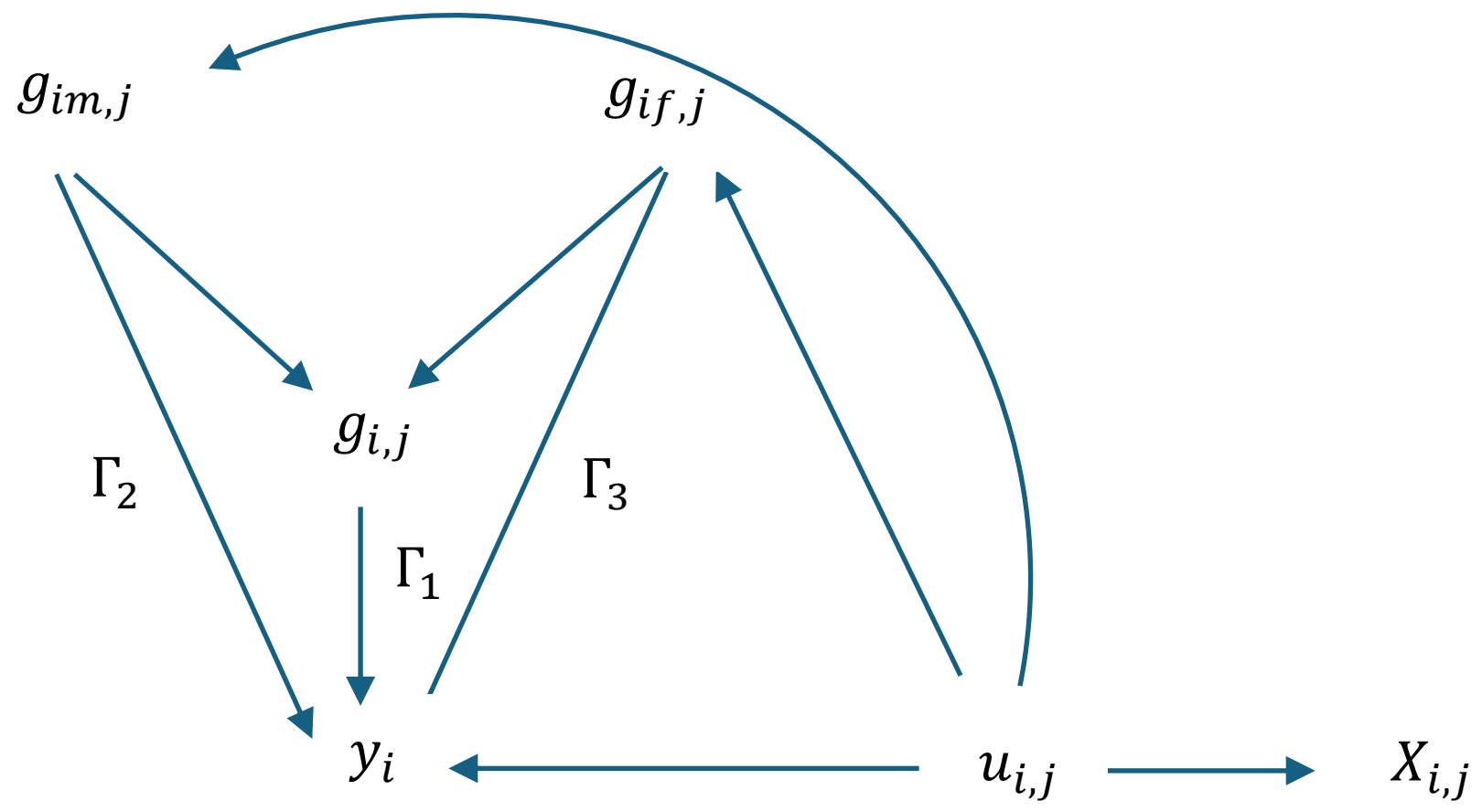

There are three causes of offspring genotype, $g_{i,j}$, mother and father's genotypes $g_{im,j}$ and $g_{if,j}$ and chance due to random Mendelian inheritance. Hence, once parental genotype are controlled for via statistical adjustment in a linear model, the confounding paths from parental genotype to the phenotype, any path that acts via parental genotype, which includes population structure, assortative mating and dynastic effects, are blocked. Hence, absent other mechanisms (e.g. pleiotropy or selection bias) adjusting for parental genotype allows for unbiased estimation of direct genetic effects. However, the interpretation of the coefficients on the parental genotypes is more complex, as any factor that affects parental genotype and the outcome can induce associations. Thus, the coefficients on the parental genotype may not merely reflect genetic nurture, but could also reflect bias from residual population stratification.".

Thus, this model has been described in the literature. Nevertheless, we would be very happy to provide more statistical details on this point, example, derivations of the causal model, and simulations to describe its property.

6. **Trio-PGS**

   - I assume that the same model specifications from Trio-GWAS were used with the genotype terms replaced by the PGS. It would be helpful to provide explicit equations for both this and all other models so that the included covariates and effect interpretations are clear.

Response: We have now added the formal representations of these models in the methods section to clarify the included covariates and effect interpretations.

> - The use of robust (empirical) standard errors in this analysis is entirely appropriate (see my comment in Trio-GWAS), but using only the mother ID to define clusters is probably not reasonable as it treats half-siblings with the same father as independent. More generally, the comment above about trio selection applies as there could be complex relatedness between trios drawn from the same extended family (e.g., child in one trio is a parent in another, or first cousins who do not share mother or father IDs). The safest approach would be to estimate the kinship matrix with an ancestry-adjusted approach like PC-Relate and define clusters based on groups of individuals with pairwise kinship exceeding a certain threshold. How to set this threshold would depend on whether the model was believed to capture all genetic factors contributing to phenotype (i.e., residual clustering due only to shared environment) or not; these assumptions should be specified. The authors could also consider using the approach used in the Trio-GCTA analysis to get a subset of unrelated trios.

Response: Thank you for these suggestions. The clustering of standard errors accounts for the non-independence of family members, but has no impact on the point estimates (estimated associations). It is possible to cluster standard errors over a wider family group to allow for dependence across half-sibs, cousins etc, as suggested. However, this represents a trade-off: while more dependence due to genetic similarity may be controlled for i) the effectiveness of the control for full siblings with the same parents (i.e., the most highly related clusters) will necessarily diminish as the threshold for relatedness in a cluster is lowered; and ii) any confounding effects of being in the same household will be similarly poorly accounted for as more non-cohabiting relatives are introduced into a cluster.

We investigated clustering over a wider group using simulations as part of the sibling GWAS paper, while there was very modest type 1 error inflation (5.84% vs 5% nominal), there was little evidence of inflation for the within family model (4.94%)[1]. Therefore, this issue is unlikely to substantively affect our results. Furthermore, because of the sampling years of MoBa no offspring can be a parent of another.

With respect to the trio-PGS, we now additionally perform sensitivity analyses using only unrelated trios, described in the Methos section (page 20 lines 566-571) as follows:

"Estimates were calculated with robust standard errors with clustering on maternal IDs to account for the presence of siblings in the data. In sensitivity analyses to assess the sufficiency of this clustering strategy, we restricted to unrelated trios, removing at random all but one trio from families in which children were siblings, half-siblings, or first cousins – leaving a sample of 30,277 trios. To estimate effects without adjustment for parental indirect genetic effects, we re-ran models without parental PGS.".

The pattern of results is very similar, as now reported in the main text (page 6 lines 184-185), with results in new supplementary figures ("Sensitivity analyses confirmed that the inclusion of individuals related across trios was not responsible for driving the pattern of results (Supplementary Figures 6-7).")

7. **Trio-GCTA**

> ---------
> - Likelihood ratio tests for variance components are known to have non-standard null distributions (i.e., mixtures of chi square distributions with varying degrees of freedom)

because the null is on the boundary of the parameter space. See DOI 10.2307/2289471 for the seminal paper on this topic or documentation for SAS PROC GLIMMIX available on the web. It is unclear from the manuscript or accompanying code whether the appropriate null distribution was used. The authors should specify the null distribution used for each nested likelihood ratio test.

Response: Thank you for bringing this to our attention. We had been using a standard null distribution to compute *p*-values and have updated our code to use non-standard null distributions in the likelihood ratio tests for variance components using the same procedures as outlined in the SAS PROC GLIMMIX documentation referenced. This can be found in file trio_output_process_v2.R. For the likelihood ratio test between the full model and the no covariance model a single chi-square distribution with 3 degrees of freedom was used as the null distribution since the three parameters (covariances between the genetic effects) specified under the null hypothesis do not fall on the boundary of the parameter space. For the two-likelihood ratio test for variance components a mixture of distributions was used depending on the number of parameters specified under the null hypothesis that do fall on the boundary. This is two for the comparison between the no covariance model and the direct effects only model (variances of the maternal and paternal genetic effects) and one for direct effects only model compared to the null model (variance of the child genetic effects). In these cases, the following equation was used to calculate *p* values, where j is the number of parameters removed [41].

$$p = 2^{-j} \sum_{i=0}^{j} \binom{j}{i} Pr(\chi_i^2 \geq \hat{\lambda})$$

We have added the following text to the methods section (pages 20-21 lines 603-608) to specify the null distribution for each test: "For the likelihood ratio test, we calculated *p*-values using the classical procedure with a single chi-square null distribution when covariance component parameters were removed. When variance component parameters were removed, we calculated *p*-values using the equation below, where $\hat{\lambda}$ denotes the test statistic, and j the number of parameters removed so that the null distribution is a mixture of chi-square distributions dependent on *j*. [41,42]".

8. **Minor Comments**

- Monozygotic twins are zeroth-degree relatives (100% IBD) rather than first-degree relatives (50% IBD).

Response: Thank you for pointing this out. We have updated text and figures to indicate that monozygotic twins are zeroth-degree relatives.

- Please make sure to expand all acronyms at first use (e.g., MBRN).

Response: Apologies, some of the abbreviations first occurrence were moved to the supplemental text to meet word count limitations. As a lot of text has been moved around in the revision process we have gone through and tried to reduce the number of acronyms and made sure those kept are expanded at the first use.

Data Availability Statement

- Please specify the repository in which the summary statistics (presumably from Trio-GWAS) will be made available.

Response: We have added to the data availability statement that all summary statistics from GWAS

conducted in this study will be available on the MoBa website for GWAS summary statistics: https://www.fhi.no/en/ch/studies/moba/for-forskere-artikler/gwas-data-from-moba/.

Data Reporting

- Figures 2a, 2b, 3b, 3d, 4b; Supplementary Figures 5, 6: Error bars were not defined (SD? SE? CI?).
- Figure 2c, Supplementary Figure 2: Reference lines were not defined (one appears to be for 5e-8 genome-wide significance).

Response: Thank you, for pointing this out, we have fixed these errors.

Remarks on code availability:
I looked through the psychgen/MoBaPsychGen-QC-pipeline and psychgen/moba-trio-analyses GitHub repositories from the perspective of someone who might want to use or adapt the code. Because I do not have access to the underlying data, I cannot test run the code to verify that I can replicate the findings presented. Both repositories are useful for providing more detailed documentation of what was done than can be provided in a manuscript, but I would find it very challenging to use the code to replicate the analysis with the same data or to adapt the code for other purposes in its current state.

Response: Our primary goal for sharing the scripts on GitHub was to provide detailed documentation of what was done for transparency purposes. Further to this, we have updated the psychgen/MoBaPsychGen-QC-protocol repository to include an explicit list of external resources (both data and software) used throughout the project, and added the exact scripts used to perform the post-imputation QC.

Key objectives of this project were to (1) enhance the MoBa cohort with imputed genotype data as a resource that can be used by other researchers and (2) demonstrate exemplar within-family analyses to show the types of novel research questions that can be addressed using MoBa. However, packaging the MoBaPsychGen pipeline into a user-friendly software was beyond the scope of this project, primarily because it is bespoke to the unique features of MoBa (e.g., local data handling regulations requiring MoBa be stored and analyzed within specific secure research environments, thus using external imputation services, such as TOPMed, was impossible).

This approach aligns with the practices used in other large studies, which have provided excellent documentation but have not released a general open-source software tool(s) that can be readily applied to other datasets. Examples include UK Biobank[32] (https://biobank.ctsu.ox.ac.uk/crystal/ukb/docs/impute_ukb_v1.pdf), Mexico City Prospective Study[43] (https://github.com/mcps-analysts/mcps-genetic-cohort-profile), and FinnGen[44] (https://finngen.gitbook.io/documentation/methods/genotype-imputation, https://github.com/FINNGEN/).

To increase transparency we have restructured the https://github.com/psychgen/MoBaPsychGen-QC-pipeline repository, to include a more intuitive folder structure and organization of scripts, and enhanced the README files with more detailed explanations. We hope these updates make the resources more accessible and useful to researchers aiming to adapt or extend our work.

- The top-level READMEs do not give the user any indication of how to start using the codebase. A good example to follow is at https://github.com/PGScatalog/pgsc_calc, which gives specifics on the required execution environment, installation, testing, and running as well as links to further documentation.

Response: We agree and improved the top-level README file to clarify software dependencies, external resources (HRC and 1KG reference data, etc.) and computational environment (Services for Sensitive data from University of Oslo - https://www.uio.no/english/services/it/research/sensitive-data/index.html) where the MoBaPsychGen QC protocol was deployed and tested. The structure of files on MoBaPsychGen-QC-pipeline GitHub repository was reorganized as follows:

- imputation-jobs folder - scripts and SLURM jobs used for phasing and imputation (modules 5-7)
- pipeline-modules - documentation of specific steps in each module of the QC and imputation pipeline
- qc-scripts folder – various tools and scripts in R, Python, perl, bash, and SLURM jobs used in the QC part of the pipeline (modules 0-4) and post-imputation QC
- resources - overview of data resources used throughout the pipeline

The structure of files is now described in the root-level README file, and README files within each respective folder. We hope that this clarifies and makes it easier for the user to navigate through the large set of scripts that we developed and used as part of this project. In the README files, we clarify how individual modules or scripts which are self-contained can be reused in other projects. Overall, the MoBaPsychGen QC protocol had to handle several MoBa-specific aspects and complexities, was not tested on data other than MoBa, and has not been transferred to other computational environments apart from our secure environment. We therefore do not expect that our end-to-end protocol can be applied to other datasets without modifications.

> - A highly motivated user could probably piece the pipeline together using the written workflow in psychgen/MoBaPsychGen-QC-pipeline/qc-modules, but the authors should provide a single shell script or nextflow pipeline for each module as well as usage instructions regarding the required execution environment, inputs, and outputs. The READMEs in scripts/{R_scripts,python_scripts} of this repository are good examples of what should be provided elsewhere for the main scripts.

Response: As noted above, providing a user-friendly end-to-end software pipeline was beyond the scope of our work. For modules 0-4, an important consideration of any QC protocol is interrogating the output generated at the intermediate steps (e.g., we identified plate shifts - which would have resulted in the exclusion of a lot of individuals if an automated pipeline was used and specific results were not checked. For modules 5-7, given their complexity, a more automated end-to-end pipeline would require not only a single shell script / Nextflow/Snakefile or similar, but also extensive software infrastructure around it including synthetic data and test suites required for routine testing and validation, which our team had no capacity to implement. We have now re-arranged the files to have clearer structure, e.g. the MoBaPsychGen QC protocol scripts (qc_m1.sh and qc_m2.sh) are now located next to the workflow descriptions in the pipeline-modules folder, hoping that this new structure will make it easier for those interested in reviewing our QC protocol to identify codes relevant to their cohort.

> - Many of the scripts are not portable and use hardcoded paths to key files that would need to be changed by a downstream user. A portable script would accept required paths and other user-specific parameters as arguments.

As noted by the reviewer, we have already documented the inputs, outputs, and execution environment for individual tools used within the MoBaPsychGen QC protocol, including those found in the qc_scripts/*{R_scripts,python_scripts}* folders, which have no hard-coded paths. For more complex data pipelines, we have provided extensive documentation in the form of written workflows. Some modules of the QC protocol involved manual steps, such as selecting threshold parameters for individual batches and inspecting updates to the family structure generated by KING, particularly in the case of MZ twins and PO pairs within the parent generation. We believe this provides transparency of the MoBaPsychGen QC and imputation protocols applied in this project.

We acknowledge the reviewer's concern that many scripts are not portable due to the presence of hard-coded paths. However, we have retained the hard-coded paths to preserve the scripts in the exact form they were originally used, thereby ensuring reproducibility of the analyses. We recognize that this choice limits portability.
To address this, we have added the following text to Supplementary Note 3 (page 31 lines 1003-1012): "Furthermore, for transparency and reproducibility purposes, detailed documentation of our protocols and scripts used to perform QC, imputation and downstream analyses have been shared exactly as originally used, including hard-coded paths (see "Code Availability" statement). This approach was taken in alignment with other large studies, which have provided excellent documentation but have not released a general open-source software tool(s) that can be readily applied to other datasets[32,43,44]. We understand and acknowledge, that this is a limitation from a portability perspective. However, given the MoBaPsychGen pipeline is a tailored in-house protocol with many elements bespoke to MoBa, rather than as a generic open-source software pipeline, we do not anticipate other cohorts applying the pipeline in its current form.".

- Many scripts are heavily dependent on the execution environment but do not provide adequate instructions for configuring it. For example, the m1_qc.sh script above assumes that plink and R are available in PATH and does not state the versions required. A standard way to ensure a reproducible execution environment would be providing a Conda environment recipe with pinned versions/builds or using a Singularity/Apptainer image, as was done in some other scripts.
- It is unclear where to obtain required Singularity/Apptainer images or recipes to produce them.

Response: We've updated root-level README files with specific versions of the software used to perform the analyses, and provided download links to all external software. Some of the software we used (e.g. impute2, impute4 & shapeit2) has a proprietary license and cannot be re-distributed by us but can be fetched from the original source. Specific Singularity image files that were used in QC and imputation protocol can be fetched from an earlier version of CoMorMent containers (https://academic.oup.com/bioinformaticsadvances/article/4/1/vbae067/7667866; https://github.com/comorment/containers/releases/tag/v1.0.0). The latest version (https://github.com/comorment/containers/releases/tag/v1.9.1) now provides an Singularity image file which covers all software dependencies used in the MoBaPsychGen QC and imputation protocol (except for proprietary software), that can be used with the Apptainer or Singularity software on most HPC infrastructures.

1 Howe, L. J. *et al.* Within-sibship genome-wide association analyses decrease bias in estimates of direct genetic effects. *Nature Genetics* (2022). https://doi.org/10.1038/s41588-022-01062-7
2 Brandlistuen, R. E. *et al.* Cohort Profile Update: The Norwegian Mother, Father and Child Cohort (MoBa). *International Journal of Epidemiology* **54**, dyaf139 (2025). https://doi.org/10.1093/ije/dyaf139
3 Davies, N. M. *et al.* The importance of family-based sampling for biobanks. *Nature* **634**, 795-803 (2024). https://doi.org/10.1038/s41586-024-07721-5
4 Veller, C., Przeworski, M. & Coop, G. Causal interpretations of family GWAS in the presence of heterogeneous effects. *Proceedings of the National Academy of Sciences* **121**, e2401379121 (2024). https://doi.org/10.1073/pnas.2401379121
5 Veller, C. & Coop, G. M. Interpreting population- and family-based genome-wide association studies in the presence of confounding. *PLoS biology* **22**, e3002511 (2024). https://doi.org/10.1371/journal.pbio.3002511

6 Kong, A. *et al.* The nature of nurture: Effects of parental genotypes. *Science* **359**, 424-428 (2018). https://doi.org/10.1126/science.aan6877
7 Zhang, G. *et al.* Assessing the causal relationship of maternal height on birth size and gestational age at birth: A Mendelian randomization analysis. *PLOS Medicine* **12**, e1001865 (2015). https://doi.org/10.1371/journal.pmed.1001865
8 Young, A. I., Benonisdottir, S., Przeworski, M. & Kong, A. Deconstructing the sources of genotype-phenotype associations in humans. *Science* **365**, 1396-1400 (2019). https://doi.org/10.1126/science.aax3710
9 McEachan, R. R. C. *et al.* Cohort Profile Update: Born in Bradford. *International Journal of Epidemiology* **53**, dyae037 (2024). https://doi.org/10.1093/ije/dyae037
10 Milbourn, H. *et al.* Generation Scotland: an update on Scotland's longitudinal family health study. *BMJ open* **14**, e084719 (2024). https://doi.org/10.1136/bmjopen-2024-084719
11 Fitzsimons, E. *et al.* Collection of genetic data at scale for a nationally representative population: the UK Millennium Cohort Study. *Longitudinal and Life Course Studies* **13**, 169-187 (2022). https://doi.org/10.1332/175795921X16223668101602
12 Golding, Pembrey, Jones & The Alspac Study, T. ALSPAC–The Avon Longitudinal Study of Parents and Children. *Paediatric and Perinatal Epidemiology* **15**, 74-87 (2001). https://doi.org/https://doi.org/10.1046/j.1365-3016.2001.00325.x
13 Jaddoe, V. W. V. *et al.* The Generation R Study: Design and cohort profile. *European Journal of Epidemiology* **21**, 475-484 (2006). https://doi.org/10.1007/s10654-006-9022-0
14 Brumpton, B. M. *et al.* The HUNT study: A population-based cohort for genetic research. *Cell Genomics* **2**, 100193 (2022). https://doi.org/https://doi.org/10.1016/j.xgen.2022.100193
15 Gudbjartsson, D. F. *et al.* Large-scale whole-genome sequencing of the Icelandic population. *Nature Genetics* **47**, 435-444 (2015). https://doi.org/10.1038/ng.3247
16 Andersson, C., Johnson, A. D., Benjamin, E. J., Levy, D. & Vasan, R. S. 70-year legacy of the Framingham Heart Study. *Nature Reviews Cardiology* **16**, 687-698 (2019). https://doi.org/10.1038/s41569-019-0202-5
17 Cheesman, R. *et al.* How important are parents in the development of child anxiety and depression? A genomic analysis of parent-offspring trios in the Norwegian Mother Father and Child Cohort Study (MoBa). *BMC medicine* **18**, 284 (2020). https://doi.org/10.1186/s12916-020-01760-1
18 Huang, Q. Q. *et al.* Examining the role of common variants in rare neurodevelopmental conditions. *Nature* **636**, 404-411 (2024). https://doi.org/10.1038/s41586-024-08217-y
19 Nivard, M. G. *et al.* More than nature and nurture, indirect genetic effects on children's academic achievement are consequences of dynastic social processes. *Nature Human Behaviour* **8**, 771-778 (2024). https://doi.org/10.1038/s41562-023-01796-2
20 Torvik, F. A. *et al.* Modeling assortative mating and genetic similarities between partners, siblings, and in-laws. *Nature Communications* **13**, 1108 (2022). https://doi.org/10.1038/s41467-022-28774-y
21 Young, A. S. Estimation of indirect genetic effects and heritability under assortative mating. *bioRxiv*, 2023.2007.2010.548458 (2023). https://doi.org/10.1101/2023.07.10.548458
22 Tan, T. *et al.* Family-GWAS reveals effects of environment and mating on genetic associations. *medRxiv*, 2024.2010.2001.24314703 (2024). https://doi.org/10.1101/2024.10.01.24314703

23 Magnus, P. *et al.* Cohort profile update: The Norwegian Mother and Child Cohort Study (MoBa). *International Journal of Epidemiology* **45**, 382-388 (2016). https://doi.org/10.1093/ije/dyw029
24 Biele, G. *et al.* Bias from self selection and loss to follow-up in prospective cohort studies. *European Journal of Epidemiology* **34**, 927-938 (2019). https://doi.org/10.1007/s10654-019-00550-1
25 Rayner, C. *et al.* Quantifying and adjusting for selection biases in the Norwegian Mother, Father and Child Cohort Study using population-wide individual-level registry information. *OSF Preprints* (2025). https://doi.org/10.31219/osf.io/ymk37_v1
26 Conomos, Matthew P. *et al.* Genetic Diversity and Association Studies in US Hispanic/Latino Populations: Applications in the Hispanic Community Health Study/Study of Latinos. *The American Journal of Human Genetics* **98**, 165-184 (2016). https://doi.org/https://doi.org/10.1016/j.ajhg.2015.12.001
27 Jordan, E. *et al.* Genetic Architecture of Dilated Cardiomyopathy in Individuals of African and European Ancestry. *JAMA* **330**, 432-441 (2023). https://doi.org/10.1001/jama.2023.11970
28 Zhang, D., Dey, R. & Lee, S. Fast and robust ancestry prediction using principal component analysis. *Bioinformatics* **36**, 3439-3446 (2020). https://doi.org/10.1093/bioinformatics/btaa152
29 Privé, F., Luu, K., Blum, M. G. B., McGrath, J. J. & Vilhjálmsson, B. J. Efficient toolkit implementing best practices for principal component analysis of population genetic data. *Bioinformatics* **36**, 4449-4457 (2020). https://doi.org/10.1093/bioinformatics/btaa520
30 Coop, G. Genetic similarity versus genetic ancestry groups as sample descriptors in human genetics. *arXiv preprint arXiv:2207.11595* (2022).
31 Conomos, Matthew P., Reiner, Alexander P., Weir, Bruce S. & Thornton, Timothy A. Model-free Estimation of Recent Genetic Relatedness. *The American Journal of Human Genetics* **98**, 127-148 (2016). https://doi.org/https://doi.org/10.1016/j.ajhg.2015.11.022
32 Bycroft, C. *et al.* The UK Biobank resource with deep phenotyping and genomic data. *Nature* **562**, 203-209 (2018). https://doi.org/10.1038/s41586-018-0579-z
33 Appadurai, V. *et al.* Accuracy of haplotype estimation and whole genome imputation affects complex trait analyses in complex biobanks. *Communications Biology* **6**, 101 (2023). https://doi.org/10.1038/s42003-023-04477-y
34 Johnson, E. O. *et al.* Imputation across genotyping arrays for genome-wide association studies: assessment of bias and a correction strategy. *Human Genetics* **132**, 509-522 (2013). https://doi.org/10.1007/s00439-013-1266-7
35 Abecasis, G. R., Cardon, L. R. & Cookson, W. O. C. A General Test of Association for Quantitative Traits in Nuclear Families. *The American Journal of Human Genetics* **66**, 279-292 (2000). https://doi.org/https://doi.org/10.1086/302698
36 Gauderman, W. J. Candidate gene association analysis for a quantitative trait, using parent-offspring trios. *Genetic Epidemiology* **25**, 327-338 (2003). https://doi.org/https://doi.org/10.1002/gepi.10262
37 Wheeler, E. & Cordell, H. J. Quantitative trait association in parent offspring trios: Extension of case/pseudocontrol method and comparison of prospective and retrospective approaches. *Genetic Epidemiology* **31**, 813-833 (2007). https://doi.org/https://doi.org/10.1002/gepi.20243

38 Brumpton, B. *et al.* Avoiding dynastic, assortative mating, and population stratification biases in Mendelian randomization through within-family analyses. *Nature communications* **11**, 1-13 (2020).
39 Guan, J. *et al.* Family-based genome-wide association study designs for increased power and robustness. *Nature Genetics* **57**, 1044-1052 (2025). https://doi.org/10.1038/s41588-025-02118-0
40 Feeney, T., Hartwig, F. P. & Davies, N. M. How to use directed acyclic graphs: guide for clinical researchers. *BMJ* **388**, e078226 (2025). https://doi.org/10.1136/bmj-2023-078226
41 Verbeke, G. & Molenberghs, G. The Use of Score Tests for Inference on Variance Components. *Biometrics* **59**, 254-262 (2003). https://doi.org/10.1111/1541-0420.00032
42 SAS Institute Inc. *The Glimmix Procedure. in SAS/STAT 9.1: User's Guide*. 3233–3237 (SAS Institute Inc, 2004).
43 Ziyatdinov, A. *et al.* Genotyping, sequencing and analysis of 140,000 adults from Mexico City. *Nature* **622**, 784-793 (2023). https://doi.org/10.1038/s41586-023-06595-3
44 Kurki, M. I. *et al.* FinnGen provides genetic insights from a well-phenotyped isolated population. *Nature* **613**, 508-518 (2023). https://doi.org/10.1038/s41586-022-05473-8

Referee #1 (Remarks to the Author):

1. In Figure 4a the bars for Sleep duration and depression full model variance explained seem to be a bit off.

I have no further comments.

Response: Thank you for reviewing our responses to your previous comments. We appreciate your continued commitment to reviewing our manuscript.

Figure 4a shows the estimated variance components from trio-GCTA variance decomposition of MoBa children's height, educational achievement, sleep duration, and depressive symptoms, as we intended. In models that acknowledge covariances, the results do not constitute a simple variance decomposition into non-overlapping parts that sum directly to the total variance. The apparent "offset" of the bars for sleep duration and depression in the full model reflects negative covariance estimates between maternal/paternal indirect genetic effects and the child's direct genetic effect. Because these covariances are negative, they appear below the zero line. To make this clearer, we have added the following text to the figure legend:

"The proportion of variance explained by child, parental genetic effects and positive covariance between parental/child genetic effects, which inflates the total variability in the phenotype explained by genetic effects, are shown in stacked bars above zero. Negative covariance between parental/child genetic effects are shown in stacked bars below zero as these effects will deflate the total variability in the phenotype explained by genetic effects.".

Referee #2 (Remarks to the Author):

I co-reviewed this manuscript with one of the reviewers who provided the listed reports.

Response: Thank you for your continued contribution to co-reviewing our manuscript.

Referee #3 (Remarks to the Author):

Thank you for the opportunity to read this revision. All of my positive comments from my review of the initial submission stand and I will not repeat them here.

Response: Thank you for taking the time to review our revised manuscript. We greatly appreciate your continued positive assessment of the work.

I remain torn on the purpose of this manuscript. On the one hand it describes an exciting resource that will be of interest to a wide variety of scientists from many fields. This cannot be overstated in my opinion. But this description is fairly limited to genetic data and quality control information. Important, to be sure, but probably not of interest to the general scientist, versus the specialist. On the other hand, it describes a series of analyses that intend to "demonstrate... the

vast potential of genotyped family cohorts like MoBa for refining genomic discoveries, clarifying genetic overlaps, and investigating intergenerational transmission”.

Throughout the manuscript I felt like this goal was not quite achieved. The family design certainly provides exciting new avenues for investigation and analysis. But for each of the four phenotypes I was left wondering what we had learned about, say, intergenerational transmission? Maybe it is just a matter of telling the reader what they should take away from each of the analyses. My hunch is that the manuscript is currently limited by its laser focus on genetic information and analysis (perhaps because the paper began as a description of a genetic data resource), without consideration of phenotypic information and analysis crucial to interpretation of the genetic findings.

Response: We appreciate this thoughtful comment. We have clarified the primary purpose and focus of the manuscript: to introduce MoBa as a unique, large-scale genotyped family cohort and to demonstrate, using four exemplar phenotypes, how such data can be used to refine genomic discovery, clarify genetic overlaps, and understand intergenerational transmission. Rather than aiming to provide definitive substantive accounts of height, educational achievement, sleep duration, and depressive symptoms, we explicitly frame the analyses as illustrative demonstrations of the kinds of questions that can be addressed in MoBa and similar family cohorts.

First, we now explicitly state in the Abstract and Introduction that MoBa currently constitutes the largest sample worldwide of genotyped parents and offspring, and we position the analytic sections as methodological and conceptual exemplars of family-based genomic analyses, not comprehensive investigations of each phenotype.

Second, to address the concern about a laser focus on genetic and QC information, we emphasize more clearly that MoBa is an exceptional resource because genomic data are linked to a unique breadth of longitudinal phenotypic information. We have added a new Figure 1 that summarizes the longitudinal phenotypic data available in MoBa across developmental stages and domains, and we have revised the Introduction and Discussion to highlight how this rich phenotyping is crucial for interpreting genetic findings and for future work on health and functioning that goes beyond the examples presented here.

Third, in line with the suggestion to tell the reader what they should take away from each of the analyses, we have added summarizing take-home sentences for each analytic approach (trio-GWAS, polygenic score approaches, Trio-GCTA).

<u>Key revisions:</u>

The latter half of the Abstract now reads:

Page 2 line 19-30: “Here, we illustrate some of the advantages of familial data using the Norwegian Mother, Father, and Child Cohort Study (MoBa), the world’s largest genotyped cohort of parents and offspring (N≈230,000), with broad and longitudinal phenotyping of health and functioning. We provide an overview of MoBa and describe the quality control of genotype data tailored to this extensively related sample. We then use trio data to illustrate how family-based genomic designs can identify distinct direct and indirect sources of genetic influence and

structural confounding. As examples, we analyse children's height, educational achievement, depressive symptoms, and sleep duration. These demonstrations highlight MoBa as a broadly valuable resource for advancing understanding of health and functioning across the lifecourse and generations."

Introduction (highlighted text added):
Page 4, Line 77-98: "MoBa represents a uniquely powerful resource for family-based genetic analyses, offering a wide range of phenotypic measures on mothers, fathers, and offspring, collected from early pregnancy and spanning more than 25 years. Within the main data collections covering the first eighteen years of life alone (Figure 1a), MoBa has amassed observations on neurodevelopment, mental health, physical health, health behaviours and medication use, social and environmental exposures (Figure 1b), and broader aspects of functioning such as sleep, school achievement, and social participation.

Ongoing waves of data collection follow the children through adolescence and into early adulthood. The rich prospective and longitudinal observations are complemented with linkage to Norwegian registry data (Figure 1c). Norway has an extensive, high-quality health data infrastructure, including national health and administrative registries with continuous information on contacts, diagnoses, functioning assessments, treatments, care and support from the primary health and care services, the specialised health care services, educational and social services. MoBa also includes an extensive range of environmental data collected through repeated questionnaires and registry linkages, as well as diverse biological samples (including plasma, urine, and primary teeth), enabling integrated studies of genetic, environmental, and biological mechanisms across the lifecourse. Furthermore, numerous nested clinic studies in MoBa have collected additional diverse biological samples and deep phenotype data, further expanding the potential to derive insights into human health and functioning using family-based genetic analyses[43].

This potential is underpinned by the availability of the world's largest sample of genotyped parents and children[3]."

Figure 1 (see below) has been revised to clarify the breadth of the longitudinal phenotypic follow-up data available in MoBa, which is linked to the family-based genotype data. This illustrates the unique opportunities for genotype-phenotype mapping in MoBa.

Figure 1. Overview of phenotypic and genetic data availability among MoBa participants across the first 14-18 years of data collection

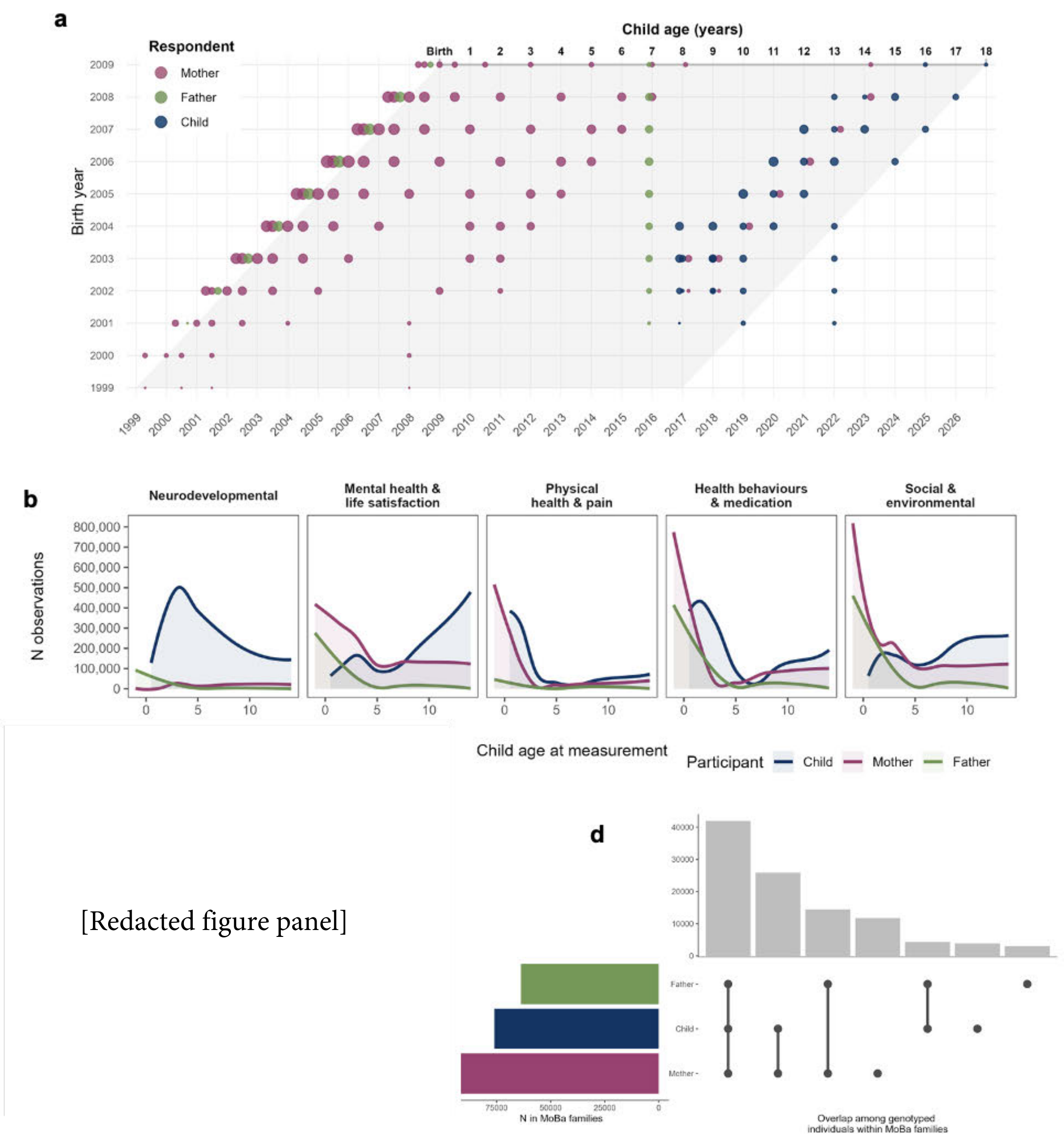

a, Core MoBa questionnaires by respondent and child year during the first 18 years, points scaled in size relative to proportion of the full baseline sample of participating MoBa children with data available at each data collection wave (participation rates are observed up to age 16 years, after which they are estimated based on latest available data at the time of publication); **b,** Summary of information obtained in different domains across core questionnaires administered in the first 14 years of core data collections; 'N observations' is calculated as the product of the number of responses by each participant type at each wave and the number of distinct measurement items in each domain at the same wave; **c,** Total N available in MoBa, including those with and without quality-control passing European ancestry genotype data currently available, with an indicative list of linkable nationwide registries; **d,** Overlap among genotyped individuals within families in panel C – NB, this is calculated at the level of individual MoBa children, meaning that genotyped mothers and fathers participating with more than one child are included more than once in these estimates;

Added take-home message for Trio-GWAS:

Page 6, line 179-185: "Taken together, these results show that a substantial proportion of SNP-based heritability and many reported SNP-trait and cross-trait associations in population-based GWAS, especially for educational achievement and depressive symptoms, reflect

indirect effects and between-family structure rather than direct genetic effects on offspring traits. Trio-GWAS in MoBa reveals smaller but more likely causal within-family genetic effects, altered patterns of genetic overlap with other traits, and fewer genome-wide significant loci, underscoring both the power and the necessity of family-based designs for refining genomic discovery."

Added take-home message for PGS methods:

Page 7-8, line 246-250: "Together, the results of the trio-PGS and PTDT analyses show that modelling parental genotypes, even in analyses using scores based on population-based GWAS, can reduce bias in the estimation of direct genetic effects. The results also clearly show that such bias can influence PGS-phenotype associations via maternal and paternal genotypes independently, highlighting the importance of trio data for solving this problem."

Take-home message for Trio-GCTA (highlighted text added):

Page 8-9, line 279-292: "The results suggest that when modelling genome-wide genetic effects in parent–offspring trios, children's own genotypes (direct effects) explain a substantial part of genetic variance for some traits (especially educational achievement and height), but not all of it. Ignoring parental genotypes can bias estimates of child genetic effects, particularly for educational achievement, where family genetics may be especially intertwined.

Inclusion of parental genetic effects in downstream genetic analyses – whether based on specific genetic propensities indexed by PGS or based on decomposition of all genetic variance in the trait – gives new insights into genetic mechanisms. Neither approach conclusively proves why children's traits associate with parental genotypes, so-called indirect 'effects' comprise many different sources of association[3,33], but both can demonstrate that the associations between children's own genotypes and phenotypes may be confounded. Moreover, both using specific genetic propensities (PGS) and genome-wide variation approaches can provide unbiased estimates of the direct effects of children's own genotypes on their phenotypes."

Conclusion:

Page 10, line 294-301: "Our work demonstrates the vast potential of MoBa, a genotyped family cohort with rich phenotype data, for refining genomic discoveries, clarifying genetic overlaps, and investigating intergenerational transmission. With large-scale genotyping of parents and offspring, deep longitudinal phenotyping, and linkage to comprehensive health and administrative registries, MoBa provides an unparalleled resource for family-based genetic analyses across health and functioning. Here, we have brought together and harmonised genotype data from multiple projects in MoBa and applied quality control procedures tailored to the unique characteristics of the sample."

I commend the authors on a significant and responsive revision, including responding to each of the ideas that I raised in my review. I found the actual changes to the manuscript itself to be quite minimal on several points about which I remain curious.

Response: We have made further changes to the manuscript as outlined below.

1. The issue of assortative mating. At the very least, as a reader I would expect a

discussion of AM in the methods and/or supplement, along with estimates of spousal phenotypic correlations (and, if the authors think it important, the correlation of polygenic scores) and how the models are robust (or not) to AM.

Response:

We appreciate this important point about assortative mating (AM) and have revised the manuscript to address this concern.

In Methods, we have clarified that the trio methods estimation of direct genetic effects are robust to assortative mating (and population stratification), however, the estimation of indirect genetic effects are not:

Page 21-22, line 595-600: "The term "indirect genetic effects" is used for consistency with the field, despite many methods being agnostic as to the specific mechanism, which can include: indirect effects of parents, population structure (the presence of subgroups within a population that can bias genetic associations), and assortative mating (the tendency for individuals with similar traits to mate non-randomly, influencing genetic associations in the next generation) among other sources."

Page 24, line 711-715: "Whereas trio estimates of direct genetic effects tend to be robust to assortative mating and population structure, estimates of indirect genetic effects can reflect not only genetic nurture effects, but also assortative mating and population structure. To aid interpretation of the potential impact of assortative mating on the indirect genetic effects estimates, we calculated spousal correlations for the four PGS used in the trio-PGS and PTDT analyses."

Supplementary Table 1. Spousal PGS correlations with 95% confidence intervals.

		Father				
		**Height**	**BMI**	**Education**	**Smoking**	**Depression symptoms**
**Mother**	**Height**	0.064 (0.054, 0.073)	-0.007 (-0.017, 0.002)	0.017 (0.008, 0.027)	0.002 (-0.007, 0.012)	-0.016 (-0.025, -0.006)
	**BMI**	-0.012 (-0.022, -0.003)	0.042 (0.032, 0.051)	-0.059 (-0.069, -0.050)	0.012 (0.003, 0.022)	0.021 (0.011, 0.030)
	**Education**	0.040 (-0.030, 0.049)	-0.057 (-0.066, -0.047)	0.141 (0.131, 0.150)	-0.048 (-0.057, -0.038)	-0.034 (-0.045, -0.026)
	**Smoking**	-0.006 (-0.016, 0.003)	0.024 (0.014, 0.033)	-0.050 (-0.059, -0.040)	0.034 (0.025, 0.044)	0.025 (0.016, 0.035)
	**Depression symptoms**	-0.010 (-0.020, -0.001)	0.023 (0.014, 0.033)	-0.045 (-0.054, -0.035)	0.025 (0.016, 0.035)	0.018 (0.008, 0.027)

Page 7, line 242-245:"Spousal PGS correlations indicated modest within-trait assortative mating for the PGS included in our analyses, most pronounced for educational attainment (r ≈ 0.14) and weaker for height, BMI, smoking, and depressive symptoms (r ≈ 0.02–0.06) (Supplemental Table 1)."

Conclusions section:

In the Conclusions, we highlight AM as one of several demographic processes that can bias estimates of indirect genetic effects and intergenerational associations. We emphasize that even with trio data, estimates of indirect genetic effects may still be confounded by AM, population stratification, dynastic effects, and genetic influences from relatives beyond parents. Further, we describe that progress will require triangulation across multiple genetic designs and family structures (e.g., mediation analyses, extended pedigrees, explicit assortative mating modelling), and leveraging increasingly large within-family GWAS that can more directly mitigate confounding from AM and population stratification:

Page 10, line 315-330 (additions highlighted):"Estimates of direct genetic effects using trio data should be reasonably robust to all the sources of indirect effects, and therefore allow for prioritization of causal variants and effects sizes. However, estimates of *indirect* genetic effects may reflect varied components, including not only genetic nurture, but also broader demographic factors, such as assortative mating, population stratification, dynastic effects, and indirect genetic effects from relatives other than parents. Strategies to disentangle these factors will involve triangulation across genetic designs – including those demonstrated here, and others such as mediation analyses (e.g., Cheesman et al. 2020[51]; Huang et al., 2024[52]), extended pedigrees (e.g., Nivard et al., 2024[53]), and assortative mating modelling (e.g., Torvik et al., 2022[40]), as well as across multiple sources of family genetic data. Systematic investigation of assortative mating across cohorts, countries, and historical periods will be important for understanding how population-level mating patterns shape indirect genetic effects and their generalisability. Furthermore, as within-family GWAS incorporating samples like MoBa become larger and more powerful, the potential to directly mitigate and control for confounding from assortative mating and population stratification in downstream within-family analyses will also increase."

These sections in the manuscript show our assumptions and limitations regarding AM and clarify how future research using MoBa and other family-based cohorts can build on our work to address AM more rigorously.

We do not include phenotypic spousal correlations because parental phenotypes are not used in the manuscript's analyses, and we do not have equivalent parental measures for all the analyzed child phenotypes.

2. The measurement/etiology issue, and how it is affected by development (age) and cohort (generation). I have two suggestions.
2a. First, please add information about how the measurements were taken for the four phenotypes. Currently, one has to dig to figure out that "educational achievement" is actually GPA in fifth grade (versus years of education on which the PGS is based) and what the exam is that is used for the PDTD analysis. As another example, are the children reporting on their own sleep and depressive symptoms? It was not until page 7 (line 224) that I found it mentioned that the mother is reporting on the child's symptoms, but this is not stated elsewhere.

This complicates interpretation. I recommend adding a section dedicated to describing all measures used.

Response:

We have now added a paragraph early in the manuscript describing how the measurements were taken, along with an added Table 1 showing descriptive and correlational information:

Page 4, lines 110-118: "Here, we demonstrate the valuable insights that can be gained by incorporating maternal and paternal genotypes in the investigation of genetic effects on health and functioning. We apply a range of trio-based methods to four example offspring phenotypes spanning different domains of health and functioning, all measured in middle childhood. Children's height (age 7 years), sleep duration (age 7 years), and depressive symptoms (age 8 years) were reported by mothers in MoBa questionnaires, while educational achievement was assessed using a composite score of numeracy, reading, and English skills derived from the educational registry's mandatory national tests in grade five (age 10 years). Descriptive information for these measures is presented in Table 1."

Table 1. Descriptive statistics and correlation matrix for the four middle childhood phenotypes.

	Phenotype	N*	Mean (SD)	Range	1	2	3	4
1	Height (cm)	39,605	126.74 (5.77)	106-149	1.00			
2	Educational achievement (composite test score)	68,538	57.68 (16.84)	0-100	0.040 (0.030, 0.050)	1.00		
3	Sleep duration (hours)	40,625	10.12 (0.72)	8-12	-0.020 (-0.030, -0.010)	-0.005 (-0.015, 0.005)	1.00	
4	Depression symptoms (SMFQ)	30,795	1.72 (2.13)	0-11	0.016 (0.005, 0.027)	-0.085 (-0.096, -0.074)	-0.065 (-0.076, -0.053)	1.00

Note: Height and sleep duration were maternally reported at age 7 years, and depression symptoms at age 8 years (SMFQ = Short Mood and Feelings Questionnaire). Educational achievement was assessed as a composite of national test scores in numeracy, reading and English at age 10 years. Scaled scores (0–100) are presented because the raw score ranges differed across test years; rescaling provides a consistent, comparable metric across test years. All the four phenotype scores were standardized for use in the trio analyses to place them on a common scale and to facilitate interpretation and comparison of effect sizes. *Genotyped.

Added to Methods:

Page 20, lines 524-553: "As shown in Figure 1 Panel A, MoBa includes questionnaire data collections at many time points, including during the pregnancy, infancy, preschool-age, school-age, adolescence and beyond. Further details about the cohort representativeness and participation in specific waves of data collection are described elsewhere[43,54-56]. Detailed

instrument documentation is available on the MoBa website (https://www.fhi.no/en/ch/studies/moba/). A wide range of national health and administrative registries can be linked to MoBa for further phenotyping without reliance on participant engagement in completing questionnaires (Figure 1, Panel B).

Phenotypic measures used in the present analyses

For our exemplar analyses, we included four phenotypes assessed in middle childhood (age 7-10 years), spanning physical health (height), mental health (depression symptoms) and aspects of functioning (sleep duration and educational achievement). Child height at age 7 years was reported by mothers in the age-7 questionnaire for the question “What is the child’s height and weight now at 7 years old?”. Mothers were asked to report their child’s current height in centimetres. Sleep duration at age 7 years was maternally reported in the 7-year-questionnaire on the item “Approximately how many hours does the child usually sleep on a weeknight?” with response categories 1) 8 hours or less, 2) 9 hours, 3) 10 hours, 4) 11 hours, 5) 12 hours or more. Depressive symptoms were measured using the 13-item Short Mood and Feelings Questionnaire[82] (SMFQ) reported by mothers in the 8-year-questionnaire. We prepared the questionnaire-assessed phenotypes using the phenotools R package (0.2.8) in R 4.1.0 (R Core Team, 2022). Educational achievement at age 10 was assessed as a composite score based on performance on three national standardized tests of skills in literacy, numeracy and English, derived from the Statistics Norway Educational Registry. The national tests are administered in the autumn of grade 5 (age 10), 8 (age 13) and 9 (age 14), and are mandatory with exemptions only upon application on the grounds that the results will not be useful for assessing the child's learning due to disability or lack of knowledge of the Norwegian language. Because the raw score ranges differed across subjects and test years, the raw scores were standardized within each test year and subject test. All the four phenotype scores were standardized for use in the trio analyses to place them on a common scale and to facilitate interpretation and comparison of effect sizes.”

2b. Second, please provide more information at the phenotypic (versus genetic) level. The genetic analysis is interesting, but without more information about the phenotypes (means and variances for children and parents [and others if relevant] pairwise relative correlations, etc.) they become quite difficult to interpret and understand. I would suggest that integrating phenotypic information directly into the current text and figures is crucial, versus punting it deep within the supplement. Or, if there is some strong scientific reason for completely divorcing the genetic and phenotypic information in this manuscript, it would be helpful to explain that to the reader.

Response:

We agree that a richer phenotypic context is key for interpreting the genetic analyses and appreciate this clear recommendation. As shown in response to your comment above, we have incorporated more information about the phenotypes used in the analyses into the main text, including means, standard deviations and score ranges, as well as pairwise correlations between the different phenotypes (Table 1), and substantially expanded Figure 1 to highlight the value of integrating phenotypic information with genotypes.

Finally, some very minor suggestions you may consider.
3. Page 3 lines 41 to 45. This is a great definition of indirect and direct effects. On my read it tends to conflate direct effects with proximal effects and indirect effects with distal effects. For example, either could operate through environmentally- or socially-mediated pathways. (Also - do indirect effects not also routinely include population stratification.)

Response: We have revised the explanation of direct and indirect genetic effects from " increasing evidence suggests that genetic associations estimated from unrelated samples reflect not only direct genetic effects - where an individual's genetic variants causally influence their own phenotype - but also correlated factors, such as indirect genetic effects on an individual's phenotype arising from the genetic influence of another individual through environmental or social processes", as follows:

Page 3, lines 40-48: "increasing evidence suggests that genetic associations estimated from unrelated samples reflect not only direct genetic effects - where an individual's genetic variants causally influence their own phenotype - but also indirect effects of other individuals' genotypes, such as parental genotypes shaping the rearing environment and thereby influencing offspring outcomes[31,32]. Estimates of indirect genetic effects in parent-offspring models capture the association between parental genotypes and offspring outcomes after conditioning on the offspring's genotype. These estimates reflect "genetic nurture" effects, as well as components arising from broader population structure, such as assortative mating and population stratification."

4. Reference 47, Karlsson-Linner did not conduct a GWAS of smoking. They used results from an existing study. I presume you then must have used results from this existing study: Liu et al. (2019) Association studies of up to 1.2 million individuals... Nature Genetics, 51, 237-244.

Response: We confirm that reference for the smoking GWAS summary statistics used in our manuscript is accurate. The smoking GWAS summary statistics used for the analyses was accessed through the genotools R package (https://github.com/psychgen/genotools). The smoking GWAS summary statistics file included in genotools is "EVER_SMOKER_GWAS_MA_UKB_TAG.txt". This file was generated in the Karlsson Linnér, R., et al. (2019) paper and is available to download through the Social Science Genetic Association Consortium (SSGAC) Data Portal (https://thessgac.com/), accessible through the SSGAC website by selecting Data under the Research tab (https://www.thessgac.org/). The meta data showing the reference and data source for the "EVER_SMOKER_GWAS_MA_UKB_TAG.txt" file is available in the genotools metadata file: https://github.com/psychgen/genotools/blob/main/data/pgs_metadata.rda.

We took the conservative approach of using these smoking GWAS summary statistics instead of the ones generated in the Liu, M., et al. (2019) paper to ensure there is no sample overlap; given that the Norwegian HUNT sample is included in the Liu, M., et al. (2019) results and there is the possibility that individuals participated in both MoBa and HUNT.

5. This is a minor stylistic question, but I became confused throughout by what is meant by "genetic propensities". In some places it seems to simply mean "score on the PGS", but there are some places where both terms are used in the same sentence and they seem to be used to mean different things. Is there an important distinction to be made here?

Response:

Thank you for pointing this out. We have changed to PGS to be more precise and consistent.

6. Consider revising the description in the main text and the legend and axis labels of Figure 3 of the PTDT method. It is difficult to follow and no interpretation is given. At the end of the paragraph (page 7 line 205) can you tell the reader why these results are useful or interesting? As it is now, the reader is left to figure that out on their own.

Response: We have revised the description of the PTDT analyses as follows (changes highlighted):

Page 7, line 213-241: "Polygenic transmission disequilibrium (PTDT; Figure 3d) examines whether children selected for a given phenotype, for example tall height, receive a higher (or lower) polygenic load for this phenotype from their parents[39]. Under Mendelian inheritance, a child's PGS should, on average, be equal to the mid-parent PGS. If the phenotype is influenced by genetic variants in the PGS, then children selected for extreme values on that phenotype should show *over-transmission* of the relevant PGS (on average, the children should have higher PGS than expected based on the average of their parents' PGS). If, instead, the PGS-phenotype association is due to structural confounding (e.g., from population stratification) that also influences parental PGS, this pattern of over-transmission would be absent or attenuated. Importantly, PGS of *unselected* siblings should not exceed the mid-parent average – regardless of the relationship between the PGS and the phenotype of selection.

For these analyses (Figure 3e), we used subsets of MoBa children based on: extreme height (>2 standard deviations [SDs] above the sample mean; N=527, plus N=84 unselected sibling controls), educational achievement above the 90th percentile, (N=3,227, $N_{siblings}$=535), sleep duration below the 10th percentile (N=438, $N_{siblings}$=78), and extreme depressive symptoms (>2SDs above the sample mean; N=618, $N_{siblings}$=95). Using PTDT, we observed significant over-transmission of PGS for height among extremely tall children (p_{FDR}<0.001) and educational attainment among children with high educational achievement (p_{FDR}<0.03). In both cases, siblings did not show evidence of similar over-transmission (Supplementary Figure 8), although estimates were considerably less precise. Neither sleep duration nor depressive symptoms PTDT analyses provided robust evidence for over- or under-transmission of any of the PGS. These findings suggest that, for height and educational achievement, the PGS signal reflects within-family genetic transmission rather than solely between-family confounding, strengthening causal interpretation of these polygenic influences. In contrast, the lack of clear over-transmission for sleep duration and depressive symptoms suggests that current PGS for these traits either capture weaker within-family genetic effects or are more strongly influenced by confounding and measurement error, highlighting areas where larger discovery samples and improved phenotyping are needed."

We have also clarified the legend and axis labels of Figure 3 of the PTDT method.
The new axes now read (new text highlighted):
Page 18: "Relative over- or under-transmission of PGS-trait associated alleles (unit is mid-parent PGS SDs) PGS trait (bars) and group as ascertained on the basis of children's observed trait scores (panels)"

Legend:

"e, Polygenic transmission disequilibrium, indexed by systematic differences in polygenic scores – as compared to a calculated mid-parental average polygenic score – among children selected for, respectively, height 2 standard deviations (SDs) above the mean, exam performance above the 90th percentile, sleep duration below the 10th percentile, and depressive symptoms 2 SDs above the mean. Over-/under-transmission relative to the mid-parental average polygenic score is usually interpreted as evidence of genetic influence on a child trait free from confounding by structural confounding such as population stratification or assortative mating. The error bars in plots b, c, and e are 95% confidence intervals."

7. You are using three fit indices in the Trio-GCTA, and these fit indices disagree. I find that is not uncommon. If you can find a way to show the three index values for all the models you considered that I would think provides more useful information than what is currently in Figure 4b, which is a small slice of the model selection space considered here.

Response:
Thank you for this helpful suggestion. In the revised manuscript we now report all three fit indices (AIC, BIC, and LRT p-values) for all four Trio-GCTA models and all phenotypes in Supplementary Table 2. This allows readers to see the full model selection space, rather than only the subset of comparisons shown in Figure 4b.

Revised paragraph reporting the fit indices:
Page 8, line 260-268: Trio-GCTA provided clear evidence for genetic effects on height and educational achievement, and statistically detectable but smaller genetic contributions to sleep duration and depressive symptoms (Figure 4a). For educational achievement, the best-fitting model included covariances between maternal, paternal, and offspring genetic effects, whereas for the other phenotypes a simpler model without covariances sufficed. Likelihood ratio tests indicated that parental genetic variance components improve model fit for height (p=0.03), sleep duration (p=3.4 x 10-4), and depressive symptoms (p=0.02) but the estimated indirect effects are modest and imprecise and the contrast between AIC and BIC (Supplementary Table 2) suggests only weak evidence for indirect effects.

Supplementary Table 2. Fit statistics for the Trio-GCTA models.

Model	AIC	BIC	LRT p *
Education achievement			
Full	58276.81	58695.56	NA
No Covariance	58300.62	58695.21	1.51548E-06
Direct	58319.68	58698.16	3.24697E-06
Null	58762.72	59133.15	4.3236E-99
Height			
Full	76595.85	76989.11	NA
No Covariance	76592.03	76963.03	0.535196321
Direct	76593.45	76949.61	0.026650699
Null	76786.42	77135.15	1.30917E-44
Sleep duration			
Full	35939.09	36334.31	NA
No Covariance	35938.61	36311.46	0.137414041
Direct	35948.47	36306.41	0.000343215
Null	35956.96	36307.45	0.000598106
Depressive symptoms			
Full	29946.34	30332.54	NA
No Covariance	29941.45	30305.79	0.773910139
Direct	29943.39	30293.15	0.020313847
Null	29942.02	30284.5	0.212104802

Note: When AIC or BIC were inconclusive LRT test was used as the deciding factor for the best fitting model. *Comparison to model in previous row. When variance components removed, p-values calculated with a null distribution that is a mixture of chi-square distributions (see methods).

8. Is there inertia in the title (and abstract)? No strong opinions here but "Health and development" do not seem to be core features of the current text. Although I suppose Nature editors will make title suggestions upon acceptance.

Response:
We appreciate this comment and agree that the previous emphasis on "health and development" did not fully capture the focus of the manuscript. We have therefore revised the title and text to centre on "health and functioning", which better reflects both the breadth of MoBa phenotyping, and the four example outcomes analysed (height, sleep duration, depressive symptoms, and educational achievement). MoBa includes detailed and longitudinal information on participants' functioning from repeated questionnaires (e.g., sleep, behaviour, wellbeing, and school and work functioning) and from continuously updated registry linkages (e.g., educational achievement, health service use, and functioning assessments according to the WHO International Classification of Functioning, Disability and Health [ICF]).

We are of course open to further editorial guidance.

Specific edits made:

Title and text: Throughout, we have replaced “health and development” with “health and functioning”.

Introduction:

Page 4, lines 82-83: We replaced “and more” in “MoBa has amassed observations on neurodevelopment, mental health, physical health, health behaviours and medication use, social and environmental exposures, *and more*” with the more specific “broader aspects of functioning such as sleep, school achievement and social participation”.

Thank you again for considering the above.

I am in the habit of signing my reviews,

Scott Vrieze
University of Minnesota

Referee #3 (Remarks on code availability):

I commend the authors for posting their code and encourage them if they have not already to making sure the code runs, perhaps with example toy datasets.

Response: Thank you for your positive comment on our code sharing. Our primary goal in sharing the scripts from the MoBaPsychGen QC pipeline (https://github.com/psychgen/MoBaPsychGen-QC-pipeline) and downstream exemplar trio analyses (https://github.com/psychgen/moba-trio-analyses) repositories was to provide detailed documentation of what was done to ensure transparency and reproducibility. Because the scripts were developed to run directly on the study datasets within the specified secure research environments, in accordance with MoBa's local data handling regulations, they cannot be meaningfully demonstrated using bespoke toy datasets. Creating synthetic or toy datasets that accurately reflect the structure and complexity of MoBa was beyond the scope of this project. We also believe that processing other datasets will require substantial adaptations to account for their technical and demographic nuances. Consequently, we did not aim to develop a generic pipeline that would be readily applicable to other studies.

This approach is consistent with other large studies, which likewise prioritised comprehensive documentation without releasing toy or synthetic datasets. Examples include the UK Biobank (https://biobank.ctsu.ox.ac.uk/crystal/ukb/docs/impute_ukb_v1.pdf), the Mexico City Prospective Study (https://github.com/mcps-analysts/mcps-genetic-cohort-profile), FinnGen (https://finngen.gitbook.io/documentation/methods/genotype-imputation, https://github.com/FINNGEN/), and the Taiwan Precision Medicine Initiative (https://github.com/HsinChouYang/TPMI_Cohort/).

In line with this standard practice, we confirm that the analytic code in https://github.com/psychgen/MoBaPsychGen-QC-pipeline and

https://github.com/psychgen/moba-trio-analyses repositories runs as provided within the appropriate secure research environments for which they were designed.

Comments on the response to reviewer #4 by Reviewer #3:

1. They find the responses ok, even if not always satisfying, but understandable for practical reasons. With regards to batch effects, they would not suggest a different imputation approach but they would like to have information about the provenance of each batch in the big supplementary table 1 devoted to batches (incl. purpose of that batch, i.e. was it a case-control study of a specific disorder? Did it focus only on complete trios? Was there something different about the tissue collection? and so on.), since this could help a reader understand how the batches differ.

Response: Thank you for commenting on our responses to Reviewer #4's comments.

The full selection criteria of each genotyping batch are described in text in "Supplementary Methods 2 – Genotyping of MoBa". In the original submission we included only the "Basic selection criteria" in Supplementary Table 3 (previously Supplementary Table 1) to prevent the table from becoming overly large.

However, we agree that including the full selection criteria directly in Supplementary Table 3 (previously Supplementary Table 1) improves accessibility for readers and clarifies how batches differ. As such we have expanded Supplementary Table 3 (previously Supplementary Table 1) to include information about the provenance of each batch. Specifically, we have renamed the "Basic selection criteria" column to "Selection criteria" and added detailed information on the selection criteria for each batch. Therefore, the table now provides a comprehensive overview of differences, covering not only technical factors (e.g., genotyping array, chip, and centre), but also complete information on batch-specific selection criteria.

Regarding tissue source, all batches used DNA extracted from blood samples. Therefore, we have not added a separate column for tissue collection.

2. They also feel that you should include some of your explanations for why you chose a bespoke processing pipeline into the supplement, again, so that a reader understands why you made certain choices.

Response: We agree that it is important for readers to understand the rationale underlying our decision to implement a bespoke processing pipeline. To address this, we have expanded Supplementary Note 1 to more explicitly describe the reasoning behind the development of the MoBaPsychGen pipeline as follows (new text in italics):

Page 31, lines 974-983: "We were unable to use a pre-existing pipeline for the Norwegian Mother, Father, and Child Cohort Study (MoBa) due to (1) the cohort size, (2) the complex interrelatedness, and (3) the idiosyncrasies of MoBa (e.g., inclusion of within and across batch duplicates and the large imbalance in number of individuals clustering with reference superpopulations). *Therefore, we developed the MoBaPsychGen pipeline to allow manual inspection and benchmarking of QC performance across the various pipeline modules in the*

context of a large-scale, highly related cohort, with imbalanced batches and population structure. Future work is required to automate the process based on empirical insights gained during the development and implementation of the pipeline, particularly to meet requirements such as re-running of various stages following consent withdrawals."

Similarly, we have added text to Supplementary Note 2 – Additional results information to clarify why ancestry adjusted QC approaches were not applied to MoBa (new text in italics):

Page 32, lines 991-998: "Approximately 95% of genotyped participants clustered with the 1000 Genomes European superpopulation. *The large imbalance of individuals clustering with the 1000 Genomes European superpopulation resulted in small numbers of individuals clustering with the African, Admixed American, East Asian, and South Asian 1000 Genomes superpopulations, which were spread across the 26 genotyping batches. This made it incredibly challenging to apply ancestry adjusted QC approaches to MoBa, given some genotype batches included <5 individuals clustering with specific superpopulations."*

In addition, Supplementary Note 3 – Additional pipeline discussion further details the QC steps that were of particular importance for MoBa and the limitations of the pipeline, including that "the MoBaPsychGen pipeline is a tailored in-house protocol with many elements bespoke to MoBa, rather than as a generic open-source software pipeline" (page 35, lines 1132-1134).

Throughout the Supplementary Methods, we also clarify the rationale behind pipeline decisions that were bespoke to MoBa. Including, the ancestry assignment methodology (Supplementary Methods 4, page 47), PCA projection methodology (Supplementary Methods 5, page 48), the relatedness inference approach (Supplementary Methods 8, page 49), and the strategy to handle phasing and imputing 26 genotyping batches (Supplementary Methods 16, page 51).

Comment from the Editor

Further, in the data availability statement, we request that you add response time for access requests and detail any access restrictions (i.e. what type of research can be done with these data).

Response:

We have added to the Data availability statement on Page 25, Line 726-734:
"MoBa can be used for health research. The purpose of MoBa is to generate new knowledge about the causes, risk factors, courses, and consequences of diseases and health outcomes, with the ultimate goal of improving prevention and treatment. The MoBa administration normally uses 3–5 weeks to reach a decision from the time a complete application has been received. The expected processing time from decision is in place until data delivery is normally between 4 and 5 weeks. Delivery of linked data from health registries usually takes up to 8 weeks. Updated information about processing time is available on the MoBa website (https://www.fhi.no/en/ch/studies/moba/for-forskere-artikler/research-and-data-access/)."

Response to reviewer #3

Reviewer #3:
This is a third review of this manuscript. I very much appreciate the care with which the authors have continued to humor my suggestions. Thank you kindly. I apologize for the delay in providing this review.

I have no new suggestions or concerns. I continue to believe that the addition of information about phenotypic similarity among relatives is crucial to interpret the genetic analyses. But I do not see it as my role as reviewer to insist on such a thing so will decline any further review request related to this issue.

Many thanks for your work on this important research enterprise. I am convinced it will fuel important work in multiple disciplines, including medicine, psychology, human genetics, and methodological development.

I am in the habit of signing my reviews.

Collegially,

Scott Vrieze
University of Minnesota

Referee #3 (Remarks on code availability):

I have not reviewed the code base.

Response:

We are grateful for the reviewer's thoughtful engagement across multiple rounds and for highlighting the importance of phenotypic information among relatives for interpreting the genetic analyses. In response, we now include:

In the main manuscript (pages 4-5, lines 118–122):

"To provide additional context for interpreting the offspring genetic and phenotypic results, descriptive statistics and Pearson correlations for phenotypically similar outcomes to the four offspring phenotypes are presented for mothers and fathers in Extended Data Table 1. Spousal Pearson correlations are presented in Extended Data Table 2."

In the methods (page 18, lines 657-676):

" Parental phenotypes were not used in the analyses presented in this manuscript. However, similar outcomes in parents to the four exemplar offspring phenotypes were

defined to provide supplementary context for interpreting the offspring genetic and phenotypic results. Parental height was self-reported in centimetres in response to the question “How tall are you?”. Educational achievement was derived from Statistics Norway data corresponding to the International Standard Classification of Education (ISCED)[59] definitions. The ISCED levels from 0-8 corresponding to “early childhood education (‘less than primary’)”, “Primary education”, “Lower secondary education”, “Upper secondary education”, “Post-secondary non-tertiary education”, “Short-cycle tertiary education”, “Bachelor’s or equivalent level”, “Master’s or equivalent level”, and “Doctoral or equivalent level” were coded as 1, 7, 10, 11, 13, 14, 16, 18, and 21 years of education, respectively. Sleep problems were self-reported by mothers when the child was 14 years old and by fathers in 2015-2016, using the total score of three items modified from the Karolinska Sleep Questionnaire[60]: “How often do you find it difficult to get to sleep at night?”, “How often have you woken up repeatedly during the night?”, and “How often do you feel tired or sleepy during the day?”. The response options “Never”, “Less than once a week”, “Once per week”, “Twice per week”, “Three times per week”, and “Four times or more per week”, were coded from 0-5, respectively. Parental depression symptoms were self-reported by mothers at about week 30 of pregnancy and fathers around week 15 of pregnancy, using the depression subscale of the short eight-item Hopkins Symptoms Checklist (SCL-8)[61].”
